# Duplication and population dynamics shape historic patterns of selection and genetic variation at the major histocompatibility complex in rodents

**DOI:** 10.1002/ece3.567

**Published:** 2013-04-22

**Authors:** Jamie C Winternitz, John P Wares

**Affiliations:** 1 Odum School of Ecology, University of GeorgiaAthens, Georgia, 30602; 2 Department of Genetics, University of GeorgiaAthens, Georgia, 30602

**Keywords:** Balancing selection, gene duplication, major histocompatibility complex, *Microtus montanus*, population dynamics, purifying selection

## Abstract

Genetic variation at the major histocompatibility complex (MHC) is vitally important for wildlife populations to respond to pathogen threats. As natural populations can fluctuate greatly in size, a key issue concerns how population cycles and bottlenecks that could reduce genetic diversity will influence MHC genes. Using 454 sequencing, we characterized genetic diversity at the DRB Class II locus in montane voles (*Microtus montanus*), a North American rodent that regularly undergoes high-amplitude fluctuations in population size. We tested for evidence of historic balancing selection, recombination, and gene duplication to identify mechanisms maintaining allelic diversity. Counter to our expectations, we found strong evidence of purifying selection acting on the DRB locus in montane voles. We speculate that the interplay between population fluctuations and gene duplication might be responsible for the weak evidence of historic balancing selection and strong evidence of purifying selection detected. To further explore this idea, we conducted a phylogenetically controlled comparative analysis across 16 rodent species with varying demographic histories and MHC duplication events (based on the maximum number of alleles detected per individual). On the basis of phylogenetic generalized linear model-averaging, we found evidence that the estimated number of duplicated loci was positively related to allelic diversity and, surprisingly, to the strength of purifying selection at the DRB locus. Our analyses also revealed that species that had undergone population bottlenecks had lower allelic richness than stable species. This study highlights the need to consider demographic history and genetic structure alongside patterns of natural selection to understand resulting patterns of genetic variation at the MHC.

## Introduction

Genetic variation provides the potential for natural populations to adapt to environmental changes, including those caused by global climate change, habitat alteration, and novel pathogens. However, adaptive genetic variation buffering wild populations against abiotic and biotic threats is itself vulnerable to fluctuations in population size and population connectivity (Saccheri et al. 1998; Hess et al. 2002; Altizer et al. 2003). A major goal for evolutionary ecology and conservation biology is to understand the impacts of population dynamics on adaptive evolution and genetic diversity (Thompson 1998; Spielman et al. 2004). The major histocompatability complex (MHC), in particular, is a gene region that is vitally important for immune defense in vertebrates (Klein 1986). This region contains the most diverse set of coding genes in vertebrates, with most species examined to date showing high levels of allelic diversity and heterozygosity (Edwards and Hedrick 1998; Knapp 2005). Although many populations with low effective population sizes have very low MHC variation (reviewed in Radwan et al. 2010), among some species with small population sizes or low genetic diversity across neutral markers, MHC genes have been shown to exhibit surprisingly high levels of variation (Hedrick et al. 2000; Aguilar et al. 2004; Hedrick and Hurt 2012). How this variation may persist in the face of population declines or fluctuations is an interesting question, with evidence suggesting that a combination of pathogen-mediated selection and mate choice for specific or dissimilar alleles maintains high diversity at the MHC (Apanius et al. 1997; Knapp 2005; Milinski 2006; Spurgin and Richardson 2010).

Glycoproteins encoded by MHC genes bind to foreign antigens and present them to T cells, initiating the immune response (Klein 1986). There are two major groups of MHC genes: Class I recognizes and presents peptides from intracellular pathogens and Class II binds and displays peptides from extracellular pathogens (Hughes and Yeager 1998). In particular, the Class II DRB locus has been the subject of many nonmodel animal studies as it harbors extensive allelic diversity (Bernatchez and Landry 2003). Baseline genetic variation at the DRB locus is thought to be generated primarily by gene duplication via a birth and death process (Nei et al. 1997) or by recombination of alleles (Parham and Ohta 1996). Variation at the DRB locus has been associated with parasite resistance in a variety of animals, including fish, ungulates, rodents, primates, and carnivores (Paterson et al. 1998; Wegner et al. 2003; Schad et al. 2005; Kloch et al. 2010; Srithayakumar et al. 2011). Exon 2 of the DRB in particular encodes the functionally important antigen-binding sites (ABS) recognized by macrophages and antibodies. Thus, proteins encoded by exon 2 bind to unique peptides derived from pathogens, and the genes encoding them can be subjected to intense positive selection, whereby novel types that confer resistance to a specific pathogen can sweep across a population, as evidenced by a greater ratio of amino acid (AA)-changing nonsynonymous substitutions to synonymous substitutions at these codons (Hughes and Yeager 1998). In addition to strong molecular signatures of positive selection at the ABS, the persistence of alleles across speciation events (transspecies polymorphisms) provides further evidence that historic balancing selection has shaped the evolution of the DRB locus (Garrigan and Hedrick 2003). Specifically, alleles conferring fitness benefits are retained in populations even as the new populations adapt to novel conditions and become reproductively isolated from each other.

Low genetic diversity at the MHC has been suggested to cause increased susceptibility to infection for some species (e.g., Tasmanian devils [Siddle et al. 2007]; amphibians [Savage and Zamudio 2011]; cheetah [O'Brien and Evermann 1988]), which could present a serious problem for endangered species with small fragmented populations where DRB diversity has been diminished (Marsden et al. 2009; reviewed in Radwan et al. 2010). However, even in the face of prolonged population bottlenecks, some populations have retained surprisingly high MHC diversity, as reported for San Nicolas Island foxes (*Urocyon littoralis*; Aguilar et al. 2004), water voles (*Arvicola amphibius*, previously *Arvicola terrestris*; Oliver and Piertney 2012), and wild African buffalo (*Syncerus caffer caffer*; Wenink et al. 1998). Species that undergo large fluctuations in population size or regular population cycles are particularly interesting to investigate in terms of MHC diversity, as their effective population sizes are dominated by low-density periods; thus, these species may harbor relatively less allelic diversity over time as rare alleles are lost through genetic drift (Hartl and Clark 1997). On the other hand, high MHC diversity could be readily maintained in fluctuating populations if population size rebounds quickly following declines, if balancing selection is strong enough, and if immigration of individuals from neighboring sites introduces novel alleles.

Here, we examined the pattern of MHC diversity in montane voles (*Microtus montanus*), which inhabit alpine grassy meadows of North America ranging from Colorado to Utah (Sera and Early 2003). Voles from the Arvicolinae subfamily (to which montane voles belong) represent an ideal system in which to investigate MHC diversity, as they tend to undergo dramatic population cycles every 3–7 years (Krebs 1996; Stenseth 1999). Montane voles in particular undergo high amplitude and frequent population cycles, peaking in abundance every 3–4 years (Pinter 1986; R. Smith, unpubl. data). They also have a diversity of parasites (Winternitz et al. 2012) and a promiscuous mating system, thus potentially enabling selection to maintain high MHC diversity through mate choice and parasite interactions. Previous studies of allelic diversity at the DRB in other vole species have shown a mix of results (Oliver and Piertney 2006; Axtner and Sommer 2007). Both the MHC Class II DQA and DRB loci appear to have been duplicated within Arvicolinae, but the timing of this is currently unresolved (Bryja et al. 2006; Axtner and Sommer 2007). It is likely that the combination of population admixture during high population density phases, short generation times, and duplicated genes for many cyclic rodents can maintain MHC polymorphisms despite low effective population sizes and population fluctuations. In addition, selection from numerous parasites (Timm 1985) and MHC-based mating preferences (Radwan et al. 2008) could oppose the effects of genetic drift on MHC diversity in cyclic rodents.

In this study, we first characterized variation at the MHC Class II DRB locus for *M. montanus* to test for evidence of historic balancing selection acting on the DRB locus. Importantly, this represents the first exploration of MHC Class II diversity for a New World arvicoline species. We also undertook a comparative analysis to examine evolution of the DRB locus across rodent species, with the goal of investigating the influence of population dynamics (i.e., stable, cyclic, or bottlenecked populations) and gene duplication on patterns of allelic diversity and signals of selection. We predicted that rodent species that experience population fluctuations or bottlenecks would harbor lower total allelic and nucleotide diversity at the MHC and would show weaker signals of balancing selection on the DRB locus if they are exposed to high degrees of genetic drift. We also predicted that gene duplication at the MHC would correlate with greater allelic divergence and stronger evidence for purifying selection, as alleles from duplicated loci can become specialized to produce products with unique and specific functions (Nei and Rooney 2005). Our ultimate goal was to better understand how species that routinely experience high degrees of genetic drift can maintain immunogenetic diversity over evolutionary timescales.

## Materials and Methods

### Study location and field sampling

Voles were trapped for two consecutive years (2008–2009) at four replicate sites less than 5 km from the Rocky Mountain Biological Laboratory, located in the Upper East River Valley, Colorado (39°N, 107°W). The four trapping sites were comprised of grassy meadows and separated by a minimum of 0.5 km at approximately 2900 m elevation. A total of 284 voles were captured using Longworth live traps for four or five consecutive days per site every 2 weeks throughout the breeding season (June 15–August 15). Animals were uniquely identified with numbered eartags (National Brand Tag Company, Newport, KY, USA), and sex and age were recorded. Full trapping methods are described in Winternitz et al. (2012). To obtain tissue samples for genetic analysis, a 2-mm tail tip was collected from nonjuveniles and stored in 95% ethanol at 5°C after briefly anesthetizing the animals with isoflurane gas.

### Tagged primer design, amplification, and 454 sequencing

Our genetic investigation focused on the MHC class II *DRB* gene exon 2 because it has previously been shown to contain most of the functionally important ABS and is, therefore, the most likely candidate for detecting balancing selection acting on MHC class II genes (Hughes and Yeager 1998). In other words, because these sites determine the range of pathogen proteins that can be recognized and displayed to T cells, different pathogens should favor the maintenance of different alleles that control their recognition by the host's immune response. We isolated and amplified the *DRB* gene for all 284 individuals using polymerase chain reaction (PCR) and then direct Next Generation (454) sequencing. Samples from 20 individuals (∼10%) were cloned and classically Sanger sequenced to establish preliminary allelic data to inform our 454 sequencing protocol. Procedures for obtaining MHC sequence data can be found in the Data S1.

### MHC genotyping and allele validation

Preliminary analysis based on cloning and Sanger sequencing results revealed 1–4 distinct alleles per individual, indicating that the DRB locus in *M. montanus* has undergone at least one duplication event. On the basis of these findings, we calculated the minimum coverage necessary to obtain at least three copies of each allele at 0.999 probability using the method of Galan et al. (2010). The analysis indicated that coverage of 46 reads per individual was sufficient for accurately genotyping individuals (amplicons) with a duplicated gene in a diploid species (Galan et al. 2010). Procedures for artifact filtering and data validation can be found in Data S1).

### Statistical analysis

#### Sequence polymorphism and testing for recombination

Sequences were aligned using Clustal W in MEGA 5.05 (Tamura et al. 2011). MEGA 5.05 was employed to calculate allele mean genetic distance (*d*) based on nucleotide divergence according to the Kimura (1980) two-parameter distance. We constructed phylogenetic trees using the neighbor-joining (NJ) approach and within a Bayesian framework to assess and visualize the genetic similarity between *M. montanus* sequences (see Data S1 for full methods).

Sequence polymorphism was analyzed using DnaSP v5 (Librado and Rozas 2009). Tests for recombination events within alleles were conducted using the program GENECONV (Sawyer 1989) and MaxChi2 (Maynard Smith 1992), which search for unusual substitution patterns, and RDP (Martin and Rybicki 2000) which uses a phylogenetic tree to detect anomalous regions of the alignment. These programs are implemented in the RDP3 program (Martin et al. 2010). These methods performed well in an assessment of 14 recombination detection methods (Posada 2002). The default parameters were used for GENCONV and RDP, and the variable window size with a fraction of 0.10 variable sites per window was selected for MaxChi2 to reduce the risk of false negatives (see RDP3 manual). We also used GARD (Pond et al. 2006), which uses a genetic algorithm to search a multiple-sequence alignment for putative recombination break points, on the web-based server datamonkey (http://www.datamonkey.org).

#### Molecular tests of balancing selection

We tested whether positive selection had been historically operating on the montane vole DRB exon 2 using two approaches, first comparing relative rates of nonsynonymous (dN) and synonymous (dS) substitutions across the gene (Hughes and Nei 1988), and second comparing likelihoods of codon-based models that assume positive selection or neutrality (Nielsen and Yang 1998). First, MEGA 5.05 was used to calculate the relative rates of dN and dS according to the method of Nei and Gojobori (1986) with Jukes and Cantor (1969) correction for multiple hits for all alleles. The substitution rates were calculated separately for nonantigen-binding sites (non-ABS) and 15 ABS based on the human *DRB* gene defined by Brown et al. (1993) and repeated using 12 sites according to the review by Bondinas et al. (2007) (results were qualitatively similar and are not shown). A codon-based two-tailed Z-test of selection was performed on ABS, non-ABS, and all codons to test the null hypothesis of neutrality (dN = dS). As we expected positive selection at ABS, one-tailed *Z*-tests were performed. We also performed directed tests to determine whether negative selection was evident at non-ABS (dN < dS).

We next determined which codon sites were under diversifying positive or negative selection by comparing the congruence of three codon-based models of sequence evolution using the server datamonkey. The three different codon-based maximum likelihood methods, SLAC (single likelihood ancestor counting), FEL (fixed effects likelihood), and the less conservative REL (random effects likelihood), can be used to estimate the dN/dS ratio at every codon in the alignment. These methods of detecting AA sites under selection have been shown to perform as well or better than the M8 model in PAML (Kosakovsky Pond and Frost 2005), and we considered a codon to be evolving under selection when it was identified as such by at least two methods (Kosakovsky Pond and Frost 2005). We also employed MEME (mixed effects model evolution) which combines fixed effects at the level of a site with random effects at the level of branches, in effect allowing some branches to be under positive selection while others are under negative selection. This method is most appropriate to detect episodic selection affecting individual codon sites (Kosakovsky Pond et al. 2011), which may be expected to occur with the introduction of novel parasite species or genotypes.

#### Phylogenetic tests of balancing selection across rodent species

To further examine whether historic balancing selection has been operating on the *M. montanus* DRB locus, we constructed phylogenetic trees to identify the position of *M. montanus* DRB alleles relative to those of other rodent species and to test for transspecies persistence of particular DRB alleles. We used NCBI BLAST searches in Geneious v5.5 to identify and extract DRB exon 2 sequences in 15 rodent species available and 2 nonrodent outgroups (tree shrew, *Tupaia belangeri* and white-tailed deer, *Odocoileus virginianus*; see next section and Table S2 for the complete dataset). We constructed a NJ phylogenetic tree based on nucleotide divergence according to the Kimura two-parameter distance using bootstrap analysis with 5000 replicates in MEGA 5.05. Three alleles were selected randomly from each rodent species. The NJ tree was based on the shared sequence section (171 bp) of all alleles and rooted with the tree shrew (Genbank ref# GU825729) and white-tailed deer (Genbank ref# AF08-2161).

#### Comparing patterns of selection and MHC diversity across rodents

Signatures of selection may be weaker in species with populations subject to high degrees of genetic drift, such as species that have undergone population bottlenecks (Miller and Lambert 2004; Ejsmond and Radwan 2011), or in cyclic species with frequent prolonged low-density periods (Oliver et al. 2009). Selection may also differ among species due to gene copy number, whereby duplicated genes may be specialized for specific biological functions, and thus experience stronger negative selection than single loci (Jarvi et al. 2004; Axtner and Sommer 2007; Burri et al. 2010). To test for patterns of selection across rodent species with varying population dynamics and gene copy numbers, we collected sequence information on montane voles and the 15 additional rodent species with data available for the DRB locus using Geneious and GenBank. Overall, MHC data comprised 27 studies representing 601 alleles from 5565 individual animals, 31 populations, and 16 species. We aligned alleles using Clustal W in MEGA 5.05 and removed all duplicate alleles, pseudogenes, and alleles with insertions or deletions, as these may be nonfunctional. We then trimmed alleles to 171 bp (codons 22–78) for comparable results across species, and assigned ABS at 15 codon sites based on Brown et al. (1993). [We repeated analyses for 12 sites according to Bondinas et al. (2007), but because the results were qualitatively similar, we only present results from ABS assignment based on Brown et al. (1993)].

For all 16 rodent species, we compiled four dependent variables to quantify MHC diversity and indicators of selection: dN/dS at ABS (indicator of balancing selection), dS–dN at non-ABS sites (indicator of purifying selection against structural changes surrounding ABS), average nucleotide divergence (*π*), and number of alleles (indicators of genetic diversity). The variable dS–dN was used to focus on the extent of excess synonymous substitutions, as opposed to the ratio dN/dS which places greater emphasis on nonsynonymous substitutions. Additional details on these four variables and their predictions are provided in Table 1.

**Table 1:** Dependent variables used in the comparative analysis representing MHC diversity and indicators of selection

Dependent variable	Indicates	Interpretation	Prediction
dN/dS at ABS	Historic balancing selection	High dN/dS indicates positive selection at sites that recognize pathogens	Reduced in species with non-functional duplicated loci; reduced in species with lower effective population sizeand stronger genetic drift
dS-dN at non-ABS	Purifying selection	High dS–dN indicates greater rates of silent substitutions that preserve amino acid configurations at sites surrounding ABS	Higher in species with duplicated loci to preserve allelic divergence; reduced in species with lower effective population size and stronger genetic drift
Average nucleotide divergence (π)	Genetic diversity	Extent of variation between sequences	Higher in species with duplicated loci; higher in species with larger effective population size
Number of alleles	Genetic diversity	Number of unique sequences	Higher in species with duplicated loci; higher in species with larger effective population size

To examine the influence of species-specific population dynamics and gene copy number on the strength of selection and MHC diversity, we assigned each species to a categorical measure of population dynamics using three levels: stable, multiannual cycles, or bottlenecked (For full methods, see Data S1). Genetic variables, including the estimated number of DRB loci per species (based on maximum number of alleles observed per individual), were compiled from the studies that provided sequence information. These were also found with systematic literature searches on Web of Science using the search terms “species binomial name and pseudonyms” and “MHC” and “class II.” We then restricted our search to studies focusing on exon 2 of the DRB gene.

For each species in the analysis, we compiled data on several additional variables that could influence measures of MHC diversity and evolution. First, to control for the effect of sampling effort on estimated genetic diversity, we recorded the number of individuals sampled per study for each species. Second, body mass is known to scale with many life-history traits, including population size, reproductive rate, and evolutionary rate (Martin and Palumbi 1993), and thus was included as a covariate. Body mass (g) data were extracted from a previously published database of mammalian traits (PanTHERIA; Jones et al. 2009). Third, effective population size (Ne) can impact genetic diversity by affecting the realized mutation rate, strength of selection, and the amount of genetic drift experienced by a population (Hartl and Clark 1997); further, Ne has been shown to correlate positively with measures of genetic diversity (Frankham et al. 2002). As we could find Ne estimates for only six species in the literature (Sommer et al. 2002; Zheng et al. 2003; Galbreath and Cook 2004; Milishnikov 2004; Busch et al. 2007; J. Winternitz, unpubl. data), we instead used census population size as a correlate of effective population size (Møller et al. 2008; Garamszegi and Nunn 2011), with the caveat that the ratio of Ne/N is approximately 0.1 (Frankham 1995). Population size for each species was estimated by multiplying average population density (individuals/km^2^) from the PanTHERIA database by the species geographic range size (km^2^) extracted from spatial data provided by the 2010 IUCN Red List (http://www.iucnredlist.org/technical-documents/spatial-data#mammals), following a similar approach taken by previous comparative analyses (Nunn et al. 2003, 2005). Studies supplying the genetic data used a mixure of universal and specifically designed primers for assessment of MHC variability, but this had no effect on the number of alleles or DRB loci recovered in a generalized linear model controlling for sampling effort (log number of alleles: *N* = 21, *χ*^2^ = 0.117, degrees of freedom [df] = 1, *P* = 0.732; number of DRB loci: *N* = 21, *χ*^2^ = 0.273, df = 1, *P* = 0.602). Similarly, four of the 27 studies used 454 pyrosequencing which may increase the number of alleles recovered compared to more traditional methods (e.g., single-stranded conformation polymorphism [SSCP], cloning, and direct sequencing). However, there was no significant effect on the log number of alleles controlling for sampling effort (*N* = 30, *χ*^2^ = 1.938, df = 1, *P* = 0.164). Additionally, one species (*Myodes glareolus*) was investigated using SSCP, cloning, and 454 and all methods detected four loci and similar allelic diversity using traditional or next generation approaches relative to sampling effort (*N* = 4, *χ*^2^ = 0.002, df = 1, *P* = 0.963). Our full comparative data set can be found in Table S1. Variables were log-transformed or square root arc-sin transformed (dS rate data; Sokal and Rohlf 1995) when necessary to meet normality assumptions.

We tested for effects of population dynamics and gene duplication on signatures of selection and MHC diversity. Model selection was performed using phylogenetic least squares (PGLS) regression analyses to control for effects of phylogeny. Our initial full models to explain dN/dS, dS–dN, log number of alleles, and *π* included the following species trait predictor variables: population dynamics, number of DRB loci, log body mass (g), log population size, and log sample size. The PGLS regression was conducted using the *caper* package in R (Orme 2011) using Pagel's *λ* statistic to account for phylogenetic nonindependence in the predictor and response variables. Following the recommendations of Bolker et al. (2009), initial models were simplified by removing main effects that appeared to have the least impact on the response. Models with strong support (≥10% Akaike information criteria corrected for smaller sample sizes [AICc] weight) were retained in the confidence set shown in Table S3 (Royall 1997). Model-averaged estimates and unconditional standard errors were calculated using the zero-averaging method (Burnham and Anderson 2002). Rodent phylogeny was constructed using information from the mammalian supertree (Bininda-Emonds et al. 2007) and polytomies were made binary for PGLS using the *multi2di* function in the *Ape* R package (Paradis et al. 2004). We tested for phylogenetic signal on each predictor variable as well as on dS and dN at ABS and non-ABS using two methods: Blomberg's *K* and Pagel's *λ*. Blomberg's *K* (Blomberg et al. 2003) was computed using the *picante* package (Kembel et al. 2010) in R; Blomberg's *K* describes phylogenetic signal of continuous traits, where *K* = 1 indicates a trait is evolving under Brownian motion (stochastic evolution) and *K* < 1 indicates a trait has less phylogenetic signal than expected. Pagel's (1992) *λ* tests for phylogenetic signal through a variance–covariance structuring of the trait data with the species tree, and returns a value of *λ* that describes the phylogenetic signal of the data. When *λ* = 0, the tree is star shaped and all trait values are independent. When *λ* = 1, the original tree best explains the phylogenetic structure of the data (Freckleton et al. 2002).

## Results

### Allele validation

A total of 34,405 total reads were processed with a cloned MHC allele serving as a marker identified in 30,697 initial reads (90%). Of those reads, 21,072 (69%) contained barcodes that were assigned to 261 out of an initial 284 samples corresponding to an average of 80.7 (SD = 88.0) reads per individual. Reads were not assigned to 23 individuals due to insufficient DNA at earlier processing stages. Based on the criteria of a minimum of 54 reads for reliable genotyping (see Data S1), 127 individuals were retained and final genotypes were based on 17,874 reads (58% of the initial number), with the mean coverage at this stage of 140.7 reads (SD = 93.3, range: 54–526). Originally, we identified 82 putative alleles, but 61 were removed from the analysis because they were classified as artifacts or deemed potentially not functional (following criteria described in Materials and Methods and Data S1). We detected a total of 21 DRB alleles among the 127 individual montane voles with sufficient read coverage. Sequences of all alleles were deposited in the Dryad repository (doi:10.5061/dryad.h04hr) following the nomenclature of Klein et al. (1990) and labeled Mimo-DRB*01 through Mimo-DRB*21. BLAST search confirmed the homology to other rodent *DRB* sequences for all alleles (99% to 91% similarity).

The mean number of alleles per individual was 2.19 (SD = 0.67), and the maximum number of alleles in a single individual was four, suggesting that the MHC DRB locus in montane voles is duplicated. A total of 89 individuals had 2 alleles, 34 had 3 alleles, and 4 had 4 alleles. We found no evidence for genetic recombination or gene conversion events between aligned sequences and/or ancestral relicts of such events using GENECONV, MaxChi2, RPD, or GARD. Results comparing alleles detected with cloning and 454 sequencing (14/20 individuals had sufficient clones and read coverage for genotyping) indicated 74% congruence between genotypes determined by the two methods. Not all alleles detected using 454 sequencing were detected with cloning, as only 6–10 colonies per individual were selected, resulting in a 47–76% probability of selecting every allele at least twice based on binomial probability. However, all alleles detected in at least two individual PCRs with cloning were also detected in those same individuals using 454 sequencing.

### Allelic diversity and clustering

There were 67 variable sites of 171 total sites, with a total of 82 mutations. The average nucleotide divergence across all sequences (*π*, average number of substitutions per site between sequences) was 0.13,299 (±0.016 SD). The average number of nucleotide differences between sequences was 22.72 with mean pairwise nucleotide distances of 0.185. The average number of AA differences between sequences was 6.5 and mean pairwise AA distance computed for all sites ranged from 0 to 0.726, with the average 0.294.

**Figure 1:**
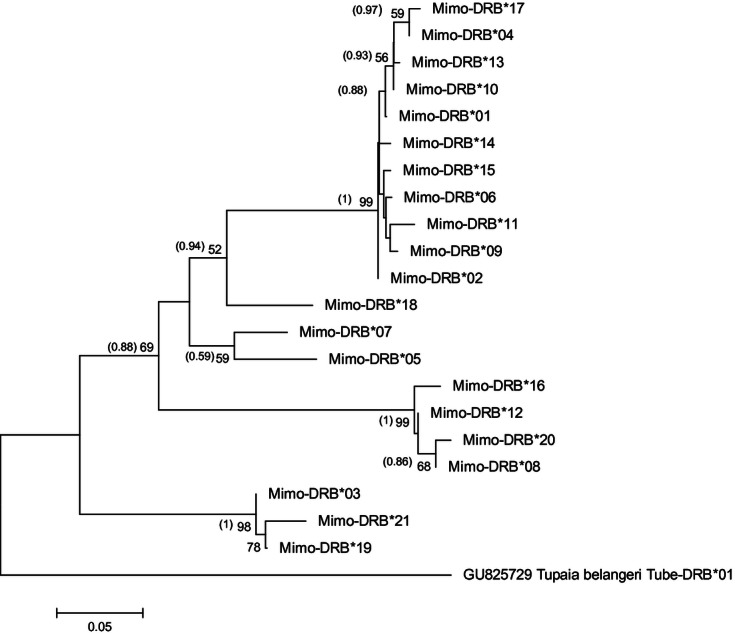
Neighbor-joining (NJ) phylogeny and Bayesian estimated posterior probabilities for all 21 *M. montanus* (Mimo-DRB) alleles using a tree shrew (Tupaia belangeri) sequence as an outgroup (GenBank accession no. GU825729). We constructed the NJ phylogeny with the Kimura-two parameter nucleotide distance considering all sites. Node numbers indicate bootstrap support ≥50 (5000 replicates) and numbers in parentheses indicate Bayesian posterior probabilities above 80%. Scale bar indicates the genetic distance. Alleles are partitioned hierarchically into two groups representing two putative loci.

Alleles did not cluster into groups indicating putative alleles based on nucleotide divergence using Kimura two-parameter distances and using a Bayesian approach (Fig. 1). Allele number per individual (2–4 alleles) indicated a duplicated locus; however, we were unable to assign individual alleles to putative loci. A PCR artifact such as allelic dropout (where primers fail to amplify specific alleles) may be present or montane voles may vary in their gene copy number. Although intraspecies gene copy number variation has been found in other species (carnivores, Bowen et al. 2004; primates, Bontrop 2006; rodents, Kloch et al. 2010), it is also possible that the generic primers used in this study were biased toward specific loci and may have missed a fraction of alleles. Species-specific primers would be necessary to test whether we have captured the complete variation at the DRB in *M. montanus*.

### Molecular evidence of balancing selection

**Figure 2:**
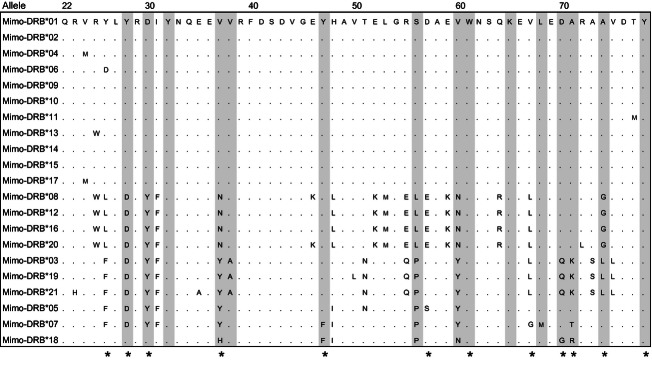
Alignment of 21 identified amino acid sequences of the partial DRB exon 2 of the montane vole, *Microtus montanus* (Mimo-DRB). A dot indicates congruence with the amino acid sequence of Mimo-DRB*01. Gray shading denotes antigen binding sites (ABS) according to Brown et al. (1993) and astericks at the bottom of the table denote ABS according to Bondinas et al. (2007).

Neither nucleotide nor AA pairwise differences was higher at ABS (10.5 ± 1.7 and 4.8 ± 0.9) than at non-ABS (12.2 ± 1.8 and 6.5 ± 1.3), indicating that measures of selection were not necessarily stronger for sites involved in antigen recognition. The ratio of dN/dS at the ABS was less than 1, indicating no support for balancing selection (Table 2). Considering only non-ABS codons (*N* = 42) and all codon sites (*N* = 57), the ratio of dN/dS was significantly less than 1, and purifying selection was indicated indirected *Z*-tests (*w* < 1) (non-ABS, *P* = 0.03; all sites, *P* = 0.02; Table 2). The codon-based maximum likelihood methods also detected signatures of purifying selection at a greater frequency than positive selection. Only one codon (codon 26) was identified as positively selected (posterior probability >95%) based on congruence with two or more methods (FEL and MEME, Table 3). In contrast, based on results from MEME, SLAC, FEL, and REL, three codons were identified as undergoing episodic diversifying selection (codons 26, 30, and 57) and eight were identified as undergoing purifying selection at non-ABS sites (codons 29, 36, 42, 43, 49, 54, 58, and 62; Table 4; Fig. 2).

**Table 2:** Nonsynonymous (dN) and synonymous (dS) substitutions (± SE from 5000 bootstraps) as well as their ratio in ABS and non-ABS based on sites defined by Brown et al. (1993)

Site	DN	dS	dN/dS	*P w* ≠ 1	*P w* < 1
ABS *N* = 15	0.311 ± 0.118	0.407 ± 0.167	0.765	0.585	0.291
Non-ABS *N* = 42	0.084 ± 0.020	0.237 ± 0.064	0.353	0.065	**0.030**
All sites *N* = 57	0.131 ± 0.026	0.276 ± 0.060	0.474	**0.039**	**0.020**

ABS, antigen-binding sites; *N*, number of codons in each category; *P*, probability (*α* < 0.05) that dN and dS are different (*w* ≠ 1) and that dN<dS (*w* < 1) using a *Z*-test. Significant *P-*values are in bold.

**Table 3:** Codons under selection based on significance determined from at least two of three different codon-based maximum likelihood methods

Type of selection	Codon	Models (S, F, R, M)
Episodic diversifying	26	M
	30^1^	M
	57	M
Positive	26	F, M
Negative	29	F, R
	36	F, R
	42	S, F, R
	43	S, F, R
	49	S, F, R
	54	F, R
	58	S, F, R
	62	F, R

S, single likelihood ancestor counting; F, fixed effects likelihood; and R, the less conservative random effects likelihood. M, episodic selection is based on significant results from mixed effects model evolution. Significance was determined with *α* < 0.05 or Bayes Factor >50, respective to the likelihood model.

1 Denotes ABS site based on Brown et al. (1993).

**Table 4:** Summary of sequence and allelic diversity for all 21 *Microtus montanus* DRB alleles

Mean nuc. distance (SE)	Mean AA distance (SE)	Total allele count (%)	No. animals with alleles
0.19 (0.03)	0.29 (0.07)	281	127

Nuc. refers to nucleotide, AA refers to amino acid.

### Phylogenetic evidence of balancing selection

**Figure 3:**
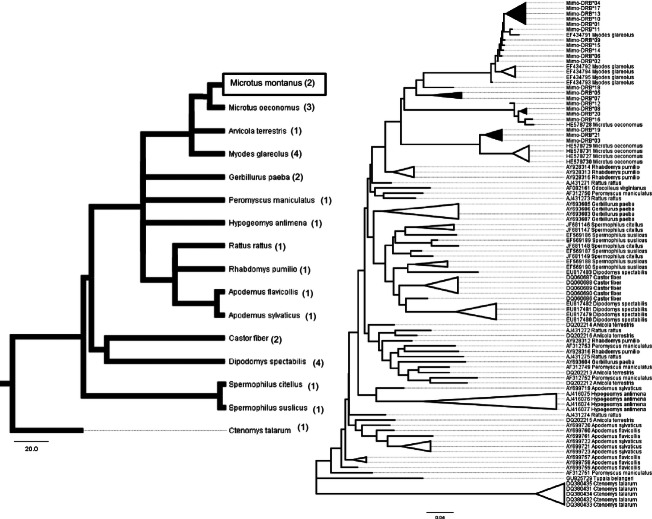
Trans-species polymorphism of the MHC class II DRB gene in 16 rodent species. The DRB tree on the right is a neighbor-joining phylogeny of all 21 *M. montanus* DRB alleles (in black) compared with 45 DRB sequences from 15 different rodent species, using a tree shrew (Tupaia belangeri) and white-tailed deer sequence (Odocoiles virginianus) as outgroups. GenBank ascension numbers are given after each species name. *M. montanus* alleles (Mimo-DRB colored black) cluster among its nearest relatives, the bank vole (Myodes glareolus) and root vole (Microtus oeconomus). The scale bar indicates genetic distance in units of nucleotide substitutions per site. The species tree on the left was derived from the mammal supertree (Bininda-Emonds et al. 2007) and depicts the evolutionary divergence between species, with the scale bar indicating Myr. Numbers at the tips indicate the putative number of DRB loci for each species. *M. montanus* is boxed.

To compare *M. montanus* DRB alleles to those of other rodents and to examine evolution in MHC allelic lineages, we constructed a phylogenetic tree of 16 rodent species using DRB sequence data (Fig. 3). *M. montanus* alleles clustered among alleles from its nearest relatives, the bank vole (*M. glareolus*) and root vole (*Microtus oeconomus*).

Across all rodents, there was evidence of transspecies evolution at the DRB, indicating that ancestral alleles with important functions are retained in descendant species (Takahata 1990), although similar patterns could arise from convergent evolution. Alleles from some distantly related species, including tuco tucos (*Ctenomys talarum*), the Malagasy giant rat (*Hypogeomys antimena*), the banner-tailed kangaroo rat (*Dipodomys spectabilis*), and the Eurasian beaver (*Castor fiber*) formed monophyletic clades, while other alleles were shared among close relatives (e.g., *Spermophilus citellus* and *S. suslicus*, *Apodemus flavicollis* and *A. sylvaticus*) (Fig. 3). Some rodent DRB alleles were widely dispersed across multiple species (*Gerbillurus paeba*, *Rhabdomys pumilio*, *Rattus rattus*, *Peromyscus maniculatus*, *A. flavicollis*, *A. sylvaticus*, and *A. terrestris*), indicating that balancing selection may have preserved alleles across speciation events.

### Predictors of selection and MHC diversity across rodents

**Figure 4:**
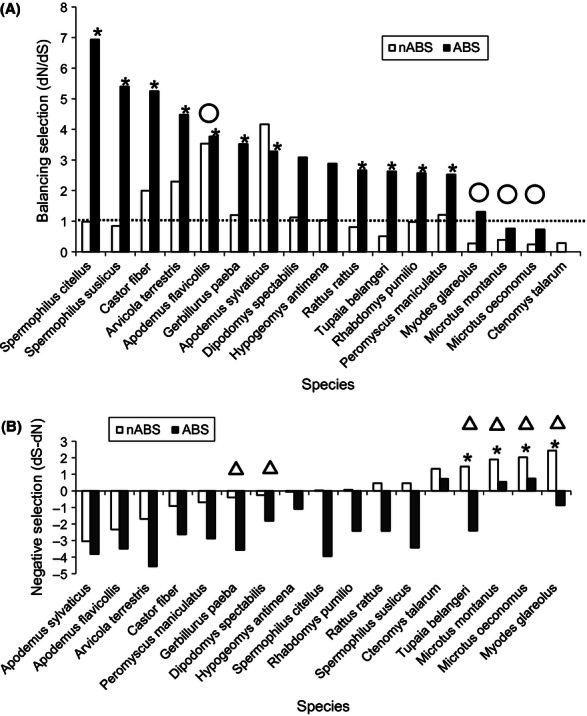
Evidence of balancing selection at the antigen binding sites (ABS) (Brown et al. 1993) and negative selection at non-ABS across 16 rodent species and Tupaia. (A) The ratio of nonsynonymous to synonymous substitutions per site (dN/dS) was calculated at non-ABS (open bar) and ABS (filled bar) sites separately for each species. Significant departures from neutrality (dN/dS) = 1 at the ABS were determined with codon based *Z*-tests using the Nei-Gojobori method with Jukes-Cantor correction in Mega 5.05. Variance was computed using the bootstrap method (1000 replicates). Asterisks denote significance at *P* < 0.05 (See Table S2 for *P*-values). Dotted line indicates neutrality (dN/dS) = 1 and open circles indicates species with cyclic population dynamics. (B) Evidence of purifying selection at non-ABS across 16 rodent species and Tupaia. We performed codon-based *Z*-tests as above. The test statistic (dS-dN) was calculated for non-ABS (open bar) and ABS (filled bar) sites separately for each species. Astericks denote significance at *P* < 0.05 and open triangles indicate species with duplicated DRB genes (See Table S2 for *P*-values).

Rates of nonsynonymous (dN) and synonymous substitutions (dS) across 601 DRB exon 2 sequences from 16 rodent species were associated with categorical measures of population dynamics and the presence of gene duplication. Most rodent species included in this study showed a significantly elevated rate of dN to dS (dN/dS>1) at the functionally important ABS (Fig. 4A), consistent with other work showing that historic positive selection acts to increase diversity at these sites (Bernatchez and Landry 2003; Sommer 2005). However, four of five species with “cyclic” population dynamics (and potentially lower effective population sizes) did not show evidence of positive selection on MHC based on nondirected *Z*-tests (*P* > 0.05). Testing for negative selection with directed *Z*-tests at non-ABS sites across species revealed significantly higher dS than dN in four of six species with duplicated loci, consistent with expectations that purifying selection retaining structural formation surrounding ABS may be the norm for duplicated MHC loci (Fig. 4B, Table S2).

To identify predictors of signals of selection and diversity at the MHC while controlling for shared evolutionary history and other species traits, we performed PGLS linear models. The confidence set of models for all four predictor variables (dN/dS at ABS, dS–dN at non-ABS, average nucleotide divergence (*π*), and number of alleles) had significant intercepts and full model *P-*values less than 0.05 (Table S3), indicating that these models were valid. The confidence sets of models showed no evidence for phylogenetic signal explaining the response variables (Table S3), indicating that evolutionary history did not strongly influence these analyses. This was generally the case for the predictor variables as well, as most species traits did not show phylogenetic signal using Blomberg's *K* or Pagel's *λ* (Table S4). However, the presence of gene duplication had a significant *λ* value (*λ* = 0.81, *P* = 0.00), as did the rate of dN at ABS (*λ* = 1, *P* = 0.00), indicating that more closely related species were more likely to share duplication status and dN rates than expected by chance.

**Figure 5:**
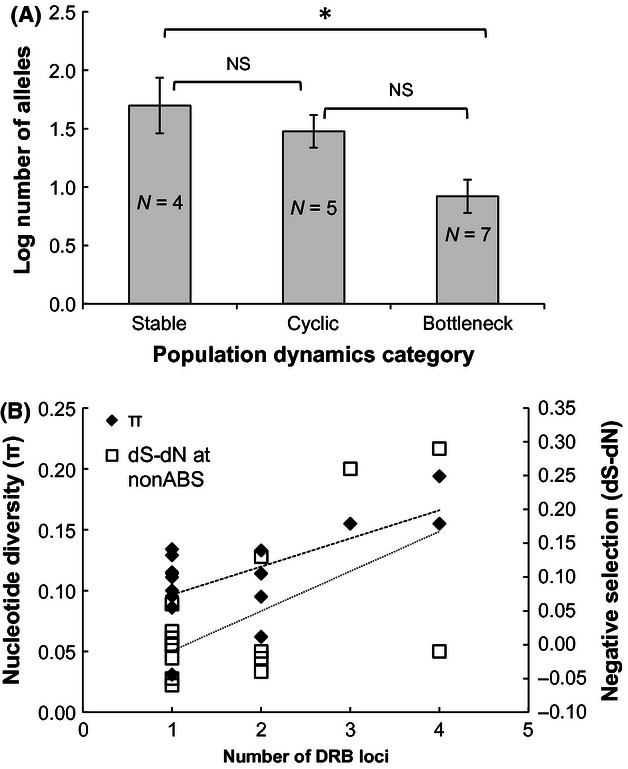
Interspecific trait effects on signals of selection and nucleotide diversity at the DRB in 16 rodent species. (A) Relationship between population dynamics categorized as stable, cyclic, and bottlenecked, and DRB allelic richness. N indicates sample size and error bars indicate 95% confidence intervals. (B) Relationship between the number of DRB loci per species and average nucleotide divergence (π, black diamonds) and strength of purifying selection (dS–dN at non antigen binding sites, open squares). For the secondary *y*-axis, zero indicates equal rates of synonymous and nonsynonymous substitutions, while positive values indicate negative selection for amino-acid changing substitutions. The black dotted line is the linear best fit for π, and the gray dotted line is the linear best fit for “purifying selection.”

Of the four dependent variables we examined in our multivariate models, all had models with statistically significant predictor variables (Table S5). In particular, the log number of alleles was significantly greater for species characterized as stable than for bottlenecked species (Fig. 5A). An analysis of variance on the log number of alleles yielded significant variation among species by population dynamics (*F*_2,15_ = 6.06, *P* = 0.01), and a post hoc Tukey test further showed that stable species had significantly greater number of alleles than bottlenecked, but not cyclic, species at *P* < 0.05 (Fig. 5A). When considering effects of gene duplication on measures of selection and genetic diversity across rodent species, both nucleotide diversity and the strength of negative (purifying) selection increased with the number of DRB loci (our measure of MHC gene duplication; Fig. 5B). The strength of balancing selection (dN/dS at ABS) was negatively related to log population size and positively related to log body mass, potentially indicating that realized substitution rate at ABS is affected by population size and life-history traits. No other predictor variables were significantly associated with measures of genetic diversity or selection at the MHC.

## Discussion

We found moderate levels of genetic diversity at the DRB locus in montane voles (21 alleles detected in 127 individuals; close to the average of the 16 rodent species from 31 populations used in this study based on a log-allele to log-sample size regression: [*y* = 0.207*x* + 0.74; *R*^2^ = 0.093]). Because this species undergoes high-amplitude population fluctuations that could lead to diversity loss through genetic drift, we further tested for evidence of historic balancing selection to explain the maintenance of this high diversity. Our results showed that the DRB in montane voles lacks molecular signals of balancing selection based on dN/dS ratios, but does show evidence of balancing selection based on the transspecies persistence of alleles. In other words, because montane vole alleles also appear across multiple rodent species, this implies these alleles have persisted longer than neutral polymorphisms (Takahata and Nei 1990) due to balancing selection retaining adaptive alleles. However, the transspecies polymorphism detected across 16 rodent species in this study may result from greater similarities between alleles shared between ancestral duplicated loci compared to alleles from paralogous loci within the same species. It is interesting that evidence from the DRB gene supports the conservation of alleles across species, a pattern that diverges from the DQA (a different MHC II gene) which does not show clear evidence for transspecies polymorphism in rodents (Pfau et al. 1999). Whether this difference in allelic preservation is due to biological function or architecture of the specific genes remains to be determined. We also found no evidence of recombination at the DRB in montane voles, in accord with results from six other mammal species (Furlong and Yang 2008), indicating that, in general, recombination has not made significant contributions to DRB variation (but see Richman et al. 2003). However, recombination was found at the DQA locus in three Old World vole species (Bryja et al. 2006), highlighting that different mechanisms could be responsible for generating high diversity in different MHC loci, despite their similar functions and close proximity to each other.

We found strong evidence for purifying selection operating on the DRB locus of *M. montanus* using multiple codon-based likelihood methods. This result runs counter to evidence for balancing selection that appears to be a rule for DRB evolution in other mammal species (Bernatchez and Landry 2003). In montane voles, purifying selection appears to be acting at eight non-ABS sites (codons 29, 36, 42, 43, 49, 54, 58, and 62) as detected by the codon-based likelihood methods used here. Importantly, two non-ABS sites (codons 36 and 54) also showed signals of purifying selection in six other mammal species (Furlong and Yang 2008), raising the question of why purifying selection might operate on the MHC. One answer might be that conserved regions that experience purifying selection are important for the basic structural elements of the ABS, beyond the more variable sites that recognize specific epitopes. Purifying selection might also occur when duplicated loci develop specific roles for resistance to specific pathogens or mate choice and undergo reduced evolutionary rates of selection (Jordan et al. 2004).

Our comparative analysis examined whether other rodent species show weak molecular evidence of balancing selection and strong evidence of purifying selection at the MHC, similar to *M. montanus*. We found some evidence that cyclic species (those that undergo regular population fluctuations, such as montane voles) may experience weaker positive selection than noncyclic species. On the other hand, other studies on rodents showed that high variance in population size does not negatively affect genetic diversity at neutral markers (Berthier et al. 2006). In fact, some previous studies found high neutral genetic diversity in periodically fluctuating rodent populations and concluded that increased gene flow through movement of individuals with novel genotypes during the low abundance phase (when population structure is more fluid) could counter the force of genetic drift (Ims and Andreassen 2005; Aars et al. 2006), pointing to the importance of meta-population dynamics. Indeed, high-amplitude fluctuations in abundance might cause an increase in genetic diversity if dispersal is negative density-dependent and if alleles invade adjacent populations during the low phase in population cycles (Ehrich et al. 2009). We also found evidence that species with larger population sizes and relatively smaller bodied species may experience weaker positive selection. This may due to a smaller realized substitution rate in larger populations where mutations for nonsynonymous substitutions take longer to spread within the population.

Species that undergo severe and/or repeated population bottlenecks could experience different evolutionary forces than those that undergo more regular population cycles. In fact, cyclic populations tend to have relatively large population sizes and are characterized by interconnected meta-populations with frequent opportunities for gene flow (Berthier et al. 2006). In contrast, bottlenecked populations tend to be isolated and without opportunity for rescue effects to replenish lost alleles. In our comparative analysis, we found lower allelic richness in bottlenecked species. Similarly, an earlier meta-analysis focusing on bottleneck effects found that both neutral and nonneutral diversity were lower in bottlenecked species, and further showed that two measures of MHC diversity (heterozygosity and allelic diversity) were reduced 15% more than neutral genetic diversity, possibly due to the combined effects of genetic drift and negative frequency-dependent selection acting to imbalance the frequency of alleles (Sutton et al. 2011).

Our comparative analysis provided strong support for an association between gene duplication and greater measures of purifying selection and allelic diversity at the DRB. This result is most consistent with the hypothesis that gene duplication allows diverse alleles to take on specific roles and purifying selection acts to maintain functional divergence between alleles on different copies of the locus (Hughes and Friedman 2004; Axtner and Sommer 2007). As multiple MHC loci have been reported from different taxonomic groups (fish, Reusch et al. 2004; mammals, Bryja et al. 2006; birds, Zagalska-Neubauer et al. 2010), and these loci could potentially contribute to increasing allelic diversity vital for immune function, it is important to consider the potential causes behind MHC gene copy number variation. Selection against T-cell depletion and autoimmune disease may be a force opposing increasing gene duplications (Nowak et al. 1992; Woelfing et al. 2009; Kloch et al. 2010). At present, it remains an open question as to what species traits, which might include life-history traits, population size, and MHC architecture, are most strongly correlated with MHC diversity and gene duplication.

We have concerns that our use of generic primers may not have captured the full allelic diversity in *M. montanus*, which was shown to have multiple loci that may have experienced purifying selection maintaining functional divergence. This is a general problem for nonmodel species as many have been shown to have both inter- and intraspecific copy number variation (Bowen et al. 2004; Bontrop 2006; Kloch et al. 2010). Use of next generation sequencing technology has improved the characterization of allelic diversity by increasing sampling coverage (Oomen et al. 2013), and has successfully been used to amplify multiple loci among 24 rodent species even using generic primers (Galan et al. 2010). Our study also utilized 454 pyrosequencing and generic primers to amplify multiple DRB loci, yet we acknowledge this is a preliminary characterization for *M. montanus*, and species-specific primers would be necessary to recover the full MHC allelic diversity and reliably genotype individuals for disease associations.

In summary, our study provides empirical support for gene duplication as a mechanism that could maintain genetic diversity at the MHC in montane voles that undergo population fluctuations, and corroborates this result using evidence from multiple rodent species in a phylogenetically controlled analysis. More generally, purifying selection acting on duplicated loci could cause the high divergence between alleles observed among species with duplicated DRB genes. In addition, we found evidence that population demographics influence the strength of historic balancing selection, causing reduced allelic diversity in bottlenecked populations and reduced positive selection in cyclic rodents. These results illustrate that patterns of natural selection in wild populations are shaped by gene duplications and demographic history.

## Data accessibility

DNA sequences (FASTA format) are uploaded as online supporting information and data used for comparative analyses are listed in Tables S1 and S2.

## Supporting Information

**Data S1.** Genetic methods and statistical analyses.

**Table S1**. The comparative dataset of 16 rodent species used in the analysis.

**Table S2**. Results from codon-based *Z-*tests for (i) departures from neutrality (dN/dS ≠ 1) at the antigen binding sites (ABS) based on Brown et al. (1993) and Bondinas et al. (2007); (ii) negative selection (dS–dN > 1) at non-ABS across 16 rodent species and *Tupaia*.

**Table S3**. The subset of highly supported phylogenetic generalized least squares regression (PGLS) models (≥10% AICc weights of the top model) explaining the following dependent variables: log number of alleles, nucleotide diversity (π), dN/dS at ABS, and dN–dS at nonABS.

**Table S4**. Phylogenetic signal in rodent traits measured by Blomberg's K and Pagel's λ.

**Table S5**. Model-averaged estimates of the different parameters in the subset of models with high confidence (≥10% AICc weights of the top model), as well as the unconditional standard error, 95% confidence intervals, and importance.

## References

[b1] Aars J, Dallas JF, Piertney SB, Marshall F, Gow JL, Telfer S (2006). Widespread gene flow and high genetic variability in populations of water voles *Arvicola terrestris* in patchy habitats. Mol. Ecol.

[b2] Aguilar A, Roemer G, Debenham S, Binns M, Garcelon D, Wayne RK (2004). High mhc diversity maintained by balancing selection in an otherwise genetically monomorphic mammal. Proc. Natl Acad. Sci. USA.

[b3] Altizer S, Harvell D, Friedle E (2003). Rapid evolutionary dynamics and disease threats to biodiversity. Trends Ecol. Evol.

[b4] Apanius V, Penn D, Slev PR, Ruff LR, Potts WK (1997). The nature of selection on the major histocompatibility complex. Crit. Rev. Immunol.

[b5] Axtner J, Sommer S (2007). Gene duplication, allelic diversity, selection processes and adaptive value of mhc class ii drb genes of the bank vole, *Clethrionomys glareolus*. Immunogenetics.

[b6] Bernatchez L, Landry C (2003). Mhc studies in nonmodel vertebrates: What have we learned about natural selection in 15 years?. J. Evol. Biol.

[b7] Berthier K, Charbonnel N, Galan M, Chaval Y, Cosson JF (2006). Migration and recovery of the genetic diversity during the increasing density phase in cyclic vole populations. Mol. Ecol.

[b8] Bininda-Emonds ORP, Cardillo M, Jones KE, MacPhee RDE, Beck RMD, Grenyer R (2007). The delayed rise of present-day mammals. Nature.

[b9] Blomberg SP, Ives T, Garland AR (2003). Testing for phylogenetic signal in comparative data: behavioral traits are more labile. Evolution.

[b10] Bolker BM, Brooks ME, Clark CJ, Geange SW, Poulsen JR, Stevens MHH (2009). Generalized linear mixed models: a practical guide for ecology and evolution. Trends Ecol. Evol.

[b11] Bondinas G, Moustakas A, Papadopoulos G (2007). The spectrum of hla-dq and hla-dr alleles, 2006: a listing correlating sequence and structure with function. Immunogenetics.

[b12] Bontrop RE (2006). Comparative genetics of mhc polymorphisms in different primate species: duplications and deletions. Hum. Immunol.

[b13] Bowen L, Aldridge B, Gulland F, Bonn W, DeLong R, Melin S (2004). Class ii multiformity generated by variable mhc drb region configurations in the California sea lion (*Zalophus californianus*. Immunogenetics.

[b14] Brown JH, Jardetzky TS, Gorga JC, Stern LJ, Urban RG, Strominger JL (1993). Three-dimensional structure of the human class ii histocompatibility antigen hla-dr1. Nature.

[b15] Bryja J, Galan M, Charbonnel N, Cosson J (2006). Duplication, balancing selection and trans-species evolution explain the high levels of polymorphism of the dqa mhc class ii gene in voles (arvicolinae). Immunogenetics.

[b16] Burnham KP, Anderson DR (2002). Model selection and multimodel inference: a practical information-theoretic approach.

[b17] Burri R, Salamin N, Studer RA, Roulin A, Fumagalli L (2010). Adaptive divergence of ancient gene duplicates in the avian mhc class ii β. Mol. Biol. Evol.

[b18] Busch JD, Waser PM, DeWoody JA (2007). Recent demographic bottlenecks are not accompanied by a genetic signature in banner-tailed kangaroo rats (*Dipodomys spectabilis*. Mol. Ecol.

[b19] Edwards SV, Hedrick PW (1998). Evolution and ecology of mhc molecules: from genomics to sexual selection. Trends Ecol. Evol.

[b20] Ehrich D, Yoccoz NG, Ims RA (2009). Multi-annual density fluctuations and habitat size enhance genetic variability in two northern voles. Oikos.

[b21] Ejsmond MJ, Radwan J (2011). Mhc diversity in bottlenecked populations: a simulation model. Conserv. Genet.

[b22] Frankham R (1995). Effective population size/adult population size ratios in wildlife: a review. Genet. Res.

[b23] Frankham R, Ballou JD, Briscoe DA (2002). Introduction to conservation genetics.

[b24] Freckleton R, Harvey P, Pagel M (2002). Phylogenetic analysis and comparative data: a test and review of evidence. Am. Nat.

[b25] Furlong R, Yang Z (2008). Diversifying and purifying selection in the peptide binding region of drb in mammals. J. Mol. Evol.

[b26] Galan M, Guivier E, Caraux G, Charbonnel N, Cosson JF (2010). A 454 multiplex sequencing method for rapid and reliable genotyping of highly polymorphic genes in large-scale studies. BMC Genomics.

[b27] Galbreath KE, Cook JA (2004). Genetic consequences of pleistocene glaciations for the tundra vole (*Microtus oeconomus*) in Beringia. Mol. Ecol.

[b28] Garamszegi LZ, Nunn CL (2011). Parasite-mediated evolution of the functional part of the mhc in primates. J. Evol. Biol.

[b29] Garrigan D, Hedrick PW (2003). Perspective: detecting adaptive molecular polymorphism: lessons from the mhc. Evolution.

[b30] Hartl DL, Clark AG (1997). Principles of population genetics.

[b31] Hedrick PW, Hurt CR (2012). Conservation genetics and evolution in an endangered species: research in sonoran topminnows. Evol. Appl.

[b32] Hedrick PW, Parker KM, Gutiérrez-Espeleta GA, Rattink A, Lievers K (2000). Major histocompatibility complex variation in the Arabian oryx. Evolution.

[b33] Hess G, Randolph S, Arneberg P, Chemini C, Furlanello C, Harwood J, Hudson P, Rizzoli A, Grenfell B, Heesterbeek H, Dobson A (2002). Spatial aspects of disease dynamics. The ecology of wildlife diseases.

[b34] Hughes AL, Friedman R (2004). Shedding genomic ballast: extensive parallel loss of ancestral gene families in animals. J. Mol. Evol.

[b35] Hughes AL, Nei M (1988). Pattern of nucleotide substitution at major histocompatibility complex class i loci reveals overdominant selection. Nature.

[b36] Hughes AL, Yeager M (1998). Natural selection at major histocompatibility complex loci of vertebrates. Ann. Rev. Genet.

[b37] Ims RA, Andreassen HP (2005). Density-dependent dispersal and spatial population dynamics. Proc. Biol. Sci.

[b38] Jarvi SI, Tarr CL, McIntosh CE, Atkinson CT, Fleischer RC (2004). Natural selection of the major histocompatibility complex (mhc) in Hawaiian honeycreepers (drepanidinae). Mol. Ecol.

[b39] Jones KE, Bielby J, Cardillo M, Fritz SA, O'Dell J, Orme CDL (2009). Pantheria: a species-level database of life history, ecology, and geography of extant and recently extinct mammals. Ecology.

[b40] Jordan IK, Wolf Y, Koonin E (2004). Duplicated genes evolve slower than singletons despite the initial rate increase. BMC Evol. Biol.

[b41] Jukes TH, Cantor CR, Munro HN (1969). Evolution of protein molecules. Mammalian protein metabolism.

[b42] Kembel SW, Cowan PD, Helmus MR, Cornwell WK, Morlon H, Ackerly DD (2010). Picante: R tools for integrating phylogenies and ecology. Bioinformatics.

[b43] Kimura M (1980). A simple method for estimating evolutionary rates of base substitutions through comparative studies of nucleotide sequences. J. Mol. Evol.

[b44] Klein J (1986). Natural history of the major histocompatibility complex.

[b45] Klein J, Bontrop RE, Dawkins RL, Erlich HA, Gyllensten UB, Heise ER (1990). Nomenclature for the major histocompatibility complexes of different species: a proposal. Immunogenetics.

[b46] Kloch A, Babik W, Bajer A, SiŃSki E, Radwan J (2010). Effects of an mhc-drb genotype and allele number on the load of gut parasites in the bank vole *Myodes glareolus*. Mol. Ecol.

[b47] Knapp LA (2005). The abcs of mhc. Evol. Anthropol.

[b48] Kosakovsky Pond SL, Frost SDW (2005). Not so different after all: a comparison of methods for detecting amino acid sites under selection. Mol. Biol. Evol.

[b49] Kosakovsky Pond SL, Murrell B, Fourment M, Frost SDW, Delport W, Scheffler K (2011). A random effects branch-site model for detecting episodic diversifying selection. Mol. Biol. Evol.

[b50] Krebs CJ (1996). Population cycles revisited. J. Mammal.

[b51] Librado P, Rozas J (2009). Dnasp v5: a software for comprehensive analysis of DNA polymorphism data. Bioinformatics.

[b52] Marsden CD, Mable BK, Woodroffe R, Rasmussen GSA, Cleaveland S, McNutt JW (2009). Highly endangered African wild dogs (*lycaon pictus*) lack variation at the major histocompatibility complex. J. Hered.

[b53] Martin AP, Palumbi SR (1993). Body size, metabolic rate, generation time, and the molecular clock. Proc. Natl Acad. Sci.

[b54] Martin D, Rybicki E (2000). Rdp: detection of recombination amongst aligned sequences. Bioinformatics.

[b55] Martin DP, Lemey P, Lott M, Moulton V, Posada D, Lefeuvre P (2010). Rdp3: a flexible and fast computer program for analyzing recombination. Bioinformatics.

[b56] Maynard Smith J (1992). Analyzing the mosaic structure of genes. J. Mol. Evol.

[b57] Milinski M (2006). The major histocompatibility complex, sexual selection, and mate choice. Ann. Rev. Ecol. Evol. Syst.

[b58] Milishnikov AN (2004). Population-genetic structure of beaver (castor fiber l., 1758) communities and estimation of effective reproductive size of an elementary population. Russ. J. Genet.

[b59] Miller HC, Lambert DM (2004). Genetic drift outweighs balancing selection in shaping post-bottleneck major histocompatibility complex variation in New Zealand robins (petroicidae). Mol. Ecol.

[b60] Møller A, Garamszegi L, Spottiswoode C (2008). Genetic similarity, breeding distribution range and sexual selection. J. Evol. Biol.

[b61] Nei M, Gojobori T (1986). Simple methods for estimating the numbers of synonymous and nonsynonymous nucleotide substitutions. Mol. Biol. Evol.

[b62] Nei M, Rooney AP (2005). Concerted and birth-and-death evolution of multigene families. Ann. Rev. Genet.

[b63] Nei M, Gu X, Sitnikova T (1997). Evolution by the birth-and-death process in multigene families of the vertebrate immune system. Proc. Natl Acad. Sci. USA.

[b64] Nielsen R, Yang Z (1998). Likelihood models for detecting positively selected amino acid sites and applications to the hiv-1 envelope gene. Genetics.

[b164] Nunn CL, Altizer S, Jones KE, Sechrest W (2003). Comparative tests of parasite species richness in primates. Am. Nat.

[b165] Nunn CL, Altizer SM, Sechrest W, Cunningham AA (2005). Latitudinal gradients of parasite species richness in primates. Divers. Distrib.

[b65] Nowak MA, Tarczy-Hornoch K, Austyn JM (1992). The optimal number of major histocompatibility complex molecules in an individual. Proc. Natl Acad. Sci. USA.

[b66] O'Brien SJ, Evermann JF (1988). Interactive influence of infectious disease and genetic diversity in natural populations. Trends Ecol. Evol.

[b67] Oliver M, Piertney S (2006). Isolation and characterization of a mhc class ii drb locus in the European water vole (*Arvicola terrestris*. Immunogenetics.

[b68] Oliver MK, Piertney SB (2012). Selection maintains mhc diversity through a natural population bottleneck. Mol. Biol. Evol.

[b69] Oliver MK, Lambin X, Cornulier T, Piertney SB (2009). Spatio-temporal variation in the strength and mode of selection acting on major histocompatibility complex diversity in water vole (*Arvicola terrestris*) metapopulations. Mol. Ecol.

[b70] Oomen RA, Gillett RM, Kyle CJ (2013). Comparison of 454 pyrosequencing methods for characterizing the major histocompatibility complex of nonmodel species and the advantages of ultra deep coverage. Mol. Ecol. Resour.

[b71] Orme D (2011). The caper package: comparative analysis of phylogenetics and evolution in R.

[b72] Pagel MD (1992). A method for the analysis of comparative data. J. Theor. Biol.

[b73] Paradis E, Claude J, Strimmer K (2004). Ape: analyses of phylogenetics and evolution in R language. Bioinformatics.

[b74] Parham P, Ohta T (1996). Population biology of antigen presentation by mhc class i molecules. Science.

[b75] Paterson S, Wilson K, Pemberton JM (1998). Major histocompatibility complex variation associated with juvenile survival and parasite resistance in a large unmanaged ungulate population (ovis aries l.). Proc. Natl Acad. Sci.

[b76] Pfau RS, McBee RA, Van Den Bussche K, Lochmiller RL (1999). Allelic diversity at the mhc-dqa locus in cotton rats (*Sigmodon hispidus*) and a comparison of dqa sequences within the family muridae (mammalia: Rodentia). Immunogenetics.

[b77] Pinter AJ (1986). Population-dynamics and litter size of the montane vole, *Microtus-montanus*. Can. J. Zool. (Revue Canadienne De Zoologie).

[b78] Pond SLK, Posada D, Gravenor MB, Woelk CH, Frost SDW (2006). Automated phylogenetic detection of recombination using a genetic algorithm. Mol. Biol. Evol.

[b79] Posada D (2002). Evaluation of methods for detecting recombination from DNA sequences: empirical data. Mol. Biol. Evol.

[b80] Radwan J, Tkacz A, Kloch A (2008). Mhc and preferences for male odour in the bank vole. Ethology.

[b81] Radwan J, Biedrzycka A, Babik W (2010). Does reduced mhc diversity decrease viability of vertebrate populations?. Biol. Conserv.

[b82] Reusch TBH, Schaschl H, Wegner KM (2004). Recent duplication and inter-locus gene conversion in major histocompatibility class ii genes in a teleost, the three-spined stickleback. Immunogenetics.

[b83] Richman AD, Herrera LG, Nash D, Schierup MH (2003). Relative roles of mutation and recombination in generating allelic polymorphism at an mhc class ii locus in *Peromyscus maniculatus*. Genet. Res.

[b84] Royall R (1997). Statistical evidence: a likelihood paradigm.

[b85] Saccheri I, Kuussaari M, Kankare M, Vikman P, Fortelius W, Hanski I (1998). Inbreeding and extinction in a butterfly metapopulation. Nature.

[b86] Savage AE, Zamudio KR (2011). Mhc genotypes associate with resistance to a frog-killing fungus. Proc. Natl Acad. Sci.

[b87] Sawyer S (1989). Statistical tests for detecting gene conversion. Mol. Biol. Evol.

[b88] Schad J, Ganzhorn JU, Sommer S (2005). Parasite burden and constitution of major histocompatibility complex in the malagasy mouse lemur, *Microcebus murinus*. Evolution.

[b89] Sera WE, Early CN (2003). Microtus montanus. Mamm. Species.

[b90] Siddle HV, Kreiss A, Eldridge MDB, Noonan E, Clarke CJ, Pyecroft S (2007). Transmission of a fatal clonal tumor by biting occurs due to depleted mhc diversity in a threatened carnivorous marsupial. Proc. Natl Acad. Sci. USA.

[b91] Sokal RR, Rohlf FJ (1995). Biometry: the principles and practice of statistics in biological research.

[b92] Sommer S (2005). The importance of immune gene variability (mhc) in evolutionary ecology and conservation. Front. Zool.

[b93] Sommer S, Toto Volahy A, Seal US (2002). A population and habitat viability assessment for the highly endangered giant jumping rat (*Hypogeomys antimena*), the largest extant endemic rodent of madagascar. Anim. Conserv.

[b94] Spielman D, Brook BW, Frankham R (2004). Most species are not driven to extinction before genetic factors impact them. Proc. Natl Acad. Sci. USA.

[b95] Spurgin LG, Richardson DS (2010). How pathogens drive genetic diversity: Mhc, mechanisms and misunderstandings. Proc. Biol. Sci.

[b96] Srithayakumar V, Castillo S, Rosatte R, Kyle C (2011). Mhc class ii drb diversity in raccoons (*Procyon lotor*) reveals associations with raccoon rabies virus (lyssavirus). Immunogenetics.

[b97] Stenseth NC (1999). Population cycles in voles and lemmings: density dependence and phase dependence in a stochastic world. Oikos.

[b98] Sutton JT, Nakagawa S, Robertson BC, Jamieson IG (2011). Disentangling the roles of natural selection and genetic drift in shaping variation at mhc immunity genes. Mol. Ecol.

[b99] Takahata N (1990). A simple genealogical structure of strongly balanced allelic lines and trans-species evolution of polymorphism. Proc. Natl Acad. Sci.

[b100] Takahata N, Nei M (1990). Allelic genealogy under overdominant and frequency-dependent selection and polymorphism of major histocompatibility complex loci. Genetics.

[b101] Tamura K, Peterson D, Peterson N, Stecher G, Nei M, Kumar S (2011). Mega5: molecular evolutionary genetics analysis using maximum likelihood, evolutionary distance, and maximum parsimony methods. Mol. Biol. Evol.

[b102] Thompson JN (1998). Rapid evolution as an ecological process. Trends Ecol. Evol.

[b103] Timm RM, Tamarin RH (1985). Parasites of new world microtus. Biology of new world microtus.

[b104] Wegner KM, Kalbe M, Kurtz J, Reusch TBH, Milinski M (2003). Parasite selection for immunogenetic optimality. Science.

[b105] Wenink PW, Groen AF, Roelke-Parker ME, Prins HHT (1998). African buffalo maintain high genetic diversity in the major histocompatibility complex in spite of historically known population bottlenecks. Mol. Ecol.

[b106] Winternitz J, Yabsley M, Altizer S (2012). Parasite infection and host dynamics in a naturally fluctuating rodent population. Can. J. Zool.

[b107] Woelfing B, Traulsen A, Milinski M, Boehm T (2009). Does intra-individual major histocompatibility complex diversity keep a golden mean? Phil. Philos. Trans. R. Soc. Lond. B Biol. Sci.

[b108] Zagalska-Neubauer M, Babik W, Stuglik M, Gustafsson L, Cichon M, Radwan J (2010). 454 sequencing reveals extreme complexity of the class ii major histocompatibility complex in the collared flycatcher. BMC Evol. Biol.

[b109] Zheng X, Arbogast BS, Kenagy GJ (2003). Historical demography and genetic structure of sister species: Deermice (peromyscus) in the North American temperate rain forest. Mol. Ecol.

