# Supplementary material for: Duplication and population dynamics shape historic patterns of selection and genetic variation at the major histocompatibility complex in rodents
Source: Ecol Evol. 2013 Apr 22;3(6):1552–68. doi: 10.1002/ece3.567 (PMC3686191; doi:10.1002/ece3.567)
Supplement: Supplementary file 1 [file ece30003-1552-SD1.docx]

>AF312749_Peromyscus_maniculatus_isolate_SB1b_MHC_class_II_antigen_beta_chain_(Pema-EB)_mRNA_partial_cds

CAGAGCAUGCGGUUUCUGGAGAGAUACUUCUACAACCGGGAGGAGUACGUGCGCUUCGACAGCGACGUGGGCGAGUACCGCGAGGUGACCGAGCUGGGGCGGGGCAUCGCCGAGAACUUGAACAGCCAGAAGGAUCUCCUGGAGAACAGGCGGGCCGUGGUGAACACGGUG

>AF312750_Peromyscus_maniculatus_isolate_SQPM10b_MHC_class_II_antigen_beta_chain_(Pema-EB)_mRNA_partial_cds

CAGCGCGUGCGGUUUCUGGAGAGAUACAUCCACAACCGGGAGGAGAACUUGCGCUUCGACAGCGACGUGGGCGAGUACCGCGCGGUGACCGAGCUGGGGCGGCCGGACGCCGAGGACUGGAAUGGCCAGAAGGAUCUCCUGGAGCAGAAGCGGGCCCAGGUGGACAAUUAC

>AF312751_Peromyscus_maniculatus_isolate_SQPM15e_MHC_class_II_antigen_beta_chain_(Pema-EB)_mRNA_partial_cds

CAGCAUGUGCAGUAUCUGGUCAGAUACUCCUACAACCGGGAGGAGUACGUGCGCUUCGACAGCGACGUGGGCGAGUACCGUGCGGUGACCGAGCUGGGGCGGCCGGACGCCAAGUACUGGAACGGCCAGAAGGAGAUCCUGGAGCAGAAGCGGGCCGAGAUAGAGACGGUG

>AF312752_Peromyscus_maniculatus_isolate_SQPM20a_MHC_class_II_antigen_beta_chain_(Pema-EB)_mRNA_partial_cds

CAGAGCGUGCGGUAUCUGGUCAGACUCAUCUACAACCGGGAGGAGUUCGUGCGCUUCGACAGCGACGUGGGCGAGUUCAUCGCGGUGACCGAGCUGGGGCGGGGCAUAGCCGAGCACUUGAACAGCCAGAAGGAUCUCCUGGAGCGGUUGCGGGCCGAGAUAGAGACGGUG

>AF312753_Peromyscus_maniculatus_isolate_SQPM26a_MHC_class_II_antigen_beta_chain_(Pema-EB)_mRNA_partial_cds

CAGAGCGUGCGGUAUCUGGAGAGACACAUCUACAACCGGGAGGAGUUCAUUCGCUUCGACAGCGACGUGGGCGAGUACCGCGCGGUGACCGAGCUGGGGCGGGGCAUCGCCGAGUACUACAACAGCCAGAAGGAUUUCCUGGAGCAGACGCGGGCCUUGGUGGACAUUUAC

>AF312754_Peromyscus_maniculatus_isolate_SQPM23a_MHC_class_II_antigen_beta_chain_(Pema-EB)_mRNA_partial_cds

CAGCGUGUGCGGCUUCUAAACAGAUACUUCUACAACCGGGAGGAGUACGCUCGCUUCGACAGCGACGUGGGCGAGUACCGCGCGGUGACCGAGCUGGGGCGGCCCUCAGCCGAGUACUGGAACAGCCAGAAGGAGAUCCUGGAGAACAGACGGGCCGCGGUGGACAAUUAC

>AF312755_Peromyscus_maniculatus_isolate_SQPM25a_MHC_class_II_antigen_beta_chain_(Pema-EB)_mRNA_partial_cds

CAGCGCGUGCGGUAUCUGGAGAGACACAUCCACAACCGGGAGGAGUACGCUCGCUUCGACAGCGACGUGGGCGAGUUCAUCGCGCUGACCGAGCUGGGGCGGCCUGACGCCGAGUACUGGAACGGCCAGAAGGAGAUCCUGGAGGACAGGCGGGCCCAGGUGGACAAUUAC

>AF312756_Peromyscus_maniculatus_isolate_SQPM26b_MHC_class_II_antigen_beta_chain_(Pema-EB)_mRNA_partial_cds

CAGCGCGUGCGGUAUCUGGAGAGACACAUCCACAACCGGGAGGAGUACGCUCGCUUCGACAGCGACUUGGGCGAGUUCAUCGCGCUGACCGAGCUGGGGCGGCCUGACGCCGAGUACUGGAACGGCCAGAAGGAUCUCCUGGAGGACAGGCGGGCCCAGGUGGACAAUUAC

>AF312757_Peromyscus_maniculatus_isolate_SQPM19a_MHC_class_II_antigen_beta_chain_(Pema-EB)_mRNA_partial_cds

CAGCGCGUGCGGUUUCUGGAGAGACACAUCCACAACCGGGAGGAGUACGCUCGCUUCGACAGCGACGUGGGCGAGUACCGCGCGGUGACCGAGCUGGGGCGGCCUGACGCCGAGUACUGGAACGGCCAGAAGGAUCUCCUGGAGCACAAGCGGGCCCAGGUGGACAANNNN

>AF312758_Peromyscus_maniculatus_isolate_SQPM17c_MHC_class_II_antigen_beta_chain_(Pema-EB)_mRNA_partial_cds

CAGCGCGUGCGGUUUCUGGAGAGAUACAUCUACAACCGGGAGGAGUACGCUCGCUUCGACAGCGACGUGGGCGAGUACCGCGCGGUGACCGAGCUGGGGCGGCCUGACGCCGAGUACUGGAACGGCCAGAAGGAUUUCCUGGAGCAGAAGCGGGCCCAGGUGGACAAUUAC

>AF312759_Peromyscus_maniculatus_isolate_SQPM7g_MHC_class_II_antigen_beta_chain_(Pema-EB)_mRNA_partial_cds

CAGCGCGUGCGGUUUCUGGAGAGACGCAUCCACAACCGGGAGGAGUACGCUCGCUUCGACAGCGACGUGGGCGAGUUCCGUGCGGUGACCGAGCUGGGGCGGCCUGACGCCAAGUACUGGAACGGCCAGAAGGAUCUCCUGGAGGACAGGCGGGCCCAGGUGGACACGGUG

>AF312760_Peromyscus_maniculatus_isolate_SQPM2g_MHC_class_II_antigen_beta_chain_(Pema-EB)_mRNA_partial_cds

CAGCGUGUGCGGUAUCUGGAGAGACACAUCUACAACCGGGAGGAGUUCAUGCACUUUGACAGCGACGUGGGCGAGUUCAUCGCGGUGACCGAGCUGGGGCGGCCCAUAGCCGAGAACUACAACAGCCGGAAGGAGAUCCUGGAGCAGAAGCGGGCCGCGGUGGACUAUUAC

>AF312761_Peromyscus_maniculatus_isolate_SQPM15b_MHC_class_II_antigen_beta_chain_(Pema-EB)_mRNA_partial_cds

CAGCGCGUGCUGUAUCUGGAGAGACGCAUCCACAACCGGGAGGAGUACGCUCGCUUCGACAGCGACGUGGGCGAGUUCCGUGCGGUGACCGAGCUGGGGCGGCCUGACGCCAAGUACUGGAACGGCCAGAAGGAUCUCCUGGAGGACAGGCGGGCCUCGGUGGACAAUUAC

>AF312762_Peromyscus_maniculatus_isolate_SQPM24b_MHC_class_II_antigen_beta_chain_(Pema-EB)_mRNA_partial_cds

CAGCGCGUGCGGUAUCUGGAGAGACACAUCCACAACCGGGAGGAGUACGCUCGCUUCGACAGCGACGUGGGCGAGUUCAUCGCGCUGACCGAGCUGGGGCGGCCUGACGCCGAGUACUGGAACGGCCAGAAGGAUCUCCUGGAGGACAGGCGGGCCCAGGUGGACAAUUAC

>AF312763_Peromyscus_maniculatus_isolate_SQPM3b_MHC_class_II_antigen_beta_chain_(Pema-EB)_mRNA_partial_cds

CAGCGCGUGCGGUUUCUGUACAGAUACAUCUACAACCGGGAGGAGUUCCUGCGCUUCGACAGCGACGUGGGCGAGCACAUCGCGGUGACCGAGCUGGGGCGUCGGCAAGCCGAGUACGCAAACAGCCAGAAGGAUCUCCUGGAGCGGGCGCGGGCCGCGGUGGACAAUUAC

>AF312764_Peromyscus_maniculatus_isolate_SQPM11f_MHC_class_II_antigen_beta_chain_(Pema-EB)_mRNA_partial_cds

CAGCGCGUGCGGUUUCUGAACAGAUACAUCUACAACCGGGAGGAGUACGCNCGGUAUGACAGCGAUGUGGGCAAGUACAUUGCGCUGACCGAGUUGGGGCGGCCUGACGCCGAGUACUGGAACGGCCAGGAGGAUCUCCUGGAGCGGGCGCGGGCCGCGGUGGACAAUUAC

>AF516929_Peromyscus_maniculatus_MHC_class_II_antigen_beta_chain_(Pema-EB)_mRNA_Pema-EB*CP14a_allele_partial_cds

CAGCGCGUGCAGUUUCUGGACAGAUACUUCUACAACCGGGAGGAGUACGUGCGCUUCGACAGCGACGUGGGCGAGUUCAUCGCGCUGACUGAGCUGGGGCGGCCUGACGCCGAGUACUGGAACAGCCAGAAGGAUUUCCUGGAGGACAGGCGGGCCGUGGUGGACACAGCG

>AF516930_Peromyscus_maniculatus_MHC_class_II_antigen_beta_chain_(Pema-EB)_mRNA_Pema-EB*CP11a_allele_partial_cds

CAGCGCGUGCAGUUUCUGGACAGAUACUUCUACAACCGGGAGGAGUUCGUGCGCUUCGACAGCGACGUGGGCGAGUUCCGCGCGGUGACCGAGCUGGGGCGGGGCAUCGCCGAGAACUGGAACGGCCAGAAGGAUAUCCUGGAGGACAGGCGGGCCGUGGUGGACACAGCG

>AF516931_Peromyscus_maniculatus_MHC_class_II_antigen_beta_chain_(Pema-EB)_mRNA_Pema-EB*CP16a_allele_partial_cds

CAGCGCGUGCGGUUUCUGGAGAGACACAUCCACAACCGGGAGGAGUACGCUCGCUUCGACAGCGACGUGGGCGAGUACCGCGCGGUGACCGAGCUGGGGCGGCCUGACGCCAAGUACUGGAACGGCCAGAAGGAUCUCCUGGAGGACAGGCGGGCCCAGGUGGACAAUUAC

>AF516932_Peromyscus_maniculatus_MHC_class_II_antigen_beta_chain_(Pema-EB)_mRNA_Pema-EB*CP17a_allele_partial_cds

CAGAGCGUGCGGUAUCUGGAGAGAUACUUCUACAACCGGGAGGAGUACGCUCGCUUCGACAGCGACGUGGGCGAGUUCCGCGCGGUGAACGAGCUGGGGCGGCCUGACGCCAAGUACUGGAACGGCCAGGAGGAUCUCCUGGAGCAGAAGCGGGCCGCGAUAGAGACCUGG

>AF516933_Peromyscus_maniculatus_MHC_class_II_antigen_beta_chain_(Pema-EB)_mRNA_Pema-EB*CP10a_allele_partial_cds

CAGCGCGUGCAGUAUCUGGUCAGAUACAUCUACAACCGGGAGGAGUACGUGCGCUUCGACAGCGACGUGGGCGAGUUCCGUGCGGUGAACGAGCUGGGGCGGCCCUCAGCCAAGUACUGGAACGGCCAGAAGGACUUCAUGGAGCAGAAGCGGGCCGAGAUAGAGACGGUG

>AF516934_Peromyscus_maniculatus_MHC_class_II_antigen_beta_chain_(Pema-EB)_mRNA_Pema-EB*CP22a_allele_partial_cds

CAGCGCGUGCGGUAUCUGGUCAGAUACAUCCACAACCGGGAGGAGAACGUGCGCUUCGACAGCGACGUGGGCGAGUUCAUCGUGGUGACCGAGCUGGGGCGGCUGGACGCCGAGAACUGGAACAGCCAGAAGGAUCUCCUGGAGCAGAAGCGGGCUGAGAUAGAGACCGUG

>AF516935_Peromyscus_maniculatus_MHC_class_II_antigen_beta_chain_(Pema-EB)_mRNA_Pema-EB*CP20a_allele_partial_cds

CAGCGUGUGCGGUAUCUGGAGAGACACAUCUACAACCGGGAGGAGUUCAUGCACUUUGACAGCGACGUGGGCGAGUUCAUCGCGCUGACCGAGCUGGGGCGGCCCAUAGCCGAGAACUACAACAGCCGGAAGGAGAUCCUGGAGCAGAAGCGGGCCGCGGUGGACUAUUAC

>AF516936_Peromyscus_maniculatus_MHC_class_II_antigen_beta_chain_(Pema-EB)_mRNA_Pema-EB*CP19b_allele_partial_cds

CAGCGCGUGCGGUUUCUAGAGAGAUACUUCUACAACCGGGAGGAGUACGUGCGCUUCGACAGCGACGUGGGCGAGUACCGCGAGGUGACCGAGCUGGGGCGGGGCAUGGCCGAGAACUUGAACAGCCAGAAGGAUCUCCUGGAGAACAGGCGGGCCCAGGUGGACACGGUG

>AF516937_Peromyscus_maniculatus_MHC_class_II_antigen_beta_chain_(Pema-EB)_mRNA_Pema-EB*CP1a_allele_partial_cds

CAGCGCGUGCGGUUUCUGGAGAGACACAUCCACAACCGGGAGGAGUACGCUCGCUUCGACAGCGACGUGGGCGAGUACCGCGCGGUGACCGAGCUGGGGCGGCCUGACGCCGAGUACUGGAACGGCCAGAAGGAUCUCCUGGAGCAGAAGCGGGCCCAGGUGGACAAUUAC

>AF516938_Peromyscus_maniculatus_MHC_class_II_antigen_beta_chain_(Pema-EB)_mRNA_Pema-EB*CP5b_allele_partial_cds

CAGCGCGUGCGGUUUCUGGACAGAUUCAUCUACAACCGGGAGGAGUACGCGCGGUACGACAGCGAUGUGGGCAAGUACAUUGCGCUGACCGAGCUGGGGCGGGCUGACGCCGAGUACUGGAACGGCCAGGAGGAUCUCCUGGAGCGGGCGCGGGCCGCGGUGGACAAUUAC

>AF516939_Peromyscus_maniculatus_MHC_class_II_antigen_beta_chain_(Pema-EB)_mRNA_Pema-EB*CP3a_allele_partial_cds

CAGAGCGUGCGGUAUCUGGAGAGACACAUCUACAACCGGGAGGAGUUCAUGCGCUUCGACAGCGACGUGGGCGAGUACCGCGCGGUGACCGAGCUGGGGCGGGGCAUCGCCGAGUACUACAACAGCCAGAAGGAUUACCUGGAGCAGACGCGGGCCUCGGUGGACAUUUAC

>AF516946_Peromyscus_maniculatus_MHC_class_II_antigen_beta_chain_(Pema-EB)_mRNA_Pema-EB*CP4a_allele_partial_cds

CAGCAUGUGCAGUAUCUGGUCAGAUACUUCUACAACCGGGAGGAGUACGUGCGCUUCGACAGCGACGUGGGCGAGUACCGUGCGGUGACCGAGCUGGGGCGGCCGGACGCCAAGUACUGGAACGGCCAGAAGGAGAUCCUGGAGCAGAAGCGGGCCGAGAUAGAGACGGUG

>AJ416074_Hypogeomys_antimena_DRB_gene_for_MHC_class_II_antigen_A_allele_exon_2

CAGCGCGTGCGGGTTCTACAGAGATACATCCANAACCAGGAGGAGTTCGTGCGCTTCGATAGCAAAGTGGGCCTGTACATTGCAGTGACCGAGCTGGGGCGTCCTGTCGCCAAGTACGGGAACAGCCAGAAGGAGACCCTGCAGTACCTGCAGGGCACAGTGAACACGTTC

>AJ416075_Hypogeomys_antimena_DRB_gene_for_MHC_class_II_antigen_B_allele_exon_2

CAGCGCGTGCGGTTTCTACAGAGATACATCTACAACCAGGAGGAGTACGCGCGCTTCGACAGCGACGTGGGCGAGTTCATTGCGGTGACCGAGCTGGGGCGTCCGGATGCCGAGTACTGGAACAGCCAGAAGGAGATCCTGGAGGACAAGCGGGCCGCAGTGGACACTTTC

>AJ416076_Hypogeomys_antimena_DRB_gene_for_MHC_class_II_antigen_C_allele_exon_2

CAGCGCGTGCGGTTTCTACAGAGATACATCCACAACCAGGAGGAGTTCGTGCGCTTCGACAGCGACGTGGGCGAGTTCATTGCGGTGACCGAGCTGGGGCGTCCGAGCGCCGAGTACGGGAACAGCCAGAAGGAGATCCTGGAGGACGAGCGGGCCACAGTGGACACTTTC

>AJ416077_Hypogeomys_antimena_DRB_gene_for_MHC_class_II_antigen_D_allele_exon_2

CAGCGCGTGCGGGTTCTACAGAGATACATCCACAACCAGGAGGAGTTCGTGCGCTTCGATAGCAAAGTGGGCCTGTACATTGCAGTGACCGAGCTGGGGCGTCCTGTCGCCAAGTACGGGAACAGCCAGAAGGAGACCCTGGAGCACCTGCGGGGCACAGTGAACACGTTC

>AJ431271_Rattus_rattus_partial_drb_gene_for_MHC_class_II_drb-A_allele_exon_2

CAGCGAGTGAGGTATCTGGAGAGATACTTCTACAACCGAGAGGAGTACGCGCGCTTCGACAGCGACGTGGGCGAGTACCGCGCGGTGACCGAGCTGGGGCGGCCGGACGCCGAGTACTGGAACAGCCAGAAGGAGCTCCTGGAGCAGAAGCGGGCCGAGGTGGACACGTAC

>AJ431272_Rattus_rattus_partial_drb_gene_for_MHC_class_II_drb-B_allele_exon_2

CAGCGCGTGCGGTTTCTGTCCAGATACATCTACAACCGGGAGGAGTACGCGCGCTTCGACAGCGACNTGGGCGAGTTCCGCGCGGTGACCGAGCTGGGGCGGCCCTCAGCCGAGTACTGGAACAGCCAGAAGGAGACTCTGGAGCGTCATCGGGCCGCGGTGGNCACGTAC

>AJ431273_Rattus_rattus_partial_drb_gene_for_MHC_class_II_drb-C_allele_exon_2

CAGCGCGTGCGGTTTCTGGAGAGATACATCCACAACCGGGAGGAGTTTCTGCGCTTCGACAGCGACGTGGGCGAGTACCGCGCGGTGACCGAGCTGGGGCGGCCGGACGCCGAGTCCTGGAACAAACAGAAGGAGTTCATGGAGCAGAAGCGGGCCAAGGTGGACACGTAC

>AJ431274_Rattus_rattus_partial_drb_gene_for_MHC_class_II_drb-D_allele_exon_2

CAGCGCGTGCGGCTTCTGGTCAGAGACATCTACAACCGAGAGGAGGTCGTGCGCTTCGACAGCGACGTGGGCGAGTACCGCGCCGTGACCGAGCTGGGGCGGCCGGACGCCGAGTACTGGAACAGCCAGAAGGAGATCTGGGAGCAGAAGCGGGCCGAGGTGGACACGGTC

>AJ431275_Rattus_rattus_partial_drb_gene_for_MHC_class_II_drb-E_allele_exon_2

CAGCGCGTGAGGTATCTGGACAGATACATCTACAACCGAGAGGAGTACGCGCGCTTCGACAACGAGGTGGGCGAGTACCGCGCGGTGACCGAGCTGGGGCGGGGCATAGCCGAGAACTTGAACAAACAGAAGGACTACATGGAGCAGACGCGGGCCGCGGTGGACACGTAC

>AY693603_Gerbillurus_paeba_MHC_class_II_antigen_(Gepa-DRB)_gene_Gepa-DRB*1_allele_exon_2_and_partial_cds

GAGCGAGTGCGGTATCTGGACAGATACTTCTACCACCGGCAGGAATACGTGCGCTTCGACAGCGACGTGGGCGAGTACCGCGCGGTGACCGAGCTGGGGCGGCCGGACGCCGAGTACTGGAACAGCCAGAAGGACCTCCTGGAGCAGAAGCGGGCCAATGTGGACACGTAC

>AY693604_Gerbillurus_paeba_MHC_class_II_antigen_(Gepa-DRB)_gene_Gepa-DRB*2_allele_exon_2_and_partial_cds

GAGCGAGTGCGGTATCTGCAAAGATACTTCTACAACCGGCAGGAATACGTGCGCTTCGACAGCGACGTGGGCGAGTTCCGCGCGGTGACCGAGCTGGGGCGGGGCATAGACAAGAACTTGAACAGCCAGAAGGAGATCCTGGAGCGTCTGCGGGCCGAGGTGGACAGGTTC

>AY693605_Gerbillurus_paeba_MHC_class_II_antigen_(Gepa-DRB)_gene_Gepa-DRB*3_allele_exon_2_and_partial_cds

GAGCGAGTGCGGCTTCTGGCCAGATNGNTCTACAACCGGCAGGAGTTGGTGCGCTTCGACAGCGACGTGGGCGAGTTCCGCGCGGTGACCGAGCTGGGGCGGCCCTCAGCCGAGTCCTGGAACAGCCAGAAGGACCTCCTGGAGCGGGCGCGGGCCTTGGTGGACACGGGG

>AY693606_Gerbillurus_paeba_MHC_class_II_antigen_(Gepa-DRB)_gene_Gepa-DRB*4_allele_exon_2_and_partial_cds

GAGCGAGTGCGGTTTCTGGACAGATACTTCTACCACCGGCAGGAGTTCGTGCGCTTCGACAGCGACGTGGGCGAGTTCCGCGCGGTGACCGAGCTGGGGCGGCCGGACGCCGAGTACTTGAACAGCCGGAAGGACCTCCTGGAGCAGCGGCGGGCCTTGGTGGACACGTAC

>AY693607_Gerbillurus_paeba_MHC_class_II_antigen_(Gepa-DRB)_gene_Gepa-DRB*5_allele_exon_2_and_partial_cds

GAGCGAGTGCGGTATCTGGACAGATACTTCTACAACCGGCAGGAATACGCGCGCTTCGACAGCGACGTGGGCGAGTTCCGCGCGGTGAACGAGCTGGGGCGGCCGGACGCCGAGTACTGGAACAGCCAGAAGGAGATCCTGGAGCATAAGCGGGCCGAGGTGGACACGGTG

>AY693608_Gerbillurus_paeba_MHC_class_II_antigen_(Gepa-DRB)_gene_Gepa-DRB*6_allele_exon_2_and_partial_cds

GAGCGAGTGCGGTATCTGCACAGATACTTCTACAACCGGCAGGAGTTCGTGCGCTTCGACAGCGACGTGGGCGAGTTCCGCGCGGTGAACGAGCTGGGGCGGCCAGTCGCCGAGTACTTGAACAGCCAGAAGGAGATCCTGGAGCAGCTGCGGGCCAAGGTGGACACGTAC

>AY693609_Gerbillurus_paeba_MHC_class_II_antigen_(Gepa-DRB)_gene_Gepa-DRB*7_allele_exon_2_and_partial_cds

GAGCGAGTGCGGCTTCTGCACAGACACTTCTACAACCGGCAGGAATACGTGCGCTTCGACAGCGACGTGGGCGAGTTCCGCGCGGTGACCGAGCTGGGGCGGCCCTCAGCCGAGTACTGGAACAGCCAGAAGGACCTCCTGGAGCGTATGCGGGCCGAGGTGGACACGTAC

>AY693610_Gerbillurus_paeba_MHC_class_II_antigen_(Gepa-DRB)_gene_Gepa-DRB*8_allele_exon_2_and_partial_cds

GAGCGAGTGCGGTATCTGGACAGATACTTCTACCACCGGCAGGAATACGTGCGCTTCGACAGCGACGTGGGCGAGTACCGCGCGGTGACCGAGCTGGGGCGGCACTCAGCCGAGTACTGGAACAGCCAGAAGGACCTCCTGGAGCAGAAGCGGGCCAATGTGGACACGTAC

>AY693612_Gerbillurus_paeba_MHC_class_II_antigen_(Gepa-DRB)_gene_Gepa-DRB*10_allele_exon_2_and_partial_cds

GAGCGAGTGCGGTATCTGGCCAGATACTTCTACAACCGGCAGGAATACGTGCGCTTCGACAGCGACGTGGGCGAGTTCCGCGCGGTGACCGAGCTGGGGCGGCCGGACGCCGAGTACTGGAACAGCCAGAAGGAGATCCTGGAGCAGCATCGGGCCTTGGTGGACACGGGG

>AY693613_Gerbillurus_paeba_MHC_class_II_antigen_(Gepa-DRB)_gene_Gepa-DRB*11_allele_exon_2_and_partial_cds

GAGCGAGTGCGGTATCTGCAAAGATACTTCTACAACCGGCAGGAGTTCGTGCGCTTCGACAGCGACGTGGGCGAGTACCGCGCGGTGACCGAGCTGGGGCGGAGCATAGACAAGAACTGGAACAGCCGGAAGGAGATCCTGGAGCGTCTGCGGGCCGAGGTGGACAGGTTC

>AY693614_Gerbillurus_paeba_MHC_class_II_antigen_(Gepa-DRB)_gene_Gepa-DRB*12_allele_exon_2_and_partial_cds

GAGCGAGTGCGGTATCTGCAAAGATACTTCTACAACCGGCAGGAATACGTGCGCTTCGACAGCGACGTGGGCGAGTTCCGCGCGGTGACCGAGCTGGGGCGGGGCATAGACAAGAACTGGAACAGCCAGAAGGACCTCCTGGAGCAGAAGCGGGCCGAGGTGGACAGGTTC

>AY693615_Gerbillurus_paeba_MHC_class_II_antigen_(Gepa-DRB)_gene_Gepa-DRB*13_allele_exon_2_and_partial_cds

GAGCGAGTGCGGTTTCTGGACAGATGCTTCTACAACCGGCAGGAATACGTGCGCTTCGACAGCGACGTGGGCGAGTTCCGCGCGGTGACCGAGCTGGGGCGGCCGGACGCCGAGTACTGGAACAGCCAGAAGGACTTCCTGGAGCATGCGCGGGCCTTGGTGGACACGTTC

>AY693616_Gerbillurus_paeba_MHC_class_II_antigen_(Gepa-DRB)_gene_Gepa-DRB*14_allele_exon_2_and_partial_cds

GAGCGAGTGCGGTATCTGGCCAGATGCTTCTACAACCGGCAGGAGTTGGTGCGCTTCGACAGCGACGTGGGCGAGTTCCGCGCGGTGACCGAGCTGGGGCGGGGCATAGACAAGAACTTGAACAGCCAGAAGGACCTCCTGGAGCAGAAGCGGGCCTTGGTGGACACGTAC

>AY693617_Gerbillurus_paeba_MHC_class_II_antigen_(Gepa-DRB)_gene_Gepa-DRB*15_allele_exon_2_and_partial_cds

GAGCGAGTGCGGTTTCTGGAAAGACACTTCTACCACCGGCAGGAGTTCCTGCGCTTCGACAGCGACGTGGGCGAGTTCCGCGCGGTGACCGAGCTGGGGCGGCCGGACGCCGAGTACTTGAACAGCCAGAAGGAGATCCTGGAGCAGCGGCGGGCCGCGGTGGACACGGTG

>AY693618_Gerbillurus_paeba_MHC_class_II_antigen_(Gepa-DRB)_gene_Gepa-DRB*16_allele_exon_2_and_partial_cds

GAGCGAGTGCGGCTTCTGCACAGACACTTCTACAACCGGCAGGAATACGCGCGCTTCGACAGCGACGTGGGCGAGTACCGCGCGGTGACCGAGCTGGGGCGGCCGGACGCCGAGTCCTGGAACAGCCAGAAGGACCTCCTGGAGCAGCATCGGGCCTTGGTGGACACGGGG

>AY693619_Gerbillurus_paeba_MHC_class_II_antigen_(Gepa-DRB)_gene_Gepa-DRB*17_allele_exon_2_and_partial_cds

GAGCGAGTGCGGTTTCTGGACAGATACTTCTACCACCGGCAGGAATACGTGCGCTTCGACAGCGACGTGGGCGAGTTCCGCGCGGTGAACGAGCTGGGGCGGCCGGACGCCGAGTACTGGAACAGCCTGAAGGACTTCATGGAGCGTCNGCGGGCCGAGGTGGACACGTAC

>AY693620_Gerbillurus_paeba_MHC_class_II_antigen_(Gepa-DRB)_gene_Gepa-DRB*18_allele_exon_2_and_partial_cds

GAGCGAGTGCGGTATCTGGACAGATACTTCTACCACCGGCAGGAGAACGTGCGCTTCGACAGCGACGTGGGCGAGTTCCGCGCGGTGACCGAGCTGGGGCGGCCGGACGCCGAGTACTGGAACAGCCAGAAGGAGATCCTGGAGCAGGAGCGGGCCGAGGTGGACACGTAC

>AY693621_Gerbillurus_paeba_MHC_class_II_antigen_(Gepa-DRB)_gene_Gepa-DRB*19_allele_exon_2_and_partial_cds

GAGCGAGTGCGGCTTCTGCACAGACACTTCTACAACCGGCAGGAATACGTGCGCTTCGACAGCGACGTGGGCGAGTACCGCGCGGTGACCGAGCTGGGGCGGCCGGACGCCGAGTACTGGAACAGCCTGAAGGACTTCATGGCGCGTATGCGGGCCGAGGTGGACACGTAC

>AY693622_Gerbillurus_paeba_MHC_class_II_antigen_(Gepa-DRB)_gene_Gepa-DRB*20_allele_exon_2_and_partial_cds

GAGCGAGTGCGGTATCTGGACAGATACTTCTACCACCGGCAGGAGTTCGTGCGCTTCGACAGCGACGTGGGCGAGTTCCGCGCGGTGACCGAGCTGGGGCGGGGCATAGACAAGAACTGGAACAGCCAGAAGGACTTCCTGGAGGAGAAGCGGGCCAAGGTGGACACGTAC

>AY693623_Gerbillurus_paeba_MHC_class_II_antigen_(Gepa-DRB)_gene_Gepa-DRB*21_allele_exon_2_and_partial_cds

GAGCGAGTGCGGTTTCTGGAAAGATACTTCTACCACCGGCAGGAATACGCGCGCTTCGACAGCGACGTGGGCGAGTACCGCGCGGTGACCGAGCTGGGGCGGCCGGACGCCGAGTACTGGAACAGCCAGAAGGACCTCCTGGAGGAGAAGCGGGCCAAGGTGGACACGTAC

>AY693624_Gerbillurus_paeba_MHC_class_II_antigen_(Gepa-DRB)_gene_Gepa-DRB*22_allele_exon_2_and_partial_cds

GAGCGAGTGCGGTTTCTGGAAAGATACTTCTCCAACCGGCAGGAGTACGTGCGCTTCGACAGCGACGTGGGCGAGTTCCGCGCGGTGACCGAGCTGGGGCGGCCGGACGCCGAGTACTGGAACAGCCAGAAGGACATCCTGGAGCAGAAGCGGGCCGATGTGGACACGTAC

>AY693625_Gerbillurus_paeba_MHC_class_II_antigen_(Gepa-DRB)_gene_Gepa-DRB*23_allele_exon_2_and_partial_cds

GAGCGAGTGCGGTTTCTGGACAGATACTTCTACCACCGGCAGGAGTTCGTGCGCTTCGACAGCGACGTGGGCGAGTTCCGCGCGGTGACCGAGCTGGGGCGGCCGGACGCCGAGTACTTGAACAGCCGGAAGGACCTCCTGGAGCAGCGGCGGGCCGAGGTGGACACGTAC

>AY693626_Gerbillurus_paeba_MHC_class_II_antigen_(Gepa-DRB)_gene_Gepa-DRB*24_allele_exon_2_and_partial_cds

GAGCGAGTGCGGTATCTGCAAAGATACTTCTACAACCGGCAGGAATACGTGCGCTTCGACAGCGACGTGGGCGAGTACCGCGCGGTGACCGAGCTGGGGCGGCCGGACGCCGAGTACTGGAACAGCCAGAAGGACCTCCTGGAGCAGCGGCGGGCCGAGGTGGACACGTAC

>AY693627_Gerbillurus_paeba_MHC_class_II_antigen_(Gepa-DRB)_gene_Gepa-DRB*25_allele_exon_2_and_partial_cds

GAGCGAGTGCGGTATCTGGCCAGATACTTCTACAACCGGCAGGAATACGTGCGCTTCGACAGCGACGTGGGCGAGTTCCGCGCGGTGAACGAGCTGGGGCGGCACTCAGCCGAGTACTGGAACAGCCAGAAGGACTTCCTGGAGCAGCGGCGGGCCGAGGTGGACACGGTG

>AY693628_Gerbillurus_paeba_MHC_class_II_antigen_(Gepa-DRB)_gene_Gepa-DRB*26_allele_exon_2_and_partial_cds

GAGCGAGTGCGGTATCTGAAAAGATACTTCTACCACCGGCAGGAATACGCGCGCTTCGACAGCGACGTGGGCGAGTACCGCGCGGTGACCGAGCTGGGGCGGCCGGACGCCGAGTACTGGAACAGCCAGAAGGACCTCCTGGAGCAGCGGCGGGCCGAGGTGGACACGTAC

>AY693629_Gerbillurus_paeba_MHC_class_II_antigen_(Gepa-DRB)_gene_Gepa-DRB*27_allele_exon_2_and_partial_cds

GAGCGAGTGCGGTTTCTGGACAGATACTTCCACCACCGGCAGGAATACGTGCGCTTCGACAGCGACGTGGGCGAGTTCCGCGCGGTGACCGAGCTGGGGCGGCACTCAGCCGAGTACTGGAACAGCCAGAAGGAGATCCTGGAGCAGCGGCGGGCCGCGGTGGACACGTAC

>AY693630_Gerbillurus_paeba_MHC_class_II_antigen_(Gepa-DRB)_gene_Gepa-DRB*28_allele_exon_2_and_partial_cds

GAGCGAGTGCGGTTTCTGGAAAGATACTTCTACCACCGGCAGGAATACGTGCGCTTCGACAGCGACGTGGGCGAGTTCCGCGCGGTGACCGAGCTGGGGCGGCACTCAGCCGAGTACTGGAACAGCCTGAAGGACTTCCTGGAGCAGCGGCGGGCCGAGGTGGACACGTAC

>AY693631_Gerbillurus_paeba_MHC_class_II_antigen_(Gepa-DRB)_gene_Gepa-DRB*29_allele_exon_2_and_partial_cds

GAGCGAGTGCGGCTTCTGCACAGACACTTCTACAACCGGCAGGAATACGTGCGCTTCGACAGCGACGTGGGCGAGTACCGCGCGGTGACCGAGCTGGGGCGGCCGGACGCCGAGTACTGGAACAGCCTGAAGGACTTCATGGTGCGTCTGCGGGCCGAGGTGGACACGTAC

>AY693632_Gerbillurus_paeba_MHC_class_II_antigen_(Gepa-DRB)_gene_Gepa-DRB*30_allele_exon_2_and_partial_cds

GAGCGAGTGCGGTTTCTGGACAGATGCTTCTACAACCGGCAGGAATACGCGCGCTTCGACAGCGACGTGGGCGAGTTCCGCGCGGTGACCGAGCTGGGGCGGCCGGACGCCGAGTACTGGAACAGCCTGAAGGACATCATGGAGCAGCGGCGGGCCGNGGTGGACACGTTC

>AY693633_Gerbillurus_paeba_MHC_class_II_antigen_(Gepa-DRB)_gene_Gepa-DRB*31_allele_exon_2_and_partial_cds

GAGCGAGTGCGGCTTCTGGCCAGATACTTCTACAACCGGCAGGAGTTCCTGCGCTTCGACAGCGACGTGGGCGAGTTCCGCGCGGTGACCGAGCTGGGGCGGGGCATAGACAAGAACTTGAACAGCCAGAAGGACCTCCTGGAGCGGCGGCGGGCCGCGGTGGACACGCAC

>AY693634_Gerbillurus_paeba_MHC_class_II_antigen_(Gepa-DRB)_gene_Gepa-DRB*32_allele_exon_2_and_partial_cds

GAGCGAGTGCGGTTTCTGGAAAGATACTTCTACAACCGGCAGGAATACGTGCGCTTCGACAGCGACGTGGGCGAGTTCCGCGCGGTGACCGAGCTGGGGCGGCCGGACGCCGAGTACTGGAACAGCCAGAAGGACCTCCTGGAGCAGCGGCGGGCCGAGGTGGACACGTAC

>AY693635_Gerbillurus_paeba_MHC_class_II_antigen_(Gepa-DRB)_gene_Gepa-DRB*33_allele_exon_2_and_partial_cds

CAGCGGGTGCGGTACCTGGACAGATACATCTACAACCAGGAGGAGTTCGTGCGCTTCGACAGCGACGTGGGCGAGTACCGGGCGGTGACGGAGCTGGGCCGGCCGGACGCCGAGTACTGGAACAGCCGGCAGGACATCATGGAGNNGANACGGGCAGAGGTGGACACGNTG

>AY693636_Gerbillurus_paeba_MHC_class_II_antigen_(Gepa-DRB)_gene_Gepa-DRB*34_allele_exon_2_and_partial_cds

CAGCGGGTGCGGTTCCTGGAGAGATACATCTACAACCGCGAGGAGTTCGTGCGCTTCGACAGCGACGTGGGCGAGTACCGGGCGGTGACGGAGCTGGGCCGGCCGGACGCCGAGTACTGGAACCGCCAGCAGGACATCCTGGAGGACGAGCGGGCCAAGGTGGACACGTNC

>AY699719_Apodemus_sylvaticus_MHC_class_II_antigen_(Apsy-DRB)_gene_Apsy-DRB*01_allele_exon_2_and_partial_cds

CAGCGCGTGCAGCTTCTGCAGAGATACATCTACAACCGGGAGGAGTTCGTGCGCTTCGACAGCGACGTGGGCAAGTTCCGCGCGGTGACCGAGCTGGGGCGGCCGGACGCCGAGTACCTCAACAGCCAGAAGGAGATCCTGGAGCAGCTGCGGGCCGCGGTGGACACGTTC

>AY699720_Apodemus_sylvaticus_MHC_class_II_antigen_(Apsy-DRB)_gene_Apsy-DRB*02_allele_exon_2_and_partial_cds

CAGCGCGTGCACTATCTGGTCAGATTCATCTACAACCAGGAGGAGTTCGTGCGCTTCGACAGCGACGTGGGCGAGTTCCGCGCGGTGACCGAGCTGGGGCGGCCGGACGCCGAGTACTTCAACAGCCAGAAGGAGATCATGGAGCGGACGCGGGCCGAGGTGGACACGTTC

>AY699721_Apodemus_sylvaticus_MHC_class_II_antigen_(Apsy-DRB)_gene_Apsy-DRB*03_allele_exon_2_and_partial_cds

CAGCACGTGCAGTTTCTGGCCAGATTAATCTACAACCGGGAGGAGTACGTGCGCTTCGACAGCGACGTGGGCGAGTTCCGCGCGGTGACCGAGCTGGGGCGGCGCTCAGCCGAGTACTTCAACAGCCAGAAGGACTACATGGAGCGGACGCGGGCCGCGGTGGACACGGTG

>AY699722_Apodemus_sylvaticus_MHC_class_II_antigen_(Apsy-DRB)_gene_Apsy-DRB*04_allele_exon_2_and_partial_cds

CAGCGCGTGCAGCTTCTGGTCAGATTTATCTACAACCGGGAGGAGTTCGTGCGCTTCGACAGCGACGAGGGCGAGTTCCGCGCGGTGACCGAGCTGGGGCGGGGCATAGCCGAGTACCTCAACAGCCAGAAGGACTTCATGGAGCGGACGCGGGCCGAGGTGGACACGGTG

>AY699723_Apodemus_sylvaticus_MHC_class_II_antigen_(Apsy-DRB)_gene_Apsy-DRB*05_allele_exon_2_and_partial_cds

CAGCGCGTGCAGCTTCTGGCCAGATTAATCTACAACCAGGAGGAGTACGTGCGCTTCGACAGCGACGTGGGCGAGTTCCGCGCGGTGACCGAGCTGGGGCGGCGCTCAGCCGAGTACTTCAACAGCCAGAAGGACTTCATGGAGCGGACGCGGGCCGTGGTGGACACGGGG

>AY699724_Apodemus_sylvaticus_MHC_class_II_antigen_(Apsy-DRB)_gene_Apsy-DRB*06_allele_exon_2_and_partial_cds

CAGCGCGTGCACTATCTGGTCAGACACTTCTACAACCAGGAGGAGTTCGTGCGCTTCGACAGCGACGTGGGCGAGTACCGCGCGGTGACCGAGCTGGGGCGGGGCATAGCCGAGGACTGGAACAGCCAGAAGGACTTCATGGAGCGGAGGCGGGCCGAGGTGGACACGGTG

>AY699725_Apodemus_sylvaticus_MHC_class_II_antigen_(Apsy-DRB)_gene_Apsy-DRB*07_allele_exon_2_and_partial_cds

CAGCGCGTGCAGTTTCTGCAGAGATACATCTACAACCGGGAGGAGTTCGTGCGCTTCGACAGCGACGTGGGCGAGTACCGCGCGGTGACCGAGCTGGGGCGGCGGGACGCCGAGTACTTCAACAGCCAGAAGGAGCTCCTGGAGCAGAAGCGGGCCGCGGTGGACACGTTC

>AY699726_Apodemus_sylvaticus_MHC_class_II_antigen_(Apsy-DRB)_gene_Apsy-DRB*08_allele_exon_2_and_partial_cds

CAGCGCGTGCAGCTTCTGGCCAGATTCATCTACAACCAGGAGGAGTACGTGCGCTTCGACAGCGACGTGGGCGAGTTCCGCGCGGTGACCGAGCTGGGGCGGCGGGACGCCGAGTACTGGAACAGTCAGAAGGACTACGTGGAGCGGCTGCGGGCCGCGGTGGACACGTAC

>AY699727_Apodemus_sylvaticus_MHC_class_II_antigen_(Apsy-DRB)_gene_Apsy-DRB*09_allele_exon_2_and_partial_cds

CAGCGCGTGCAGCTTCTGCAGAGATACATCTACAACCGGGAGGAGTTCGTGCGCTTCGACAGCGACGTGGGCAAGTTCCGCGCGGTGACCGAGCTGGGGCGGCCGGACGCCGAGTACCTCAACAGCCAGAAGGAGATCCTGGAGCAGAAGCGGGCCGAGGTGGACACGTTC

>AY699728_Apodemus_sylvaticus_MHC_class_II_antigen_(Apsy-DRB)_gene_Apsy-DRB*10_allele_exon_2_and_partial_cds

CAGCGCGTGCAGTTTCTGGAGAGATACATCTACAACCGGGAGGAGTACGCGCGCTTCGACAGCGACGTGGGCGAGTACCGCGCGGTGACCGAGCTGGGGCGGCCGGACGCCGAGTACTGGAACAGCCAGAAGGAGCTCCTGGAGCAGAAGCGGGCCCGGGTGGACACGTAC

>AY699729_Apodemus_sylvaticus_MHC_class_II_antigen_(Apsy-DRB)_gene_Apsy-DRB*11_allele_exon_2_and_partial_cds

CAGCGCATACGGTACGTGACCAGATACATCTACAACCAGGAGGAGTTCGTGCGCTTCGACAGCGACGTGGACGAGTACCGCGCGGTGACCGAGCTGGGGCGGCCGGACGCCGAGTACTGGAACAGCCGGAAGGAGATCCTGGAGCGGACGCGGGCCGAGGTGGACACGTTC

>AY699730_Apodemus_sylvaticus_MHC_class_II_antigen_(Apsy-DRB)_gene_Apsy-DRB*12_allele_exon_2_and_partial_cds

CAGCGCGTGCACTATCTGGTCAGATTCTTCTACAACCGGGAGGAGTACGTGCGCTTCGACAGCGACGTGGGCGAGTTCCGCGCGGTGACCGAGCTGGGGCGGCGGGACGCCGAGTACTGGAACAGCCTTAAGGACTACATGGAGCAGAAGCGGGCCGCGGTGGACACGGTG

>AY699731_Apodemus_sylvaticus_MHC_class_II_antigen_(Apsy-DRB)_gene_Apsy-DRB*13_allele_exon_2_and_partial_cds

CAGCGCGTGCAGCTTCTGGTCAGACACATCTACAACCGGGAGGAGTTCGTGCGCTTCGACAGCGACGTGGGCGAGTACCGCGCGGTGACCGAGCTGGGGCGGGGCATAGCCGAGTACTACAACAGCCAGAAGGACTACATGGAGCGGAAGCGGGCCGAGGTGGACACGTAC

>AY699732_Apodemus_sylvaticus_MHC_class_II_antigen_(Apsy-DRB)_gene_Apsy-DRB*14_allele_exon_2_and_partial_cds

CAGCGCGTGCACTATCTGGAGAGACACATCTACAACCAGGAGGAGCACATGCGCTTCGACAGCGACGTGGGCGAGTACCGCGCGGTGACCGAGCTGGGGCGGGGCATAGCCGAGTACTTCAACAGCCAGAAGGACTACATGGAGCGGAAGCGGGCCGCGGTGGACACGTAC

>AY699733_Apodemus_sylvaticus_MHC_class_II_antigen_(Apsy-DRB)_gene_Apsy-DRB*15_allele_exon_2_and_partial_cds

CAGCGCGTGCAGTTTCTGGTCAGACACATCTACAACCGGGAGGAGTTCGTGCGCTTCGACAGCGACGTGGGCGAGTACCGCGCGGTGACCGAGCTGGGGCGGGGCATAGCCGAGTACTACAACAGCCAGAAGGACTACATGGAGCGGAAGCGGGCCGAGGTGGACACGTAC

>AY699734_Apodemus_sylvaticus_MHC_class_II_antigen_(Apsy-DRB)_gene_Apsy-DRB*16_allele_exon_2_and_partial_cds

CGGCACGTGCAGTTTCTGCAGAGATACATCTACAACCAGGAGGAGTTCGTGCGCTTCGACAGCGACGTGGGCGAGTTCCGCGCGGTGACCGAGCTGGGGCGGCCGGACGCCGAGTACCTCAACAGCCTGAAGGACTACGTGGAGCAGCTGCGGGCCGTGGTGGACACGGTG

>AY699735_Apodemus_sylvaticus_MHC_class_II_antigen_(Apsy-DRB)_gene_Apsy-DRB*17_allele_exon_2_and_partial_cds

CAGCGCGTGAAGTTTCTGGTCAGAGACATCTACAACCGGGAGGAGAACGTGCGCTTCGACAGCGACGTGGGCGAGTTCCGCGCATTGACCGAGCTGGGGCGGGGCATGGCCGAGTACCTCAACAGCCAGAAGGACTTCATGGAGCAGACGCGGGCCGCGGTGGACACGGTG

>AY699736_Apodemus_sylvaticus_MHC_class_II_antigen_(Apsy-DRB)_gene_Apsy-DRB*18_allele_exon_2_and_partial_cds

CGGCACGTGCAGCTTCTGCAGAGATACATCTACAACCAGGAGGAGTTCGTGCGCTTCGACAGCGACGTGGGCGAGTTCCGCGCGGTGACCGAGCTGGGGCGGCCGGACGCCGAGTACTTCAACAGCCTGAAGGACTACGTGGAGCAGCTGCGGGCCGTGGTGNACACGGTG

>AY699737_Apodemus_sylvaticus_MHC_class_II_antigen_(Apsy-DRB)_gene_Apsy-DRB*19_allele_exon_2_and_partial_cds

CAGCGCGTGCACTATCTGGTCAGACACTTCTACAACCAGGAGGAGTTCCTGTGCTTCGACAGCGACGTGGGCGAGTACCGCGCGGTGACCGAGCTGGGGCGGGGCATAGCCGAGTACTACAACAGCCAGAAGGAGCTCCTGGAGCGGAGGCGGGCCGAGGTGGACACGGTG

>AY699738_Apodemus_sylvaticus_MHC_class_II_antigen_(Apsy-DRB)_gene_Apsy-DRB*20_allele_exon_2_and_partial_cds

CAGCGCGTGCAGTTTCTGGAGAGACACTTCTACAACCGGGAGGAGTTCGTGCGCTTCGACAGCGACGTGGGCGAGTTCCGCGCGGTGACCGAGCTGGGGCGGCCGGACGCCGAGTACTGGAACAGCCTGAAGGACTACGTGGAGCAGAAGCGGGCCCAGGTGGACACGTAC

>AY699739_Apodemus_sylvaticus_MHC_class_II_antigen_(Apsy-DRB)_gene_Apsy-DRB*21_allele_exon_2_and_partial_cds

CAGCGCGTGCAGCTTCTGGCCAGATTAATCTACAACCAGGAGGAGTACGTGCGCTTCGACAGCGACGTGGGCGAGTTCCGCGCGGTGACCGAGCTGGGGCGGCGCTCAGCCGAGTACTGGAACAGCCAGAAGGACTTCATGGAGCAGCTGCGGGCCGCGGTGGACACGGTG

>AY699740_Apodemus_sylvaticus_MHC_class_II_antigen_(Apsy-DRB)_gene_Apsy-DRB*22_allele_exon_2_and_partial_cds

CAGCGCGTGCAGCTTCTGGAGAGATACATCCACAACCAGGAGGAGAACGTGCGCTTCGACAGCGACGTGGGCGAGTTCCGCGCGGTGACCGAGCTGGGGCGGGGCATAGCCGAGTACTACAACAGCCAGAAGGACTTCATGGAGCAGAAGCGGGCCGAGGTGGACACGGTG

>AY699741_Apodemus_sylvaticus_MHC_class_II_antigen_(Apsy-DRB)_gene_Apsy-DRB*23_allele_exon_2_and_partial_cds

CAGCGCGTGCAGTTTCTGGAGAGATACATCCACAACCAGGAGGAGAACGTGCGCTTCGACAGCGACGTGGGCGAGTTCCGCGCGGTGACCGAGCTGGGGCGGCCGGACGCCGAGTACTGGAACAGCCAGAAGGACTTCATGGAGCAGAAGCGGGCCGAGGTGGACACGGTG

>AY699742_Apodemus_sylvaticus_MHC_class_II_antigen_(Apsy-DRB)_gene_Apsy-DRB*24_allele_exon_2_and_partial_cds

CAGCGCGTGCAGCTTCTGGACAGATTCATCCACAACCAGGAGGAGTTCGTGCGCTTCGACAGCGACGTGGGCGAGTTCCGCGCGGTGACCGAGCTGGGGCGGGGCATAGCCGAGTACTNCAACAGCCAGAAGGACTTCATGGAGCAGAAGCGGGCCGAGGTGGACACGGTG

>AY699743_Apodemus_sylvaticus_MHC_class_II_antigen_(Apsy-DRB)_gene_Apsy-DRB*25_allele_exon_2_and_partial_cds

CAGCGCGTGCAGCTTCTGGTCAGACACATCTACAACCGGGAGGAGATCGTGCGCTTCGACAGCGACGTGGGCGAGTACCGCGCGGTGACCGAGCTGGGGCGGGGCATAGCCGAGTACTACAACAGCCAGAAGGACTACATGGAGCAGAAGCGGGCCGCGGTGGACACGTAC

>AY699744_Apodemus_sylvaticus_MHC_class_II_antigen_(Apsy-DRB)_gene_Apsy-DRB*26_allele_exon_2_and_partial_cds

CGGCACGTGCAGCTTCTGCAGAGATACATCTACAACCGGGAGGAGTACGCGCGCTTCGACAGCGACGTGGGCGAGTTCCGCGCGGTGACCGAGCTGGGGCGGGGCATAGCCGAGTACTACAACAGCCTGAAGGACTACGTGGAGCGGCTGCGGGCCGCGGTGGACACGTAC

>AY699745_Apodemus_sylvaticus_MHC_class_II_antigen_(Apsy-DRB)_gene_Apsy-DRB*27_allele_exon_2_and_partial_cds

CAGCGCGTGCACTATCTGGTCAGATACATCTACAACCAGGAGGAGCACATGCGCTTCGACAGCGACGTGGGCGAGTTCCGCGCGGTGACCGAGCTGGGGCGGGGCATAGCCGAGGACTTCAACAGCCGGAAGGAGATCCTGGAGCAGAAGCGGGCCGAGGTGGACACGGTG

>AY699746_Apodemus_sylvaticus_MHC_class_II_antigen_(Apsy-DRB)_gene_Apsy-DRB*28_allele_exon_2_and_partial_cds

CAGCGCGTGCACTATCTGGTCAGACACTTCTACAACCAGGAGGAGTTCGTGCGCTTCGACAGCGACGTGGGCGAGTACCGCGCGGTGACCGAGCTGGGGCGGGGCATAGCCGAGGACTGGAACAGCCAGAAGGACTTCATGGAGCGGAAGCGGGCCGAGGTGGACACGGTA

>AY699747_Apodemus_sylvaticus_MHC_class_II_antigen_(Apsy-DRB)_gene_Apsy-DRB*29_allele_exon_2_and_partial_cds

CAGGGCGTGCAGCTTCTGGCCAGATACATCTACAACCGGGAGGAGTTCGTGCGCTTCGACAGCGACGTGGGCGAGTTCCGCGCGGTGACCGAGCTGGGGCGGGGCATAGCCGAGTACTGGAACAGCCAGAAGGAGCTCCTGGAGCAGAAGCGGGCCGCGGTGGACACGCTC

>AY699748_Apodemus_sylvaticus_MHC_class_II_antigen_(Apsy-DRB)_gene_Apsy-DRB*30_allele_exon_2_and_partial_cds

CAGCGCGTGCACTATCTGGAGAGACGTATCCACAACCAGGAGGAGTACGCGCGCTACGACAGCGACGTGGGCGAGTACCGCGCGGTGACCGAGCTGGGGCGGCCGGACGCCGAGTACTGGAACAGCCGGAAGGAGATCCTGGAGGATGCGCGGGCCGCGGTGGACACGTAC

>AY699749_Apodemus_sylvaticus_MHC_class_II_antigen_(Apsy-DRB)_gene_Apsy-DRB*31_allele_exon_2_and_partial_cds

CAGCGCGTGCACTATCTGCAGAGATACTTCTACAACCAGGAGGAGTACGTGCGCTTCGACAGCGACGTGGGCGAGTTCCGCGCGGTGACCGAGCTGGGGCGGGGCATAGCCGAGTACTACAACAGCCAGAAGGAGATCCTGGAGCAGAAGCGGGCCGCGGTGGACACGTAC

>AY699750_Apodemus_sylvaticus_MHC_class_II_antigen_(Apsy-DRB)_gene_Apsy-DRB*32_allele_exon_2_and_partial_cds

CAGCGCGTGCACTATCTGGTCAGATTCATCTACAACCAGGAGGAGTACGTGCGCTTCGACAGCGACGTGGGCGAGTTCCGCGCGGTGACCGAGCTGGGGCGGCGCTCAGCCGAGTACTTCAACAGCCAGAAGGACTTCATGGAGCGGACGCGGGCCGTGGTGGACACGGGG

>AY699751_Apodemus_sylvaticus_MHC_class_II_antigen_(Apsy-DRB)_gene_Apsy-DRB*33_allele_exon_2_and_partial_cds

CAGCGCGTGCACTATCTGGTAAGACACTTCTACAACCAGGAGGAGTTCCTGTGCTTCGACAGCGACGTGGGCGAGTACCGCGCGGTGACCGAGCTGGGGCGGCCGGACGCCGAGTACTGGAACAGCCGGAAGGAGATCCTGGAGGATGCGCGGGCCGCGGTGGACACGTAC

>AY699752_Apodemus_sylvaticus_MHC_class_II_antigen_(Apsy-DRB)_gene_Apsy-DRB*34_allele_exon_2_and_partial_cds

CAGCGCGTGCACTATCTGGTCAGATTCATCTACAACCAGGAGGAGTTCGTGCGCTTCGACAGCGACGTGGGCGAGTACCGCGCGGTGACCGAGCTGGGGCGGCCGGACGCCGAGTACTGGAACAGCCAGAAGGACTTCATGGAGCGGACGCGGGCCGTGGTGGACACGGGG

>AY699753_Apodemus_sylvaticus_MHC_class_II_antigen_(Apsy-DRB)_gene_Apsy-DRB*35_allele_exon_2_and_partial_cds

CAGGGCGTGCGGTTTCTGCTCAGATGCATCTACAACCGGGAGGAGTACGTGCGCTTCGACAGCGACGTGGGCGAGTTCCGCGCGGTGACCGAGCTGGGGCGGCGCTCAGCCGAGTACTACAACAGCCAGAAGGACTACATGGAGCAGATGCGGGCCGTGGTGGACACGGGG

>AY699754_Apodemus_sylvaticus_MHC_class_II_antigen_(Apsy-DRB)_gene_Apsy-DRB*36_allele_exon_2_and_partial_cds

CAGCGCGTGCAGCTTCTGCAGAGATACATCTACAACCGGGAGGAGATCGTGCGCTTCGACAGCGACGTGGGCGAGTTCCGCGCGGTGACCGAGCTGGGGCGGTCGTGGGCCGAGGACTTCAACAGCCGGAAGGACTTCCTGGAGCAGCTGCGGGCCGCGGTGGACACGTAC

>AY699755_Apodemus_sylvaticus_MHC_class_II_antigen_(Apsy-DRB)_gene_Apsy-DRB*37_allele_exon_2_and_partial_cds

CAGCGCGTGCACTATCTGGTCAGACACTTCTACAACCAGGAGGAGTTCGTGCGCTTCGACAGCGACGTGGGCGAGTACCGCGCGGTGACCGAGCTGGGGCGGCCGGACGCCGAGTACTGGAACAGCCAGAAGGACTTCATGGAGCGGAGGCGGGCCGAGGTGGACACGGTG

>AY699756_Apodemus_sylvaticus_MHC_class_II_antigen_(Apsy-DRB)_gene_Apsy-DRB*38_allele_exon_2_and_partial_cds

CAGCGCGTGCACTATCTGGTCAGATTAATCTACAACCAGGAGGAGTTCGTGCGCTTCGACAGCGACGTGGGCGAGTACCGCGCGGTGACCGAGCTGGGGCGGGGCATAGCCGAGTACTTCAACAGCCTGAAGGACTACGTGGAGCGGACGCGGGCCGAGGTGGACACGGTG

>AY699757_Apodemus_flavicollis_MHC_class_II_antigen_(Apfl-DRB)_gene_Apfl-DRB*01_allele_exon_2_and_partial_cds

CAGCGCGTGCAGCTTCTGGTCAGATACATCCACAACCAGGAGGAGAACGTGCGCTTCGACAGCGACGTGGGCGAGTTCCGCGCGGTGACCGAGCTGGGGCGGCCGGACGCCGAGTACTGGAACAGCCAGAAGGAGATCCTGGAGCAGAAGCGGGCCGAGGTGGACACGGTG

>AY699758_Apodemus_flavicollis_MHC_class_II_antigen_(Apfl-DRB)_gene_Apfl-DRB*02_allele_exon_2_and_partial_cds

CAGCGCGTGCAGCTTCTGGTCAGATACATCTACAACCGGGAGGAGTACGCGCGCTTCGACAGCGACGTGGGCGAGTTCCGCGCGGTGACCGAGCTGGGGCGGCGGGACGCCGAGTACTGGAACAGCCAGAAGGAGATCCTGGAGCAGAAGCGGGCCGAGGTGGACACGGTG

>AY699759_Apodemus_flavicollis_MHC_class_II_antigen_(Apfl-DRB)_gene_Apfl-DRB*03_allele_exon_2_and_partial_cds

CAGCGCGTGCACTATCTGGTCAGATACTTCTACAACCGGGAGGAGTACGCGCGCTTCGACAGCGACGTGGGCGAGTACCGCGCGGTGACCGAGCTGGGGCGGCCGGACGCCGAGTACTGGAACAGCCAGAAGGAGATCCTGGAGCGGAAGCGGGCCGAGGTGGACACGGTG

>AY699760_Apodemus_flavicollis_MHC_class_II_antigen_(Apfl-DRB)_gene_Apfl-DRB*04_allele_exon_2_and_partial_cds

CAGCACGTGCAGCTTCTGGTCAGATACATCTACAACCGGGAGGAGTACGTGCGCTTCGACAGCGACGTGGGCGAGTTCCGCGCGGTGACCGAGCTGGGGCGGCGGGACGCCGAGTACTACAACAGCCAGAAGGACTACATGGAGCAGACGCGGGCCGAGGTGGACACGGTG

>AY699761_Apodemus_flavicollis_MHC_class_II_antigen_(Apfl-DRB)_gene_Apfl-DRB*05_allele_exon_2_and_partial_cds

CAGCGCGTGCACTATCTGGAGAGACACATCTACAACCAGGAGGAGCACATGCGCTTCGACAGCGACGTGGGCGAGTACCGCGCGGTGACCGAGCTGGGGCGGGGCATAGCCGAGTACTACAACAGCCAGAAGGACTACATGGAGCGGAAGCGGGCCGAGGTGGACACGGTG

>AY699762_Apodemus_flavicollis_MHC_class_II_antigen_(Apfl-DRB)_gene_Apfl-DRB*06_allele_exon_2_and_partial_cds

CAGCGCGTGCACTATCTGGTCAGATTCTTCTACAACCGGGAGGAGTACGCGCGCTTCGACAGCGACGTGGGCGAGTTCCGCGCGGTGACCGAGCTGGGGCGGCGCTCAGCCGAGTACTGGAACAGCCAGAAGGAGATCCTGGAGCGGAAGCGGGCCGAGGTGGACACGGTG

>AY699763_Apodemus_flavicollis_MHC_class_II_antigen_(Apfl-DRB)_gene_Apfl-DRB*07_allele_exon_2_and_partial_cds

CAGCGCGTGCACTATCTGGTCAGATACATCTACAACCAGGAGGAGCACATGCGCTTCGACAGCGACGTGGGCGAGTTCCGCGCGGTGACCGAGCTGGGGCGGGGCATAGCCGAGGACTTCAACAGCCGGAAGGAGATCCTGGAGCAGAAGCGGGCCGAGGTGGACACGGTG

>AY699764_Apodemus_flavicollis_MHC_class_II_antigen_(Apfl-DRB)_gene_Apfl-DRB*08_allele_exon_2_and_partial_cds

CAGCGCGTGCACTATCTGGTCAGATACATCTACAACCGGGAGGAGTTCGTGCGCTTCGACAGCGACGTGGGCGAGTACCGCGCGGTGACCGAGCTGGGGCGGCCGGACGCCGAGTACTTCAACAGCCAGAAGGAGATCCTGGAGCGGAAGCGGGCCGAGGTGGACACGGTG

>AY699765_Apodemus_flavicollis_MHC_class_II_antigen_(Apfl-DRB)_gene_Apfl-DRB*09_allele_exon_2_and_partial_cds

CAGCACGTGCAGCTTCTGCAGAGATACATCTACAACCGGGAGGAGTACGCGCGCTTCGACAGCGACGTGGGCGAGTTCCGCGCGGTGACCGAGCTGGGGCGGCCGGACGCCGAGTACTACAACAGCCAGAAGGACTACATGGAGCAGCTGCGGGCCGCGGTGGACACGTAC

>AY699766_Apodemus_flavicollis_MHC_class_II_antigen_(Apfl-DRB)_gene_Apfl-DRB*10_allele_exon_2_and_partial_cds

CAGCGCGTGCACTATCTGGTCAGATACTTCTACAACCGGGAGGAGTACGTGCGCTTCGACAGCGACGTGGGCGAGTACCGCGCGGTGACCGAGCTGGGGCGGCCGGACGCCGAGTACTGGAACAGCCAGAAGGAGATCCTGGAGCGGAAGCGGGCCGAGGTGGACACGGTG

>AY699767_Apodemus_flavicollis_MHC_class_II_antigen_(Apfl-DRB)_gene_Apfl-DRB*11_allele_exon_2_and_partial_cds

CAGCGCGTGCACTATCTGGTCAGATTCTTCTACAACCGGGAGGAGTACGCGCGCTTCGACAGCGACGTGGGCGAGTTCCGCGCGGTGACCGAGCTGGGGCGGCCGGACGCCGAGTACTGGAACAGCCAGAAGGAGATCCTGGAGCGGAAGCGGGCCGAGGTGGACACGGTG

>AY699768_Apodemus_flavicollis_MHC_class_II_antigen_(Apfl-DRB)_gene_Apfl-DRB*12_allele_exon_2_and_partial_cds

CAGCGCGTGCAGCTTCTGGTCAGATACATCTACAACCGGGAGGAGTTCGTGCGCTTCGACAGCGACGTGGGCGAGTTCCGCGCGGTGACCGAGCTGGGGCGGGGCATAGCCGAGTACTGGAACAGCCAGAAGGAGATCCTGGAGCAGAAGCGGGCCGAGGTGGACACGGCG

>AY699769_Apodemus_flavicollis_MHC_class_II_antigen_(Apfl-DRB)_gene_Apfl-DRB*13_allele_exon_2_and_partial_cds

CAGCGCGTGCAGCTTCTGCAGAGATACATCTACAACCGGGAGGAGATCGTGCGCTTCGACAGCGACGTGGGCGAGTTCCGCGCGGTGACCGAGCTGGGGCGGTCGTGGGCCGAGGACTTCAACAGCCGGAAGGAGATCCTGGAGCAGCTGCGGGCCGCGGTGGACACGTAC

>AY699770_Apodemus_flavicollis_MHC_class_II_antigen_(Apfl-DRB)_gene_Apfl-DRB*14_allele_exon_2_and_partial_cds

CAGCACGTGCAGCTTCTGGTCAGATTCTTCTACAACCGGGAGGAGTACGCGCGCTTCGACAGCGACGTGGGCGAGTTCCGCGCGGTGACCGAGCTGGGGCGGCGCTCAGCCGAGTACTACAACAGCCGGAAGGAGATCCTGGAGCAGCTGCGGGCCGAGGTGGACACGGTG

>AY699771_Apodemus_flavicollis_MHC_class_II_antigen_(Apfl-DRB)_gene_Apfl-DRB*15_allele_exon_2_and_partial_cds

CAGCGCGTGCACTATCTGGAGAGACACATCTACAACCAGGAGGAGCACATGCGCTTCGACAGCGACGTGGGCGAGTACCGCGCGGTGACCGAGCTGGGGCGGGGCATAGCCGAGTACTACAACAGCCAGAAGGACTACATGGAGCAGAAGCGGGCCGAGGTGGACACGGTG

>AY918078_Apodemus_flavicollis_MHC_class_II_antigen_(Apfl-DRB)_gene_Apfl-DRB*16_allele_exon_2_and_partial_cds

CAGCGCGTGCAGCTTCTGGTCAGATTCTTCTACAACCGGGAGGAGTTCGTGCGCTTCGACAGCGACGTGGGCGAGTACCGCGCGGTGACCGAGCTGGGGCGGGGCATAGCCGAGTACTACAACAGCCAGAAGGACTACATGGAGCGGAAGCGGGCCGAGGTGGACACGTAC

>AY918079_Apodemus_flavicollis_MHC_class_II_antigen_(Apfl-DRB)_gene_Apfl-DRB*17_allele_exon_2_and_partial_cds

CAGCGCGTGCAGCTTCTGCAGAGATACATCTACAACCGGGAGGAGTACGTGCGCTTCGACAGCGACGTGGGCGAGTTCCGCGCGGTGACCGAGCTGGGGCGGGGCATAGCCGAGTACTTCAACAGCCAGAAGGACTTCTTGGAGCAGCTGCGGGCCGCGGTGGACACGTAC

>AY918081_Apodemus_flavicollis_MHC_class_II_antigen_(Apfl-DRB)_gene_Apfl-DRB*19_allele_exon_2_and_partial_cds

CAGCGCGTGCAGTTTCTGCAGAGATACATCTACAACCGGGAGGAGTACGTGCGCTTCGACAGCGACGTGGGCGAGTTCCGCGCGGTGACCGAGCTGGGGCGGCGCTCAGCCGAGTACTGGAACAGCCAGAAGGAGATCCTGGAGGATGCGCGGGCCGCGGTGGACACGTAC

>AY918082_Apodemus_flavicollis_MHC_class_II_antigen_(Apfl-DRB)_gene_Apfl-DRB*20_allele_exon_2_and_partial_cds

CAGCGCGTGCACTATCTGGACAGATACATCTACAACCAGGAGGAGTACGCGCGCTTCGACAGCGACGTGGGCGAGTTCCGCGCGGTGACCGAGCTGGGGCGGCGCTCAGCCGAGTACTACAACAGCCAGAAGGACTTCCTGGAGCAGAAGCGGGCCGAGGTGGACACGTAC

>AY918083_Apodemus_flavicollis_MHC_class_II_antigen_(Apfl-DRB)_gene_Apfl-DRB*21_allele_exon_2_and_partial_cds

CAGCGCGTGCACTATCTGGTCAGATACATCCACAACCAGGAGGAGAACGTGCGCTTCGACAGCGACATGGGCGAGTTCCGCGCGGTGACCGAGCTGGGGCGGCCGGACGCCGAGTACTGGAACAGCCGGAAGGAGATCCTGGAGCGGAAGCGGGCCGAGGTGGACACGGTG

>AY918085_Apodemus_flavicollis_MHC_class_II_antigen_(Apfl-DRB)_gene_Apfl-DRB*23a_allele_exon_2_and_partial_cds

CAGCGCGTGCACTATCTGGACAGATACTTCTACAATCGGGAGGAGTACGTGCGCTTCGACAGCGACGTGGGCGAGTTCCGCGCGGTGACCGAGCTGGGGCGGCGGGACGCCGAGTACTGGAACAGCCAGAAGGACTTCATGGAGCAGAAGCGGGCCGAGGTGGACACGTAC

>AY918086_Apodemus_flavicollis_MHC_class_II_antigen_(Apfl-DRB)_gene_Apfl-DRB*23b_allele_exon_2_and_partial_cds

CAGCGCGTGCACTATCTGGACAGATACTTCTACAACCGGGAGGAGTACGTGCGCTTCGACAGCGACGTGGGCGAGTTCCGCGCGGTGACCGAGCTGGGGCGGCGGGACGCCGAGTACTGGAACAGCCAGAAGGACTTCATGGAGCAGAAGCGGGCCGAGGTGGACACGTAC

>AY918087_Apodemus_flavicollis_MHC_class_II_antigen_(Apfl-DRB)_gene_Apfl-DRB*24_allele_exon_2_and_partial_cds

CAGCACGTGCAGCTTCTACAGAGATACATCTACAACCGGGAGGAGTACGTGCGCTTCGACAGCGACGTGGGCGAGTTCCGCGCGGTGACCGAGCTGGGGCGGCCGGACGCCGAGTACTACAACAGCCAGAAGGACTACATGGAGCAGCTGCGGGCCGCGGTGGACACGTAC

>AY918088_Apodemus_flavicollis_MHC_class_II_antigen_(Apfl-DRB)_gene_Apfl-DRB*25_allele_exon_2_and_partial_cds

CAGCGCGTGCACTATCTGGTCAGATACATCTACAACCAGGAGGAGTACGTGCGCTTCGACAGCGACGTGGGCGAGTTCCGCGCGGTGACCGAGCTGGGGCGGGGCATAGCCGAGGACTTCAACAGCCGGAAGGAGATCCTGGAGCAGAAGCGGGCCGAGGTGGACACGGTG

>AY918089_Apodemus_flavicollis_MHC_class_II_antigen_(Apfl-DRB)_gene_Apfl-DRB*26_allele_exon_2_and_partial_cds

CAGCGCGTGCAGCTTCTGGTCAGATTCTTCTACAACCGGGAGGAGTACGCGCGCTTCGACAGCGACGTGGGCGAGTTCCGCGCGGTGACCGAGCTGGGGCGGCGCTCAGCCGAGTACTACAACAGCCGGAAGGAGATCCTGGAGCAGCTGCGGGCCGAGGTGGACACGGTG

>AY918090_Apodemus_flavicollis_MHC_class_II_antigen_(Apfl-DRB)_gene_Apfl-DRB*27_allele_exon_2_and_partial_cds

CAGCGCGTGCAGTTTCTGGACAGATACATCCACAATCAGGAGGAGTACGTGCGCTTCGACAGCGACGTGGGCGAGTACCGCGCGGTGACCGAGCTGGGGCGGCCGGACGCCGAGTACTACAACAGCCAGAAGGAGATCCTGGAGGATGCGCGGGCCGCGGTGGACACGTAC

>AY928312_Rhabdomys_pumilio_MHC_class_II_antigen_(Rhpu-DRB)_gene_Rhpu-DRB*1_allele_exon_2_and_partial_cds

CAGCGCGTGCGGTTTCTGGACAGATACTTCTACAACCGGGAGGAGTACCTGCGCTTCGACAGCGACGTGGGCGAGTACCGCGCGGTGACCGAGCTGGGGCGGCGCACAGCCAAGTACTGGAACAGCCAGAAGGAGCTCCTGGAGCATAAGCGGGCCCAGGTGGACGCGTAC

>AY928313_Rhabdomys_pumilio_MHC_class_II_antigen_(Rhpu-DRB)_gene_Rhpu-DRB*2_allele_exon_2_and_partial_cds

CAGCGCGTGCGGTTTCTGGACAGATACTTCTACAACCGGGAGGAGTACGCGCGCTTCGACAGCGACGTGGGCGAGTTCCGCGCGGTGACCGAGCTGGGGCGGCCGGACGCTGAGTACTGGAACAGCCAGAAGGAGCTCCTGGAGGATCGGCGGGCCCAGGTGGACGCGTAC

>AY928314_Rhabdomys_pumilio_MHC_class_II_antigen_(Rhpu-DRB)_gene_Rhpu-DRB*3_allele_exon_2_and_partial_cds

CAGCGCGTGCGGTTTCTGGACACATACTTCTACAACCGGGAGGAGTACCTGCGCTTCGACAGCGACGTGGGCGAGTACCGCGCGGTGACCGAGCTGGGGCGGCCGGACGCTGATTACTGGAACAGCCAGAAGGAGCTCCTGGAGCATAGGCGGGCCCAGGTGGACACGTAC

>AY928315_Rhabdomys_pumilio_MHC_class_II_antigen_(Rhpu-DRB)_gene_Rhpu-DRB*4_allele_exon_2_and_partial_cds

CAGCGCGTGCGGTTTCTGGACAGATACTTCTACAACCGGGAGGAGTACGTGCGCTTCGACAGCGACGTGGGCGAGCACCGGGCGGTGACCGAGCTGGGGCGGCCGGACGCTGATTACTGGAACAGCCAGAAGGAGCTCCTGGAGGATCGGCGGGCCCAGGTGGACACGTAC

>AY928316_Rhabdomys_pumilio_MHC_class_II_antigen_(Rhpu-DRB)_gene_Rhpu-DRB*5_allele_exon_2_and_partial_cds

CAGCACGTGAGGTTTCTGGAGAGACACATCTACAACCGGGAGGAGTTCATGCGCTTCGACAGCGACGTGGGCGAGTACCGCACTGTGACCGAGCTGGGGCGGCGCATAGCTGAGGACTTGAACAGCCAGAAGGAGCTCCCGGAGCAGAAGCGGGCCCAGGTGGACGCGTAC

>AY928317_Rhabdomys_pumilio_MHC_class_II_antigen_(Rhpu-DRB)_gene_Rhpu-DRB*6_allele_exon_2_and_partial_cds

CAGCACGTGAGGTTTCTGGACAGATACTTCTACAACCGGGAGGAGTACGTGCGCTTCGACAGCGACGTGGGCGAGTTCCGCGCGGTGACCGAGCTGGGGCGGCCGGACGCTGAGTACTGGAACAGCCAGAAGGAGTTCCTGGAGGATCGGCGGGCCCAGGTGGACACAGTG

>AY928318_Rhabdomys_pumilio_MHC_class_II_antigen_(Rhpu-DRB)_gene_Rhpu-DRB*7_allele_exon_2_and_partial_cds

CAGCACGTGCGGTTTCTGGAGAGACACATCTACAACCGGGAGGAGTTCGTGCGCTTCGACAGCGACGTGGGCGAGCACCGCGCGGTGACCGAGCTGGGGCGGCGCATAGCTGAGGACTGGAACAGCCAGAAGGAGCTCCTGGAGCGGAAGCGGGCCGAGCTGGACACGTAC

>AY928319_Rhabdomys_pumilio_MHC_class_II_antigen_(Rhpu-DRB)_gene_Rhpu-DRB*8_allele_exon_2_and_partial_cds

CAGCGCGTGCGGTTTCTGGACAGATACTTCTACAACCGGGAGGAGTACGTGCGCTTCGACAGCGACGTGGGCGAGCACCGCGCGGTGACCGAGCTGGGGCGGCCGGACGCTGAGTACTGGAACAGCCAGAAGGAGCTCCTGGAGGATCGGCGGGCCCAGGTGGACACGTAC

>AY928320_Rhabdomys_pumilio_MHC_class_II_antigen_(Rhpu-DRB)_gene_Rhpu-DRB*9_allele_exon_2_and_partial_cds

CAGCGCGTGCGGTTTCTGGAGAGACACATCTACAACCGGGAGGAGTTCGTGCGCTTCGACAGCGACGTGGGCGAGTACCGCGCGGTGACCGAGCTGGGGCGGGGCATAGCTGAGGACTGGAACAGCCAGAAGGAGTTCCTGGAGCAGAGGCGGGCCGCGGTGGACACGTAC

>AY928321_Rhabdomys_pumilio_MHC_class_II_antigen_(Rhpu-DRB)_gene_Rhpu-DRB*10_allele_exon_2_and_partial_cds

CAGCGCGTGCGGTTTCTGGAGAGATACTTCTACAACCGGGAGGAGTACGCGCGCTTCGACAGCGACGTGGGCGAGTACCGCGCGGTGACCGAGCTGGGGCGGCGCACAGCCAAGTACTGGAACAGCCAGAAGGAGATCCTGGAGCGGAAGCGGGCCCAGGTGGACGCGTAT

>AY928322_Rhabdomys_pumilio_MHC_class_II_antigen_(Rhpu-DRB)_gene_Rhpu-DRB*11_allele_exon_2_and_partial_cds

CAGCACGTGCGGTTTCTGGACAGATACTTCTACAACCGGGAGGAGTACGCGCGCTTCGACAGCGACGTGGGCGAGTTCCGCGCGGTGACCGAGCTGGGGCGGCCGGACGCCGAGTACTGGAACAGCCAGAAGGAGATCCTGGAGCGGAAGCGGGCCGAGGTGGACACGGTG

>AY928323_Rhabdomys_pumilio_MHC_class_II_antigen_(Rhpu-DRB)_gene_Rhpu-DRB*12_allele_exon_2_and_partial_cds

CAGCGCGTGCGGTTTCTGGACAGATACTTCTACAACCGGGAGGAGTACGTGCGCTTCGACAGCGACGTGGGCGAGTACCGCGCGGTGACCGAGCTGGGGCGGCGGGACGCTGATTACTGGAACAGCCAGAAGGAGCTCCTGGAGCAGAGGCGGGCCCAGGTGGACACGTAC

>AY928324_Rhabdomys_pumilio_MHC_class_II_antigen_(Rhpu-DRB)_gene_Rhpu-DRB*13_allele_exon_2_and_partial_cds

CAGCGCGTGCGGTTTCTGGACAGATACTTCTACAACCGGGAGGAGTACGTGCGCTTCGACAGCGACGTGGGCGAGTACCGCGCGGTGACCGAGCTGGGGCGGCGCACAGCCAAGTACTGGAACAGCCAGAAGGAGCTCCTGGAGCAGAAGCGGGCCCAGGTGGACGCGTAC

>AY928325_Rhabdomys_pumilio_MHC_class_II_antigen_(Rhpu-DRB)_gene_Rhpu-DRB*14_allele_exon_2_and_partial_cds

CAGCGCGTGCGGTTTCTGGAGAGACACATCTACAACCGGGAGGAGTTCGTGCGCTTCGACAGCGACGTGGGCGAGTACCGCGCGGTGACCGAGCTGGGGCGGGGCATAGCTGAGGACTGGAACAGCCAGAAGGAGTACCTGGAGGATGCGCGGGCCGCGGTGGACACGTAC

>AY928326_Rhabdomys_pumilio_MHC_class_II_antigen_(Rhpu-DRB)_gene_Rhpu-DRB*15_allele_exon_2_and_partial_cds

CAGCACGTGCGGTTTCTGGACAGATACTTCTACAACCGGGAGGAGTACGCGCGCTTCGACAGCGACGTGGGCGAGTACCGCGCGGTGACCGAGCTGGGGCGGCGGGACGCCGAGTACTGGAACAGCCAGAAGGAGCTCCTGGAGCGGAAGCGGGCCCGGGTGGACACGTAC

>AY928327_Rhabdomys_pumilio_MHC_class_II_antigen_(Rhpu-DRB)_gene_Rhpu-DRB*16_allele_exon_2_and_partial_cds

CAGCGCGTGCGGTTTCTGGAGAGATACTTCTACAACCGGGAGGAGTACGCGCGCTTCGACAGCGACGTGGGCGAGTACCGCGCGGTGACCGAGCTGGGGCGGCGCACAGCCAAGTACTGGAACAGCCAGAAGGAGATCCTGGAGCGGAAGCGGGCCCAGGTGGACGCGTAC

>AY928328_Rhabdomys_pumilio_MHC_class_II_antigen_(Rhpu-DRB)_gene_Rhpu-DRB*17_allele_exon_2_and_partial_cds

CAGCGCGTGCGGTTTCTGGAGAGATACTTCTACAACCGGGAGGAGTACCTGCGCTTCGACAGCGACGTGGGCGAGTTCCGCGCGGTGACCGAGCTGGGGCGGCCGGACGCTGAGTACTGGAACAGCCAGAAGGAGCTCCTGGAGGATCGGCGGGCCCAGGTGGACGCGTAC

>AY928329_Rhabdomys_pumilio_MHC_class_II_antigen_(Rhpu-DRB)_gene_Rhpu-DRB*18_allele_exon_2_and_partial_cds

CAGCGCGTGCGGTTTCTGGACAGATACTTCTACAACCGGGAGGAGTACGCGCGCTTCGACAGCGACGTGGGCGAGTACCGCGCGGTGACCGAGCTGGGGCGGCGCACAGCCAAGTACTGGAACAGCCAGAAGGAGCTCCTGGAGCAGAAGCGGGCCCGGGTGGACACGTAC

>AY928330_Rhabdomys_pumilio_MHC_class_II_antigen_(Rhpu-DRB)_gene_Rhpu-DRB*19_allele_exon_2_and_partial_cds

CAGCGCGTGCGGTTTCTGGACAGATACTTCTACAACCGGGAGGAGTTCGTGCGCTTCGACAGCGACGTGGGCGAGTTCCGCGCGGTGACCGAGCTGGGGCGGCGCATAGCTGAGGACTTGAACAGCCAGAAGGAGCTCCTGGAGCAGAAGCGGGCCGCGGTGGACACGTAC

>AY928331_Rhabdomys_pumilio_MHC_class_II_antigen_(Rhpu-DRB)_gene_Rhpu-DRB*20_allele_exon_2_and_partial_cds

CAGCGCGTGCGGTTTCTGGACAGATACTTCTACAACCGGGAGGAGTACGTGCGCTTCGACAGCGACGTGGGCGAGTACCGCGCGGTGACCGAGCTGGGGCGGCGCACAGCCAATTACTGGAACAGCCAGAAGGAGCTCTTGAAGCAGAAGCGGGCCCAGGTGGACGCGTAC

>DQ060686_Castor_fiber_albicus_MHC_class_II_antigen_(Cafi-DRB)_gene_Cafi-DRB*01_allele_exon_2_and_partial_cds

GAGCGGGTGCGGTTTCTGAACAGATACGTCTACAACCGGGAGGAGTTCGTGCGCTTCGACAGCGACGTCGGGGAGTTCCGCGCGGTGACCGAGCTGGGGCGGCCCGACGCCGAGTACTGGAACGGCCAGAAGGACCTCCTGGAGCGGAAGCGGGCCGCGGTGGACACCGTG

>DQ060687_Castor_fiber_fiber_MHC_class_II_antigen_(Cafi-DRB)_gene_Cafi-DRB*02_allele_exon_2_and_partial_cds

GAGCGGGTGCGGCTTCTGGACAGATACTTCTACAACCGGGAGGAGTACGTGCGCTTCGACAGCGACGTCGGGGAGTTCCGCGCGGTGACCGAGCTGGGGCGGCCCGACGCCGAGTACTGGAACGGCCAGAAGGACGTCCTGGAGGACGCGCGGGCCGCGGTGGACACCGTG

>DQ060688_Castor_fiber_MHC_class_II_antigen_(Cafi-DRB)_gene_Cafi-DRB*03_allele_exon_2_and_partial_cds

GAGCGGGTGCGGTTTCTGGACAGATACTTCCATAACGGGGAGGAGAACGTGCGCTTCGACAGCGACGTCGGGGAGTTCCGCGCGGTGACCGAGCTGGGGCGGCCCGACGCCGAGTACTGGAACGGCCAGAAGGACGTCCTGGAGGACGCGCGGGCCGCGGTGGACACATTC

>DQ060689_Castor_fiber_birulai_MHC_class_II_antigen_(Cafi-DRB)_gene_Cafi-DRB*04_allele_exon_2_and_partial_cds

GAGCGGGTGCGGTTTCTGGACAGATACTTCTACAACCGGGAGGAGTACGTGCGCTTCGACAGCGACGTCGGGGAGTACCGCGCGGTGACCGAGCTGGGGCGGCGCTCAGCCGAGTCCTGGAACGGCCAGAAGGACATCCTGGAGGACGCGCGGGCCGCGGTGGACACATAC

>DQ060690_Castor_fiber_tuvinicus_MHC_class_II_antigen_(Cafi-DRB)_gene_Cafi-DRB*05_allele_exon_2_and_partial_cds

GAGCGGGTGCGGTTTCTGGAGAGACACATCTACAACCGGGAGGAGCACGTGCGCTTCGACAGCGACGTCGGGGAGTACCGCGCGGTGACCGAGCTGGGGCGGCCCATCGCCGAGTCCTGGAACGGCCAGAAGGACGTCCTGGAGGACGCGCGGGCCGCGGTGGACACATAC

>DQ060691_Castor_fiber_pohlei_MHC_class_II_antigen_(Cafi-DRB)_gene_Cafi-DRB*06_allele_exon_2_and_partial_cds

GAGCGGGTGCGGCTTCTGGACAGATACTTCTACAACCGGGAGGAGTACGTGCGCTTCGACAGCGACGTCGGGGAGTTCCGCGCGGTGACCGAGCTGGGGCGGCCCGACGCCGAGTACTGGAACGGCCAGAAGGACGTCCTGGAGGACGCGCGGGCCGCGGTGGACACATTC

>DQ060692_Castor_fiber_galliae_MHC_class_II_antigen_(Cafi-DRB)_gene_Cafi-DRB*07_allele_exon_2_and_partial_cds

GAGCGGGTGCGGCTTCTGGACAGATACTTCTACAACCGGGAGGAGCACGTGCGCTTCGACAGCGACGTCGGGGAGTTCCGCGCGGTGACCGAGCTGGGGCGGCCCGACGCCGAGTACTGGAACGGCCAGAAGGACGTCCTGGAGGACGCGCGGGCCGCGGTGGACACATTC

>DQ060693_Castor_fiber_pohlei_MHC_class_II_antigen_(Cafi-DRB)_gene_Cafi-DRB*08_allele_exon_2_and_partial_cds

GAGCGGGTGCGGTTTCTGGAGAGACACATCTACAACCGGGAGGAGCACGTGCGCTTCGACAGCGACGTCGGGGAGTACCGCGCGGTGACCGAGCTGGGGCGGCCCATCGCCGAGTCCTGGAACGGCCAGAAGGACCTCCTGGAGCAGAGGCGGGCCGCGGTGGACACATAC

>DQ060694_Castor_fiber_pohlei_MHC_class_II_antigen_(Cafi-DRB)_gene_Cafi-DRB*09_allele_exon_2_and_partial_cds

GAGCGGGTGCGGTTTCTGGAGAGACACATCTACAACCGGGAGGAGCACGTGCGCTTCGACAGCGACGTCGGGGAGTACCGCGCGGTGACCGAGCTGGGGCGGCCCGACGCCGAGTACTGGAACGGCCAGAAGGACCTCCTGGAGCAGAGGCGGGCCGCGGTGGACACATAC

>DQ060695_Castor_fiber_pohlei_MHC_class_II_antigen_(Cafi-DRB)_gene_Cafi-DRB*10_allele_exon_2_and_partial_cds

GAGCGGGTGCGGTTTCTGGAGAGACACATCTACAACCGGGAGGAGCACGTGCGCTTCGACAGCGACGTCGGGGAGTACCGCGCGGTGACCGAGCTGGGGCGGCCCGACGCCGAGTACTGGAACGGCCAGAAGGACGTCCTGGAGGACGCGCGGGCCGCGGTGGACACATTC

>DQ202212_Arvicola_terrestris_MHC_class_II_antigen_(Arte-DRB)_gene_Arte-DRB*01_allele_exon_2_and_partial_cds

CAGCGCGTGCGGTATCTAGAGAGACAGTTCTACAACCGGGAGGAGTACGTGCGCTTCGACAGCGACGTGGGCGAGTTCCGCGAGGTGACCGAGCAGGGGCGGGGCATAGCCGAGAACTTGAACAGCCAGAAGGAGCTCCTGGAGCGGAAGCGGGCCGAGAAAGACACGGTG

>DQ202213_Arvicola_terrestris_MHC_class_II_antigen_(Arte-DRB)_gene_Arte-DRB*02_allele_exon_2_and_partial_cds

CAGCGCGTGCGGCTTCTAGAGAGATACATCTACAACCGGGAGGAGTTCGTGCGCTTCGACAGCGACGTGGGCGAGCACCGCGCGGTGACCGAGCTGGGGCGGGGCATAGCCGAGAACTTCAACAGCCGGAAGGAGCTCCTGGAGAACAGGCGGGCCGCGGTGGACACGGTG

>DQ202214_Arvicola_terrestris_MHC_class_II_antigen_(Arte-DRB)_gene_Arte-DRB*03_allele_exon_2_and_partial_cds

CAGCGCGTGCGGTATCTGGCCAGAGTCATCTACAACCGGGAGGAGTACGTGCGCTTCGACAGCGACGTGGGCGAGTTCCGCGCGGTGACCGAGCTGGGGCGGCCGGACGCCGAGTACTGGAACAGCCAGAAGGACTTCATGGAGCAGAAGCGGGCCGCGGTGGACACGTAC

>DQ202215_Arvicola_terrestris_MHC_class_II_antigen_(Arte-DRB)_gene_Arte-DRB*04_allele_exon_2_and_partial_cds

CAGCGCGTGCGGCTTCTGGTCAGATACATCTACAACCGGGAGGAGTTCGTGCGCTTCGACAGCGACGTGGGCGAGTTCCGCGCGGTGACCGAGCTGGGGCGGCCGGACGCCAAGTACTTGAACAGCCGGAAGGACTTCATAGAGCAGTTGCGGGCCGCGGTGGACACGGTG

>DQ202216_Arvicola_terrestris_MHC_class_II_antigen_(Arte-DRB)_gene_Arte-DRB*05_allele_exon_2_and_partial_cds

CAGCGCGTGCGGTATCTGGCCAGAGTCATCTACAACCGGGAGGAGTACGCGCGCTTCGACAGCGACGTGGGCGAATTCCGCGCGGTGACCGAGCTGGGGCGGCGCTCAGCCGAGTACTGGAACAGCCAGAAGGAGCTCCTGGAGCAGAAGCGGGCCGCGGTGGACACGTAC

>DQ380431_Ctenomys_talarum_MHC_class_II_antigen_(Cta-DRB)_gene_Cta-DRB*0102_allele_exon_2_and_partial_cds

GAGCATGTTCAGATGGTGGTCAGACACATCTACAAGCGGGAGGAGTTCTTACGCTACGACAGTGACCTGGGCAAGTACCTGGCAGTGGCTGGGTTGGGGCGGCAGGAGGCAGAAGACTGGAACACCAGGAAAGACCTCCTGGAGCAGAGGCGTTCCCAGCTGGACACCTTG

>DQ380432_Ctenomys_talarum_MHC_class_II_antigen_(Cta-DRB)_gene_Cta-DRB*0103_allele_exon_2_and_partial_cds

GAGCATGTTCAGATGGTGGTCAGACACATCTACAAGCGGGAGGAGTTCCTGCGCTATGACAATGACCTGGGCAAGTACCTGGCAGTGGCTGGGTTGGGGCGGCAGGAGGCAGAAGACTGGAACACCAGGAAAGACCTCCTGGAGCAGAGGCGTTCCCAGCTGGACACCTTG

>DQ380433_Ctenomys_talarum_MHC_class_II_antigen_(Cta-DRB)_gene_Cta-DRB*0106_allele_exon_2_and_partial_cds

GAGCATGTTCAGATGGTGGTCAGACACATCTACAAGCGGGAGGGGTTCCTGCGCTATGACAATGACCTGGGCAAGTACCTGGCAGTGGCTGGGCTGGGGCGGCAGGAGGCAGAAGACTGGAACACCAGGAAAGACCTCCTGGAGCAGAGGCGTTCCCAGCTGGACACCTTG

>DQ380434_Ctenomys_talarum_MHC_class_II_antigen_(Cta-DRB)_gene_Cta-DRB*0107_allele_exon_2_and_partial_cds

GAGCATGTTCAGATGGTGGTCAGACACATCTACAAGCGGGAGGAGTTCCTGCGCTATGACAATGACCTGGGCAAGTACCTGGCAGTGGCTGGGCTGGGGCGGCAGGAGGCAGAAGACTGGAACACCAGGAAAGACCTCCTGGAGCAGAGGCGTTCCCAGCTGGACACCTTG

>DQ380435_Ctenomys_talarum_MHC_class_II_antigen_(Cta-DRB)_gene_Cta-DRB*0108_allele_exon_2_and_partial_cds

GAGCATGTTCAGATGGTGGTCAGACACATCTACAAGCGGGAGGAGTTCCTGCGCTATGACAATGACCTGGGCAAGTACCTGGCAGTGGCTGGGCTGGGGCGGCAGGAGGCGGAGGACTGGAACACCAGGAAAGACCTCCTGGAGCAGAGGCGTTCCCAGCTGGACACCTTG

>DQ380436_Ctenomys_talarum_MHC_class_II_antigen_(Cta-DRB)_gene_Cta-DRB*0109_allele_exon_2_and_partial_cds

GAGCATGTTCAGATGGTGGTCAGACACATCTACAAGCAGGAGGAGTTCTTACGCTACGACAGTGACCTGGGCAAGTACCTGGCAGTGGCTGGGTTGGGGCGGCAGGAGGCAGAGGACTGGAACACCAGGAAAGACCTCCTGGAGCAGAGGCATTCCCAGCTGGACACCTTG

>DQ380437_Ctenomys_talarum_MHC_class_II_antigen_(Cta-DRB)_gene_Cta-DRB*0110_allele_exon_2_and_partial_cds

GAGCATGTTCAGATGGTGGTCAGACACATCTACAAGCAGGAGGAGTTCTTACGCTACGACAGTGACCTGGGCAAGTACCTGGCAGTGGCTGGGTTGGGGCGGCAGGAGGCAGAAGACTGGAACACCAGGAAAGACCTCCTGGAGCAGAGGCGTTCCCAGCTGGACACCTTG

>DQ380438_Ctenomys_talarum_MHC_class_II_antigen_(Cta-DRB)_gene_Cta-DRB*0111_allele_exon_2_and_partial_cds

GAGCATGTTCAGATGGTGGTCAGACACATCTACAAGCGGGAGGAGTTCCTGCGCTATGACAGTGACCTGGGCAAGTACCTGGCAGTGACTGGGCTGGGGCGGCAGGAGGCAGAAGACTGGAACACCAGGAAAGACCTCCTGGAGCAGAGGCGTTCCCAGCTGGACACCTTG

>DQ380439_Ctenomys_talarum_MHC_class_II_antigen_(Cta-DRB)_gene_Cta-DRB*0112_allele_exon_2_and_partial_cds

GAGCATGTTCAGATGGTGGTCAGACACATCTACAAGCGGGAGGAGTTCTTGCGCTACGACAATGACCTGGGCAAGTACCTGGCAGTGGCTGGGTTGGGGCGGCAGGAGGCAGAAGACTGGAACACCAGGAAAGACCTCCTGGAGCAGAGGCGTTCCCAGCTGGACACCTTG

>DQ380440_Ctenomys_talarum_MHC_class_II_antigen_(Cta-DRB)_gene_Cta-DRB*0113_allele_exon_2_and_partial_cds

GAGCATGTTCAGATGGTGGTCAGACACATCTACAAGCAGGAGGAGTTCTTACGCTACGACAATGACCTGGGCAAGTACCTGGCAGTGGCTGGGTTGGGGCGGCAGGAGGCAGAAGACTGGAACACCAGGAAAGACCTCCTGGAGCAGAGGCGTTCCCAGCTGGACACCTTG

>DQ380441_Ctenomys_talarum_MHC_class_II_antigen_(Cta-DRB)_gene_Cta-DRB*0114_allele_exon_2_and_partial_cds

GAGCATGTTCAGATGGTGGTCAGACACATCTACAAGCAGGAGGAGTTCTTACGCTACGACAATGACCTGGGCAAGTACCTGGCAGTGGCTGGGTTGGGGCGGCAGGAGGCAGAAGACTGGAACACCAGGAAAGACCCCCTGGAGCAGAGGCGTTCCCAGCTGGACACCTTG

>DQ380442_Ctenomys_talarum_MHC_class_II_antigen_(Cta-DRB)_gene_Cta-DRB*0115_allele_exon_2_and_partial_cds

GAGCATGTTCAGATGGTGGTCAGACACATCTACAAGCAGGAGGAGTTCTTACGCTACGACAATGACCTGGGCAAGTACCTGGCAGTGGCTGGGTTGGGGCGGCAGGAGGCAGAAGACTGGAACACCAGGAAAGACCTCCTGGAGCAGAGGCATTCCCAGCTGGACACCTTG

>DQ380443_Ctenomys_talarum_MHC_class_II_antigen_(Cta-DRB)_gene_Cta-DRB*0116_allele_exon_2_and_partial_cds

GAGCATGTTCAGATGGTGGTCAGACACATCTACAAGCGGGAGGAGTTCCTGCGCTATGACAATGACCTGGGCAAGTACCTGGCAGTGGCTGGGCTGGGGCGGCAGGAGGCAGAAGACTGGAACACCAGGAAAGACCTCCTGGAGCAGAGGCGCTCCCAGCTGGACACCTTG

>DQ380444_Ctenomys_talarum_MHC_class_II_antigen_(Cta-DRB)_gene_Cta-DRB*0102_allele_exon_2_and_partial_cds

GAGCATGTTCAGATGGTGGTCAGACACATCTACAAGCGGGAGGAGTTCTTACGGTACGACAGTGACCTGGGCAAGTACCTGGCAGTGGCTGGGTTGGGGCGGCAGGAGGCAGAAGACTGGAACACCAGGAAAGACCTCCTGGAGCAGAGGCGTTCCCAGCTGGACACCTTG

>DQ380447_Ctenomys_talarum_MHC_class_II_antigen_(Cta-DRB)_gene_Cta-DRB*0104_allele_exon_2_and_partial_cds

GAGCATGTTCAGATGGTGGTCAGACACATCTACAAGCGGGAGGAGTTCCTGCGCTATGACAGTGACCTGGGCAAGTACCTGGCAGTGGCTGGGTTGGGGCGGCAGGAGGCAGAAGACTGGAACACCAGGAAAGACCTCCTGGAGCAGAGGCGTTCCCAGCTGGACACCTTG

>DQ380448_Ctenomys_talarum_MHC_class_II_antigen_(Cta-DRB)_gene_Cta-DRB*0105_allele_exon_2_and_partial_cds

GAGCATGTTCAGATGGTGGTCAGACACATCTACAAGCGGGAGGAGTTCCTGCGCTATGACAATGACCTGGGCGAGTACCTGGCAGTGGCTGGGTTGGGGCGGCAGGAGGCAGAAGACTGGAACACCAGGAAAGACCTCCTGGAGCAGAGGCGTTCCCAGCTGGACACCTTG

>EF434791_Myodes_glareolus_MHC_class_II_antigen_(Mygl-DRB)_gene_Mygl-DRB*01_allele_exon_2_and_partial_cds

CAGCGCGTGCGGTATCTGTACAGAGACATCTACAATCAGGAGGAGGTCGTGCGCTTCGACAGTGATGTGGGCGAGTATCACGCGGTGACCGAGCTGGGTCGCAGTGATGCTGAGGTCTGGAACAGCCAGAAGGAGGTCCTGGAGGACGCACGGGCTGCGGTGGACATGTAC

>EF434792_Myodes_glareolus_MHC_class_II_antigen_(Mygl-DRB)_gene_Mygl-DRB*03_allele_exon_2_and_partial_cds

CAGCGCGTGCGGTATCTGTACAGAGACATCTACAATCAGGAGGAGGTCGTGCGCTTCGACAGTGATGTGGGCGAGTATCACGCGGTGACCGAGCTGGGTCGCAGTGATGCTGAGGTCTGGAACAGCCAGAAGGAGGTCCTGGAGGACGCACGGGCCGCGGTGGACACGTAC

>EF434793_Myodes_glareolus_MHC_class_II_antigen_(Mygl-DRB)_gene_Mygl-DRB*04_allele_exon_2_and_partial_cds

--GCGCGTGCGGTATCTGTACAGATACATCTACAATCAGGAGGAGGTCGTGCGCTTCGACAGTGATGTGGGCGAGTATCACGCGGTGACCGAGCTGGGTCGCANTGATGCTGAGGTCTGGAACAGCCAGAAGGAGGTCCTGGAGGACGCACGGGCCGCGGTGGACACGTNC

>EF434794_Myodes_glareolus_MHC_class_II_antigen_(Mygl-DRB)_gene_Mygl-DRB*05_allele_exon_2_and_partial_cds

CAGCGCGTGCGGTATCTGTACAGAGACATCTACAACCAGGAGGAGGTCGTGCGCTTCGACAGTGATGTGGGCGAGTATCACGCGGTGACCGAGCTGGGTCGCAGTGATGCTGAGGTCTGGAACAGCCAGAAGGAGGTCCTGGAGGACGCACGGGCCGCGGTGGACACATAT

>EF434795_Myodes_glareolus_MHC_class_II_antigen_(Mygl-DRB)_gene_Mygl-DRB*06_allele_exon_2_and_partial_cds

-AGCGCGTGCGGTATCTGTACAGAGACATCTACANCCAGGAGGAGGTCGTGCGCTTCGACAGTGATGTGGGCGAGTATCACGCGGTGACCGAGCTGGGTCGCAGTGATGCTGATGTCTGGAACAGCCAGAAGGAGGTCCTGGAGGACGCACGGGCCGCGGTGGACACATAT

>EF434796_Myodes_glareolus_MHC_class_II_antigen_(Mygl-DRB)_gene_Mygl-DRB*07_allele_exon_2_and_partial_cds

CAGCGCGTGCGGTATCTGTACAGANACATCTACAACCAGGAGGAGGTCGTGCGCTTCGACAGTGATGTGGGCGAGTATCACGCGGTGACCGAGCTGGGTCGCAGTGATGCTGAGGTCTGGAACAGCCAGAAGGAGGTTCTGGAGGACGCACGGGCCGCGGTGGACACGTAC

>EF434797_Myodes_glareolus_MHC_class_II_antigen_(Mygl-DRB)_gene_Mygl-DRB*08_allele_exon_2_and_partial_cds

CAGCGCGTGCGGTATCTGTACAGAGACATCTACAACCAGGAGGAGGTCGTGCGCTTCGACAGTGATGTGGGCAAGTATCACGCGGTGACCGAGCTGGGTCGGAGTGATGCTGAGGTCTGGAACAGCCAGAAGGAGGTCCTGGAGGACGCACGGGCCGCGGTGGACACGTAC

>EF434798_Myodes_glareolus_MHC_class_II_antigen_(Mygl-DRB)_gene_Mygl-DRB*10_allele_exon_2_and_partial_cds

CAGCGCGTGCGGTATCTGTACAGAGACATCTACAACCAGGAGGAGGTCGTGCGCTTCGACAGTGATGTGGGCAAGTATCACGCGGTGACCGAGCTGGGTCGGAGTGATGCTGAGGTCTGGAACAGCCAGAAGGAGGTCCTGGAGGACGCACGGGCCGCGGTGGACACGTAT

>EF434799_Myodes_glareolus_MHC_class_II_antigen_(Mygl-DRB)_gene_Mygl-DRB*11_allele_exon_2_and_partial_cds

CAGCGCGTGCGGTATCTGTACAGAGACATCTACAACCAGGAGGAGGTCGTGCGCTTCGACAGTGATGTGGGCAAGTATCACGCGGTGACCGAGCTGGGTCGGAGTGATGCTGAGGTCTGGAACAGCCAGAAGGAGGTCCTGGAGGACGCACAGGCCGCGGTGGACACGTAC

>EF434800_Myodes_glareolus_MHC_class_II_antigen_(Mygl-DRB)_gene_Mygl-DRB*12_allele_exon_2_and_partial_cds

CAGCGCGTGCGGTATCTGTACAGAGACATCTACAACCAGGAGGAGGTCGTGCGCTTCGACAGTGATGTGGGCGAGTATCATGCGGTGACTGAGCTGGGTCGGAGTGATGCTGAGGTCTGGAACAGCCAGAAGGAGGTCCTGGAGGACGCACAGGCCGCGGTGGACACATAC

>EF434801_Myodes_glareolus_MHC_class_II_antigen_(Mygl-DRB)_gene_Mygl-DRB*13_allele_exon_2_and_partial_cds

CAGCGCGTGCGGTATCTGTACAGAGACATCTACAACCAGGAGGAGGTCGTGCGCTTCGACAGTGATGTGGGCGAGTATCATGCGGTGACTGAGCTGGGTCGGAGTGATGCTGAGGTCTGGAACAGCCAGAAGGAGGTCCTGGAGGACGCACGGGCCGCGGTGGACACATAC

>EF434802_Myodes_glareolus_MHC_class_II_antigen_(Mygl-DRB)_gene_Mygl-DRB*14_allele_exon_2_and_partial_cds

CAGCGCGTGCGGTATCTGTACAGAGACATCTACAACCAGGAGGAGGTCGTGCGCTTCGACAGTGATGTGGGCGAGTATCATGCGGTGACTGAGTTGGGTCGGAGTGATGCTGAGGTCTGGAACAGCCAGAAGGAGGTCCTGGAGGACGCACAGGCCGCGGTGGACACATAC

>EF434803_Myodes_glareolus_MHC_class_II_antigen_(Mygl-DRB)_gene_Mygl-DRB*15_allele_exon_2_and_partial_cds

CAGCGCGTGCGGTATCTGTACAGAGACATCTACAACCAGGAAGAGGTCGTGCGCTTCGACAGTGATGTGGGCAAGTATCACGCGGTGACCGAGTTGGGTCGGAGTGATGCTGAGGTCTGGAACAGCCAGAAGGAGGTCCTGGAGGATGCACGGGCCGCGGTGGACACTTAC

>EF434804_Myodes_glareolus_MHC_class_II_antigen_(Mygl-DRB)_gene_Mygl-DRB*16_allele_exon_2_and_partial_cds

CAGCACGTGCGGCTTCTGGACAGATTCTTCTACAACCGGGAGGAGTACGTGCGCTTTGACAGCGACTTGGGCGAGTTCCGTGCGGTGACCGAGCTGGGGCGGCCCTCAGCCAAGTACTGGAACAGCCAGAAGGAGATCCTGGACTACAGGCGGGCCGCGCTGGACACGTAC

>EF434805_Myodes_glareolus_MHC_class_II_antigen_(Mygl-DRB)_gene_Mygl-DRB*17_allele_exon_2_and_partial_cds

CAGCGCGTGCGGTTTCTGGACAGATACTTCTACAACCGGGAGGAGCTCGTGCGCTTTGACAGCGACATTGGCGAGCACCGTGCGGTGAACGAGCTGGGGCGGCGGGACGCCGAGTACTGGAACAGCCAGAAGGACATCCTGGAGCGGAAGCGGGCCTATGTGGACACGTTC

>EF434806_Myodes_glareolus_MHC_class_II_antigen_(Mygl-DRB)_gene_Mygl-DRB*18_allele_exon_2_and_partial_cds

CAGCGCGTGCGGTTTCTGGACAGATACTTCTACAACCAGGAGGAGTACGCACGCTTCGACAGCGACATTGGCGAGCACCGTGCAGTGAACGAGCTGGGGCGGCCGGACGCCGAGTACTGGAACAGCCAGAAGGAGCTCCTGGAGCAGAAGCGGGCCTATGTGGACACGTTC

>EF434807_Myodes_glareolus_MHC_class_II_antigen_(Mygl-DRB)_gene_Mygl-DRB*19_allele_exon_2_and_partial_cds

------------------------------TACAACCAGGAGGAGTATGCACGCTTTGACAGTGATGTGGGCAAGTACCGCGCAGTGAACGAGCTGGGGCGGCCTGACTCCGAGTACTGGAACAGCCAGGAGGAGCTCCTGGAGCAGAAGCGGTCCCTTGTGGACACGTAC

>EF434808_Myodes_glareolus_MHC_class_II_antigen_(Mygl-DRB)_gene_Mygl-DRB*20_allele_exon_2_and_partial_cds

------------------------------TACAACCAGGAGGAGTATGCACGCTTTGACAGTGATGTGGGCGAGTACCGCGCAGTGAAGGAGCTGGGGCTGCCGGACTCCGAGTACTGGAACAGCCAGGAGGAGCTCCTGGAGCAGAAGCGGTCCCTTGTGGACACGTAC

>EF434809_Myodes_glareolus_MHC_class_II_antigen_(Mygl-DRB)_gene_Mygl-DRB*21_allele_exon_2_and_partial_cds

CAGCACGTGCGGTTTCTGGACAGATACTTCTACAACCGGGAGGAGTACGTGCGCTTCGACAGTGACGTGGGCGAGTACCGTGCGGTGACCGAGCTGGGGCGGCCGGACGCCAAGTACTGGAACAGTCGGAAGGAGATCCTGGAGCAGAAGCGGGCCGCGGTGGACGCATTC

>EF434811_Myodes_glareolus_MHC_class_II_antigen_(Mygl-DRB)_gene_Mygl-DRB*23_allele_exon_2_and_partial_cds

------------------------------TACAACCAGGAGGAGTATGCACGCTTTGACAGTGATGTGGGCAAGTACCGCGCAGTGAATGAGCTGGGGCGGCCTGACTCCGAGTACTGGAACAGCCAGGAGGAGCTCCTGGAGCAGAAGCGGTCCCTTGTGGACACGTAC

>EF434812_Myodes_glareolus_MHC_class_II_antigen_(Mygl-DRB)_gene_Mygl-DRB*24_allele_exon_2_and_partial_cds

------------------------------TACAACCAGGAGGAGTACGCACGCTTTGACAGTGATGTGGGCGAGTACCGCGCAGTGAACGAGCTGGGGCGGCCTGACTCCGAGTACTGGAACAGCCAGGAGGAGCTCCTGGAGCAGAAGCGGTCCCTTGTGGACACGTAC

>EF434814_Myodes_glareolus_MHC_class_II_antigen_(Mygl-DRB)_gene_Mygl-DRB*26_allele_exon_2_and_partial_cds

------------------------------TACAACCAGGAGGAGTACGCACGCTTTGACAGTGATGTGGGCAAGTACCGCGCAGTGAACGAGCTGGGGCGGCCTGACTCCGAGTACTGGAACAGCCAGGAGGAGCTCCTGGAGCAGAAGCGGTCCCTTGTGGACACGTAC

>EF434815_Myodes_glareolus_MHC_class_II_antigen_(Mygl-DRB)_gene_Mygl-DRB*27_allele_exon_2_and_partial_cds

------------------------------TACAACCAGGAGGAGTATGCACGCTTTGACAGTGACGTGGGCGAGTACCGCGCAGTGAAGGAGCTGGGGCTGCCGGACTCCGAGTACTGGAACAGCCAGGAGGAGCTCCTGGAGCAGAAGCGGTCCCTTGTGGACACGTAC

>EF434816_Myodes_glareolus_MHC_class_II_antigen_(Mygl-DRB)_gene_Mygl-DRB*28_allele_exon_2_and_partial_cds

------------------------------TACAACCAGGAGGAGTACGCACGCTTTGACAGTGACGTGGGCGAGTACCGCGCAGTGAACGAGCTGGGGCGGCCTGACTCCGAGTACTGGAACAGCCAGGAGGAGCTCCTGGAGCAGAAGCGGTCCCTTGTGGACACGTAC

>EF469837_Myodes_glareolus_MHC_class_II_antigen_(Mygl-DRB)_gene_Mygl-DRB*29_allele_exon_2_and_partial_cds

CAGCGCGTGCGGTTTCTGGACAGATACTTCTACAACCGGGAGGAGTACGCACGCTTTGACAGTGATGTGGGCGAGTACCGCGTAGTGAACGAGCTGGGGCTGCCTGACTCCGAGTACTGGAACAGCCAGGAGGAGCTCCTGGAGCAGAAGCGGTCCCTTGTGGACACGTAC

>EF469839_Myodes_glareolus_MHC_class_II_antigen_(Mygl-DRB)_gene_Mygl-DRB*30_allele_exon_2_and_partial_cds

CAGCGCGTGCGGTATCTGTACAGAGACATCTACAACCAGGAGGAGTTCGTGCGCTTCGACAGTGATGTGGGCGAGTACCGTGCGGTGACCGAGCTGGGTCGGAGAGATGCTGAGGTCTGGAACAGCCAGAAGGAGCTCCTGGACAACAGGCGGGCCTATGTGGACACGTAC

>EF469841_Myodes_glareolus_MHC_class_II_antigen_(Mygl-DRB)_gene_Mygl-DRB*31_allele_exon_2_and_partial_cds

CAGCGCGTGCGGTTTCTGGACAGATACTTCTACAACCGGGAGGAGTATGCACGCTTTGACAGTGATGTGGGCGAGTACCGCGCAGTGAAGGAGCTGGGGCTGCCGGACTCCGAGTACTGGAACAGCCAGGAGGAGCTCCTGGAGCAGAAGCGGTCCCTTGTGGACACGTAC

>EF469842_Myodes_glareolus_MHC_class_II_antigen_(Mygl-DRB)_gene_Mygl-DRB*32_allele_exon_2_and_partial_cds

CAGCACGTGCGGTATCTGGACAGATACTTCTACAACCGGAAGGAGTACGTGCGCTTTGACAGCGACGTGGGCGAGTACCGTGCAGTGACCGAGCTGGGGCGACCGGACGCCAAGTACTGGAACAGCCAGAAGGAGCTCCTGGAGCAGAAGCGGGCCAAGGTGGACACATAC

>EF469843_Myodes_glareolus_MHC_class_II_antigen_(Mygl-DRB)_gene_Mygl-DRB*33_allele_exon_2_and_partial_cds

CAGCATGTGCTGTTTGTGGAGAGATTCATCTACAACCGGGAAGAGTATGTGCGCTTCGACAGTGACGTGGGCGAGTACCGTGCGCTGAATGAGCTGGGGCGGCCGGACGCTGAGTACTGGAACAGCAGGAAGGAGATCCTGGACAACAGGCGGGCCGCGGTGGACACGTAC

>EF469847_Myodes_glareolus_MHC_class_II_antigen_(Mygl-DRB)_gene_Mygl-DRB*35_allele_exon_2_and_partial_cds

CAGCGCGTGCGGTATCTGTACAGAGACATCTACAACCAGGAGGAGTTCGTGCGCTTCGACAGTGATGTGGGCGAGTATCACGCGGTGACCGAGCTGGGTCGGAGAGATGCTGAGGTCTGGAACAGCCAGAAGGAGCTCCTGGACAACAGGCGGGCCTATGTGGACACGTAC

>EF469848_Myodes_glareolus_MHC_class_II_antigen_(Mygl-DRB)_gene_Mygl-DRB*36_allele_exon_2_and_partial_cds

CAGCGCGTGCGGTTTCTGGACAGATACTTCCACAACCAGGAGGAGTACGCACGCTTTGACAGTGATGTGGGCGAGTACCGCGCAGTGAACGAGCTGGGGCTGCCGGACTCCGAGTACTGGAACAGCCAGGAGGAGCTCCTGGAGCAGAAGCGGTCCCTTGTGGACACGTAC

>EF469849_Myodes_glareolus_MHC_class_II_antigen_(Mygl-DRB)_gene_Mygl-DRB*37_allele_exon_2_and_partial_cds

CGGCGCGTGCGGTTTCTGGACAGATACTTCTACAACCAGGAGGAGTACGCACGCTTTGACAGTGACGTGGGCGAGTACCGCGCAGTGAACGAGCTGGGGCGGCCTGACTCCGAGTACTGGATCAGCCAGGAGGAGCTCCTGGAGCAGAAGCGGTCCCTTGTGGACACGTAC

>EF469850_Myodes_glareolus_MHC_class_II_antigen_(Mygl-DRB)_gene_Mygl-DRB*38_allele_exon_2_and_partial_cds

CAGCGTGTGCAGTTTGTGGAGAGATACATCTCCAACCAGGAGGAAAACGTGCGCTTTGACAGCGACGTGGGCGAGTACCGTGCAGTGACTGAGCTGGGGCGGCCAGACGCCGAGTACTGGAACAGCCAGAAGGAGATCCTGGAGGACGCACGGGCCGCGGTGGACACGTAC

>EF469851_Myodes_glareolus_MHC_class_II_antigen_(Mygl-DRB)_gene_Mygl-DRB*39_allele_exon_2_and_partial_cds

CAGCGCGTGCGGTATCTGTTCAGAGACATCTACAACCAGGAGGAGCATGTGCGCTTCGACAGCGACGTGGGCGAGTTCCGCGCGGTGACCGAGCTGGGGCGGCCAGACGCCGAGTACTGGAACAGCCAGAAGGACATCCTGGAGCGGACGCGGGCCGAGACGGACACGGTG

>EF507430_Arvicola_terrestris_MHC_class_II_antigen_(Arte-DRB)_gene_Arte-DRB*22_allele_exon_2_and_partial_cds

CAGCGCGTGCGGTATCTGGTCAGAGACTTCTACAACCGGGAGGAGTACGTGCGCTTCGACAGCGACGTGGGCGAGTACCGCGCGGTGACCGAGCTGGGGCGGCGCTCAGCCGAGTACTGGAACAGCCAGAAGGACATCCTGGAGCGGAAGCGGGCCGAGATAGACACGGTG

>EF507431_Arvicola_terrestris_MHC_class_II_antigen_(Arte-DRB)_gene_Arte-DRB*23_allele_exon_2_and_partial_cds

CAGCGCGTGCGGCTTCTAGAGAGATACATCTACAACCGGGAGGAGTACGTGCGCTTCGACAGCGACGTGGGCGAGCACCGCGCGGTGACCGAGCTGGGGCGGGGCATAGCCGAGAACTTGAACAGCCGGAAGGAGCTCCTGGAGAACAGGCGGGCCGCGGTGGACACGGTG

>EF569186_Spermophilus_suslicus_MHC_class_II_antigen_(Spsu-DRB)_gene_Spsu-DRB*01_allele_exon_2_and_partial_cds

GAGCGGGTGCGGCTCCTACACAGATACATCTACAACCGGGAGGAGGTCGCGCGCTTCGACAGCGACGTCGGGGAGTACCGCGCGGTGACCGAGCTGGGGCGGCCGTCAGCCGAGTACTTCAACAGCCAGAAGGACCTCCTGGAGCGGAGGCGGGCCGAGGTGGACACTGTG

>EF569187_Spermophilus_suslicus_MHC_class_II_antigen_(Spsu-DRB)_gene_Spsu-DRB*02_allele_exon_2_and_partial_cds

GAGCGGGTGCGGTTCCTGGAGAGATACTTCCACAACCGGGAGGAGTTCGCGCGCTTCGACAGCGACGTGGGGGAGTACCGCGCGGTGACCGAGCTGGGGCGGCCGGACGCCGAGTACTGGAACAGCCAGAGGGACTTCCTGGAGCGGAAGCGGGCGGAGGTGGACACGTTC

>EF569188_Spermophilus_suslicus_MHC_class_II_antigen_(Spsu-DRB)_gene_Spsu-DRB*03_allele_exon_2_and_partial_cds

GAGCGGGTGCGGTTCCTGGTCAGATACTTCCACAACCGGGAGGAGTTCGTGCGCTTCGACAGCGACGTGGGGGAGTTCCGCGCGGTGAGCGAGCTGGGGCGGCCGGACGCCGAGAGCTGGAACCGCCAGAAGGACGTCCTGGAGGAGAGGCGGGCCCAGGTGGACAACTAC

>EF569189_Spermophilus_suslicus_MHC_class_II_antigen_(Spsu-DRB)_gene_Spsu-DRB*04_allele_exon_2_and_partial_cds

GAGCGGGTGCAGTTACTGGTCAGATACTTCTACAACCGGGAGGAGTTCGCGCGCTTCGACAGCGACGTGGGGGAGTACCGCGCGGTGACCGAGGTGGGGCGGCAGGACGCCAAGTACTGGAACAGCCAGAAGGACGCCCTGGAGCGGAGGCGGGCCGAGGTGGACACTGTG

>EF569190_Spermophilus_suslicus_MHC_class_II_antigen_(Spsu-DRB)_gene_Spsu-DRB*05_allele_exon_2_and_partial_cds

GAGCGGATACGGTTCCTGGACAGATACTTCTACAACCGGGAGGAGTACGTGCGCTTCGACAGCGACGTGGGGGAGTTCCGCGCGGTGAGCGAGCTGGGGCGGCCGGACGCCGAGTACTGGAACAGCCAGAAGGACTTCCTGGAGGATGAGCGGGCCACGGTGGACAACTAC

>EF569191_Spermophilus_suslicus_MHC_class_II_antigen_(Spsu-DRB)_gene_Spsu-DRB*06_allele_exon_2_and_partial_cds

GAGCGGGTGCGGTTCCTGGTCAGATACTTCTACAACCGGGAGGAGTACGTGCGCTTCGACAGCGACGTGGGGGAGTTCCGCGCGGTGACCGAGCTGGGGCGGCCGGACGCCGAGTACTGGAACCGCCAGAAGGACGTCCTGGAGGGGAGGCGGGCCCAGGTGGACAACTAC

>EF569192_Spermophilus_suslicus_MHC_class_II_antigen_(Spsu-DRB)_gene_Spsu-DRB*07_allele_exon_2_and_partial_cds

GAGCGGGTGCGGTTCCTGGAGAGATACTTCTACAACCGGGAGGAGTACGTGCGCTTCGACAGCGACGTGGGGGAGTACCGAGCGGTGACCGAGCTGGGGCGGCGGGACGCCGAGTACTGGAACAGCCAGAAGGACAGCCTGGAGTATAAGCGGGGACAGGTGGACAACTAC

>EF569193_Spermophilus_suslicus_MHC_class_II_antigen_(Spsu-DRB)_gene_Spsu-DRB*08_allele_exon_2_and_partial_cds

GAGCGGGTGCGGTTCCTGGAGAGACACTTCTACAACCGGGAGGAGAACGTGCGCTTCGACAGCGACGTGGGGGAGTACCGCGCGGTGACCGAGCTGGGGCGGCCGGACGCCGAGTACTGGAACAGCCAGAAGGACGTCCTGGAGTATAAGCGGGGACAGGTGGACAACTAC

>EF569194_Spermophilus_suslicus_MHC_class_II_antigen_(Spsu-DRB)_gene_Spsu-DRB*09_allele_exon_2_and_partial_cds

GAGCGGATAAGGTTCCTACACAGATACATCTACAACCGGGAGGAGGTCGCGCGCTTCGACAGCGACGTGGGGGAGTACCGCGCGGTGAGCGAGCTGGGGCGGCGGACAGCCGAGTACTGGAACAGCCAGAAGGACCTCCTGGAGCGGAAGCGGGCCGTGGTGGACACAGTG

>EF569195_Spermophilus_suslicus_MHC_class_II_antigen_(Spsu-DRB)_gene_Spsu-DRB*10_allele_exon_2_and_partial_cds

GAGCGGGTGCGGTTCCTGGAGAGATACTTCCACAACCGGGAGGAGTTCGTGCGCTTCGACAGCGACGTGGGGGAGTACCGCGCGGTGACCGAGCTGGGGCGGCGGGACGCCGAGTACTGGAACAGCCAGAAGGACAGCCTGGAGTATAAGCGGGGACAGGTGGACAACTAC

>EF569196_Spermophilus_suslicus_MHC_class_II_antigen_(Spsu-DRB)_gene_Spsu-DRB*11_allele_exon_2_and_partial_cds

GAGCGGGTGCGGTTCCTGGACAGATACTTCCACAACCGGGAGGAGTTCGTGCGCTTCGACAGCGACGTGGGGGAGTACCGCGCGGTGACCGAGCTGGGGCGGCCGTCAGCCGAGTACTGGAACAGCCAGAAGGACCTCCTGGAGCAGAAGCGGGGACAGGTGGACAACTAC

>EF569197_Spermophilus_suslicus_MHC_class_II_antigen_(Spsu-DRB)_gene_Spsu-DRB*12_allele_exon_2_and_partial_cds

GAGCGGGTGCGGCTCCTGGACAGATACTTCTACAACCGGGAGGAGTACGTGCGCTTCGACAGCGACGTGGGGGAGTACCGCGCGGTGACAGAGCTGGGGCGGCCGGACGCCGAGTACTGGAACAGCCAGAAGGACATCCTGGAGGATGCGCGGGCCTCGGTGGACAACTAC

>EF569198_Spermophilus_suslicus_MHC_class_II_antigen_(Spsu-DRB)_gene_Spsu-DRB*13_allele_exon_2_and_partial_cds

GAGCGGATACGGTTCCTGGACAGATACTTCTACAACCGGGAGGAGTACGCGCGCTTCGACAGCGACGTGGGGGAGTTCCGCGCGGTGAGCGAGCTGGGGCGGCCGGACGCCGAGTACTGGAACAGCCAGAAGGACGTCCTGGAGGAGAGGCGGGCCCAGGTGGACAACTAC

>EF569199_Spermophilus_suslicus_MHC_class_II_antigen_(Spsu-DRB)_gene_Spsu-DRB*14_allele_exon_2_and_partial_cds

GAGCGGGTGCGGTTCCTGGACAGATACTTCCACAACCGGGAGGAGTTCGTGCGCTTCGACAGCGACGTGGGGGAGTTCCGCGCGGTGAGCGAGCTGGGGCGGCTGGACGCCGAGAGCTGGAACAGCCAGAAGGACTTCCTGGAGCGGAAGCGGGCCGAGGTGGACACGTTC

>EF569200_Spermophilus_suslicus_MHC_class_II_antigen_(Spsu-DRB)_gene_Spsu-DRB*15_allele_exon_2_and_partial_cds

GAGCGGGTGCAGTTACTGGTCAGATTCTTCTACAACCGGGAGGAGTTCGCGCGCTTCGACAGCGACGTGGGGGAGTACCGCGCGGTGACCGAGGTGGGGCGGCAGGACGCCAAGTACTGGAACAGCCAGAAGGACTCCCTGGAGCGGAGGCGGGCCGAGGTGGACACTGTA

>EF569201_Spermophilus_suslicus_MHC_class_II_antigen_(Spsu-DRB)_gene_Spsu-DRB*16_allele_exon_2_and_partial_cds

GAGCGGGTGCGGTTCCTGGACAGATACTTCCACAACCGGGAGGAGTTCGTGCGCTTCGACAGCGACGTGGGGGAGTACCGCGCGGTGACCGAGCTGGGGCGGCCGTACGCCAAGTACTGGAACAGCCAGAAGGACTTCCTGGAGCAGAAGCGGGGACAGGTGGACAACTAC

>EF660500_Arvicola_terrestris_MHC_class_II_antigen_(Arte-DRB)_gene_Arte-DRB*06_allele_partial_cds

CAGCGCGTGCGGTTTCTGGTCAGAGACATCTACAACCGGGAGGAGGTCGTGCGCTTCGACAGCGACGTGGGCGAGTTCCGCGCGGTGACCGAGCTGGGGCGGCCGGACGCCGAGTACTTCAACAGCCTGAAGGACTTCCTGGAGCAGAAGCGGGCCGAGGTGGACACGTAC

>EF660501_Arvicola_terrestris_MHC_class_II_antigen_(Arte-DRB)_gene_Arte-DRB*07_allele_partial_cds

CAGCGCGTGCGGTTTCTGGTCAGATACATCTACAACCGGGAGGAGTTCGTGCGCTTCGACAGCGACGTGGGCGAGCACCGCGCGGTGACCGAGCTGGGGCGGGGCATAGCCGAGAACTTCAACAGCCGGAAGGAGCTCCTGGAGCGGACGCGGGCCGCGGTGGACACGGTG

>EF660502_Arvicola_terrestris_MHC_class_II_antigen_(Arte-DRB)_gene_Arte-DRB*08_allele_partial_cds

CAGCGCGTGCGGTATCTGGTCAGAGACTTCTACAACCGGGAGGAGTACGTGCGCTTCGACAGCGAGGTGGGCGAGTACCGCGCGGTGACCGAGCTGGGGCGGCGCTCAGCCGAGTACTGGAACAGCCAGAAGGACGTCCTGGAGCGGAAGCGGGCCGAGATAGACACGGTG

>EF660503_Arvicola_terrestris_MHC_class_II_antigen_(Arte-DRB)_gene_Arte-DRB*09_allele_partial_cds

CAGCGCGTGCGGTTTCTGGACAGATACTTCTTCAACCGGGAGGAGTACGTGCGCTTCGACAGCGACGTGGGCGAGTTCCGCGCGGTGACCGAGCTGGGGCGGGGCATAGCCGAGAACTTGAACAGCCAGAAGGACATCCTGGAGCGGTTGCGGGCCGAGGTAGACACGGTG

>EF660504_Arvicola_terrestris_MHC_class_II_antigen_(Arte-DRB)_gene_Arte-DRB*10_allele_partial_cds

CAGCGCGTGCGGTTTCTGGACAGATACATCCACAACCAGGAGGAGTTCGTGCGCTTCGACAGCGACGTGGGCGAGCACCGCGCGGTGACCGAGCTGGGGCGGGGCATAGCCGAGTACTGGAACAGCCAGAAGGACATCCTGGAGCGGAAGCGGGCCGCGGTGGACACGTAC

>EF660505_Arvicola_terrestris_MHC_class_II_antigen_(Arte-DRB)_gene_Arte-DRB*11_allele_partial_cds

CAGCGCGTGCGGTATCTGGTCAGAGTCATCCACAACCGGGAGGAGTACGTGCGCTTCGACAGCGACGTGGGCGAGTACCGCGCGGTGACCGAGCTGGGGCGGGGCATAGCCGAGTACTTGAACAGCCTGAAGGACTTCATGGAGCAGAAGCGGGCCGCGGTGGACACGTAC

>EF660506_Arvicola_terrestris_MHC_class_II_antigen_(Arte-DRB)_gene_Arte-DRB*12_allele_partial_cds

CAGCGCGTGCGGTTTCTGGACAGATACATCCACAACCAGGAGGAGTTCATGCGCTTCGACAGCGACGTGGGCGAGCACCGCGCGGTGACCGAGCTGGGGCGGGGCATAGCCGAGTACTGGAACAGCCAGAAGGACATCCTGGAGCGGAAGCGGGCCGCGGTGGACACGTAC

>EF660507_Arvicola_terrestris_MHC_class_II_antigen_(Arte-DRB)_gene_Arte-DRB*13_allele_partial_cds

CAGCGCGTGCGGCTTCTGGACAGATACTTCTTCAACCAGGAGGAGTACTTGCGCTTCGACAGCGACGTGGGCGAGTTCCGCGCGGTGACCGAGCTGGGGCGGCCGGACGCCGAGTACTACAACAGCCTGAAGGACTTCATGGAGGACAGGCGGGCCGCGGTGGACACGTAC

>EF660508_Arvicola_terrestris_MHC_class_II_antigen_(Arte-DRB)_gene_Arte-DRB*14_allele_partial_cds

CAGCGCGTGCGGTATCTGGTCAGAGACATCTACAACCTGGAGGAGGTCGTGCGCTTCGACAGCGACGTGGGCGAGTACCGCGCGGTGACCGAGCTGGGGCGGCCGGACGCCGAGTACTGGAACAGCCTGAAGGACGCCCTGGAGCGGAAGCGGGCCGCGGTGGACACGGTG

>EF660509_Arvicola_terrestris_MHC_class_II_antigen_(Arte-DRB)_gene_Arte-DRB*15_allele_partial_cds

CAGCGCGTGCGGCTTCTGGACAGAGTCATCTACAACCGGGAGGAGTACGTGCGCTTCGACAGCGACGTGGGCGAGTTCCGCGCGGTGACCGAGCTGGGGCGGCGCTCAGCCGAGTACCGGAACAGCCTGAAGGACTTCATAGAGCAGTTGCGGGCCTCGGTGGACACGTAC

>EF660510_Arvicola_terrestris_MHC_class_II_antigen_(Arte-DRB)_gene_Arte-DRB*16_allele_partial_cds

CAGCGCGTGCGGTTTCTAGAGAGATACTTCTACAACCGGGAGGAGTACGTGCGCTTCGACAGCGACGTGGGCGAGTACCGCGAGGTGACCGAGCTGGGGCGGGGCATAGCCGAGAACTTGAACAGCCGGAAGGAGCTCCTGGAGCAGAAGCGGGCCGAGATAGACACGTAC

>EF660511_Arvicola_terrestris_MHC_class_II_antigen_(Arte-DRB)_gene_Arte-DRB*17_allele_partial_cds

CAGCGCGTGCGGTATCTGGTCAGAGACATCTACAACCTGGAGGAGGTCGTGCGCTTCGACAGCGACGTGGGCGAGTACCGCGCGGTGACCGAGCTGGGGCGGGGCATAGCCGAGTACTGGAACAGCCTGAAGGACGCCCTGGAGCGGAAGCGGGCCGCGGTGGACACGGTG

>EF660512_Arvicola_terrestris_MHC_class_II_antigen_(Arte-DRB)_gene_Arte-DRB*18_allele_partial_cds

CAGCGCGTGCGGTTTCTGGACAGATACTTCTTCAACCGGGAGGAGTACGTGCGCTTCGACAGCGACGTGGGCGAGTACCGCGCGGTGACCGAGCTGGGGCGGCGCTCAGCCGAGTACTGGAACAGCCAGAAGGAGCTCCTGGAGAACAGGCGGGCCGAGGTAGACACGGTG

>EF660513_Arvicola_terrestris_MHC_class_II_antigen_(Arte-DRB)_gene_Arte-DRB*19_allele_partial_cds

CAGCGCGTGCGGCTTCTGGTCAGAGACATCTACAACCGGGAGGAGGTCGTGCGCTTCGACAGCGACGTGGGCGAGTTCCGCGCGGTGACCGAGCTGGGGCGGCCGGACGCCGAGTACTTCAACAGCCTGAAGGACTTCCTGGAGCAGAAGCGGGCCGAGATAGACACGTTC

>EF660514_Arvicola_terrestris_MHC_class_II_antigen_(Arte-DRB)_gene_Arte-DRB*20_allele_partial_cds

CAGCGCGTGCGGTTTCTGGACAGATACATCCACAACCAGGAGGAGTTCGTGCGCTTCGACAGCGACGTGGGCGAGCACCGCGCGGTGACCGAGCTGGGGCGGGGCATAGCCGAGTACTGGAACAGCCAGAAGGAGCTCCTGGAGCGGAAGCGGGCCGCGGTGGACACGTAC

>EF660515_Arvicola_terrestris_MHC_class_II_antigen_(Arte-DRB)_gene_Arte-DRB*21_allele_partial_cds

CAGCGCGTGCGGCTTCTGGACAGATACTTCTTCAACCAGGAGGAGTACGTGCGCTTCGACAGCGACGTGGGCGAGTTCCGCGCGGTGACCGAGCTGGGGCGGCCGGACGCCGAGTACTACAACAGCCTGAAGGACTTCATGGAGGACAGGCGGGCCGCGGTGGACACGTAC

>EU817479_Dipodomys_spectabilis_MHC_class_II_antigen_(Disp-DRB)_mRNA_Disp-DRB*01_allele_complete_cds

CAGCGGGUGCGGUUUGUGCGGAGAUACAUCUACAACCGGGAGGAGUUCCUGCGCUUCGACAGCGAGGUCGGGGAGUACCGCGCGGUGAAUGAGCUGGGGCGGCCCAUCGCCAAGGACUGGAACGGGCAGCAGGACCUCCUGGAGCAGAAGCGGGCGGCGGUGGACACCUAC

>EU817480_Dipodomys_spectabilis_MHC_class_II_antigen_(Disp-DRB)_mRNA_Disp-DRB*02_allele_complete_cds

CAGCGGGUGCGCUACGUGCGGAGAUACAUCUACAACCGGGAGGAGUUCCUGCGCUUCGACAGCGACGUCGGGGAGUACCGCGCGGUGAAUGAGCUGGGGCGGCCCAUCGCCAAGGACUGGAACGGGCAGCAGGACCUCCUGGAGCAGAAGCGGGCGGCGGUGGACACCUAC

>EU817481_Dipodomys_spectabilis_MHC_class_II_antigen_(Disp-DRB)_mRNA_Disp-DRB*03_allele_complete_cds

CAGCGGGUGCGGUUUGUGCGGAGAUACAUCUACAACCGGGAGGAGUUCCUGCGCUUCGACAGCGACGUCGGGGAGUACCGCGCGGUGAAUGAGCUGGGGCGGCCCAUCGCCAAGGACUGGAACGGGCAGCAGGACCUCCUGGAGCAGAAGCGGGCGGCGGUGGACACCUAC

>EU817482_Dipodomys_spectabilis_MHC_class_II_antigen_(Disp-DRB)_mRNA_Disp-DRB*04_allele_complete_cds

CAGCGGGUGCGCUUACUGGUGAGAUUCAUCUACAACCGGGAGGAGUUCCUGCGCUUCGACAGCGAGGUCGGGGAGUACCGCGCGGUGACCGAGCUGGGGCGGCCCAUCGCCAAGGACUGGAACGGGCAGCAGGACCUCCUGGAGCAGAAGCGGGCGGAGGUGGACACCGUG

>EU817483_Dipodomys_spectabilis_MHC_class_II_antigen_(Disp-DRB)_mRNA_Disp-DRB*05_allele_complete_cds

CAGCGGGUGCGCUAUCUGGACGGAUACUUCUACAACCGGGAGGAGUACGUGCGCUUCGACAAUGAGGUCGGGGAGUACCGCGCGGUGAGCGAGCUGGGGCGGCGGGACGCCGAGUACUGGAACAGCCAGCAGGACUUCCUGGAGUACAAGCGGGGGCAGGUGGACAACUAC

>GQ901811_Myodes_glareolus_MHC_class_II_antigen_(Mygl-DRB)_gene_Mygl-DRB*40_allele_exon_2_and_partial_cds

---------------------------------------GAAGAGTACTTGCGCTTCGACAGCGATGTGGGCGAGTTCCGTGCGGTGAATGAGCTGGGGCGGCTGGACGCTGAATACTGGAACAGCAGGAAGGAGATCCTGGACAACAGGCGGGCCGCGGT----------

>GQ901812_Myodes_glareolus_MHC_class_II_antigen_(Mygl-DRB)_gene_Mygl-DRB*41_allele_exon_2_and_partial_cds

---------------------------------------GAGGAAAACGTGCGCTTCGACAGTGACGTGGGCAAGTACCTTGCTGTGACCAAGCTGGGGCGGCTGGAGGCGGAGAACTGGAACAGTCGGAAGGAGCTCCTGGAGGACGCAAGGGCCGGGGT----------

>GQ901813_Myodes_glareolus_MHC_class_II_antigen_(Mygl-DRB)_gene_Mygl-DRB*42_allele_exon_2_and_partial_cds

---------------------------------------GAGGAAAACGTGCGCTTCGACAGTGATGTGGGCAAGTACCTTGCTGTGACCAAGCTGGGGCAGCTGGAGGCGGAGAACTGGAACAGCCGGAAGGAGCTCCTGGAGGACGCACGGGCCGGGGT----------

>GQ901814_Myodes_glareolus_MHC_class_II_antigen_(Mygl-DRB)_gene_Mygl-DRB*43_allele_exon_2_and_partial_cds

---------------------------------------GAAGAGTACTTGCGCTTCGACAGCGATGTGAGCGAGTTCCGTGCCGTGAATGAGCTGGGGCGGCTGGACGCTGAATACTGGAACAGCAGGAAGGAGCTCCTGGACAACAGGCGGGCCGCGGT----------

>GQ901815_Myodes_glareolus_MHC_class_II_antigen_(Mygl-DRB)_gene_Mygl-DRB*44_allele_exon_2_and_partial_cds

---------------------------------------GAAGAGTACCTGCGCTTCGACAGCGATGTGGGCGAGTTCCGTGCGGTGAATGAGCTGGGGCGGCTGGATGCCAAGTACTGGAACAGCAGGAAGGAGATCCTGGACAACAGGCGGGCCGCGGT----------

>GQ901816_Myodes_glareolus_MHC_class_II_antigen_(Mygl-DRB)_gene_Mygl-DRB*45_allele_exon_2_and_partial_cds

---------------------------------------GAAGAGTACTTGCGCTTCGACAGCGATGTGGGCGAGTTCCGTGCGGTGAATGAGCTGGGGTGGCCCTCAGCCAAGAACTACAACAGCCGGAAGGAGCTCCTGGACAACAGGCGGGCCGCGGT----------

>GQ901817_Myodes_glareolus_MHC_class_II_antigen_(Mygl-DRB)_gene_Mygl-DRB*46_allele_exon_2_and_partial_cds

---------------------------------------GAAGAGTACTTGCGCTTCGACAACGATGTGGGCAAGTTCCGTGCGGTGAATGAGCTGGGGCGGCTGGACGCTGAATACTGGAACAGCAGGAAGGAGATTCTGGACAACAGGCGGACCGCGGT----------

>GQ901818_Myodes_glareolus_MHC_class_II_antigen_(Mygl-DRB)_gene_Mygl-DRB*47_allele_exon_2_and_partial_cds

---------------------------------------GAAGAGTACTTGCGTTTCGACAGCGATGTGGGCGAGTTCCATGCCGTGAATGAGCTGGGGCGGCTGGACGCTGAATACTGGAACAGCAGGAAGGAGATCCTGGACAACAGGCGGGCCGCGGT----------

>GQ901819_Myodes_glareolus_MHC_class_II_antigen_(Mygl-DRB)_gene_Mygl-DRB*48_allele_exon_2_and_partial_cds

---------------------------------------GAGGAGTTCTTGCGCTTCGACAGCGACGTGGGCGAGTACCGCGCGGTGACCGAGCTGGGGCGGCCCTCAGCCGAGTACTACAACAGCCAGAAGGAGCACCTGGAGCAGAAGCGGGCCGAGAC----------

>GQ901820_Myodes_glareolus_MHC_class_II_antigen_(Mygl-DRB)_gene_Mygl-DRB*49_allele_exon_2_and_partial_cds

---------------------------------------GAAGAGTACTTGCGCTTCGACAGCGATGTGGGCGAGTTCCGTGCGGTGAATGAGCTGGGGCGGCTAGACGCTGAATACTGGAACAGCAGGAAGGAGATCCTGGACAACAGGCGGGCCGCGGT----------

>GQ901821_Myodes_glareolus_MHC_class_II_antigen_(Mygl-DRB)_gene_Mygl-DRB*50_allele_exon_2_and_partial_cds

---------------------------------------GAGGAAAACGTGCGCTTCGACAGTGACGTGGGCAAGTACCTTGCTGTGACCAAGCTGGGGCAGCTGGAGGCGGAGAACTGGAACAGCCGGAAGGAGCTCCTGGAGGATGCGCGGGCCGGGGT----------

>GQ901822_Myodes_glareolus_MHC_class_II_antigen_(Mygl-DRB)_gene_Mygl-DRB*51_allele_exon_2_and_partial_cds

---------------------------------------GAGGAGGTCGTGCGCTTCGACAGTGATGTGGGCAAGTATCACGCGGTGACCGAGCTGGGTCGCAGTGATGCTGAGGTCTGGAACAGCCAGAAGGAGGTCCTGGAGGACGCACGGGCCGCGGT----------

>GQ901823_Myodes_glareolus_MHC_class_II_antigen_(Mygl-DRB)_gene_Mygl-DRB*52_allele_exon_2_and_partial_cds

---------------------------------------GAGGAGGTCGTGCGCTTCGACAGTGATGTGGGCGAGTATCACGCGGTGACCGAGCTGGGTCGGAGTGATGCTGAGGTCTGGAACAGCCAGAAGGAGGTCCTGGAGGACGCACTGGCCGCGGT----------

>GQ901824_Myodes_glareolus_MHC_class_II_antigen_(Mygl-DRB)_gene_Mygl-DRB*53_allele_exon_2_and_partial_cds

---------------------------------------GAGGAGTATGCACGCTTTGACAGTGATGTGGGCGAGTACCGCGCAGTGAACGAGCTGGGGCTGCCGGACTCCGAGTACTGGAACAGCCAGGAGGAGCTCCTGGAGCAGAAGCGGTCCCTTGT----------

>GQ901825_Myodes_glareolus_MHC_class_II_antigen_(Mygl-DRB)_gene_Mygl-DRB*54_allele_exon_2_and_partial_cds

---------------------------------------GAGGAGTTCTTGCGCTACGACAGCGACGTGGGCGAGCACCGCGCGGTGACCGAGCTGGGGCGGTCGTGGGCCGAGGACTTCAACAGCCAGAAGGACTTCATGGAGCAGAAGCGGGCTGAGAT----------

>GQ901826_Myodes_glareolus_MHC_class_II_antigen_(Mygl-DRB)_gene_Mygl-DRB*55_allele_exon_2_and_partial_cds

---------------------------------------GAAGAGTACTTGCGCTTCGACAGCGATGTGGGCCAGTTCCGTGCGGTGAATGAGCTGGGCCGGCTGGACGCTGAATACTGGAACAGCAGGAAGGAGATCCTGGACAACAGGCGGGCCGCGGT----------

>GQ901827_Myodes_glareolus_MHC_class_II_antigen_(Mygl-DRB)_gene_Mygl-DRB*56_allele_exon_2_and_partial_cds

---------------------------------------GAAGAGTACTTGCGCTTCGACAGCGATGTGGGCGAGTTCCGTGCGGTGAATGAGCTGGGGCGGCTGGACGCTGAATACTGGAACAGCAGGAAGGAGATCCTGGACAACAGGCAGGGCGCGGT----------

>GQ901828_Myodes_glareolus_MHC_class_II_antigen_(Mygl-DRB)_gene_Mygl-DRB*57_allele_exon_2_and_partial_cds

---------------------------------------GAGGAAAACGTGCGCTTCGACAGTGATGTGGGCAAGTACCTTGCTGTGACCAAGCTGGGGCAGCTGGAGGCGGAGAACTGGAACAGCCGGAAGGAGCTCCTGGAGGACGCATGGGCCGGGGT----------

>GQ901829_Myodes_glareolus_MHC_class_II_antigen_(Mygl-DRB)_gene_Mygl-DRB*58_allele_exon_2_and_partial_cds

---------------------------------------GAGGAGTATGCACGCTTTGACAGTGATGTGGGCAAGTACCGCGCAGTGAACGAGCTGGGGCGGCCTGACTCCCAGTACTGGAACAGCCAGGAGGAGCTCCTGGAGCAGAAGCGGTCCCTTGT----------

>GQ901830_Myodes_glareolus_MHC_class_II_antigen_(Mygl-DRB)_gene_Mygl-DRB*59_allele_exon_2_and_partial_cds

---------------------------------------GAGGAAAACGTGCGCTTCGACAGTGACGTGGGCAAGTACCTTGCTGTGACCAAGCTGGGGCGGCTGGAGGCGGAGAACTGGAACAGTCGAAAGGAGCTCCTGGAGGACGCAAGGGCCGGGGT----------

>GQ901831_Myodes_glareolus_MHC_class_II_antigen_(Mygl-DRB)_gene_Mygl-DRB*60_allele_exon_2_and_partial_cds

---------------------------------------GAAGAGTACTTGCGCTTCGACAGCGATGTGGGCGAGTTCCGTGCCGTGAATGAGCTGGGGCGGCTGGACGCTGAATACTGGAACAGCAGGAAGGAGCTCCTGGACAACAGGCGGGCCGCGGT----------

>GQ901832_Myodes_glareolus_MHC_class_II_antigen_(Mygl-DRB)_gene_Mygl-DRB*61_allele_exon_2_and_partial_cds

---------------------------------------GAAGAGTACCTGCGCTTCGACAGCGATGTGGGCGAGTTCCGTGCGGTGAATGAGCTGGGGCGGCTGGACGCTGAATACTGGAACAGCAGGAAGGAGATCCTGGACAACAGGCGGGCCGCGGT----------

>GQ901833_Myodes_glareolus_MHC_class_II_antigen_(Mygl-DRB)_gene_Mygl-DRB*62_allele_exon_2_and_partial_cds

---------------------------------------GAAGAGTACTTGCGCTTCGACAGCGATGTGGGCGAGTTCCGTGCCGTGAATGAGCTGGGGCGGCTGGACGCTGAATACTGGAACAGCAGGAAGGAGATCCTGGACAACAGGCGGGCCGCGGT----------

>GQ901834_Myodes_glareolus_MHC_class_II_antigen_(Mygl-DRB)_gene_Mygl-DRB*63_allele_exon_2_and_partial_cds

---------------------------------------GAAGAGTACTTGCGTTTCGACAACGATGTGGGCGAGTTCCATGCCGTGAATGAGCTGGGGCGGCTGGACGCTGAATACTGGAACAGCAGGAAGGAGATCCTGGACAACAGGCGGGCCGCGGT----------

>GQ901835_Myodes_glareolus_MHC_class_II_antigen_(Mygl-DRB)_gene_Mygl-DRB*64_allele_exon_2_and_partial_cds

---------------------------------------GAAGAGTACCTGCGCTTCGACAGCGATGTGGGCGAGTTCCGTGCCGTGAATGAGCTGGGGCGGCTGGATGCCAAGTACTGGAACAGCAGGAAGGAGATCCTGGACAACAGGCGGGCCGCGGT----------

>GQ901836_Myodes_glareolus_MHC_class_II_antigen_(Mygl-DRB)_gene_Mygl-DRB*65_allele_exon_2_and_partial_cds

---------------------------------------GAGGAGTACGCAGGCTTTGACAGTGATGTGGGCGAGTACCGCGCAGTGAACGAGCTGGGGCTGCCGGACTCCGAGTACTGGAACAGCCAGGAGGAGCTCCTGGAGCAGAAGCGGTCCCTTGT----------

>GQ901837_Myodes_glareolus_MHC_class_II_antigen_(Mygl-DRB)_gene_Mygl-DRB*66_allele_exon_2_and_partial_cds

---------------------------------------GAGGAGTTCGTGCGCTACGACAGCGACGTGGGCAAGTTCATTGCGGTGACCGAGCTGGGGCGGTCGTGGGCCGAGTACTTCAACAGCCAGAAGGACATCCTGGAGCGGAAGCGGGCTGAGAT----------

>GQ901838_Myodes_glareolus_MHC_class_II_antigen_(Mygl-DRB)_gene_Mygl-DRB*67_allele_exon_2_and_partial_cds

---------------------------------------GAAGAGTACTTGCGCTTCGACAGCGATGTGGGCGAGTTCCGTGCGGTGAATGAGCTGGGCCGGCTGGACGCTGAATACTGGAACAGCAGGAAGGAGATCCTGGACAACAGGCGGGCCGCGGT----------

>GQ901839_Myodes_glareolus_MHC_class_II_antigen_(Mygl-DRB)_gene_Mygl-DRB*68_allele_exon_2_and_partial_cds

---------------------------------------GAGGAGTATGCACGCTTTGACAGTGATGTGGGCGAGTACCGCGCAGTGAACGAGCTGGGGCTGCCGGACTCCGAGTACTGGAACAGCCAGGAGGAGCTCCTGGAGCAGAAGCAGTCCCTTGT----------

>GQ901840_Myodes_glareolus_MHC_class_II_antigen_(Mygl-DRB)_gene_Mygl-DRB*69_allele_exon_2_and_partial_cds

---------------------------------------GAGGAGCATGTGCGCTTCGACAGCGACGTGGGCGAGTTCCGCGCGGTGACCGAGCTGGGGCGGCCAGACGCCGAGTACTGGAACAGCCAGAAGGACATCCTGGAGCGGAAGCGGGCCGAGAC----------

>GQ901841_Myodes_glareolus_MHC_class_II_antigen_(Mygl-DRB)_gene_Mygl-DRB*70_allele_exon_2_and_partial_cds

---------------------------------------GAGGAGTTCGTGCGCTTCGACAGCGATGTGGGCGAGTTCCGCGCGGTGACCGAGCTGGGGCGGTCGTGGGCCGAGTACTTCAACAGCCAGAAGGACTATCTGGAGCAGAAGCGGGCCGAGAC----------

>GQ901842_Myodes_glareolus_MHC_class_II_antigen_(Mygl-DRB)_gene_Mygl-DRB*71_allele_exon_2_and_partial_cds

---------------------------------------GAAGAGTACTTGCGCTTCGACAGCGATGTGGGCGAGTTCCGTGCGGTGAATGAGCTGGGGCGGCCCTCAGCCAAGAACTACAACAGCCGGAAGGAGCTCCTGGACAACAGGCGGGCCGCGGT----------

>GQ901843_Myodes_glareolus_MHC_class_II_antigen_(Mygl-DRB)_gene_Mygl-DRB*72_allele_exon_2_and_partial_cds

---------------------------------------GAAGAGTACTTGCGTTTCGACAGCGATGTGGGCGAGTTCCATGCCGTGAATGAGCTGGGGCGGCTGGACGCTGAATACTGGAACAGCAGGAAGGAGATCCTGGACAACAGGCGGGCCGCAGT----------

>GQ901844_Myodes_glareolus_MHC_class_II_antigen_(Mygl-DRB)_gene_Mygl-DRB*73_allele_exon_2_and_partial_cds

---------------------------------------GAAGAGTACCTGCGCTTCGACAGCGATGTGGGCGAGCTCCGTGCGGTGAATGAGCTGGGGCGGCTGGATGCCAAGTACTGGAACAGCAGGAAGGAGATCCTGGACAACAGGCGGGCCGCGGT----------

>GQ901845_Myodes_glareolus_MHC_class_II_antigen_(Mygl-DRB)_gene_Mygl-DRB*74_allele_exon_2_and_partial_cds

---------------------------------------GAGGAGTTCTTGCGCTTCGACAGCGACATGGGCGAGTACCGCGCGGTGACCGAGCTGGGGCGGCCCTCAGCCGAGTACTACAACAGCCAGAAGGAGCACCTGGAGCAGAAGCGGGCCGAGAC----------

>GQ901846_Myodes_glareolus_MHC_class_II_antigen_(Mygl-DRB)_gene_Mygl-DRB*75_allele_exon_2_and_partial_cds

---------------------------------------GAGGAGTTCGTGCGCTACGACAGCAATGTGGGCGAGCACCGCGCGGTGACCGATTGGGGGCGATCGTGGGCCGAGGACTTCAACAGCCAGAAGGACTTCATGGAGCAGAAGCGGGCCGAGGT----------

>GQ901847_Myodes_glareolus_MHC_class_II_antigen_(Mygl-DRB)_gene_Mygl-DRB*76_allele_exon_2_and_partial_cds

---------------------------------------GAGGAGTTCTTGCGCTACGACAGCGACGTGGGCGAGCACCGCGCGGTGACCGAGCTGGGGCGGTCGTGGGCCGAGGACTTCAACAGCCAGAAGGACTATATGGAGCAGAAGCGGGCTGAGAT----------

>GQ901848_Myodes_glareolus_MHC_class_II_antigen_(Mygl-DRB)_gene_Mygl-DRB*77_allele_exon_2_and_partial_cds

---------------------------------------GAAGAGTACCTGCGCTTCGACAGCGATGTGGGCGAGTTCCGTGCGGTGAATGAGCTGGGGCGGCCCTCAGCCAAGAACTACAACAGCCTGAAGGAGCTCCTGGACAACAGGCGGGCCGCGGT----------

>GQ901849_Myodes_glareolus_MHC_class_II_antigen_(Mygl-DRB)_gene_Mygl-DRB*78_allele_exon_2_and_partial_cds

---------------------------------------GAAGAGTACCTGCGCTTCGACAGCGATGTGGGCGAGTTCCGTGCGGTGAATGAGCTGGGGCGGCTGGATGCTGAATACTGGAACAGCAGGAAGGAGATCCTGGACAACAGGCGGGCCGCGGT----------

>GQ901850_Myodes_glareolus_MHC_class_II_antigen_(Mygl-DRB)_gene_Mygl-DRB*79_allele_exon_2_and_partial_cds

---------------------------------------GAAGAGTACCTGCGCTTCGACAGCGATGTGGGCGAGTTCCGTGCGGTGAATGAGCTGGGGCGGCTGGATGCCAAGTACTGGAACAGCAGGAAGGAGATCCTAGACAACAGGCGGGCCGCGGT----------

>GQ901851_Myodes_glareolus_MHC_class_II_antigen_(Mygl-DRB)_gene_Mygl-DRB*80_allele_exon_2_and_partial_cds

---------------------------------------GAAGAGGTCGTGCGCTTCGACAGTGATGTGGGCAAGTATCACGCGGTGACCGAGTTGGGTCGGAGTGATGCTGAGGTCTGGAACAGCCAGAAGGAGGTCCTGGAGGACGCACGGGCCGCGGT----------

>GQ901852_Myodes_glareolus_MHC_class_II_antigen_(Mygl-DRB)_gene_Mygl-DRB*81_allele_exon_2_and_partial_cds

---------------------------------------GAGGAGGTCGTGCGCTTCGACAGTGATGTGGGCGAGTATCATGCGGTGACTGAGCTGGGTCGCAGTGATGCTGAGGTCTGGAACAGCCAGAAGGAGGTCCTGGAGGACGCACAGGCCGCGGT----------

>GQ901853_Myodes_glareolus_MHC_class_II_antigen_(Mygl-DRB)_gene_Mygl-DRB*82_allele_exon_2_and_partial_cds

---------------------------------------GAGGAGTATGCAGGCTTTGACAGTGATGTGGGCGAGTACCGCGCAGTGAACGAGCTGGGGCTGCCGGACTCCGAGTACTGGAACAGCCAGGAGGAGCTCCTGGAGCAGAAGCGGTCCCTTGT----------

>GQ901854_Myodes_glareolus_MHC_class_II_antigen_(Mygl-DRB)_gene_Mygl-DRB*83_allele_exon_2_and_partial_cds

---------------------------------------GAGGAAAACGTGCGCTTCGACAGTGATGTGGGCAAGTACCTTGCTGTGACCAAGCTGGGGCAGCTGGAGGCGGAGAACTGGAACAGCCGGAAGGAGCTCCTGGAGGACGCACGGACCGGGGT----------

>GQ901855_Myodes_glareolus_MHC_class_II_antigen_(Mygl-DRB)_gene_Mygl-DRB*84_allele_exon_2_and_partial_cds

---------------------------------------GAGGAGGTCGTGCGCTTCGACAGTGATGTGGGAGAGTATCACGCGGTGACCGAGCTGGGTCGCAGTGATGCTGAGGTCTGGAACAGCCAGAAGGAGGTCCTGGAGGACGCACGGGCCGCGGT----------

>GQ901856_Myodes_glareolus_MHC_class_II_antigen_(Mygl-DRB)_gene_Mygl-DRB*85_allele_exon_2_and_partial_cds

---------------------------------------GAGGAGGTCGTGCGCTTCGACAGTGATGTGGGCGAGTATCACGCGGTGACCGAGCTGGGTCGCAGTGATGCTGAGGTCTGGAACAGCCAGAAGGAGGTCCTGGAGGATGCACGGGCCGCGGT----------

>GQ901857_Myodes_glareolus_MHC_class_II_antigen_(Mygl-DRB)_gene_Mygl-DRB*86_allele_exon_2_and_partial_cds

---------------------------------------GAAGAGTACTTGCGCTTCGACAGCGATGTGGGCGAGTTCCGTGCAGTGAATGAGCTGGGGTGGCCCTCAGCCAAGAACTACAACAGCCGGAAGGAGCTCCTGGACAACAGGCGGGCCGCGGT----------

>GQ901858_Myodes_glareolus_MHC_class_II_antigen_(Mygl-DRB)_gene_Mygl-DRB*87_allele_exon_2_and_partial_cds

---------------------------------------GAAGAGTACCTGCGCTTCGACAGCGATGTGGGCAAGTTCCGTGCGGTGAATGAGCTGGGGCGGCTGGACGCTGAATACTGGAACAGCAGGAAGGAGATCCTGGACAACAGGCGGGCCGCGGT----------

>GQ901859_Myodes_glareolus_MHC_class_II_antigen_(Mygl-DRB)_gene_Mygl-DRB*88_allele_exon_2_and_partial_cds

---------------------------------------GAGGAGTTCGTGCGCTACGACAGCAATGTGGGCGAGTTCATCGCGGTGACCGAGATGGGGCGTCGGCAGGCCGAGTACTGGAACAGCCAGAAGGACATCCTGGAGCAGAAGCGGGCCGAGAC----------

>GQ901860_Myodes_glareolus_MHC_class_II_antigen_(Mygl-DRB)_gene_Mygl-DRB*89_allele_exon_2_and_partial_cds

---------------------------------------GAGGAGTACGCACGCTTTGACAGTGATGTGGGCGAGTACCGTGCGGTGACCGAGCTGGGTCGGAGAGATGCTGAGGTCTGGAACAGCCAGAAGGAGCTCCTGGACAACAGGCGGGCCTATGT----------

>GU332030_Rhabdomys_pumilio_MHC_class_II_antigen_(Rhpu-DRB)_gene_Rhpu-DRB*21_allele_exon_2_and_partial_cds

CAGCACGTGAGGTTTCTGGAGAGACACATCTACAACCGGGAGGAGTTCATGCGCTTCGACAGCGACGTGGGCGAGTACCGCACTGTGACCGAGCTGGGGCGGGGCATAGCTGAGGACTGGAACAGCCAGAAGGAGTACCTGGAGCAGAAGCGGGCCCAGGTGGACGCGTAC

>GU332031_Rhabdomys_pumilio_MHC_class_II_antigen_(Rhpu-DRB)_gene_Rhpu-DRB*22_allele_exon_2_and_partial_cds

CAGCGCGTGCGGTTTCTGGACAGATACTTCTACAACCGGGAGGAGTACGCGCGCTTCGACAGCGACGTGGGCGAGTACCGCGCGGTGACCGAGCTGGGGCGGCGCACAGCCAAGTACTGGAACAGCCAGAAGGAGGTCCTGGAGGATGCGCGGGCCGCGGTGGACACGTAC

>GU332032_Rhabdomys_pumilio_MHC_class_II_antigen_(Rhpu-DRB)_gene_Rhpu-DRB*23_allele_exon_2_and_partial_cds

CAGCACGTGAGGTTTCTGGAGAGACACATCTACAACCGGGAGGAGTTCATGCGCTTCGACAGCGACGTGGGCGAGTACCGCACTGTGACCGAGCTGGGGCGGCGCATAGCTGAGGACTGGAACAGCCAGAAGGAGTACCTGGAGGATCGGCGGGCCCAGGTGGACGCGTAC

>GU332033_Rhabdomys_pumilio_MHC_class_II_antigen_(Rhpu-DRB)_gene_Rhpu-DRB*24_allele_exon_2_and_partial_cds

CAGCGCGTGCGGTTTCTGGACAGATACATCCACAACCGGGAGGAGTTCGTGCGCTTCGACAGCGACGTGGGCGAGTACCGCGCGGTGACCGAGCTGGGGCGGCGCACAGCCAAGTACTGGAACAGCCAGAAGGAGGTCCTGGAGGATGCGCGGGCCGCGGTGGACACGTAC

>GU332034_Rhabdomys_pumilio_MHC_class_II_antigen_(Rhpu-DRB)_gene_Rhpu-DRB*25_allele_exon_2_and_partial_cds

CAGCTCGTGCGGTTTCTGGAGAGATACTTCTACAACCGGGAGGAGTACGCGCGCTTCGACAGCGACGTGGGCGAGTACCGCGCGGTGACCGAGCTGGGGCGGCGGGACGCTGAGTACTGGAACAGCCAGAAGGAGGAACTGGAGCAGAAGCGGGCCCAGGTGGACGCGTAC

>GU332035_Rhabdomys_pumilio_MHC_class_II_antigen_(Rhpu-DRB)_gene_Rhpu-DRB*26_allele_exon_2_and_partial_cds

CAGCGCGTGCGGTTTCTGGACAGATACTTCTACAACCGGGAGGAGTACGCGCGCTTCGACAGCGACGTGGGCGAGTACCGCGCGGTGACCGAGCTGGGGCGGCGCACAGCCAAGTACTGGAACAGCCAGAAGGAGCTCCTGGAGCAGAAGCGGGCCGCGGTGGACACGTAC

>GU332036_Rhabdomys_pumilio_MHC_class_II_antigen_(Rhpu-DRB)_gene_Rhpu-DRB*27_allele_exon_2_and_partial_cds

CAGCGCGTGCGGTTTCTGGAGAGATACTTCTACAACCGGGAGGAGTACGCGCGCTTCGACAGCGACGTGGGCGAGTACCGCGCGGTGACCGAGCTGGGGCGGCGGGACGCTGAGCACTGGAACAGCCAGAAGGAGATCCTGGAGGATGCGCGGGCCGCGGTGGACACGTAC

>GU332037_Rhabdomys_pumilio_MHC_class_II_antigen_(Rhpu-DRB)_gene_Rhpu-DRB*28_allele_exon_2_and_partial_cds

CAGCGCGTGCGGTTTCTGGACAGATACTTCTACAACCGGGAGGAGTACGCGCGCTTCGACAGCGACGTGGGCGAGTTCCGCGCGGTGACCGAGCTGGGGCGGCCGGACGCTGAGTACTGGAACAGCCAGAAGGAGATCCTGGAGGATGCGCGGGCCGCGGTGGACACGTAC

>GU332038_Rhabdomys_pumilio_MHC_class_II_antigen_(Rhpu-DRB)_gene_Rhpu-DRB*29_allele_exon_2_and_partial_cds

CAGCGCGTGCGGTTTCTGATCAGATACTTCTACAACCGGGAGGAGTACGTGCGCTTCGACAGCGACGTGGGCGAGCACCGCGCGGTGACCGAGCTGGGGCGGCGGGACGCTGATTACTGGAACAGCCAGAAGGAGCTCCTGGAGCAGAGGCGGGCCCAGGTGGACACGTAC

>GU332039_Rhabdomys_pumilio_MHC_class_II_antigen_(Rhpu-DRB)_gene_Rhpu-DRB*30_allele_exon_2_and_partial_cds

CAGCGCGTGCGGTTTCTGGACAGATACTTCTACAACCGGGAGGAGTACGTGCGCTTCGACAGCGACGTGGGCGAGTACCGCGCGGTGACCGAGCTGGGGCGGCCGGACGCTGATTACTGGAACAGCCAGAAGGAGCTCCTGGAGCAGAGGCGGGCCCAGGTGGACACGTAC

>GU332040_Rhabdomys_pumilio_MHC_class_II_antigen_(Rhpu-DRB)_gene_Rhpu-DRB*31_allele_exon_2_and_partial_cds

CAGCGCGTGCGGTTTCTGGACAGATACTTCTACAACCGGGAGGAGTTCGTGCGCTTCGACAGCGACGTGGGCGAGTACCGCACTGTGACCGAGCTGGGGCGGGGCATAGCTGAGGACTGGAACAGCCAGAAGGAGTTCCTGGAGCAGAAGCGGGCCCGGGTGGACACGTAC

>GU332041_Rhabdomys_pumilio_MHC_class_II_antigen_(Rhpu-DRB)_gene_Rhpu-DRB*32_allele_exon_2_and_partial_cds

CAGCGCGTGCGGTTTCTGGAGAGATACTTCTACAACCGGGAGGAGTACGCGCGCTTCGACAGCGACGTGGGCGAGTACCGCGCAGTGACCGAGCTGGGGCGGCGCACAGCCAAGTACTGGAACAGCCAGAAGGAGATCCTGGAGCGGAAGCGGGCCCAGGTGGACGCGTAC

>GU332042_Rhabdomys_pumilio_MHC_class_II_antigen_(Rhpu-DRB)_gene_Rhpu-DRB*33_allele_exon_2_and_partial_cds

CAGCGCGTGCGGTTTCTGGAGAGATACTTCTACAACCGGGAGGAGTACGCGCGCTTCGACAGCGACGTGGGCGAGTACCGCGCGGTGACCGAGCTGGGGCGGCGCACAGCCAAGTACTGGAACAGCCAGAAGGAGCTCCTGGAGCGGAAGCGGGCCCAGGTGGACGCGTAC

>GU332043_Rhabdomys_pumilio_MHC_class_II_antigen_(Rhpu-DRB)_gene_Rhpu-DRB*34_allele_exon_2_and_partial_cds

CAGCGCGTGCGGTTTCTGGAGAGATACTTCTACAACCGGGAGGAGTACGCGCGCTTCGACAGCGACGTGGGCGAGTACCGCGCGGTGACCGAGCTGGGGCGGCGCACAGCCAAGTATTGGAACAGCCAGAAGGAGGTCCTGGAGGATGCGCGGGCCGCGGTGGACACGTAC

>GU332044_Rhabdomys_pumilio_MHC_class_II_antigen_(Rhpu-DRB)_gene_Rhpu-DRB*35_allele_exon_2_and_partial_cds

CAGCTCGTGCGGTTTCTGGAGAGATACTTCTACAACCGGGAGGAGTACGCGCGCTTCGACAGCGACGTGGGCGAGTACCGCGCGGTGACCGAGCTGGGGCGGCGGGACGCTGATTACTGGAACAGCCAGAAGGAGGAACTGGAGCAGAAGCGGGCCCAGGTGGACGCGTAC

>GU332045_Rhabdomys_pumilio_MHC_class_II_antigen_(Rhpu-DRB)_gene_Rhpu-DRB*36_allele_exon_2_and_partial_cds

CAGCGCGTGCGGTTTCTGGAGAGACACATCTACAACCGGGAGGAGTTCATGCGCTTCGACAGCGACGTGGGCGAGTACCGCGCGGTGATCGAGCTGGGGCGGCGCATAGCTGAGGACTTCAACAGCCGGAAGGAGATCCTGGAGGATGCGCGGGCCCAGGTGGACACGTAC

>GU332046_Rhabdomys_pumilio_MHC_class_II_antigen_(Rhpu-DRB)_gene_Rhpu-DRB*37_allele_exon_2_and_partial_cds

CAGCGCGTGCGGTTTCTGGACAGATACATCCACAACCGGGAGGAGTTCGTGCGCTTCGACAGCGACGTGGGCGAGCACCGCGCGGTGACCGAGCTGGGGCGGCGCATAGCTGAGGACTTGAACAGCCAGAAGGAGCTCCTGGAGCGGAAGCGGGCCGAGCTGGACACGTAC

>GU332047_Rhabdomys_pumilio_MHC_class_II_antigen_(Rhpu-DRB)_gene_Rhpu-DRB*38_allele_exon_2_and_partial_cds

CAGCGCGTGCGGTTTCTGGACAGATACATCCACAACCGGGAGGAGTTCGTGCGCTTCGACAGCGACGTGGGCGAGCACCGCGCGGTGACCGAGCTGGGGCGGCGCATAGCTGAGGACTGGAACAGCCAGAAGGAGCTCCTGGAGCGGAAGCGGGCCGAGCTGGACACGTAC

>GU332048_Rhabdomys_pumilio_MHC_class_II_antigen_(Rhpu-DRB)_gene_Rhpu-DRB*39_allele_exon_2_and_partial_cds

CAGCGCGTGCGGTTTCTGGACAGACACATCTACAACCGGGAGGAGTTCATGCGCTTCGACAGCGACGTGGGCGAGTACCGCACTGTGACCGAGCTGGGGCGGCGCATAGCTGAGGACTGGAACAGCCAGAAGGAGCTCCTGGAGCAGAAGCGGGCCCAGGTGGACGCGTAC

>GU332049_Rhabdomys_pumilio_MHC_class_II_antigen_(Rhpu-DRB)_gene_Rhpu-DRB*40_allele_exon_2_and_partial_cds

CAGCGCGTGCGGTTTCTGGACAGATACATCCACAACCGGGAGGAGTTCGTGCGCTTCGACAGCGACGTGGGCGAGCACCGCGCGGTGACCGAGCTGGGGCGGCGCACAGCTGAGGACTTGAACAGCCAGAAGGAGCTCCTGGAGCGGAAGCGGGCCGAGGTGGACACGTAC

>GU332050_Rhabdomys_pumilio_MHC_class_II_antigen_(Rhpu-DRB)_gene_Rhpu-DRB*41_allele_exon_2_and_partial_cds

CAGCGCGTGCGGTTTCTGGAGAGATACTTCTACAACCGGGAGGAGTACGCGCGCTTCGACAGCGACGTGGGCGAGTACCGCGCGGTGACCGAGCTGGGGCGGCGCACAGCCAAGTACTGGAACAGCCAGAAGGAGGTCCTGGAGGATGCGCGGGCCGCGGTGGACACGTAC

>GU332051_Rhabdomys_pumilio_MHC_class_II_antigen_(Rhpu-DRB)_gene_Rhpu-DRB*42_allele_exon_2_and_partial_cds

CAGCGCGTGCGGTTTCTGGAGAGACACATCTACAACCGGGAGGAGTTCATGCGCTTCGACAGCGACGTGGGCGAGTACCGCGCGGTGACCGAGCTGGGGCGGCGCATAGCTGAGGACTTCAACAGCCGGAAGGAGCTCCTGGAGCGGAAGCGGGCCGCGGTGGACACGTAC

>GU332052_Rhabdomys_pumilio_MHC_class_II_antigen_(Rhpu-DRB)_gene_Rhpu-DRB*43_allele_exon_2_and_partial_cds

CAGCGCGTGCGGTTTCTGATCAGATACTTCTACAACCGGGAGGAGTACGCGCGCTTCGACAGCGACGTGGGCGAGTACCGCGCGGTGACCGAGCTGGGGCGGCGGGACGCTGAGTATTGGAACAGCCAGAAGGAGCTCCTGGAGCATACGCGGGCCGCGGTGGACACGTAC

>GU332053_Rhabdomys_pumilio_MHC_class_II_antigen_(Rhpu-DRB)_gene_Rhpu-DRB*44_allele_exon_2_and_partial_cds

CAGCGCGTGCGGTTTCTGGAGAGATACTTCTACAACCGGGAGGAGTACGCGTGCTTCGACAGCGACGTGGGCGAGTACCGCGCGGTGACCGAGCTGGGGCGGCGGGACGCCGAGTACTGGAACAGCCAGAAGGAGCTCCTGGAGCAGAAGCGGGCCCAGGTGGACGCGTAC

>GU332054_Rhabdomys_pumilio_MHC_class_II_antigen_(Rhpu-DRB)_gene_Rhpu-DRB*45_allele_exon_2_and_partial_cds

CAGCGCGTGCGGTTTCTGGAGAGATACTTCTACAACCGGGAGGAGTACGCGCGCTTCGACAGCGACGTGGGCGAGTACCGCGCGGTGACCGAGCTGGGGCGGCGCACAGCCAAGTACTGGAACAGCCAGAAGGAGATCCTGGAGCGGAAGCGGGCCCAGCTGGACGCGTAC

>GU332055_Rhabdomys_pumilio_MHC_class_II_antigen_(Rhpu-DRB)_gene_Rhpu-DRB*46_allele_exon_2_and_partial_cds

CAGCGCGTGCGGTTTCTGGAGAGATACTTCTACAACCGGGAGGAGTACGCGCGCTTCGACAGCGACGTGGGCGAGTACCGCGCGGTGACCGAGCTGGGGCGGCGCACAGCCAAGTATTGGAACAGCCAGAAGGAGGTCCTGGAGGATGCGCGGGCCCCGGTGGACACGTAC

>GU332056_Rhabdomys_pumilio_MHC_class_II_antigen_(Rhpu-DRB)_gene_Rhpu-DRB*47_allele_exon_2_and_partial_cds

CAGCGCGTGCGGTATCTGCAGAGATATTTCTACAACCGTCAGGAGTACGTGCGCTTCGACAGCGACGTGGGCGAGTTCCGCGCGGTGACCGAGCTGGGGCGGCCGGACGAGGAATACTGGAACAGCCAGAAGGACTTCGTGGAGCAGAAGCGGTCACAGGTGGACGCATAC

>GU332057_Rhabdomys_pumilio_MHC_class_II_antigen_(Rhpu-DRB)_gene_Rhpu-DRB*48_allele_exon_2_and_partial_cds

CAGCACGTGCGGTTTCTGGTCAGATTCTTCTACAACCGGCAGGAGTACATGCGCTTCGACAGCGACGTGGGCGAGTTCCGCGCGGTGACCGAGCTGGGGCGGCGCGATGAGGAATACTGGAACAGCCAGAAGGAGATCCTGGAGCATAAGCGGGCCGAGGTGGACACGTAC

>GU332058_Rhabdomys_pumilio_MHC_class_II_antigen_(Rhpu-DRB)_gene_Rhpu-DRB*49_allele_exon_2_and_partial_cds

CAGCACGTGCGGCTTCTGGACAGATACATCTACAACCGGGAGGAGTACGTGCGCTTCGACAGCGACGTGGGCGAGTACCGCGCGGTGACCGAGCTGGGGCGGGGCATAGCTGAGCACTGGAACAGCCAGAAGGAGTTCCTGGAGCAGAGGCGGGCCGCGGTGGACACGTAC

>GU332059_Rhabdomys_pumilio_MHC_class_II_antigen_(Rhpu-DRB)_gene_Rhpu-DRB*50_allele_exon_2_and_partial_cds

CAGCGCGTGCGGTTTCTGGACAGATACATCCACAACTGGGAGGAGAACGTGCGCTTCGACAGCGACGTGGGCGAGTACCGCGCGGTGACCGAGCTGGGGCGGCCGGACGCTGAGCACTGGAACAGCCAGAAGGAGATCCTGGAGCAGAAGCGGGCCCGGGTGGACACGTAC

>GU332060_Rhabdomys_pumilio_MHC_class_II_antigen_(Rhpu-DRB)_gene_Rhpu-DRB*51_allele_exon_2_and_partial_cds

CAGCGCGTGCGGTTTCTGGACAGATACTTCTACAACCGGGAGGAGTTCGTGCGCTTCGACAGCGACGTGGGCGAGTACCGCGCGGTGACCGAGCTGGGGCGGCGGGACGCTGAGCACTGGAACAGCCAGAAGGAGCTCCTGGAGCAGAAGCGGGCCCGGGTGGACACGTAC

>GU332061_Rhabdomys_pumilio_MHC_class_II_antigen_(Rhpu-DRB)_gene_Rhpu-DRB*52_allele_exon_2_and_partial_cds

CAGCGCGTGCGGTTTCTGGACAGATACTTCTACAACCGGGAGGAGTACGCGCGCTTCGACAGCGACGTGGGCGAGTACCGCGCGGTGACGGAGTTGGGGCGGCCGGACGCTGAGTATTGGAACAGCCAGAAGGAGATCCTGGAGGATCGGCGGGCCCAGGTGGACACGTAC

>GU332062_Rhabdomys_pumilio_MHC_class_II_antigen_(Rhpu-DRB)_gene_Rhpu-DRB*53_allele_exon_2_and_partial_cds

CAGCGCGTGCGGTTTCTGGACAGATACATCCACAACCGGGAGGAGAACGTGCGCTTCGACAGCGACGTGGGCGAGTACCGCGCGGTGACCGAGCTGGGGCGGCCGGACGCTGAGGACTGGAACAGCCAGAAGGAGCTCCTGGAGGATCGGCGGGCCCAGGTGGACACGTAC

>GU332063_Rhabdomys_pumilio_MHC_class_II_antigen_(Rhpu-DRB)_gene_Rhpu-DRB*54_allele_exon_2_and_partial_cds

CAGCGCGTGCGGTTTCTGGAGAGACACATCTACAACCGGGAGGAGTTCATGCGCTTCGACAGCGACGTGGGCGAGTACCGCGCGGTGACCGAGCTGGGGCGGCGCATAGCTGAGGACTTCAACAGCCGGAAGGAGCTCCTGGAGCGGAAGCGGGCCGAGCTGGACACGTAC

>GU332064_Rhabdomys_pumilio_MHC_class_II_antigen_(Rhpu-DRB)_gene_Rhpu-DRB*55_allele_exon_2_and_partial_cds

CAGCACGTGCGGCTTCTGGACAGATACTTCTACAACCGGGAGGAGTTCGTGCGCTTCGACAGCGACGTGGGCGAGTACCGCGCGGTGACCGAGCTGGGGCGGCGGGACGCCGAGCACTGGAACAGCCAGAAGGAGTTCCTGGAGCAGAGGCGGGCCGCGGTGGACACGTAC

>GU332065_Rhabdomys_pumilio_MHC_class_II_antigen_(Rhpu-DRB)_gene_Rhpu-DRB*56_allele_exon_2_and_partial_cds

CAGCGCGTGCGGTTTCTGTTCAGAGACATCTACAACCAGGAGGAGCATGTGCGCTTCGACAGCGACGTGGGCGAGTACCGCGCGGTGACCGAGCTGGGGCGGCCGGACGCTGAGTACTGGAACAGCCAGAAGGAGATCCTGGAGGATGCGCGGGCCGCGGTGGACACGTAC

>GU332066_Rhabdomys_pumilio_MHC_class_II_antigen_(Rhpu-DRB)_gene_Rhpu-DRB*57_allele_exon_2_and_partial_cds

CAGCGCGTGCGGTTTCTGGACAGATACATCCACAACCGGGAGGAGTTCGTGCGCTTCGACAGCGACGTGGGCGAGTTCCGCGCGGTGACCGAGCTGGGGCGGCGCATAGCTGAGGACTTGAACAGCCAGAAGGAGCTCCTGGAGCAGAAGCGGGCCGCGGTGGACACGTAC

>GU332067_Rhabdomys_pumilio_MHC_class_II_antigen_(Rhpu-DRB)_gene_Rhpu-DRB*58_allele_exon_2_and_partial_cds

CAGCACGTGCGGCTTCTGGACAGATACTTCTACAACCGGGAGGAGTTCGTGCGCTTCGACAGCGACGTGGGCGAGTACCGCGCGGTGACCGAGCTGGGGCGGGGCATAGCTGAGGACTGGAACAGCCAGAAGGAGTTCCTGGAGCAGAGGCGGGCCGCGGTGGACACGTAC

>GU332068_Rhabdomys_pumilio_MHC_class_II_antigen_(Rhpu-DRB)_gene_Rhpu-DRB*59_allele_exon_2_and_partial_cds

CAGCACGTGCGGTTTCTGGAGAGACACATCTACAACCGGGAGGAGTTCATGCGCTTCGACAGCGACGTGGGCGAGTACCGCGCGGTGACCGAGCTGGGGCGGCGCACAGCCAAGTACTGGAACAGCCAGAAGGAGTTCCTGGAGCGGAAGCGGGCCCAGGTGGACACGTAC

>GU332069_Rhabdomys_pumilio_MHC_class_II_antigen_(Rhpu-DRB)_gene_Rhpu-DRB*60_allele_exon_2_and_partial_cds

CAGCGCGTGCGGTTTCTGGACAGATACATCCACAACCGGGAGGAGTTCGTGCGCTTCGACAGCGACGTGGGCGAGCACCGCGCGGTGACCGAGCTGGGGCGGCGCACAGCCAAGTACTGGAACAGCCAGAAGGAGCTCCTGGAGCAGAAGCGGGCCCAGGTGGACGCGTAC

>GU332070_Rhabdomys_pumilio_MHC_class_II_antigen_(Rhpu-DRB)_gene_Rhpu-DRB*61_allele_exon_2_and_partial_cds

CAGCGCGTGCGGTTTCTGGACAGATACTTCTACAACCGGGAGGAGTACGCGCGCTTCGACAGCGACGTGGGCGAGTACCGCGCGGTGACCGAGCTGGGGCGGCGCACAGCCAAGTACTGGAACAGCCAGAAGGAGATCCTGGAGGATGCGCGGGCCGCGGTGGACACGTAC

>GU332071_Rhabdomys_pumilio_MHC_class_II_antigen_(Rhpu-DRB)_gene_Rhpu-DRB*62_allele_exon_2_and_partial_cds

CAGCACGTGCGGTTTCTGGACAGATACTTCTACAACCGGGAGGAGTACGCGCGCTTCGACAGCTACGTGGGCGAGTTCCGCGCGGTGACCGAGCTGGGGCGGCGCACAGCCAAGTACTGGAACAGCCAGAAGGAGCTCCTGGAGGATCGGCGGGCCCAGGTGGACGCGTAC

>GU332072_Rhabdomys_pumilio_MHC_class_II_antigen_(Rhpu-DRB)_gene_Rhpu-DRB*63_allele_exon_2_and_partial_cds

CAGCGCGTGCGGTTTCTGGAGAGACACATCTACAACCGGGAGGAGTACGCGCGCTTCGACAGCGACGTGGGCGAGTACCGCGCGGTGACCGAGCTGGGGCGGCGCATAGCTGAGGACTGGAACAGCCGGAAGGAGATCCTGGAGGATGCGCGGGCCGAGGTGGACACATAC

>GU332073_Rhabdomys_pumilio_MHC_class_II_antigen_(Rhpu-DRB)_gene_Rhpu-DRB*64_allele_exon_2_and_partial_cds

CAGCGCGTGCGGTTTCTGGAGAGATACTTCTACAACCGGGAGGAGTTCGCGCGCTTCGACAGCGACGTGGGCGAGTACCGCGCGGTGACCGAGCTGGGGCGGCGCACAGCCAAGTACTGGAACAGCCAGAAGGAGTACCTGGAGCGGAAGCGGGCCCAGGTGGACGCGTAC

>GU332074_Rhabdomys_pumilio_MHC_class_II_antigen_(Rhpu-DRB)_gene_Rhpu-DRB*65_allele_exon_2_and_partial_cds

CAGCACGTGCGGCTTCTGAACAGACACTTCTACAACCGGGAGGAGTTCGTGCTCTTCGACAGCGACGTGGGCGAGTTCCGCGCGGTGACCGAGCTGGGGCGGCCGGACGCTGAGGACTTGAACAGCCAGAAGGAGCTCCTGGAGCAGAGGCGGGCCGCGGTGGACACGTAC

>GU332075_Rhabdomys_pumilio_MHC_class_II_antigen_(Rhpu-DRB)_gene_Rhpu-DRB*66_allele_exon_2_and_partial_cds

CAGCGCGTGCGGTTTCTGGAGAGATACTTCTACAACCGGGAGGAGTACGCGCGCTTCGACAGCGACGTGGGCGAGTACCGCGCGGTGACCGAGCTGGGGCGGCGCACAGCCAAGTACTGGAACAGCCAGAAGGAGTACCTGGAGCGGAAGCGGGCCCAGGTGGACGCGTAC

>GU332076_Rhabdomys_pumilio_MHC_class_II_antigen_(Rhpu-DRB)_gene_Rhpu-DRB*67_allele_exon_2_and_partial_cds

CAGCACGTGTGGTTTCTGGACAGATACTTCTACAACCGGGAGGAGTACGCGCGCTTCGACAGCGACGTGGGCGAGTTCCGCGCGGTGACCGAGCTGGGGCGGCCGGACGCTGAGTACTGGAACAGCCAGAAGGAGTTCCTGGAGCAGAAGCGGGCCGAGCTGGACACAGTG

>GU332077_Rhabdomys_pumilio_MHC_class_II_antigen_(Rhpu-DRB)_gene_Rhpu-DRB*68_allele_exon_2_and_partial_cds

CAGCGCGTGCGTTTTCTGATCAGATACTTCTACAACCGGGAGGAGTACGCGCGCTTCGACAGCGACGTGGGCGAGTACCGCGCGGTGACCGAGCTGGGGCGGCGGGACGCTGAGTATTGGAACAGCCAGAAGGAGCGCCTGGAGCAGAGGCGGGCCGCGGTGGACACGTAC

>GU332078_Rhabdomys_pumilio_MHC_class_II_antigen_(Rhpu-DRB)_gene_Rhpu-DRB*69_allele_exon_2_and_partial_cds

CAGCGCGTGCGGTTTCTGGACAGATACATCCACAACCGGGAGGAGAACGTGCGCTTCGACAGCGACGTGGGCGAGTTCCGCGCGGTGACCGAGCTGGGGCGGCCGGACGCTGATTACTGGAACAGCCAGAAGGAGATCCTGGAGGATGCGCGGGCCCAGGTGGACACGTAC

>GU332079_Rhabdomys_pumilio_MHC_class_II_antigen_(Rhpu-DRB)_gene_Rhpu-DRB*70_allele_exon_2_and_partial_cds

CAGCACGTGCGGCTTCTGATCAGATACTTCTACAACCGGGAGGAGTACGCGCGCTTCGACAGCGACGTGGGCGAGTACCACGCGGTGACGGAGCTGGGGCGGCGGGACGCTGAGTACTGGAACAGCCAGAAGGAGCTCCTGGAGCAGAGGCGGGCCCAGGTGGACACGTAC

>GU332080_Rhabdomys_pumilio_MHC_class_II_antigen_(Rhpu-DRB)_gene_Rhpu-DRB*71_allele_exon_2_and_partial_cds

GAGCGTGTGCGGTATCTGGAGAGACTATTCTACAACCGGGAGGAGTTCGCGCGCTTCGACAGTGACGTGGGGGAGTTCCGCGCGGTGTCCGAGCTGGGGCGGCCGGACGAGGAATACTGGAACAGCCAGAAGGATCTCCTGGAGCGGAAGCGTGCAGAGGTGGACACGTAC

>GU332081_Rhabdomys_pumilio_MHC_class_II_antigen_(Rhpu-DRB)_gene_Rhpu-DRB*72_allele_exon_2_and_partial_cds

CAGCGCGTGCGGTTTCTGGACAGATACTTCTACAACCGGGAGGAGTACGTGCGCTTCGACAGCGACGTGGGCGAGTTCCGCGCGGTGACCGAGCTGGGGCGGCCGGACGCTGAGTACTGGAACAGCCAGAAGGAGTTCCTGGAGCAGAAGCGGGCCGAGCTGGACACAGTG

>GU332082_Rhabdomys_pumilio_MHC_class_II_antigen_(Rhpu-DRB)_gene_Rhpu-DRB*73_allele_exon_2_and_partial_cds

CAGCGCGTGCGGTTTCTGGACAGATACTTCTACAACCGGGAGGAGTACGTGCGCTTCGACAGCGACGTGGGCGAGTTCCGCGCGGTGACCGAGCTGGGGCGGCCGGACGCTGAGTACTGGAACAGCCAGAAGGAGTTCCTGGAGCAGAAGCGGGCCCAGCTGGACACGTAC

>GU332083_Rhabdomys_pumilio_MHC_class_II_antigen_(Rhpu-DRB)_gene_Rhpu-DRB*74_allele_exon_2_and_partial_cds

CAGATCGTGCGGTTTCTGGACAGATACTTCTACAACCGGGAGGAGTACGTGCGCTTCGACAGCGACGTGGGCGAGTTCCGCGCGGTGACCGAGCTGGGGCGGCCGGACGCTGAGTACTGGAACAGCCAGAAGGAGTTCCTGGAGCAGAAGCGGGCCGAGCTGGACACAGTG

>GU332084_Rhabdomys_pumilio_MHC_class_II_antigen_(Rhpu-DRB)_gene_Rhpu-DRB*75_allele_exon_2_and_partial_cds

CAGCGCGTGCGGTTTCTGGACAGATACTTCTACAACCGGGAGGAGTACGTGCGCTTCGACAGCGACGTGGGCGAGTTCCGCGCGGTGACCGAGCTGGGGCAGCCGGACGCTGAGCACTGGAACAGCCAGAAGGAGTTCCTGGAGGATCGGCGGGCCCAGGTGGACACATGC

>GU332085_Rhabdomys_pumilio_MHC_class_II_antigen_(Rhpu-DRB)_gene_Rhpu-DRB*76_allele_exon_2_and_partial_cds

CAGCGCGTGCGGTTTCTGGACAGATACTTCTACAACCGGGAGGAGTACGTGCGCTTCGACAGCGACGTGGGCGAGTTCCGCGCGGTGACCGAGCTGGGGCAGCCGGACGCTGAGTACTGGAACAGCCAGAAGGAGTTCCTGGAGGATCGGCGGGCCGCGGTGGACACGTAC

>GU332086_Rhabdomys_pumilio_MHC_class_II_antigen_(Rhpu-DRB)_gene_Rhpu-DRB*77_allele_exon_2_and_partial_cds

CAGATCGTGCGGTTTCTGGACAGATACTTCTACAACCGGGAGGAGTTCGTGCGCTTCGACAGCGACGTGGGCGAGTACCGCGCGGTGACCGAGCTGGGGCGGCGGGACGCTGAGCACTGGAACAGCCAGAAGGAGCTCCTGGAGCAGAAGCGGGCCCGGGTGGACACGTAC

>GU332087_Rhabdomys_pumilio_MHC_class_II_antigen_(Rhpu-DRB)_gene_Rhpu-DRB*78_allele_exon_2_and_partial_cds

CAGCGCGTGCGGTTTCTGGACAGATACTTCCACAACCGGGAGGAGTACGTGCGCTTCGACAGCGACGTGGGCGAGTACCGCGCGGTGACCGAGCTGGGGCGGCGCACAGCCAAGCACTGGAACAGCCAGAAGGAGATCCTGGAGCATGCGCGGGCCCCGGTGGACACGTAC

>GU332088_Rhabdomys_pumilio_MHC_class_II_antigen_(Rhpu-DRB)_gene_Rhpu-DRB*79_allele_exon_2_and_partial_cds

CAGCGCGTGAGGTTTCTGGAGAGACACATCTACAACCGGGAGGAGTTCATGCGCTTCGACAGCGACGTGGGCGAGTACCGCACTGTGACCGAGCTGGGGCGGCGCATAGCTGAGGACTGGAACAGCCAGAAGGAGCTCCCGGAGCAGAAGCGGGCCCAGGTGGACGCGTAC

>GU332089_Rhabdomys_pumilio_MHC_class_II_antigen_(Rhpu-DRB)_gene_Rhpu-DRB*80_allele_exon_2_and_partial_cds

CAGCACGTGCGGTTTCTGGACAGATACTTCTACAACCGGGAGGAGTACGTGCGCTTCGACAGCGACGTGGGCGAGCACCGCGCGGTGACCGAGCTGGGGCGGCCGGACGCTGAGTACTGGAACAGCCAGAAGGAGCTCCTGGAGGATCGGCGGGCCCAGGTGGACACGTAC

>GU332090_Rhabdomys_pumilio_MHC_class_II_antigen_(Rhpu-DRB)_gene_Rhpu-DRB*81_allele_exon_2_and_partial_cds

CAGATCGTGAGGTTTCTGGAGACACACATCTACAACCGGGAGGAGTTCGTGCGCTTCGACAGCGACGTGGGCGAGTACCGCGCGGTGACCGAGCTGGGGCGGCGCATAGCTGAGGACTTCAACAGCCGGAAGGAGATCCTGGAGGATGCGCGGGCCGCGGTGGACACGTAC

>GU332091_Rhabdomys_pumilio_MHC_class_II_antigen_(Rhpu-DRB)_gene_Rhpu-DRB*82_allele_exon_2_and_partial_cds

CAGCACGTGAGGTTTCTGGAGAGACACATCTACAACCGGGAGGAGTACGCGCGCTTCGACAGCGACGTGGGCGAGTACCGCGCGGTGACCGAGCTGGGGCGGCGCATAGCTGAGGACTTCAACAGCCGGAAGGAGATCCTGGAGGATGCGCGGGCCGCGGTGGACACGTAC

>GU332092_Rhabdomys_pumilio_MHC_class_II_antigen_(Rhpu-DRB)_gene_Rhpu-DRB*83_allele_exon_2_and_partial_cds

CAGCGCGTGCGGTTTCTGATCAGATACTTCTACAACCGGGAGGAGTACGCGCGCTTCGACAGCGACGTGGGCGAGTACCGCGCGGTGACCGAGCTGGGGCGGCGGGACGCTGAGTATTGGAACAGCCAGAAGGAGGAACTGGAGCAGAGGCGGGCCGCGGTGGACACGTAC

>GU332093_Rhabdomys_pumilio_MHC_class_II_antigen_(Rhpu-DRB)_gene_Rhpu-DRB*84_allele_exon_2_and_partial_cds

CAGATCGTGCGGTTTCTGGAGAGACACATCTACAACCGGGAGGAGTTCATGCGCTTCGACAGCGACGTGGGCGAGTACCGCGCGGTGACCGAGCTGGGGCGGCGCATAGCTGAGGACTTCAACAGCCGGAAGGAGCTCCTGGAGCAGAGGCGGGCCGCGGTGGACACGTAC

>GU332094_Rhabdomys_pumilio_MHC_class_II_antigen_(Rhpu-DRB)_gene_Rhpu-DRB*85_allele_exon_2_and_partial_cds

CAGCGCGTGCGGTTTCTGGAGAGACACATCTACAACCGGGAGGAGTTCATGCGCTTCGACAGCGACGTGGGCGAGTACCGCGCGGTGACCGAGCTGGGGCGGCGCATAGCTGAGGACTTCAACAGCCGGAAGGAGCTTCTGGAGCGGAAGCGGGCCCAGCTGGACACGTAC

>GU332095_Rhabdomys_pumilio_MHC_class_II_antigen_(Rhpu-DRB)_gene_Rhpu-DRB*86_allele_exon_2_and_partial_cds

CAGATCGTGCGGTTTCTGGACAGATACTTCTACAACCGGGAGGAGTACGCGCGCTTCGACAGCGACGTGGGCGAGTACCGCGCGGTGACCGAGCTGGGGCGGCGGGACGCCGAGTACTGGAACAGCCAGAAGGAGCTCCTGGAGGATCGGCGGGCCCAGGTGGACACAGTG

>GU332096_Rhabdomys_pumilio_MHC_class_II_antigen_(Rhpu-DRB)_gene_Rhpu-DRB*87_allele_exon_2_and_partial_cds

CAGCTCGTGCGGTTTCTGGACAGATACTTCTACAACCGGGAGGAGTACGTGCGCTTCGACAGCGACGTGGGCGAGCACCGCGCGGTGACCGAGCTGGGGCGGCCGGACGCTGAGTACTGGAACAGCCAGAAGGAGCTCCTGGAGGATCGGCGGGCCCAGGTGGACACGTAC

>GU332097_Rhabdomys_pumilio_MHC_class_II_antigen_(Rhpu-DRB)_gene_Rhpu-DRB*88_allele_exon_2_and_partial_cds

CAGCACGTGCGGTTTCTGGACAGATACTTCTACAACCGGGAGGAGTACGCGCGCTTCGACAGCGACGTGGGCGAGTACCGCGCGGTGACCGAGCTGGGGCGGCCGGACGCTGAGTACTGGAACAGCCAGAAGGAGCTCCTGGAGCAGAAGCGGGCCCGGGTGGACACGTAC

>GU332098_Rhabdomys_pumilio_MHC_class_II_antigen_(Rhpu-DRB)_gene_Rhpu-DRB*89_allele_exon_2_and_partial_cds

CAGCACGTGAGGTTTCTGGAGAGACACATCTACAACCGGGAGGAGTTCATGCGCTTCGACAGCGACGTGGGCGAGTTCCGCACTGTGACCGAGCTGGGGCGGCGCATAGCTGAGGACTGGAACAGCCAGAAGGAGTACCTGGAGGATCGGCGGGCCCAGGTGGACGCGTAC

>GU332099_Rhabdomys_pumilio_MHC_class_II_antigen_(Rhpu-DRB)_gene_Rhpu-DRB*90_allele_exon_2_and_partial_cds

CAGCGCGTGCGGTTTCTGGAGAGACTCTTCTACAACCGGGAGGAGTACGCGCGCTTCGACAGCGACGTGGGCGAGTTCCGCGCGGTGTCCGAGCTGGGGCGGCCGGACGAGGAATACTGGAACAGCCAGAAGGATCTCCTGGAGCGGAAGCGTGCAGAGGTGGACACGTAC

>GU332100_Rhabdomys_pumilio_MHC_class_II_antigen_(Rhpu-DRB)_gene_Rhpu-DRB*91_allele_exon_2_and_partial_cds

CAGCGCGTGCGGTTTCTGGAGAGATACTTCTACAACCGGGAGGAGTACGCGCGCTTCGACAGCGACGTGGGCGAGTACCGCGCGGTGACCGAGCTGGGGCGGCGCACAGCCAAGTATTGGAACAGCCAGAAGGAGGTCCTGGAGGATGCGCGGGCCCAGGTGGACACGTAC

>GU332101_Rhabdomys_pumilio_MHC_class_II_antigen_(Rhpu-DRB)_gene_Rhpu-DRB*92_allele_exon_2_and_partial_cds

CAGCGCGTGCGGTTTCTGGAGAGATACTTCTACAACCGGGAGGAGTACGCGCGCTTCGACAGCGACGTGGGCGAGTACCGCGCAGTGACCGAGCTGGGGCGGCGCACAGCCAAGTACTGGAACAGCCAGAAGGAGATCCTGGAGCGGAAGCGTGCCCAGGTGGACGCGTAC

>GU332102_Rhabdomys_pumilio_MHC_class_II_antigen_(Rhpu-DRB)_gene_Rhpu-DRB*93_allele_exon_2_and_partial_cds

CAGCACGTGCGGTTTCTGGACAGATACTTCTACAACCGGGAGGAGTACGCGCGCTTCGACAGCGACGTGGGCGAGTACCGCGCGGTGACCGAGCTGGGGCGGCGGGACGCTGAGCACTGGAACAGCCAGAAGGAGTACCTGGAGCGGAAGCGGGCCCAGGTGGACGCGTAC

>GU332103_Rhabdomys_pumilio_MHC_class_II_antigen_(Rhpu-DRB)_gene_Rhpu-DRB*94_allele_exon_2_and_partial_cds

CAGCGCGTGCGGTTTCTGGATAGACACTTCTACAACCGGGAGGAGTACGCGCGCTTCGACAGCGACGTGGGCGAGTACCGCGCGGTGACCGAGCTGGGGCGGCGGGACGCTGAATACTGGAACAGCCAGAAGGATCTCCTGGAGCAGAGGCGGGCCCAGGTGGACACGTAC

>GU332104_Rhabdomys_pumilio_MHC_class_II_antigen_(Rhpu-DRB)_gene_Rhpu-DRB*95_allele_exon_2_and_partial_cds

CAGCGCGTGCGGTTTCTGGACAGATACATCCACAACCGGGAGGAGTTCGTGCGCTTCGACAGCGACGTGGGCGAGTTCCGCGCGGTGACCGAGCTGGGGCGGCGCATAGCTGAGGACTTGAACAGCCAGAAGGAGCTCCTGGAGCAGAGGCGGGCCGCGGTGGACACGTAC

>GU332105_Rhabdomys_pumilio_MHC_class_II_antigen_(Rhpu-DRB)_gene_Rhpu-DRB*96_allele_exon_2_and_partial_cds

CAGCGCGTGCGGTTTCTGGACAGATACTTCTACAACCGGGAGGAGTACGTGCGCTTCGACAGCGACGTGGGCGAGCACCGCGCGGTGACCGAGCTGGGGCGGCCGGACGCTGAGTACTGGAACAGCCAGAAGGAGCTCCTGGAGGATCGGCGGGCCGAGGTGGACGCGTAC

>GU332106_Rhabdomys_pumilio_MHC_class_II_antigen_(Rhpu-DRB)_gene_Rhpu-DRB*97_allele_exon_2_and_partial_cds

CAGCGCGTGCGGTTTCTGGAGAGATACTTCTACAACCGGGAGGAGTTCGTGCGCTTCGACAGCGACGTGGGCGAGTACCGCGCGGTGACCGAGCTGGGGCGGCGCACAGCCGAGTACTGGAACAGCCAGAAGGAGATCCTGGAGCGGAAGCGGGCCCAGGTGGACGCGTAC

>GU332107_Rhabdomys_pumilio_MHC_class_II_antigen_(Rhpu-DRB)_gene_Rhpu-DRB*98_allele_exon_2_and_partial_cds

CAGCGCGTGCGGTTTCTGGACAGATACATCCACAACCGGGAGGAGTTCGTGCGCTTCGACAGCGACGTGGGCGAGCACCGCGCGGTGACCGAGCTGGGGCGGCGCATAGCTGAGGACTGGAACAGCCAGAAGGAGATCCTGGAGCGGAAGCGGGCCGAGGTGGACGCGTAC

>GU332108_Rhabdomys_pumilio_MHC_class_II_antigen_(Rhpu-DRB)_gene_Rhpu-DRB*99_allele_exon_2_and_partial_cds

CAGCGCGTGCGGTTTCTGGACAGATACTTCTACAACCGGGAGGAGTACGCGCGCTTCGACAGCGACGTGGGCGAGTACCGCGCGGTGACCGAGCTGGGGCGGCGCACAGCCAAGTACTGGAACAGCCAGAAGGAGCTCCTGGAGGATGCGCGGGCCGCGGTGGACACGTAC

>GU332109_Rhabdomys_pumilio_MHC_class_II_antigen_(Rhpu-DRB)_gene_Rhpu-DRB*100_allele_exon_2_and_partial_cds

CAGCACGTGCGGTTTCTGGACAGATACTTCTACAACCGGGAGGAGTACGTGCGCTTCGACAGCGACGTGGGCGAGTACCGCGCGGTGACCGAGCTGGGGCGGCCGGACGCCGAGTATCGGAACAGCCAGAAGGAAGTCCTGGAGCGGAAGCGGGCCGAGGTGGACACGTAC

>GU332110_Rhabdomys_pumilio_MHC_class_II_antigen_(Rhpu-DRB)_gene_Rhpu-DRB*101_allele_exon_2_and_partial_cds

CAGCGCGTGCGGTTTCTGGACAGATACTTCTACAACCGGGAGGAGTACGCGCGCTTCGACAGCGACGTGGGCGAGTACCGCGCGGTGACCGAGCTGGGGCGGCGGGACGCCGAGTACTGGAACAGCCAGAAGGAGCTCCTGGAGGATCGGCGGGCCCAGGTGGACACGTAC

>GU332111_Rhabdomys_pumilio_MHC_class_II_antigen_(Rhpu-DRB)_gene_Rhpu-DRB*102_allele_exon_2_and_partial_cds

CAGCACGTGTGGTTTCTGGACAGATACTTCTACAACCGGGAGGAGTACGCGCGCTTCGACAGCGACGTGGGCGAGTTCCGCGCGGTGACCGAGCTGGGGCGGCCGGACGCTGAGTACTGGAACAGCCAGAAGGAGTTCCTGGAGCAGAAGCGGGCCGAGCTGGACACGTAC

>GU332112_Rhabdomys_pumilio_MHC_class_II_antigen_(Rhpu-DRB)_gene_Rhpu-DRB*103_allele_exon_2_and_partial_cds

CAGCGCGTGCGGTTTCTGGACAGATACTTCCACAACCGGGAGGAGTTCGTGCGCTTCGACAGCGACGTGGGCGAGCACCGCGCGGTGACCGAGCTGGGGCGGCGCACAGCTAAGGACTGGAACAGCCAGAAGGAGCTCCTGGAGCGGAAGCGGGCCCAGGTGGACACGTAC

>GU332113_Rhabdomys_pumilio_MHC_class_II_antigen_(Rhpu-DRB)_gene_Rhpu-DRB*104_allele_exon_2_and_partial_cds

CAGCGCGTGCGGTTTCTGGACAGATACTTCTACAACCGGGAGGAGTACGTGCGCTTCGACAGCGACGTGGGCGAGTTCCGCGCGGTGACCGAGCTGGGGCGGCCGGACGCTGAGTACTGGAACAGCCAGAAGGAGTTCCTGGAGGATCGGCGGGCCGCGGTGGACACGTAC

>GU332114_Rhabdomys_pumilio_MHC_class_II_antigen_(Rhpu-DRB)_gene_Rhpu-DRB*105_allele_exon_2_and_partial_cds

CAGCGCGTGCGGTTTCTGGACAGCTACTTCTACAACCGGGAGGAGTACGTGCGCTTCGACAGCGACGTGGGCGAGCACCGCGCGGTGACCGAGCTGGGGCGGCCGGCCGCTGAGTACTGGAACAGCCAGAAGGAGCTCCTGGAGGATCGGCGGGCCCAGGTGGACACGTAC

>GU332115_Rhabdomys_pumilio_MHC_class_II_antigen_(Rhpu-DRB)_gene_Rhpu-DRB*106_allele_exon_2_and_partial_cds

CAGCACGTGAGGTTTCTGGACAGATACTTCTACAACCGGGAGGAGTACGCGCGCTTCGACAGCGACGTGGGCGAGTTCCGCGCGGTGACCGAGCTGGGGCGGCGCACAGCCAAGTACTGGAACAGCCAGAAGGAGCTCCTGGAGCAGAAGCGGGCCGCGGTGGACACGTAC

>GU332116_Rhabdomys_pumilio_MHC_class_II_antigen_(Rhpu-DRB)_gene_Rhpu-DRB*107_allele_exon_2_and_partial_cds

CAGCGCGTGCGGTTTCTGGAGAGACACATCTACAACCGGGAGGAGTTCATGCGCTTCGACAGCGACGTGGGCGAGTACCGCGCGGTGACCGAGCTGGGGCGGCGCATAGCTGAGGACTTCAACAGCCGGAAGGAGATCCTGGAGGATGCACGGGCCCAGGTGGACACGTAC

>GU332117_Rhabdomys_pumilio_MHC_class_II_antigen_(Rhpu-DRB)_gene_Rhpu-DRB*108_allele_exon_2_and_partial_cds

CAGCGCGTGCGGTTTCTGGACAGATACTTCTACAACCGGGAGGAGTACGCGCGCTTCGACAGCGACGTGGGCGAGTACCGCGCGGTGACCGAGCTGGGGCGGCGGGACGCTGAGTATTGGAACAGCCAGAAGGAGGAACTGGAGCAGAGGCGGGCCGCGGTGGACACGTAC

>GU332118_Rhabdomys_pumilio_MHC_class_II_antigen_(Rhpu-DRB)_gene_Rhpu-DRB*109_allele_exon_2_and_partial_cds

CAGCGCGTGCGGTTTCTGGACAGATACTTCTACAACCGGGAGGAGTACGTGCGCTTCGACAGCGACGTGGGCGAGTTCCGCGCGGTGACCGAGCTGGGGCGGCCGGACGCTGAGTACTGGAACAGCCAGAAGGAGTTCCTGGAGGATCGGCGGGCCCAGGTGGACACGTAC

>GU332119_Rhabdomys_pumilio_MHC_class_II_antigen_(Rhpu-DRB)_gene_Rhpu-DRB*110_allele_exon_2_and_partial_cds

CAGCGCGTGCGGTTTCTGGACAGATACTTCTACAACCGGGAGGAGTACGTGCGCTTCGACAGCGACATGGGCGAGTACCGCGCGGTGACCGAGCTGGGGCGGGGCATAGCTGAGGACTGGAACAGCCAGAAGGAGTACCTGGAGGATGCGCGGGCCGCGGTGGACACGTAC

>GU332120_Rhabdomys_pumilio_MHC_class_II_antigen_(Rhpu-DRB)_gene_Rhpu-DRB*111_allele_exon_2_and_partial_cds

CAGCGCGTGCGGTTTCTGGACAGATACTTCTACAACCGGGAGGAGTACGCGCGCTTCGACAGCGACGTGGGCGAGTACCGCGCGGTGACCGAGCTGGGGCGGCGGGACGCCGAGTACTACAACAGCCAGAAGGAGATCCTGGAGGATGCGCGGGCCGCGGTGGACACGTAC

>GU332121_Rhabdomys_pumilio_MHC_class_II_antigen_(Rhpu-DRB)_gene_Rhpu-DRB*112_allele_exon_2_and_partial_cds

CAGCGCGTGCGGTTTCTGGACAGATACTTCTACAACCGGGAGGAGTACGCGCGCTTCGACAGCGACGTGGGCGAGTACCGCGCGGTGACCGAGCTGGGGCGGCGGGACGCCGAGTACTGGAACAGCCAGAAGGAGCTCCTGGAGCAGAGGCGGGCCCAGGTGGACACATAC

>GU332122_Rhabdomys_pumilio_MHC_class_II_antigen_(Rhpu-DRB)_gene_Rhpu-DRB*113_allele_exon_2_and_partial_cds

CAGCGCGTGAGGTTTCTGGACAGATACTTCTACAACCGGGAGGAGTACGCGCGCTTCGACAGCGACGTGGGCGAGTTCCGCGCGGTGACCGAGCTGGGGCGGCCGGACGCTGAGTACTGGAACAGCCAGAAGGAGATCCTGGAGGATGCGCGGGCCGCGGTGGACACGTAC

>GU332123_Rhabdomys_pumilio_MHC_class_II_antigen_(Rhpu-DRB)_gene_Rhpu-DRB*114_allele_exon_2_and_partial_cds

CAGCGCGTGCGGTTTCTGGACAGATACTTCTACAACCGGGAGGAGTACGCGCGCTTCGACAGCGACGTGGGCGAGTACCGCGCGGTGACGGAGTTGGGGCGGCCGGACGCTGAGTATTGGAACAGCCAGAAGGAGATCCTGGAGGATGGGCGGGCCCAGGTGGACACGTAC

>GU332124_Rhabdomys_pumilio_MHC_class_II_antigen_(Rhpu-DRB)_gene_Rhpu-DRB*115_allele_exon_2_and_partial_cds

CAGCACGTGCGGTTTCTGGACAGATACATCCACAACCGGGAGGAGTTCGTGCGCTTCGACAGCGACGTGGGCGAGTACCGCGCGGTGACCGAGCTGGGGCGGGGCATAGCTGAGGACTTGAACAGCCAGAAGGAGTTCCTGGAGCGGAAGCGGGCCGAGCTGGCACAGTAC

>GU332125_Rhabdomys_pumilio_MHC_class_II_antigen_(Rhpu-DRB)_gene_Rhpu-DRB*116_allele_exon_2_and_partial_cds

GAGCGTGTGCGGTATCTGGAGAGACTATTCTACAACCGGGAGGAGTTTGCGCGCTTCGACAGTGACGTGGGCGAGTTCCGCGCGGTGTCCGAGCTGGGGCGGCCGGACGAGGAATACTGGAACAGCCAGAAGGATCTCCTGGAGCGGAAGCGTGCAGAGGTGGACACGTAC

>GU332126_Rhabdomys_pumilio_MHC_class_II_antigen_(Rhpu-DRB)_gene_Rhpu-DRB*117_allele_exon_2_and_partial_cds

CAGCGCGTGCGGTTTCTGGAGAGATACTTCTACAACCGGGAGGAGTACGCGCGCTTCGACAGCGACGTGGGCGAGTACCGCGCGGTGACCGAGCTGGGGCGGCGGACAGCCGAGTACTGGAACAGCCAGAAGGAGGTCCTGGAGGATGGGCGGGCCGAGGTGGACACGTAC

>GU332127_Rhabdomys_pumilio_MHC_class_II_antigen_(Rhpu-DRB)_gene_Rhpu-DRB*118_allele_exon_2_and_partial_cds

CAGCGCGTGCGGTTTCTGGAGAGACACATCTACAACCGGGAGGAGTTCGTGCGCTTCGACAGCGACGTGGGCGAGTACCGCGCGGTGACCGAGCTGGGGCGGGGCATAGCTGAGTACTGGAACAGCCAGAAGGAGTACCTGGAGGATGCGCGGGCCGCGGTGGACACGTAC

>GU332128_Rhabdomys_pumilio_MHC_class_II_antigen_(Rhpu-DRB)_gene_Rhpu-DRB*119_allele_exon_2_and_partial_cds

CAGCGCGTGCGGTTTCTGGAGAGACACATCCACAACCGGGAGGAGTTCGTGCGCTTCGACAGCGACGTGGGCGAGTTCCGCGCGGTGACCGAGCTGGGGCGGCGCATAGCTGAGGACTTGAACAGCCAGAAGGAGCTCCTGGAGCAGAAGCGGGCCGCGGTGGACACGTAC

>GU332129_Rhabdomys_pumilio_MHC_class_II_antigen_(Rhpu-DRB)_gene_Rhpu-DRB*120_allele_exon_2_and_partial_cds

CAGCGCGTGCGGTTTCTGGACAGATACTTCTACAACCGGGAGGAGTACGCGCGCTTCGACAGCGACGTGGGCGAGTACCGCGCGGTGACCGAGCTGGGGCGGCCGGACGCTGAGTACTGGAACAGCCAGAAGGAGCTCCTGGAGCAGAAGCGGGCCCAGGTGGACACGTAC

>GU332130_Rhabdomys_pumilio_MHC_class_II_antigen_(Rhpu-DRB)_gene_Rhpu-DRB*121_allele_exon_2_and_partial_cds

CAGCGCGTGCGGTTTCTGGACAGATACTTCTACAACCGGGAGGAGTACGTGCGCTTCGACAGCGACGTGGGCGAGCACCGCGCGGTGACCGAGCTGGGGCGGCCGGACGCTGAGTACTGGAACAGCCAGAAGGAGCTCCTGGAGGAGCGGCGGGCCCAGGTGGACACGTAC

>GU332131_Rhabdomys_pumilio_MHC_class_II_antigen_(Rhpu-DRB)_gene_Rhpu-DRB*122_allele_exon_2_and_partial_cds

CAGCGCGTGCGGTTTCTGGAGAGATACTTCTACAACCGGGAGGAGTACGCGCGCTTCGACAGCGACGTGGGCGAGTACCGCGCGGTGACCGAGCTGGGGCGGCGGGACGCTGAGCACTGGAACAGCCAGAAGGAGCTCCTGGAGGATGCGCGGGCCGAGNTGGACACGTAC

>GU332132_Rhabdomys_pumilio_MHC_class_II_antigen_(Rhpu-DRB)_gene_Rhpu-DRB*123_allele_exon_2_and_partial_cds

CAGCGCGTGCGGTTTCTGGACAGATACATCCACAACCGGGAGGAGTTCGTGCGCTTCGACAGCGACGTGGGCGAGTTCCGCGCGGTGACCGAGCTGGGGCGGCGCATAGCTGAGGACTTGAACAGCCAGAAGGAGTACCTGGAGCAGAAGCGGGCCGCGGTGGACACGTAC

>GU332133_Rhabdomys_pumilio_MHC_class_II_antigen_(Rhpu-DRB)_gene_Rhpu-DRB*124_allele_exon_2_and_partial_cds

CAGCGCGTGCGGTTTCTGGACAGATATTTCTACAACCGGGAGGAGTACGTGCGCTTCGACAGCGACGTGGGCGAGTTCCGCGCGGTGACCGAGCTGGGGCGGCGGGMTGAGGAATACTGGAACAGCCAGAAGGAGATTCTGGAGCGGGCTCGGGCCGCGGTGGACACGTTC

>GU332134_Rhabdomys_pumilio_MHC_class_II_antigen_(Rhpu-DRB)_gene_Rhpu-DRB*125_allele_exon_2_and_partial_cds

CAGCGCGTGCGGTTTCTGGACAGATATTTCTACAACCGGGAGGAGTACGTGCGCTTCGACAGCGACGTGGGCGAGTACCGCGCGGTGACCGAGCTGGGGCGGCGGGGCGAGGAATACTGGAACAGCCAGAAGGAGATTCTGGAGGAGGCGCGGGCCGCGGTGGACACGTTC

>GU332135_Rhabdomys_pumilio_MHC_class_II_antigen_(Rhpu-DRB)_gene_Rhpu-DRB*126_allele_exon_2_and_partial_cds

CAGCACGTGCGGTTTCTGGACAGATACATCCACAACCGGGAGGAGTTCGTGCGCTTCGACAGCGACGTGGGCGAGTACCGCGCGGTGACCGAGCTGGGGCGGGGCATAGCTGAGGACTGGAACAGCCAGAAGGAGTTCCTGGAGCGGAAGCGGGCCGTAGTGGACACGTAC

>GU332136_Rhabdomys_pumilio_MHC_class_II_antigen_(Rhpu-DRB)_gene_Rhpu-DRB*127_allele_exon_2_and_partial_cds

CAGCGCGTGCGGTTTCTGGACAGATACTTCTACAACCGGGAGGAGTACGTGCGCTTCGACAGCGACGTGGGCGAGCACCGCGCGGTGACCGAGCTGGGGCGGCCGGACGCTGAGTACTGGAACAGCCAGAAGGAGCTCCTGGAGGAGAAGCGGGCCCAGGTGGACACGTAC

>GU332137_Rhabdomys_pumilio_MHC_class_II_antigen_(Rhpu-DRB)_gene_Rhpu-DRB*128_allele_exon_2_and_partial_cds

CAGCGCGTGCGGTTTCTGGACAGATACTTCTACAACCGGGAGGAGTTCGTGCGCTTCGACAGCGACGTGGGCGAGTACCGCACTGTGACCGAGCTGGGGCGGGGCATAGCTGAGGACTGGAACAGCCAGAAGGAGTTCCTGGAGCGGAAGCGGGCCCGGGTGGACACGTAC

>GU332138_Rhabdomys_pumilio_MHC_class_II_antigen_(Rhpu-DRB)_gene_Rhpu-DRB*129_allele_exon_2_and_partial_cds

CAGCGCGTGCGGTTTCTGGAGAGATACTTCTACAACCGGGAGGAGTACGCGCGCTTCGACAGCGACGTGGGCGAGTACCGCGCGGTGACCGAGCTGGGGCGGCGCACAGCCAAGTACTGGAACAGCCAGAAGGAGTACCTGGAGCGGAAGCGGGCCCAGGTGGACACGTAC

>GU332139_Rhabdomys_pumilio_MHC_class_II_antigen_(Rhpu-DRB)_gene_Rhpu-DRB*130_allele_exon_2_and_partial_cds

CAGCGCGTGCGGTTTCTGGACAGATACTTCTACAACCGGGAGGAGTACGCGCGCTTCGACAGCGACGTGGGCGAGTTCCGCGCGGTGACCGAGCTGGGGCGGCGCACAGCCAAGTACTGGAACAGCCAGAAGGAGCTCCTGGAGCAGAAGCGGGCCGCGGTGGACACGTAC

>GU332140_Rhabdomys_pumilio_MHC_class_II_antigen_(Rhpu-DRB)_gene_Rhpu-DRB*131_allele_exon_2_and_partial_cds

CAGCGCGTGCGGTTTCTGGAGAGCTACTTCTACAACCGGGAGGAGTACGCGCGCTTCGACAGCGACGTGGGCGAGTACCGCGCGGTGACCGAGCTGGGGCGGCGCACAGCCAAGTACTGGAACAGCCAGAAGGAGATCCTGGAGCGGAAGCGGGCCCAGGTGGACGCGTAC

>GU332141_Rhabdomys_pumilio_MHC_class_II_antigen_(Rhpu-DRB)_gene_Rhpu-DRB*132_allele_exon_2_and_partial_cds

GAGCGTGTGCGGTATCTGGAGAGACTATTCTACAACCGGGAGGAGTTTGCGCGCTTCGACAGTGACGTGGGCGAGTTCCGCGCGGTGTCCGAGCTGGGGCGGCCGGACGAGGAATACTGGAACAGCCAGAAGGAGATCCTGGAGCGGAGGCGGGCCCAGGTGGACACGTAC

>GU332142_Rhabdomys_pumilio_MHC_class_II_antigen_(Rhpu-DRB)_gene_Rhpu-DRB*133_allele_exon_2_and_partial_cds

CAGCGCGTGCGGTTTCTGGAGAGATACTTCTACAACCGGGAGGAGTACGCGCGCTTCGACAGCGACGTGGGCGAGTACCGCGCGGTGACCGAGCTGGGGCGGCCGACAGCCAAGTACTGGAACAGCCAGAAGGAGATCCTGGAGGATGCGCGGGCCCGGGTGGACACGTAC

>GU332143_Rhabdomys_pumilio_MHC_class_II_antigen_(Rhpu-DRB)_gene_Rhpu-DRB*134_allele_exon_2_and_partial_cds

CAGCGCGTGCGGTTTCTGGACAGATACTTCTACAACCGGGAGGAGTACGTGCGCTTCGACAGCGACGTGGGCGAGTACCGCGCGGTGACCGAGCTGGGGCGCGGGGACGCTGAGTATTGGAACAGCCAGAAGGAGCTCCTGGAGCGGAAGCGGGCCCAGGTGGACACGTAC

>GU332144_Rhabdomys_pumilio_MHC_class_II_antigen_(Rhpu-DRB)_gene_Rhpu-DRB*135_allele_exon_2_and_partial_cds

CAGCGCGTGCGGTTTCTGGACAGATACATCCACAACCGGGAGGAGTTCGTGCGCTTCGACAGCGACGTGGGCGAGCACCGCGCGGTGACCGAGCTGGGGCGGCCGATAGCTGAGGACTGGAACAGCCAGAAGGAGCTCCTGGAGCGGAAGCGGGCCCAGCTGGACACGTAC

>GU332145_Rhabdomys_pumilio_MHC_class_II_antigen_(Rhpu-DRB)_gene_Rhpu-DRB*136_allele_exon_2_and_partial_cds

CAGCGCGTGCGGTTTCTGGACAGATACATCCACAACCGGGAGGAGTTCGTGCGCTTCGACAGCGACGTGGGCGAGCACCGCGCGGTGACCGAGCTGGGGCGGCCGATAGCTGAGGACTGGAACAGCCAGAAGGAGCTCCTGGAGCGGAAGCGGGCCGAGCTGGACACGTAC

>GU332146_Rhabdomys_pumilio_MHC_class_II_antigen_(Rhpu-DRB)_gene_Rhpu-DRB*137_allele_exon_2_and_partial_cds

CAGCTCGTGCGGTTTCTGGAGAGATACTTCTACAACCGGGAGGAGTACGCGCGCTTCGACAGCGACGTGGGCGAGTACCGCGCGGTGACCGAGCTGGGGCGCGGGCATGCTGATCACTGGAACAGCCAGAAGGAGGAACTGGAGCAGAAGCGGGCCCAGGTGGACGCGTAC

>GU332147_Rhabdomys_pumilio_MHC_class_II_antigen_(Rhpu-DRB)_gene_Rhpu-DRB*138_allele_exon_2_and_partial_cds

CAGCGCGTGCGGTTTCTGATCAGATACTTCTACAACCGGGAGGAGTACGCGCGCTTCGACAGCGACGTGGGCGAGTACCGCGCGGTGACCGAGCTGGGGCGGCGGGACGCTGAGTATTGGAACAGCCAGAAGGAGCTCCTGGAGCAGAGGCGGGCCCAGGTGGACACGTAC

>GU332148_Rhabdomys_pumilio_MHC_class_II_antigen_(Rhpu-DRB)_gene_Rhpu-DRB*139_allele_exon_2_and_partial_cds

CAGCGCGTGCGGTTTCTGGAGAGATACTTCTACAACCGGGAGGAGTACGTGCGCTTCGACAGCGACGTGGGCGAGTACCGCGCGGTGACCGAGCTGGGGCGGCCCTCAGCCGAGAACTGGAACAGCCAGAAGGAGAGCCTGGAGCGGAAGCGGGCCCGGGTGGACACGTAC

>GU332150_Rhabdomys_pumilio_MHC_class_II_antigen_(Rhpu-DRB)_gene_Rhpu-DRB*141_allele_exon_2_and_partial_cds

CAGCGCGTGCGGTTTCTGGACAGATACTTCTACAACCGGGAGGAGTACGTGCGCTTCGACAGCGACGTGGGCGAGTACCGCGCGGTGACCGAGCTGGGGCGGCGGGACGCTGAGTATTGGAACAGCCAGAAGGAGCTCCTGGAGCGGAAGCGGGCCCAGGTGGACGCGTAC

>GU332151_Rhabdomys_pumilio_MHC_class_II_antigen_(Rhpu-DRB)_gene_Rhpu-DRB*142_allele_exon_2_and_partial_cds

CAGCGCGTGCGGTTTCTGGACAGATACTTCTACAACCGGGAGGAGTTCATGCGCTTCGACAGCGACGTGGGCGAGTACCGCGCGGTGACCGAGCTGGGGCGGCGGGACGCTGAGCACTGGAACAGCCAGAAGGAGCTCCTGGAGCAGAAGCGGGCCCGGGTGGACACGTAC

>GU332152_Rhabdomys_pumilio_MHC_class_II_antigen_(Rhpu-DRB)_gene_Rhpu-DRB*143_allele_exon_2_and_partial_cds

CAGCGCGTGCGGTTTCTGGAGAGATATTTCTACAACCGGGAGGAGTACGTGCGCTTCGACAGCGACGTGGGCGAGTACCGCGCGGTGACCGAGCTGGGGCGGCCCTCAGCCGAGAACTGGAACAGCCAGAAGGAGATCCTGGAGCGGAAGCGGGCCGCGGTGGACGCGTAC

>GU332153_Rhabdomys_pumilio_MHC_class_II_antigen_(Rhpu-DRB)_gene_Rhpu-DRB*144_allele_exon_2_and_partial_cds

CAGCGCGTGCGGTTTCTGGAGAGATACTTCTACAACCGGGAGGAGTACGCGCGCTTCGACAGCGACGTGGGCGAGTACCGCGCGGTGACCGAGCTGGGGCGGCGGGACGCTGAGCACTGGAACAGCCAGAAGGAGATCCTGGAGCAGAAGCGGGCCGCGGTGGACACGTAC

>GU332154_Rhabdomys_pumilio_MHC_class_II_antigen_(Rhpu-DRB)_gene_Rhpu-DRB*145_allele_exon_2_and_partial_cds

CAGCGCGTGCGGTTTCTGGACAGATACTTCTACAACCGGGAGGAGTACGCGCGCTTCGACAGCGACGTGGGCGAGTACCGCGCGGTGACCGAGCTGGGGCGGCCGGACGCTGAGTACTGGAACAGCCAGAAGGAGCTCCTGGAGGATCGGCGGGCCCAGGTGGACGCGTAC

>GU332155_Rhabdomys_pumilio_MHC_class_II_antigen_(Rhpu-DRB)_gene_Rhpu-DRB*146_allele_exon_2_and_partial_cds

CAGCGCGTGCGGTTTCTGGACAGATACTTCTACAACCGGGAGGAGTACGTGCGCTTCGACAGCGACGTGGGCGAGCACCGCGCGGTGACCGAGCTGGGGCGGCCGGACGCTGAGTACTGGAACAGCCAGAAGGAGCTCCTGGAGGATGCGCGGGCCGAGGTGGACGCGTAC

>GU332156_Rhabdomys_pumilio_MHC_class_II_antigen_(Rhpu-DRB)_gene_Rhpu-DRB*147_allele_exon_2_and_partial_cds

CAGCACGTGCGGTTTCTGGACAGATACTTCTACAACCGGGAGGAGTACGCGCGCTTCGACAGCGACGTGGGCGAGTACCGCGCGGTGACCGAGCTGGGGCGGCGGGACGCCGAGTACTGGAACAGCCAGAAGGAGCTCCTGGAGGATCGGCGGGCCCAGGTGGACACGTAC

>GU332157_Rhabdomys_pumilio_MHC_class_II_antigen_(Rhpu-DRB)_gene_Rhpu-DRB*148_allele_exon_2_and_partial_cds

CAGCGCGTGCGGTTTCTGGACAGATACTTCTACAACCGGGAGGAGTACGCGCGCTTCGACAGCGACGTGGGCGAGTACCGCGCGGTGACCGAGCTGGGGCGGCGCACAGCCAAGTACTGGAACAGCCAGAAGGAGTACCTGGAGGATGCGCGGGCCGCGGTGGACACGTAC

>GU332158_Rhabdomys_pumilio_MHC_class_II_antigen_(Rhpu-DRB)_gene_Rhpu-DRB*149_allele_exon_2_and_partial_cds

CAGCGCGTGCGGTTTCTGGACAGATACTTCTACAACCGGGAGGAGTACGCGCGCTTCGACAGCGACGTGGGCGAGTTCCGCGCGGTGACCGAGCTGGGGCGGCGCACAGCCAAGTACTGGAACAGCCAGAAGGAGCTCCTGGAGGATGCGCGGGCCGCGGTGGACACGTAC

>GU332159_Rhabdomys_pumilio_MHC_class_II_antigen_(Rhpu-DRB)_gene_Rhpu-DRB*150_allele_exon_2_and_partial_cds

CAGCGCGTGCGGTTTCTGGACAGATACTTCTACAACCGGGAGGAGTTCATGCGCTTCGACAGCGACGTGGGCGAGTACCGCGCGGTGACCGAGCTGGGGCGGCGCATAGCTGAGGACTTCAACAGCCGGAAGGAGATCCTGGAGGATGCACGGGCCCAGGTGGACACGTAC

>GU332160_Rhabdomys_pumilio_MHC_class_II_antigen_(Rhpu-DRB)_gene_Rhpu-DRB*151_allele_exon_2_and_partial_cds

CAGCACGTGAGGTTTCTGGAGAGACACATCTACAACCGGGAGGAGTTCATGCGCTTCGACAGCGACGTGGGCGAGTTCCGCACTGTGACCGAGCTGGGGCGGCGCATAGCTGAGGACTGGAACAGCCAGAAGGAGTACCTGGAGGATCGGCGGGCCCAGGTGGACACGTAC

>GU332161_Rhabdomys_pumilio_MHC_class_II_antigen_(Rhpu-DRB)_gene_Rhpu-DRB*152_allele_exon_2_and_partial_cds

CAGCGCGTGCGGTTTCTGGACAGATACTTCTACAACCGGGAGGAGTACGCGCGCTTCGACAGCGACGTGGGCGAGTACCGCGCGGTGACCGAGCTGGGGCGGCCGGACGCTGAGTATTGGAACAGCCAGAAGGAGATCCTGGAGGATCGGCGGGCCCAGGTGGACACGTAC

>GU332162_Rhabdomys_pumilio_MHC_class_II_antigen_(Rhpu-DRB)_gene_Rhpu-DRB*153_allele_exon_2_and_partial_cds

CAGCGCGTGCGGTTTCTGGACAGATACTTCTACAACCGGGAGGAGTTCGTGCGCTTCGACAGCGACGTGGGCGAGCACCGCGTGGTGACCGAGCTGGGGCGGCCGGTCGCTGAGTACTGGAACAGCCAGAAGGAGCTCCTGGAGGATCGGCGGGCCCAGGTGGACACGTAC

>GU332163_Rhabdomys_pumilio_MHC_class_II_antigen_(Rhpu-DRB)_gene_Rhpu-DRB*154_allele_exon_2_and_partial_cds

CAGCACGTGCGGCTTCTGGACAGATACTTCTACAACCGGGAGGAGTTCGTGCGCTTCGACAGCGACGTGGGCGAGTACCGCGCGGTGACCGAGCTGGGGCGGCCGGACGCCGAGCACTGGAACAGCCAGAAGGAGTTCCTGGAGCATAGGCGGGCCGCGGTGGACACGTAC

>GU332164_Rhabdomys_pumilio_MHC_class_II_antigen_(Rhpu-DRB)_gene_Rhpu-DRB*155_allele_exon_2_and_partial_cds

CAGCACGTGCGGCTTCTGGACAGATACTTCTACAACCGGGAGGAGTACGTGCGCTTCGACAGCGACGTGGGCGAGCACCGCGCGGTGACCGAGCTGGGGCGGCCGGACGCTGATTACTGGAACAGCCAGAAGGAGTTCCTGGAGCAGAGGCGGGCCGCGGTGGACACGTAC

>GU332165_Rhabdomys_pumilio_MHC_class_II_antigen_(Rhpu-DRB)_gene_Rhpu-DRB*156_allele_exon_2_and_partial_cds

CAGCGCGTGCGGTTTCTGGACAGATACATCNACAACCGGGAGGAGTTCGTGCGCTTCGACAGCGACGTGGGCGAGCACCGCGCGGTGACCGAGCTGGGGCGGCGCATNGCTGAGTACTGGAACAGCCAGAAGGAGCTCCTGGAGGATAGGCGGGCCCAGGTGGACACGTAC

>GU332166_Rhabdomys_pumilio_MHC_class_II_antigen_(Rhpu-DRB)_gene_Rhpu-DRB*157_allele_exon_2_and_partial_cds

CAGCGCGTGCGGTTTCTGTTCAGAGACATCTACAACCGGGAGGAGCATGTGCGCTTCGACAGCGACGTGGGCGAGTNCCGCGCGGTGACCGAGCTGGGGCGGCCGGACGCTGAGTATTGGAACAGCCAGAAGGAGCTCCTGGAGCATAGGCGGGCCCAGGTGGACACGTAC

>GU332167_Rhabdomys_pumilio_MHC_class_II_antigen_(Rhpu-DRB)_gene_Rhpu-DRB*158_allele_exon_2_and_partial_cds

CAGCACGTGAGGTTTCTGGAGAGACACATCTACAACCGGGAGGAGTTCATGCGCTTCGACAGCGACGTGGGCGAGTACCGCACTGTGACCGAGCTGGGGCGGCGCATAGCTGAGGACTGGAACAGCCAGAAGGAGCTCCCGGAGCAGAAGCGGGCCCAGGTGGACGCGTAC

>GU332168_Rhabdomys_pumilio_MHC_class_II_antigen_(Rhpu-DRB)_gene_Rhpu-DRB*159_allele_exon_2_and_partial_cds

CAGCGCGTGCGGNTTCTGGTCAGATACATCTACAACCGTCAGGAGTACGTGCGCTTCGACAGCGACGTGGGCGAGTTCCGCGCGGTGACCGAGCTGGGGCGGCGGAGTGAGGAATACTGGAACAGCCAGAAGGAGATCCTGGAGCAGAAGCGGGCCGAGGTGGACACGGTC

>GU332169_Rhabdomys_pumilio_MHC_class_II_antigen_(Rhpu-DRB)_gene_Rhpu-DRB*160_allele_exon_2_and_partial_cds

CAGCGCGTGCGGTTTCTGGAGAGATACTTCTACAACCGGGAGGAGTACGTGCGCTTCGACAGCGACGTGGGCGAGTACCGCGCGGTGACCGAGCTGGGGCGGCGGGACGCTGAGTACTGGAACAGCCAGAAGGAGATCCTGGAGGATGCGCGGGCCGAGGTGGACACGTAC

>GU332170_Rhabdomys_pumilio_MHC_class_II_antigen_(Rhpu-DRB)_gene_Rhpu-DRB*161_allele_exon_2_and_partial_cds

CAGCACGTGCGGCTTCTGGACAGATACTTCTACAACCGGGAGGAGTTCGTGCGCTTCGACAGCGACGTGGGCGAGTACCGCGCGGTGACCGAGCTGGGGCGGCCGGACGCCGAGCACTGGAACAGCCAGAAGGAGTTCCTGGAGCAGAGGCGGGCCGCGGTGGACACGTAC

>GU332171_Rhabdomys_pumilio_MHC_class_II_antigen_(Rhpu-DRB)_gene_Rhpu-DRB*162_allele_exon_2_and_partial_cds

CAGCGCGTGCGGTTTCTGGACAGATACTTCTACAACCGGGAGGAGTACGTGCGCTTCGACAGCGACGTGGGCGAGNNCCGCGCGGTGACCGAGCTGGGGCGGCCGGACGCTGAGTACTGGAACAGCCAGAAGGAGCTCCTGGAGGATAGGCGGGCCCAGGTGGACACGTAC

>GU332172_Rhabdomys_pumilio_MHC_class_II_antigen_(Rhpu-DRB)_gene_Rhpu-DRB*163_allele_exon_2_and_partial_cds

CAGCGCGTGCGGTTTCTGGACAGATACTTCTACAACCGGGAGGAGTACGCGCGCTTCGACAGCGACGTGGGCGAGTACCGCGCGGTGACCGAGCTGGGGCGGCGCACAGCCAAGTACTGGAACAGCCAGAAGGAGTTCCTGGAGCATGCACGGGCCCAGGTGGACACGTAC

>GU332173_Rhabdomys_pumilio_MHC_class_II_antigen_(Rhpu-DRB)_gene_Rhpu-DRB*164_allele_exon_2_and_partial_cds

CAGCGCGTGCGGTTTCTGGACAGATACTTCTACAACCGGGAGGAGTACGTGCGCTTCGACAGCGACGTGGGCGAGTACCGCGCGGTGACCGAGCTGGGCGGGCGCACAGCCAAGTACTGGAACAGCCAGAAGGAGCTCCTGGAGCAGAAGCGGGCCCAGGTGGACGCGTAC

>GU332174_Rhabdomys_pumilio_MHC_class_II_antigen_(Rhpu-DRB)_gene_Rhpu-DRB*165_allele_exon_2_and_partial_cds

CAGCACGTGCGGCTTCTGGACAGATACTTCTACAACCGGGAGGAGTACGCGCGCTTCGACAGCGACGTGGGCGAGTTCCGCGCGGTGACCGAGCTGGGGCGGCGCACAGCCAAGTACTGGAACAGCCAGAAGGAGCTCCTGGAGCAGAAGCGGGCCGCGGTGGACACGTAC

>GU332175_Rhabdomys_pumilio_MHC_class_II_antigen_(Rhpu-DRB)_gene_Rhpu-DRB*166_allele_exon_2_and_partial_cds

CAGCACGTGCGGTTTCTGGACAGATACATCCACAACCGGGAGGAGTTCGTGCGCTTCGACAGCGACGTGGGCGAGTACCGCGCGGTGACCGAGCTGGGGCGGGGCATAGCTGAGGACTTGAACAGCCAGAAGGAGTTCCTGGAGCGGAAGCGGGCCGAGCTGGACACGTAC

>GU332176_Rhabdomys_pumilio_MHC_class_II_antigen_(Rhpu-DRB)_gene_Rhpu-DRB*167_allele_exon_2_and_partial_cds

CAGCGCGTGCGGTTTCTGGACAGATACTTCTACAACCGGGAGGAGTACGTGCGCTTCGACAGCGACGTGGGCGAGTTCCGCGCGGTGACCGAGCTGGGGCGGCCGGACGCTGAGTACTGGAACAGCCAGAAGGAGTTCCTGGAGGATCGGCGGGCCCAGGTGGACACAGTG

>GU332177_Rhabdomys_pumilio_MHC_class_II_antigen_(Rhpu-DRB)_gene_Rhpu-DRB*168_allele_exon_2_and_partial_cds

CAGCGCGTGCGGTTTCTGGACAGATACATCCACAACCGGGAGGAGTTCGTGCGCTTCGACAGCGACGTGGGCGAGCACCGCGCGGTGACCGAGCTGGGGCGGCGCATAGCTGAGGACTGGAACAGCCAGAAGGAGTTCCTGGAGCATAAGCGGGCCCGGGTGGACACGTAC

>GU332178_Rhabdomys_pumilio_MHC_class_II_antigen_(Rhpu-DRB)_gene_Rhpu-DRB*169_allele_exon_2_and_partial_cds

CAGCGCGTGCGGTTTCTGGACAGATACTTCTACAACCGGGAGGAGTACGCGCGCTTCGACAGCGACGTGGGCGAGTTCCGCGCGGTGACCGAGCTGGGGCGGCCGGACGCTGAGTACTGGAACAGCCAGAAGGAGCTCCTGGAGCAGAAGCGGGCCGAGCTGGACACGTAC

>GU332179_Rhabdomys_pumilio_MHC_class_II_antigen_(Rhpu-DRB)_gene_Rhpu-DRB*170_allele_exon_2_and_partial_cds

CAGCGCGTGCGGTTTCTGGACAGATACATCCACAACCGGGAGGAGTTCGTGCGCTTCGACAGCGACGTGGGCGAGCACCGCGCGGTGACCGAGCTGGGGCGGCGCATAGCTGAGGACTGGAACAGCCAGAAGGAGCTCCTGGAGGATCGGCGGGCCCAGGTGGACGCATAC

>GU332180_Rhabdomys_pumilio_MHC_class_II_antigen_(Rhpu-DRB)_gene_Rhpu-DRB*171_allele_exon_2_and_partial_cds

CAGCGCGTGCGGTATCTGCACAGATATTTCTACAACCGTCAGGAGTACGTGCGCTTCGACAGCGACGTGGGCGAGTTCCGCGCGGTGACCGAGCTGGGGCGGCCGATCGAGGAATACTGGAACAGCCAGAAGGAGCTCCTGGAGCGGAAGCGGGCCGAGCTGGACACGTAC

>GU332181_Rhabdomys_pumilio_MHC_class_II_antigen_(Rhpu-DRB)_gene_Rhpu-DRB*172_allele_exon_2_and_partial_cds

CAGCGCGTGCGGTTTCTGGACAGACACATCCACAACCGGGAGGAGTACGTGCGCTTCGACAGCGACGTGGGCGAGTTCCGCGCGGTGACCGAGCTGGGGCGGCCGGACGAGGAATACTGGAACAGCCAGAAGGACTTCCTGGAGCATAAGCGGGCCCAGGTGGACGCATAC

>GU332182_Rhabdomys_pumilio_MHC_class_II_antigen_(Rhpu-DRB)_gene_Rhpu-DRB*173_allele_exon_2_and_partial_cds

CAGCGCGTGCGGTTTCTGGAGAGACACATCTACAACCGGGAGGAGTTCATGCGCTTCGACAGCGACGTGGGCGAGTACCGCACTGTGACCGAGCTGGGGCGGCGCATAGCTGAGGACTGGAACAGCCAGAAGGAGTACCTGGAGGATCGGCGGGCCCAGGTGGACGCGTAC

>GU332183_Rhabdomys_pumilio_MHC_class_II_antigen_(Rhpu-DRB)_gene_Rhpu-DRB*174_allele_exon_2_and_partial_cds

CAGCGCGTGCGGTTTCTGGAGAGATACATCTACAACCGGGAGGAGTACGCGCGCTTCGACAGCGACGTGGGCGAGTACCGCGCGGTGACCGAGCTGGGGCGGCGCACAGCCAAGTACTGGAACAGCCAGAAGGAGCTCCTGGAGCATAAGCGGGCCCGGGTGGACACGTAC

>GU332184_Rhabdomys_pumilio_MHC_class_II_antigen_(Rhpu-DRB)_gene_Rhpu-DRB*175_allele_exon_2_and_partial_cds

CAGCACGTGCGGTTTCTGGACAGATACTTCTACAACCGGGAGGAGTACGCGCGCTTCGACAGCGACGTGGGCGAGTACCGCGCGGTGACCGAGCTGGGGCGGCCGGACGCTGAGTACTGGAACAGCCAGAAGGAGTTCCTGGAGGATGCGCGGGCCGAGGTGGACACGTAC

>GU332185_Rhabdomys_pumilio_MHC_class_II_antigen_(Rhpu-DRB)_gene_Rhpu-DRB*176_allele_exon_2_and_partial_cds

CAGCGCGTGCGGTTTCTGGAGAGATACTTCTACAACCGGGAGGAGTACGCGCGCTTCGACAGCGACGTGGGCGAGTACCGCGCGGTGACCGAGCTGGGGCGGCGCACAGCCAAGTACTGGAACAGCCAGAAGGAGTACCTGGAGGATGCGCGGGCCGCGGTGGACACGTAC

>GU332186_Rhabdomys_pumilio_MHC_class_II_antigen_(Rhpu-DRB)_gene_Rhpu-DRB*177_allele_exon_2_and_partial_cds

CAGCGCGTGCGGTTTCTGGACAGATACATCCACAACCGGGAGGAGTTCGTGCGCTTCGACAGCGACGTGGGCGAGTACCGCGCGGTGACCGAGCTGGGGCGGGGCATAGCTGAGGACTTGAACAGCCAGAAGGAGTTCCTGGAGCAGAGGCGGGCCGCGGTGGACACGTAC

>GU332187_Rhabdomys_pumilio_MHC_class_II_antigen_(Rhpu-DRB)_gene_Rhpu-DRB*178_allele_exon_2_and_partial_cds

CAGCGCGTGCGGTTTCTGGACAGATACTTCTACAACCGGGAGGAGTACGCGCGCTTCGACAGCGACGTGGGCGAGTACCGCGCGGTGACCGAGCTGGGGCGGCCGGACGCTGAGTACTGGAACAGCCAGAAGGAGCTCCTGGAGCAGAAGCGGGCCCGGGTGGACACGTAC

>GU332188_Rhabdomys_pumilio_MHC_class_II_antigen_(Rhpu-DRB)_gene_Rhpu-DRB*179_allele_exon_2_and_partial_cds

CAGCGCGTGCGGTTTCTGGAGAGATACTTCTACAACCGGGAGGAGTACGCGCGCTTCGACAGCGACGTGGGCGAGTACCGCGCGGTGACCGAGCTGGGGCGGCGGGACGCTGAGCACTGGAACAGCCAGAAGGAGATCCTGGAGCGGAGGCGGGCCGCGGTGGACACGTAC

>GU332189_Rhabdomys_pumilio_MHC_class_II_antigen_(Rhpu-DRB)_gene_Rhpu-DRB*180_allele_exon_2_and_partial_cds

CAGCGCGTGCGGTTTCTGGAGAGACACATCTACAACCGGGAGGAGTTCATGCGCTTCGACAGCGACGTGGGCGAGTACCGCGCGGTGACCGAGCTGGGGCGGCGCATAGCTGAGGACTTCAACAGCCGGAAGGAGATCCTGGAGGATGCGCGGGCCCAGGTGGACACGTAC

>GU332190_Rhabdomys_pumilio_MHC_class_II_antigen_(Rhpu-DRB)_gene_Rhpu-DRB*181_allele_exon_2_and_partial_cds

CAGCGCGTGCGGTTTCTGGAGAGACACATCTACAACCGGGAGGAGTTCATGCGCTTCGACAGCGACGTGGGCGAGTACCGCGCGGTGACCGAGCTGGGGCGGCGCATAGCTGAGGACTTCAACAGCCGGAAGGAGCTCCTGGAGCAGAGGCGGGCCGCGGTGGACACGTAC

>GU332191_Rhabdomys_pumilio_MHC_class_II_antigen_(Rhpu-DRB)_gene_Rhpu-DRB*182_allele_exon_2_and_partial_cds

CAGCGCGTGCGGTTTCTGGAGAGACACATCTACAACCGGGAGGAGTACGCGCGCTTCGACAGCGACGTGGGCGAGTACCGCGCGGTGACCGAGCTGGGGCGGCGCATAGCTGAGGACTTCAACAGCCGGAAGGAGATCCTGGAGGATGCGCGGGCCGCGGTGGACACGTAC

>GU332192_Rhabdomys_pumilio_MHC_class_II_antigen_(Rhpu-DRB)_gene_Rhpu-DRB*183_allele_exon_2_and_partial_cds

CAGCGCGTGCGGTTTCTGGAGAGATACTTCTACAACCGGGAGGAGTACGTGCGCTTCGACAGCGACGTGGGCGAGTTCCGCGCGGTGACCGAGCTGGGGCGGCCGGACGCTGAGTACTGGAACAGCCAGAAGGAGTTCCTGGAGGATCGGCGGGCCCAGGTGGACACAGTG

>GU332193_Rhabdomys_pumilio_MHC_class_II_antigen_(Rhpu-DRB)_gene_Rhpu-DRB*184_allele_exon_2_and_partial_cds

CAGCACGTGCGGTTTCTGGACAGATACTTCTACAACCGGGAGGAGTTCGTGCGCTTCGACAGCGACGTGGGCGAGTACCGCGCGGTGACCGAGCTGGGGCGGGGCATAGCTGAGGACTGGAACAGCCAGAAGGAGTTCCTGGAGCAGAGGCGGGCCGCGGTGGACACGTAC

>GU332194_Rhabdomys_pumilio_MHC_class_II_antigen_(Rhpu-DRB)_gene_Rhpu-DRB*185_allele_exon_2_and_partial_cds

CAGCGCGTGCGGTTTCTGGAGAGATACTTCTACAACCGGGAGGAGTACGCGCGCTTCGACAGCGACGTGGGCGAGTACCGCGCGGTGACCGAGCTGGGGCGGCGGGACGCTGAGTACTGGAACAGCCAGAAGGAGGAACTGGAGCAGAAGCGGGCCCAGGTGGACGCGTAC

>GU332195_Rhabdomys_pumilio_MHC_class_II_antigen_(Rhpu-DRB)_gene_Rhpu-DRB*186_allele_exon_2_and_partial_cds

CAGCGCGTGCGGTTTCTGGACAGATACTTCTACAACCGGGAGGAGTACGCGCGCTTCGACAGCGACGTGGGCGAGTACCGCGCGGTGACCGAGCTGGGGCGGCGGGACGCCGAGTACTGGAACAGCCAGAAGGAGCTCCTGGAGCAGAGGCGGGCCCAGGTGGACACAGTG

>GU332196_Rhabdomys_pumilio_MHC_class_II_antigen_(Rhpu-DRB)_gene_Rhpu-DRB*187_allele_exon_2_and_partial_cds

CAGCGCGTGCGGTTTCTGGACAGATACTTCTACAACCGGGAGGAGTACGCGCGCTTCGACAGCGACGTGGGCGAGTACCGCGCGGTGACCGAGCTGGGGCGGCGGGACGCCGAGTACTGGAACAGCCAGAAGGAGCTCCTGGAGCAGAGGCGGGCCCAGGTGGACGCGTAC

>GU332197_Rhabdomys_pumilio_MHC_class_II_antigen_(Rhpu-DRB)_gene_Rhpu-DRB*188_allele_exon_2_and_partial_cds

CAGCGCGTGCGGTTTCTGGAGAGACACATCTACAACCGGGAGGAGTTCATGCGCTTCGACAGCGACGTGGGCGAGTACCGCGCGGTGACCGAGCTGGGGCGGCGCATAGCTGAGGACTTCAACAGCCGGAAGGAGATCCTGGAGGATGCGCGGGCCGCGGTGGACACGTAC

>GU332198_Rhabdomys_pumilio_MHC_class_II_antigen_(Rhpu-DRB)_gene_Rhpu-DRB*189_allele_exon_2_and_partial_cds

CAGCGCGTGCGGTTTCTGGACAGATACTTCTACAACCGGGAGGAGTACGTGCGCTTCGACAGCGACGTGGGCGAGCACCGCGCGGTGACCGAGCTGGGGCGGCCGGACGCTGAGTACTGGAACAGCCAGAAGGAGCTCCTGGAGGATGCGCGGGCCCAGGTGGACACGTAC

>GU332199_Rhabdomys_pumilio_MHC_class_II_antigen_(Rhpu-DRB)_gene_Rhpu-DRB*190_allele_exon_2_and_partial_cds

CAGCACGTGAGGTTTCTGGAGAGACACATCTACAACCGGGAGGAGTTCATGCGCTTCGACAGCGACGTGGGCGAGTTCCGCACTGTGACCGAGCTGGGGCGGCGCATAGCTGAGGACTGGAACAGCCAGAAGGAGTACCTGGAGGATAGGCGGGCCCAGGTGGACGCGTAC

>GU332200_Rhabdomys_pumilio_MHC_class_II_antigen_(Rhpu-DRB)_gene_Rhpu-DRB*191_allele_exon_2_and_partial_cds

CAGCGCGTGCGGTTTCTGGAGAGATACTTCTACAACCGGGAGGAGTTCGCGCGCTTCGACAGCGACGTGGGCGAGTACCGCGCGGTGACCGAGCTGGGGCGGCGGGACGCCGAGTACTGGAACAGCCAGAAGGAGTTCCTGGAGCATAGGCGGGCCGCGGTGGACACGTAC

>GU332201_Rhabdomys_pumilio_MHC_class_II_antigen_(Rhpu-DRB)_gene_Rhpu-DRB*192_allele_exon_2_and_partial_cds

CAGCGCGTGGGGTTTCTGGACAGACACATCCACAACCGGGAGGAGTTCGTGCGCTTCGACAGCGACGTGGGCGAGTTCCGCGCTGTGACCGAGCTGGGGCGGCGCATAGCTGAGGACTGGAACAGCCAGAAGGAGTTCCTGGAGGATACGCGGGCCGGGGTGGACGCGTAC

>GU332202_Rhabdomys_pumilio_MHC_class_II_antigen_(Rhpu-DRB)_gene_Rhpu-DRB*193_allele_exon_2_and_partial_cds

CAGCGCGTGCGGTTTCTGGAGAGACACATCTACAACCGGGAGGAATTCATGCGCTTCGACAGCGACGTGGGCGAGTACCGCGCGGTGACCGAGCTGGGGCGGCGCATAGCTGAGGACTTCAACAGCCGGAAGGAGCTCCTGGAGCGGAAGCGGGCCGCGGTGGACACGTAC

>GU332203_Rhabdomys_pumilio_MHC_class_II_antigen_(Rhpu-DRB)_gene_Rhpu-DRB*194_allele_exon_2_and_partial_cds

CAGCGCGTGCGGTTTCTGGACAGATACTTCTACAACCGGGAGGAGTACGCGCGCTTCGACAGCGACGTGGGCGAGTACCGCGCGGTGACCGAGCTGGGGCGGCGCACAGCTAAGTACTGGAACAGCCAGAAGGAGCTCCTGGAGCAGAAGCGGGCCCAGGTGGACGCGTAC

>GU332204_Rhabdomys_pumilio_MHC_class_II_antigen_(Rhpu-DRB)_gene_Rhpu-DRB*195_allele_exon_2_and_partial_cds

CAGCGCGTGCGGTTTCTGGAGAGATACTTCTACAACCGGGAGGAGTACGCGCGCTTCTACAGCGACGTGGGCGAGTTCCGCGCGGTGACCGAGCTGGGGCGGCCGGACGCTGAGTACTGGAACAGCCAGAAGGAGTTCCTGGAGGATGCGCGGGCCCAGGTGGACAACTAC

>GU332205_Rhabdomys_pumilio_MHC_class_II_antigen_(Rhpu-DRB)_gene_Rhpu-DRB*196_allele_exon_2_and_partial_cds

CAGCGCGTGCGGTTTCTGGACAGATACTTCTACAACCGGGAGGAGTACGCGCGCTTCGACAGCGACGTGGGCGAGTACCGCGCGGTGACCGAGCTGGGGCGGCGCACAGCCAAGTACTGGAACAGCCAGAAGGAGCTCCTGGAGCATGAGCGGGCCCGGGTGGACACGTAC

>GU332206_Rhabdomys_pumilio_MHC_class_II_antigen_(Rhpu-DRB)_gene_Rhpu-DRB*197_allele_exon_2_and_partial_cds

CAGCGCGTGCGGCTTCTGGAGAGACACATCTACAACCGGGAGGAGTTCATGCGCTTCGACAGCGACGTGGGCGAGTACCGCGCGGTGATCGAGCTGGGGCGGCGCATAGCTGAGGACTTCAACAGCCGGAAGGAGATCCTGGAGGATGCGCGGGCCCAGGTGGACACGTAC

>GU332207_Rhabdomys_pumilio_MHC_class_II_antigen_(Rhpu-DRB)_gene_Rhpu-DRB*198_allele_exon_2_and_partial_cds

CAGCGCGTGCGGTATCTGCAGAGATATTTCTACAACCGTCAGGAGTACGTGCGCTTCGACAGCGACGTGGGCGAGTTCCGCGCGGTGACCGAGCTGGGGCGGCCGGACGAGGAATACTGGAACAGCCAGAAGGACTTCCTGGAGCAGAAGCGGTCACAGGTGGACGCATAC

>GU332208_Rhabdomys_pumilio_MHC_class_II_antigen_(Rhpu-DRB)_gene_Rhpu-DRB*199_allele_exon_2_and_partial_cds

CAGCGCGTGCGGTTTCTGGACAGATACTTCTACAACCGGGAGGAGTTCATGCGCTTCGACAGCGACGTGGGCGAGNACCGCGCGGTGACCGAGCTGGGGCGGCGCCACGCTGAGGACTGGAACAGCCAGAAGGAGCTCCTGGAGGATACCCGGGCCCAGGTGGACACGTAC

>GU332209_Rhabdomys_pumilio_MHC_class_II_antigen_(Rhpu-DRB)_gene_Rhpu-DRB*200_allele_exon_2_and_partial_cds

CAGCGCGTGCGGTTTCTGATCAGATACTTCTACAACCGGGAGGAGAACGTGCGCTTCGACAGCGACGTGGGCGAGTACCGCGCGGTGACCGAGCTGGGGCGGCGGGACGCTGATTACTGGAACAGCCAGAAGGAGCTCCTGGAGCAGAGGCGGGCCCAGGTGGACACGTAC

>GU332210_Rhabdomys_pumilio_MHC_class_II_antigen_(Rhpu-DRB)_gene_Rhpu-DRB*201_allele_exon_2_and_partial_cds

CAGCGCGTGAGGTTTCTGGAGAGACACATCTACAACCGGGAGGAGTTCATGCGCTTCGACAGCGACGTGGGCGAGTACCGCACTGTGACCGAGCTGGGGCGGCGCATAGCTGAGGACTGGAACAGCCAGAAGGAGTACCTGGAGGATCGGCGGGCCCAGGTGGACGCGTAC

>GU332211_Rhabdomys_pumilio_MHC_class_II_antigen_(Rhpu-DRB)_gene_Rhpu-DRB*202_allele_exon_2_and_partial_cds

CAGCTCGTGCGGTTTCTGGAGAGATACTTCTACAACCGGGAGGAGTACGCGCGCTTCGACAGCGACGTGGGCGAGTACCGCGCGGTGACCGAGCTGGGGCGGCGCACAGCCAAGTACTGGAACAGCCAGAAGGAGCTCCTGGAGCGGAAGCGGGCCCAGGTGGACGCGTAC

>GU332212_Rhabdomys_pumilio_MHC_class_II_antigen_(Rhpu-DRB)_gene_Rhpu-DRB*203_allele_exon_2_and_partial_cds

CAGCGCGTGCGGTTTCTGCAGAGATATTTCTACAACCGTCAGGAGTTCGTGCGCTTCGACAGCGACGTGGGCGAGTTCCGCGCGGTGACCGAGCTGGGGCGGCCGGTCGATGAATACTGGAACAGCCAGAAGGACTTCCTGGAGCATAAGCGGTCCCAGGTGGACGCATAC

>GU332213_Rhabdomys_pumilio_MHC_class_II_antigen_(Rhpu-DRB)_gene_Rhpu-DRB*204_allele_exon_2_and_partial_cds

CAGCGCGTGCGGTTTCTGGTCAGATATTTCTACAACCGTCAGGAGTACGTGCGCTTCGACAGCGACGTGGGCGAGTTCCGCGCGGTGACCGAGCTGGGGCGGCGGANTGAGGAATACTGGAACAGCCAGAAGGAGTTCCTGGAGCATAAGCGGTCACAGGTGGACACGTAC

>GU332214_Rhabdomys_pumilio_MHC_class_II_antigen_(Rhpu-DRB)_gene_Rhpu-DRB*205_allele_exon_2_and_partial_cds

CAGCACGTGAGGTTTCTGGAGAGACACATCTACAACCGGGAGGAGTTCATGCGCTTCGACAGCGACGTGGGCGAGTACCGCACTGTGACCGAGCTGGGGCGGCGCATAGCTGAGGACTGGAACAGCCAGAAGGAGTACCTGGAGGATCGGCGGGCCCAGGTGGACACGTAC

>GU332215_Rhabdomys_pumilio_MHC_class_II_antigen_(Rhpu-DRB)_gene_Rhpu-DRB*206_allele_exon_2_and_partial_cds

CAGCTCGTGCGGTTTCTGGAGAGATACTTCTACAACCGGGAGGAGTACGCGCGCTTCGACAGCGACGTGGGCGAGTACCGCGCGGTGACCGAGCTGGGGCGGCGGGACGCTGAGTACTGGAACAGCCAGAAGGAGATCCTGGAGCAGAAGCGGGCCCAGGTGGACGCGTAC

>GU332216_Rhabdomys_pumilio_MHC_class_II_antigen_(Rhpu-DRB)_gene_Rhpu-DRB*207_allele_exon_2_and_partial_cds

CAGCGCGTGCGGTTTCTGGAGAGATACTTCTACAACCGGGAGGAGTACGCGCGCTTCGACAGCGACGTGGGCGAGTACCGCGCGGTGACCGAGCTGGGGCGGCCGGACGCTGTATACTGGAACAGCCAGAAGGAGCTCCTGGAGCAGAGGCGGGCCCAGGTGGACACGTAC

>GU332217_Rhabdomys_pumilio_MHC_class_II_antigen_(Rhpu-DRB)_gene_Rhpu-DRB*208_allele_exon_2_and_partial_cds

CAGCGCGTGCGGTTTCTGGAGAGATACTTCTACAACCGGGAGGAGTACGTGCGCTTCGACAGCGACGTGGGCGAGTACCGCGCGGTGACCGAGCTGGGGCGGCGGGACGCCGAGAACTGGAACAGCCAGAAGGAGATCCTGGAGCGGAAGCGGGCCGCGGTGGACGCGTAC

>GU332218_Rhabdomys_pumilio_MHC_class_II_antigen_(Rhpu-DRB)_gene_Rhpu-DRB*209_allele_exon_2_and_partial_cds

CAGCGCGTGCGGTTTCTGGACAGATACTTCTACAACCGGGAGGAGTTCGTGCGCTTCGACAGCGACGTGGGCGAGTACCGCGCGGTGACCGAGCTGGGGCGGCCGGACGCCGAGCACTGGAACAGCCAGAAGGAGTTCCTGGAGCAGAGGCGGGCCGCGGTGGACACGTAC

>GU332219_Rhabdomys_pumilio_MHC_class_II_antigen_(Rhpu-DRB)_gene_Rhpu-DRB*210_allele_exon_2_and_partial_cds

CAGCACGTGCGGTTTCTGGACAGATACTTCTACAACCGGGAGGAGTACGCGCGCTTCGACAGCGACGTGGGCGAGTACCGCGCGGTGACCGAGCTGGGGCGGCCGGACGCTGAGTATTGGAACAGCCAGAAGGAGATCCTGGAGGATGCGCGGGCCGAGGTGGACACGTAC

>GU332220_Rhabdomys_pumilio_MHC_class_II_antigen_(Rhpu-DRB)_gene_Rhpu-DRB*211_allele_exon_2_and_partial_cds

CAGCACGTGAGGTTTCTGGAGAGACACATCTACAACCGGGAGGAGTTCGTGCGCTTCGACAGCGACGTGGGCGAGTACCGCGCGGTGACCGAGCTGGGGCGGCGCATAGCTGAGGACTTCAACAGCCGGAAGGAGATCCTGGAGGATGCGCGGGCCGCGGTGGACACGTAC

>GU332221_Rhabdomys_pumilio_MHC_class_II_antigen_(Rhpu-DRB)_gene_Rhpu-DRB*212_allele_exon_2_and_partial_cds

CAGCGCGTGCGGTTTCTGGACAGATACTTCTACAACCGGGAGGAGTACGTGCGCTTCGACAGCGACGTGGGCGAGTTCCGCGCGGTGACCGAGCTGGGGCGGCCGGACGAGGAATACTGGAACAGCCGGAAGGATCTCCTGGAGCGGAAGCGTGCAGAGGTGGACACGTAC

>GU332222_Rhabdomys_pumilio_MHC_class_II_antigen_(Rhpu-DRB)_gene_Rhpu-DRB*213_allele_exon_2_and_partial_cds

CAGCGCGTGCGGTTTCTGGAGAGACACTTCTACAACCGGGAGGAGTACGCGCGCTTCGACAGCGACGTGGGCGAGTACCGCGCGGTGACCGAGCTGGGGCGGCGCACAGCCAAGTACTGGAACAGCCAGAAGGAGGTCCTGGAGGATGCGCGGGCCGCGGTGGACACGTAC

>GU332223_Rhabdomys_pumilio_MHC_class_II_antigen_(Rhpu-DRB)_gene_Rhpu-DRB*214_allele_exon_2_and_partial_cds

CAGCGCGTGCGGTTTCTGGACAGACACTTCTCCAACCGGGAGGAGTTCGTGCGCTTCGACAGCGACGTGGGCGAGCACCGCGCGGTGACCGAGCTGGGGCGGCGCATAGCTGAGGACTGGAACAGCCAGAAGGAGCTCCTGGAGCGGAAGCGGGCCGAGCTGGACACGTAC

>GU332224_Rhabdomys_pumilio_MHC_class_II_antigen_(Rhpu-DRB)_gene_Rhpu-DRB*215_allele_exon_2_and_partial_cds

CAGCGCGTGCGGTTTCTGGACATACACATCCACAACCGGGAGGAGTTCGTGCGCTTCGACAGCGACGTGGGCGAGCACCGCGCGGTGACCGAGCTGGGGCGGCGCATAGCTGAGGACTGGAACAGCCAGAAGGAGCTCCTGGAGCGGAAGCGGGCCGAGCTGGACACGTAC

>GU332225_Rhabdomys_pumilio_MHC_class_II_antigen_(Rhpu-DRB)_gene_Rhpu-DRB*216_allele_exon_2_and_partial_cds

CAGCGCGTGCGGTTTCTGGACAGATACTTCTACAACCGGGAGGAGTACGCGCGCTTCGACAGCGACGTGGGCGAGTACCGCGCGGTGACCGAGCTGGGGCGGCGGGACGCTGAGCACTGGAACAGCCAGAAGGAGATCCTGGAGCATAAGCGGGCCGCGGTGGACACGTAC

>GU332226_Rhabdomys_pumilio_MHC_class_II_antigen_(Rhpu-DRB)_gene_Rhpu-DRB*217_allele_exon_2_and_partial_cds

CAGCGCGTGCGGTTTCTGGAGACATACATCTACAACCGGGAGGAGTACGTGCGCTTCGACAGCGACGTGGGCGAGTACCGCGCGGTGACCGAGCTGGGGCGGGGCATAGCTGAGCACTGGAACAGCCAGAAGGAGTTCCTGGAGCAGAGGCGGGCCGCGGTGGACACGTAC

>GU332227_Rhabdomys_pumilio_MHC_class_II_antigen_(Rhpu-DRB)_gene_Rhpu-DRB*218_allele_exon_2_and_partial_cds

CAGCGCGTGCGGTTTCTGGACAGATACTTCTACAACCGGGAGGAGTACGCGCGCTTCGACAGCGACGTGGGCGAGTTCCGCGCGGTGACCGAGCTGGGGCGGCGCACAGCCAAGTACTGGAACAGCCAGAAGGAGCTCCTGGAGGATCGGCGTGCCGCGGTGGACACGTAC

>GU332228_Rhabdomys_pumilio_MHC_class_II_antigen_(Rhpu-DRB)_gene_Rhpu-DRB*219_allele_exon_2_and_partial_cds

CAGCGCGTGAGGTTTCTGGAGAGACACTTCTACAACCGGGAGGAGTTCATGCGCTTCGACAGCGACGTGGGCGAGTTCCGCACTGTGACCGAGCTGGGGCGGCGCATAGCTGAGGACTGGAACAGCCAGAAGGAGTACCTGGAGGATCGGCGGGCCCAGGTGGACGCGTAC

>GU332229_Rhabdomys_pumilio_MHC_class_II_antigen_(Rhpu-DRB)_gene_Rhpu-DRB*220_allele_exon_2_and_partial_cds

CAGCACGTGAGGTTTCTGGAGAGACACATCTACAACCGGGAGGAGTTCATGCGCTTCGACAGCGACGTGGGCGAGTTCCGCACTGTGACCGAGCTGGGGCGGCGCATAGCTGAGGACTGGAACAGCCAGAAGGAGTACCTGGAGGAGACGCGGGCCGAGGTGGACACGTAC

>GU332230_Rhabdomys_pumilio_MHC_class_II_antigen_(Rhpu-DRB)_gene_Rhpu-DRB*221_allele_exon_2_and_partial_cds

CAGCGCGTGCGGTTTCCGGAGAGACACATCTACAACCGGGAGGAGTTCATGCGCTTCGACAGCGACGTGGGCGAGTACCGCGCGGTGATCGAGCTGGGGCGGCGCATAGCTGAGGACTTCAACAGCCGGAAGGAGATCCTGGAGGATGCGCGGGCCCAGGTGGACACGTAC

>GU332231_Rhabdomys_pumilio_MHC_class_II_antigen_(Rhpu-DRB)_gene_Rhpu-DRB*222_allele_exon_2_and_partial_cds

CAGCGCGTGCGGCTTCTGGAGAGATACATCTACAACCGGGAGGAGTTCATGCGCTTCGACAGCGACGTGGGCGAGTACCGCACTGTGACCGAGCTGGGGCGGCGCATAGCTGAGGACTGGAACAGCCAGAAGGAGCTCCTGGAGCAGAAGCGGGCCCAGGTGGACGCGTAC

>GU332232_Rhabdomys_pumilio_MHC_class_II_antigen_(Rhpu-DRB)_gene_Rhpu-DRB*223_allele_exon_2_and_partial_cds

CAGCGCGTGCGGTTTCTGGACAGATACTTCTACAACCGGGAGGAGTACGCGCGCTTCGACAGCGACGTGGGCGAGTACCGCGCGGTGACCGAGCTGGGGCGGCCGGACGCTGAGTATTGGAACAGCCAGAAGGAGATCCTGGAGGATGCGCGGGCCCAGGTGGACACGTAC

>GU332233_Rhabdomys_pumilio_MHC_class_II_antigen_(Rhpu-DRB)_gene_Rhpu-DRB*224_allele_exon_2_and_partial_cds

CAGCGCGTGCGGTTTCTGGACAGATACATCCACAACCGGGAGGAGTTCGTGCGCTTCGACAGCGACGTGGGCGAGCACCGCGCGGTGACCGAGCTGGGGCGGCGCATAGCTGAGGACTGGAACAGCCAGAAGGAGCTCCTGGAGCGGAAGCGGGCCGCGGTGGACACGTAC

>GU332234_Rhabdomys_pumilio_MHC_class_II_antigen_(Rhpu-DRB)_gene_Rhpu-DRB*225_allele_exon_2_and_partial_cds

CAGCGCGTGCGGTTTCTGGACAGATACATCCACAACCGGGAGGAGTTCGTGCGCTTCGACAGCGACGTGGGCGAGCACCGCGCGGTGACCGAGCTGGGGCGGCGCATAGCTGAGGACTGGAACAGCCAGAAGGAGCTCCTGGAGGATGAGCGGGCCCGGGTGGACACGTAC

>GU332235_Rhabdomys_pumilio_MHC_class_II_antigen_(Rhpu-DRB)_gene_Rhpu-DRB*226_allele_exon_2_and_partial_cds

CAGCGCGTGCGGTTTCTGGACAGATACTTCTACAACCGGGAGGAGTACGTGCGCTTCGACAGCGACGTGGGCGAGTACCGCGCGGTGACCGAGCTGGGGCGGCCGGACGCTGAGGACTGGAACAGCCAGAAGGAGATCCTGGAGGATGCGCGGGCCGCGGTGGACACGTAC

>GU332236_Rhabdomys_pumilio_MHC_class_II_antigen_(Rhpu-DRB)_gene_Rhpu-DRB*227_allele_exon_2_and_partial_cds

CAGCACGTGCGGCTTCTGGACAGATACTTCTACAACCGGGAGGAGTTCGTGCGCTTCGACAGCGACGTGGGCGAGTACCGCGCGGTGACCGAGCTGGGGCGGCCGGACGCCGAGCACTGGAACAGCCAGAAGGAGTTCCTGGAGCAGCAGCGGGCCCAGGTGGACACGTAC

>GU332237_Rhabdomys_pumilio_MHC_class_II_antigen_(Rhpu-DRB)_gene_Rhpu-DRB*228_allele_exon_2_and_partial_cds

CAGCGCGTGCGGTTTCTGGAGAGATATTTCTACAACCGTCAGGAGTACGTGCGCTTCGACAGCGACGTGGGCGAGTTCCGCGCGGTGACCGAGCTGGGGCGGCGCACAGCCAAGTACTGGAACAGCCAGAAGGAGATCCTGGAGCAGAAGCGGGCCGCGGTGGACACGTAC

>GU332238_Rhabdomys_pumilio_MHC_class_II_antigen_(Rhpu-DRB)_gene_Rhpu-DRB*229_allele_exon_2_and_partial_cds

CAGCACGTGCGGCTTCTGGACAGATACTTCTACAACCGGGAGGAGTTCGTGCGCTTCGACAGCGACGTGGGCGAGTACCGCGCGGTGACCGAGCTGGGGCGGCGGGACGCCGAGCACTGGAACAGCCAGAAGGAGTTCCTGGAGCGGAGGCGGTCACAGGTGGACGCATAC

>GU332239_Rhabdomys_pumilio_MHC_class_II_antigen_(Rhpu-DRB)_gene_Rhpu-DRB*230_allele_exon_2_and_partial_cds

CAGCGCGTGCGGTTTCTGGAGAGACACATCTACAACCGGGAGGAGTTCGTGCGCTTCGACAGCGACGTGGGCGAGTACCGCGCGGTGACCGAGCTGGGGCGGCGCATAGCTGAGGACTTCAACAGCCGGAAGGAGCTCCTGGAGCGGAAGCGGTCCCAGGTGGACACGTAC

>GU332240_Rhabdomys_pumilio_MHC_class_II_antigen_(Rhpu-DRB)_gene_Rhpu-DRB*231_allele_exon_2_and_partial_cds

CAGCGCGTGCGGTTTCTGGAGAGATACTTCTACAACCGGGAGGAGTACGCGTGCTTCGACAGCGACGTGGGCGAGTACCGCGCGGTGACCGAGCTGGGGCGGCGGGACGCCGAGTACTGGAACAGCCAGAAGGAGCTCCTGGAGCAGAGGCGGTCCCAGGTGGACACGTAC

>GU332241_Rhabdomys_pumilio_MHC_class_II_antigen_(Rhpu-DRB)_gene_Rhpu-DRB*232_allele_exon_2_and_partial_cds

CAGCGCGTGCGGTTTCTGGAGAGATACTTCTACAACCGGGAGGAGTACGCGCGCTTCGACAGCGACGTGGGCGAGTACCGCGCGGTGACCGAGCTGGGGCGGCGCACGGCCAAGTATTGGAACAGCCAGAAGGAGGTCCTGGAGGATGCGCGGGCCGCGGTGGACACGTAC

>GU332242_Rhabdomys_pumilio_MHC_class_II_antigen_(Rhpu-DRB)_gene_Rhpu-DRB*233_allele_exon_2_and_partial_cds

CAGCGCGTGCGGTTTCTGGACAGATACATCCACAACCGGGAGGAGTTCGTGCGCTTCGACAGCGACGTGGGCGAGCACCGCGCGGTGACCGAGCTGGGGCGGCGCATAGCTGAGGACTGGAACAGCCAGAAGGAGCTCCTGGAGGAGCAGCGGGCCCAGGTGGACACGTAC

>GU332243_Rhabdomys_pumilio_MHC_class_II_antigen_(Rhpu-DRB)_gene_Rhpu-DRB*234_allele_exon_2_and_partial_cds

CAGCACGTGCGGTTTCTGGACAGATACTTCTACAACCGGGAGGAGTACGCGCGCTTCGACAGCTACGTGGGCGAGTTCCGCGCGGTGACCGAGCTGGGGCGGCGCACAGCCAAGTACTGGAACAGCCAGAAGGAGCTCCTGGAGCAGAAGCGGGCCCAGGTGGACACGTAC

>GU332245_Rhabdomys_pumilio_MHC_class_II_antigen_(Rhpu-DRB)_gene_Rhpu-DRB*236_allele_exon_2_and_partial_cds

CAGCGCGTGCGGTTTCTGGAGAGATACTTCTACAACCGGGAGGAGTACGCGCGCTTCGACAGCGACGTGGGCGAGTACCGCGCGGTGACCGAGCTGGGGCGGCGCACAGCCAAGTACTGGAACAGCCAGAAGGATCTCCTGGAGCGGAAGCGGGCCCAGGTGGACACGTAC

>GU332246_Rhabdomys_pumilio_MHC_class_II_antigen_(Rhpu-DRB)_gene_Rhpu-DRB*237_allele_exon_2_and_partial_cds

CAGCGTGTGCGGTATCTGGAGAGACTATTCTACAACCGGGAGGAGTTTGCGCGCTTCGACAGTGACGTGGGGGAGTTCCGCGCGGTGTCCGAGCTGGGGCGGCCGGACGAGGAATACTGGAACAGCCAGAAGGATCTCCTGGAGCGGAAGCGTGCAGAGGTGGACACGTAC

>GU332247_Rhabdomys_pumilio_MHC_class_II_antigen_(Rhpu-DRB)_gene_Rhpu-DRB*238_allele_exon_2_and_partial_cds

CAGCGCGTGCGGTTTCTGGAGAGACACATCTACAACCGGGAGGAGTTCATGCGCTTCGACAGCGACGTGGGCGAGTACCGCGCGGTGACCGAGCTGGGGCGGCGCACAGCCAAGTACTGGAACAGCCAGAAGGAGTACCTGGAGCGGAAGCGGGCCCAGGTGGACGCGTAC

>GU332248_Rhabdomys_pumilio_MHC_class_II_antigen_(Rhpu-DRB)_gene_Rhpu-DRB*239_allele_exon_2_and_partial_cds

CAGCGCGTGCGGTTTCTGGACAGATACTTCTACAACCGGGAGGAGTACGCGCGCTTCGACAGCGACGTGGGCGAGTTCCGCGCGGTGACCGAGCTGGGGCGGCCGGACGCTGAGTACTGGAACAGCCAGAAGGAGCTCCTGGAGGATGGGCGGGCCCAGGTGGACGCGTAC

>GU332249_Rhabdomys_pumilio_MHC_class_II_antigen_(Rhpu-DRB)_gene_Rhpu-DRB*240_allele_exon_2_and_partial_cds

CAGCGCGTGCGGTTTCTGTTCAGAGATATCTACAACCAGGAGGAGCATGTGCGCTTCGACAGCGACGTGGGCGAGTACCGCGCGGTGACCGAGCTGGGGCGGCCGGACGCTGAGTACTGGAACAGCCAGAAGGAGATCCTGGAGGAGGCGCGGGCCCAGGTGGACACGTAC

>GU332250_Rhabdomys_pumilio_MHC_class_II_antigen_(Rhpu-DRB)_gene_Rhpu-DRB*241_allele_exon_2_and_partial_cds

CAGCGCGTGCGGTTTCTGTTCAGAGATATCTACAACCAGGAGGAGCATGTGCGCTTCGACAGCGACGTGGGCGAGTACCGCGCGGTGACCGAGCTGGGGCGGCCGGACGCTGAGTACTGGAACAGCCAGAAGGAGATCCTGGAGGATGCGCGGGCCGCGGTGGACACGTAC

>GU332251_Rhabdomys_pumilio_MHC_class_II_antigen_(Rhpu-DRB)_gene_Rhpu-DRB*242_allele_exon_2_and_partial_cds

CAGCGCGTGCGGTATCTGCAGAGATATTTCTACAACCGTCAGGAGTACGTGCGCTTCGACAGCGACGTGGGCGAGTTCCGCGCGGTGACCGAGCTGGGGCGGCCGGACGATGAATACTGGAACAGCCAGAAGGACTTCCTGGAGCATAAGCGGTCACAGGTGGACGCATAC

>GU332252_Rhabdomys_pumilio_MHC_class_II_antigen_(Rhpu-DRB)_gene_Rhpu-DRB*243_allele_exon_2_and_partial_cds

CAGCGCGTGCGGCTTCTGGAGAGATACATCTACAACCGGGAGGAGTACGTGCGCTTCGACAGCGACGTGGGCGAGTTCCGCGCGGTGACCGAGCTGGGGCGGCGCATAGATGAAGACTGGAACAGCCAGAAGGAGTTCCTGGAGCATAAGCGGGCCCAGGTGGACACGTAC

>GU332253_Rhabdomys_pumilio_MHC_class_II_antigen_(Rhpu-DRB)_gene_Rhpu-DRB*244_allele_exon_2_and_partial_cds

CAGCACGTGCGGCTTCTGGACAGATACTTCTACAACCGGGAGGAGTTCGTGCGCTTCGACAGCGACGTGGGCGAGTACCGCGCGGTGACCGAGCTGGGGCGGCGGGACGCCGAGCACTGGAACAGCCAGAAGGAGTTCCTGGAGCATGCGCGGGCCGCGGTGGACACGTAC

>GU332254_Rhabdomys_pumilio_MHC_class_II_antigen_(Rhpu-DRB)_gene_Rhpu-DRB*245_allele_exon_2_and_partial_cds

CAGCGCGTGCGGTTTCTGGACAGATACTTCTACAACCGGGAGGAGTTCGTGCGCTTCGACAGCGACGTGGGCGAGTACCGCGCGGTGACCGAGCTGGGGCGGCGGGACGCTGAGCACTGGAACAGCCAGAAGGAGCTCCTGGAGCAGAAGCGGGCCCAGGTGGACACGTAC

>GU332255_Rhabdomys_pumilio_MHC_class_II_antigen_(Rhpu-DRB)_gene_Rhpu-DRB*246_allele_exon_2_and_partial_cds

CAGCGCGTGCGGTTTCTGGACAGATACTTCTACAACCGGGAGGAGTTCATGCGCTTCGACAGCGACGTGGGCGAGTACCGCGCGGTGACCGAGCTGGGGCGGCGGGACGCTGAGCACTGGAACAGCCAGAAGGAGGTCCTGGAGCAGAAGCGGGCCCGGGTGGACACGTAC

>GU332256_Rhabdomys_pumilio_MHC_class_II_antigen_(Rhpu-DRB)_gene_Rhpu-DRB*247_allele_exon_2_and_partial_cds

CAGCTCGTGCGGTTTCTGGAGAGATACTTCTACAACCGGGAGGAGTACGCGCGCTTCGACAGCGACGTGGGCGAGTACCGCGCGGTGACCGAGCTGGGGCGGCGGGACGCTGAGTACTGGAACAGCCAGAAGGAGTACCTGGAGCAGAAGCGGGCCCAGGTGGACGCGTAC

>GU332257_Rhabdomys_pumilio_MHC_class_II_antigen_(Rhpu-DRB)_gene_Rhpu-DRB*248_allele_exon_2_and_partial_cds

CAGCTCGTGCGGTTTCTGTTCAGAGATATCTACAACCAGGAGGAGCATGTGCGCTTCGACAGCGACGTGGGCGAGTACCGCGCGGTGACCGAGCTGGGGCGGCCGGACGCTGAGTACTGGAACAGCCAGAAGGAGATCCTGGAGGATGCGCGGGCCGCGGTGGACACGTAC

>GU332258_Rhabdomys_pumilio_MHC_class_II_antigen_(Rhpu-DRB)_gene_Rhpu-DRB*249_allele_exon_2_and_partial_cds

CAGCGCGTGCGGTTTCTGGACAGATACTTCTACAACCGGGAGGAGTTCGTGCGCTTCGACAGCGACGTGGGCGAGTACCGCACTGTGACCGAGCTGGGGCGGGGCATAGCTGAGGACTGGAACAGCCAGAAGGAGTTCCTGGAGCATAAGCGGGCCGCGGTGGACACGTAC

>GU332259_Rhabdomys_pumilio_MHC_class_II_antigen_(Rhpu-DRB)_gene_Rhpu-DRB*250_allele_exon_2_and_partial_cds

CAGCACGTGAGGTTTCTGGAGAGACACATCTACAACCGGGAGGAGTTCATGCGCTTCGACAGCGACGTGGGCGAGTACCGCACTGTGACCGAGCTGGGGCGGCGCACAGCTGAGGACTGGAACAGCCAGAAGGAGGTCCTGGAGGATGCGCGGGCCGAGGTGGACACGTAC

>GU332260_Rhabdomys_pumilio_MHC_class_II_antigen_(Rhpu-DRB)_gene_Rhpu-DRB*251_allele_exon_2_and_partial_cds

CAGCGCGTGCGGTTTCTGGACAGATACTTCTACAACCGGGAGGAGTACGCGCGCTTCGACAGCGACGTGGGCGAGTACCGCGCGGTGACCGAGCTGGGGCGGCGGGACGCTGAGCACTGGAACAGCCAGAAGGAGATCCTGGAGCATGCGCGGGCCCGGGTGGACACGTAC

>GU332261_Rhabdomys_pumilio_MHC_class_II_antigen_(Rhpu-DRB)_gene_Rhpu-DRB*252_allele_exon_2_and_partial_cds

CAGCGCGTGCGGTTTCTGGACAGATACTTCTACAACCGGGAGGAGTACGCGCGCTTCGACAGCGACGTGGGCGAGTACCGCGCGGTGACCGAGCTGGGGCGGCCGGACGCTGAGTACTGGAACAGCCAGAAGGAGCTCCTGGAGGAGCAGCGGGCCCGGGTGGACACGTAC

>GU332262_Rhabdomys_pumilio_MHC_class_II_antigen_(Rhpu-DRB)_gene_Rhpu-DRB*253_allele_exon_2_and_partial_cds

CAGCACGTGCGGTTTCTGGACAGATACATCCACAACCGGGAGGAGTTCGTGCGCTTCGACAGCGACGTGGGCGAGTACCGCGCGGTGACCGAGCTGGGGCGGCGCATAGCTGAGGACTTGAACAGCCAGAAGGAGCTCCTGGAGCAGAAGCGGGCCGCGGTGGACACGTAC

>GU332263_Rhabdomys_pumilio_MHC_class_II_antigen_(Rhpu-DRB)_gene_Rhpu-DRB*254_allele_exon_2_and_partial_cds

CAGCACGTGCGGTTTCTGGACAGATACTTCTACAACCGGGAGGAGTACGCGCGCTTCGACAGCGACGTGGGCGAGTACCGCGCGGTGACCGAGCTGGGGCGGCCGGACGCTGAGTACTGGAACAGCCAGAAGGAGCTCCTGGAGGATCGGCGGGCCCAGGTGGACGCGTAC

>GU332264_Rhabdomys_pumilio_MHC_class_II_antigen_(Rhpu-DRB)_gene_Rhpu-DRB*255_allele_exon_2_and_partial_cds

CAGCGCGTGCGGTTTCTGGACAGATACATCCACAACCGGGAGGAGTTCGTGCGCTTCGACAGCGACGTGGGCGAGCACCGCGCGGTGACCGAGCTGGGGCGGCGCATAGCTGAGGACTGGAACAGCCAGAAGGAGCTCCTGGAGCAGAAGCGGGCCGAGCTGGACACGTAC

>GU332265_Rhabdomys_pumilio_MHC_class_II_antigen_(Rhpu-DRB)_gene_Rhpu-DRB*256_allele_exon_2_and_partial_cds

CAGCGCGTGCGGTTTCTGATCAGATACTTCTACAACCGGGAGGAGTACGTGCGCTTCGACAGCGACGTGGGCAGGTACCGCGCGGTGACCGAGCTGGGGCGGCGGGACGCTGATTACTGGAACAGCCAGAAGGAGATCCTGGAGGATGCGCGGGCCCAGGTGGACACGTAC

>GU332266_Rhabdomys_pumilio_MHC_class_II_antigen_(Rhpu-DRB)_gene_Rhpu-DRB*257_allele_exon_2_and_partial_cds

CAGCGCGTGCGGTTTCTGGAGAGATACTTCTACAACCGGGAGGAGTACGCGCGCTTCGACAGCGACGTGGGCGAGTACCGCGCGGTGACCGAGCTGGGGCGGCCGACAGCCAAGTACTGGAACAGCCAGAAGGAGTTCCTGGAGGATGCGCGGGCCCGGGTGGACACGTAC

>GU332267_Rhabdomys_pumilio_MHC_class_II_antigen_(Rhpu-DRB)_gene_Rhpu-DRB*258_allele_exon_2_and_partial_cds

CAGCGCGTGCGGTTTCTGGACAGATACTTCTACAACCGGGAGGAGTACGTGCGCTTCGACAGCGACGTGGGCGAGCACCGCGCGGTGACCGAGCTGGGGCGGCGCGCAGCCGAGTACTGGAACAGCCAGAAGGAGCTCCTGGAGGATGCGCGGGCCCAGGTGGACACGTAC

>GU332268_Rhabdomys_pumilio_MHC_class_II_antigen_(Rhpu-DRB)_gene_Rhpu-DRB*259_allele_exon_2_and_partial_cds

CAGCGCGTGCGGTTTCTGGACAGATACATCCACAACCGGGAGGAGTTCGTGCGCTTCGACAGCGACGTGGGCGAGTACCGCGCGGTGACCGAGCTGGGGCGGCGCATAGCTGAGGACTTGAACAGCCAGAAGGAGCTCCTGGAGCAGAAGCGGGCCGCGGTGGACACGTAC

>HE578727_Microtus_oeconomus_partial_Mioe-DRB_gene_for_MHC_class_II_antigen_allele_Mioe-DRB*01_exon_2

CAGCGCGTGCGGTTTCTGGACAGATACTTCTACAATCAGGAGGAGTATGCATGCTTTGACAGTGATGTGGGCAAGTACCACGCGGTGAACGAGCTGGGGCAGCCGGACGCTGAGTATTGGAACAGCCAGACGGAGCTCCTGGAGCAGGAGAGATCACTTGTGGACACTTAC

>HE578728_Microtus_oeconomus_partial_Mioe-DRB_gene_for_MHC_class_II_antigen_allele_Mioe-DRB*02_exon_2

CAGCGCGTGTGGCTTTTGGACAGATACTTCTACAACCAAGAGGAAAACGTGCGCTTCGACAGTGATGTGGGCGAATACCTTGCTGTGACCAAGATGGGGGAGCTGGAGGCCAAGAACTGGAACAGCCGGAAGGAGCTCCTGGAGGATGCGCGGGCCGGGGTGGACACATAC

>HE578729_Microtus_oeconomus_partial_Mioe-DRB_gene_for_MHC_class_II_antigen_allele_Mioe-DRB*03_exon_2

CAGCGCGTGCGGTTTCTGGACAGATACTTCTACAATCAGGAGGAGTATGCATGCTTTGACAGTGATGTGGGCAAGTACCACGCGGTGAACGAGCTGGGGCAGCCGGACGCTGAGTATTGGAACAGCCAGAAGGAGCTCCTGGAGCAGGAGAGATCCCTTGTGGACACTTAC

>HE578730_Microtus_oeconomus_partial_Mioe-DRB_gene_for_MHC_class_II_antigen_allele_Mioe-DRB*04_exon_2

CAGCGCGTGCGGTTTCTGGACAGATACTTCTACAATCAGGAGGAGTATGCATGCTTTGACAGTGATGTGGGCAAGTACCACGCGGTGAACGAGCTGGGGCAGCTGGACGCTGAGTATTGGAACAGCCAGACGGAGCTCCTGGAGCAGGAGAGATCGCTTGTGGACACTTAC

>HE578731_Microtus_oeconomus_partial_Mioe-DRB_gene_for_MHC_class_II_antigen_allele_Mioe-DRB*05_exon_2

CAGCGCGTGCGGTTTCTGGACAGATACTTCTACAATCAGGAGGAGTATGCATGCTTTGACAGTGATGTGGGCAAGTACCACGCGGTGAACGAGCTGGGGCAGCCGGACGCTGAGTATTGGAACAGCCAGACGGAGCTCCTGGAGCAGAAGAGATCGCTTGTGGACACTTAC

>HE578732_Microtus_oeconomus_partial_Mioe-DRB_gene_for_MHC_class_II_antigen_allele_Mioe-DRB*06_exon_2

CAGCGCGTGCGGTTTCTGGACAGATACTTCTACAATCAGGAGGAGTATGCATGCTTTGACAGTGATGTGGGCAAGTACCACGCGGTGAACCAGCTGGGGCAGCCGGACGCTGAGTATTGGAACAGCCAGACGGAGCTCCTGGAGCAGGAGAGATCGCTTGTGGACACTTAC

>HE578733_Microtus_oeconomus_partial_Mioe-DRB_gene_for_MHC_class_II_antigen_allele_Mioe-DRB*07_exon_2

CAGCGCGTGCGGTATCTGTACAGAGACATCTACAACCAGGAGGAGGTCGTGCGCTTCGACAGTGATGTGGGCAAGTATCACGCGGTGACCGAGCTGGGTCGGAGTGATGCCGAGGTCTGGAACAGCCAGAAGGAGGTCCTGCAGGACGCACGGGCTGCGGTGGACACGTAC

>HE578734_Microtus_oeconomus_partial_Mioe-DRB_gene_for_MHC_class_II_antigen_allele_Mioe-DRB*08_exon_2

CAGCGCGTACGGTATCTGTACAGAGACATTTACAACCAGGAGGAGGTCGTGCGCTTCGACAGTGATGTGGGCAAGTATCACGCGGTGACCGAGCTGGGTCGGAGTGATGCTGAGGTCTGGAACAGCCAGAAGGAGGTCCTGCAGGACGCACGGGCTGCGGTGGACACGTAC

>HE578735_Microtus_oeconomus_partial_Mioe-DRB_gene_for_MHC_class_II_antigen_allele_Mioe-DRB*09_exon_2

CAGCGCGTGCGGTATCTGTACAGAGACATCTACAACCAGGAGGAGGTCGTGCGCTTCGACAGTGATGTGGGCAAGTATCACGCGGTGACCGAGCTGGGTCGGAGTGATGCTGAGGTCTGGAACAGCCAGAAGGAGGTCCTGCAGGACGCACGGGCTGCGGTGGACACGTAC

>HE578736_Microtus_oeconomus_partial_Mioe-DRB_gene_for_MHC_class_II_antigen_allele_Mioe-DRB*10_exon_2

CAGCGCGTGCGGTTTCTGGACAGATACTTCTACAATCAGGAGGAGTATGCATGCTTTGACAGTGATGTGGGCAAGTACCACGCGGTGAACGAGCTGGGGCAGCCGGACGCTGAGTATTGGAACAGCCAGACGGAGCTCCTGGAGCAGGAGAGATCGCTTGTGGACACTTAC

>HE578737_Microtus_oeconomus_partial_Mioe-DRB_gene_for_MHC_class_II_antigen_allele_Mioe-DRB*11_exon_2

CAGCGCGTGCGGTTTCTGGACAGATACTTCTACAATCAGGAGGAGTATGCATGCTTTGACAGTGATGTGGGCAAGTACCACGCGGTGAACGAGCTGGGGCAGCCGGACGCTGAGTATTGGAACAGCCAGACGGAGCTCCTGGAGCAGGAGAGATCCCTTGTGGACACTTAC

>HE578738_Microtus_oeconomus_partial_Mioe-DRB_gene_for_MHC_class_II_antigen_allele_Mioe-DRB*12_exon_2

CAGAGCGTGCGGTATCTGTACAGAGACATCTACAACCAGGAGGAGGTCGTGCGCTTCGACAGTGATGTGGGCGAGTATCACGCGGTGACCGAGCTGGGTCGGAGTGATGCTGAGGTCTGGAACAGCCAGAAGGAGGTCCTGCAGGACGCACGGGCTGCGGTGGACACGTAC

>HE578739_Microtus_oeconomus_partial_Mioe-DRB_gene_for_MHC_class_II_antigen_allele_Mioe-DRB*13_exon_2

CAGCGCGTGCGGTATCTGTACAGAGACATCTACAACCAGGAGGAGGTCGTGCGCTTCGACAGTGATGTGGGCAAGTATCACGCGGTGACCGAGCTGGGTCGGAGTGATGCTGAGGTCTGGAACAGCCAGAAGGAGGTCCTGCAGGATGCACGGGCTGCGGTGGACACGTAC

>HE578740_Microtus_oeconomus_partial_Mioe-DRB_gene_for_MHC_class_II_antigen_allele_Mioe-DRB*14_exon_2

CAGCGCGTGCGGTTTCTGGACAGATACTTCTACAATCAGGAGGAGTATGCATGCTTTGACAGTGATGTGGGCAAGTACCACGCGGTGAACGAGCTGGGGCAGCCGGACGCTGAATATTGGAACAGCCAGACGGAGCTCCTGGAGCAGAAGAGATCGCTTGTGGACACTTAC

>HE578741_Microtus_oeconomus_partial_Mioe-DRB_gene_for_MHC_class_II_antigen_allele_Mioe-DRB*15_exon_2

CAGCGCGTGTGGCTTTTGGACAGATACTTCTACAACCAAGAGGAAAACGTGCGCTTCGACAGTGATGTGGGCGAATACCTTGCTGTGACCAAGATGGGGGAGCTGGAGGCCAAGAACTGGAACAGCCGGAAGGAGCTCCTGGAGGATGCGCGGGCCGGGGTGGACACGTAC

>HE578742_Microtus_oeconomus_partial_Mioe-DRB_gene_for_MHC_class_II_antigen_allele_Mioe-DRB*16_exon_2

CAGCGCGTGTGGCTTTTGGACAGATACTTCTACAACCAAGAGGAAAACGTGCGCTTTGACAGTGATGTGGGCGAATACCTTGCTGTGACCAAGATGGGGGAGCTGGAGGCCAAGAACTGGAACAGCCGGAAGGAGCTCCTGGAGGATGCGCGGGCCGGGGTGGACACATAC

>HE578743_Microtus_oeconomus_partial_Mioe-DRB_gene_for_MHC_class_II_antigen_allele_Mioe-DRB*17_exon_2

CAGCACGTGCGGTATCTGTACAGAGACATCTACAACCAGGAGGAGGTCGTGCGCTTCGACAGTGATGTGGGCAAGTATCACGCGGTGACCGAGCTGGGTCGGAGTGATGCTGAGGTCTGGAACAGCCAGAAGGAGGTCCTGCAGGACGCACGGGCTGCGGTGGACACGTAC

>HE578744_Microtus_oeconomus_partial_Mioe-DRB_gene_for_MHC_class_II_antigen_allele_Mioe-DRB*18_exon_2

CAGCGCGTGCGGTATCTGTACAGAGACATCTACAACCAGGAGGAGGTCGTGCGCTTCGACAGTGATGTGGGCGAGTATCACGCGGTGACCGAGCTGGGTCGGAGTGATGCTGAGGTCTGGAACAGCCAGAAGGAGGTCCTGCAGGACGCACGGGCTGCGGTGGACACGTAC

>HM107849_Myodes_glareolus_MHC_class_II_antigen_(Mygl-DRB)_gene_Mygl-DRB*93_allele_exon_2_and_partial_cds

CAGCGCGTGCGGTATCTGTACAGAGACATCTACAACCAGGAGGAGGTCGTGCGCTTCGACAGTGATGTGGGCGAGTATCACGCGGTGACCGAGCTGGGTCGCAGTGATGCTGAGGTCTGGAACAGCCAGAAGGAGGTCCTGGAGGACGCACGGGCCGCGGTGGACACGTAC

>HM107852_Myodes_glareolus_MHC_class_II_antigen_(Mygl-DRB)_gene_Mygl-DRB*96_allele_exon_2_and_partial_cds

CAGCGCGTGCGGTATCTGTTCAGAGACATCTACAACCAGGAGGAGCATGTGCGCTTCGACAGCGACGTGGGCGAGTTCCGCGCGGTGACCGAGCTGGGGCGGCCAGACGCCGAGTACTGGAACAGCCAGAAGGACTACCTGGAGCGGAAGCGGGCCGAGACGGACACGGTG

>HM107853_Myodes_glareolus_MHC_class_II_antigen_(Mygl-DRB)_gene_Mygl-DRB*97_allele_exon_2_and_partial_cds

CAGCACGTGCGGCTTCTGGACAGGTTCATCTACAATCGGGAAGAGTACGTGCGCTTTGACAGCGACTTGGGCGAGTTCCGTGCGGTGACCGAGCTGGGGCGGCCCTCAGCCAAGTACTGGAACAGCCAGAAGGAGATCCTGGACAACAGGCAGGCCGCGCTGGACACGTAC

>HM107854_Myodes_glareolus_MHC_class_II_antigen_(Mygl-DRB)_gene_Mygl-DRB*98_allele_exon_2_and_partial_cds

CAGCGCGTGCGGTATCTGTACAGAGACATCTACAACCAGGAGGAGTTCGTGCGCTTCGACAGTGATGTGGGCGAGTACCGCGAGGTGACTGAGCTGGGGCAGCGGGACGCCGAGTACCGGAACAGCCAGAAGGAGCTCCTGGACCACAGGCGGGCCGCGGTGGACACATAC

>HM107855_Myodes_glareolus_MHC_class_II_antigen_(Mygl-DRB)_gene_Mygl-DRB*99_allele_exon_2_and_partial_cds

CAGCGCGTGCGGTATCTGTACAGAGACATCTACAATCAGGAGGAGGTCGTGCGCTTCGACAGTGATGTGGGCGAGTATCACGCGGTGACCGAGCTGGGTCGGAGTGATGCTGAGGTCTGGAACAGCCAGAAGGAGGTCCTGGAGGACGCACGGGCCGCGGTGGACACGTAC

>HM107856_Myodes_glareolus_MHC_class_II_antigen_(Mygl-DRB)_gene_Mygl-DRB*100_allele_exon_2_and_partial_cds

CAGCACGTGCGGCTTCTGGACAGATTCTTCTACAACCGGGAGGAGTACGTGCGCTTTGACAGCGACTTGGGCGAGTTCCGTGCGGTGACCGAGCTGGGGCGGCCCTCAGCCAAGTACTGGAACAGCCAGAAGGAGATCCTGGACTACAGGCGGGCCGCGCTGGACACGTTC

>HM107857_Myodes_glareolus_MHC_class_II_antigen_(Mygl-DRB)_gene_Mygl-DRB*101_allele_exon_2_and_partial_cds

CAGCGCGTGCGGTATCTGTACAGAGACATCTACAATCAGGAGGAGGTCGTGCGCTTCAACAGTGATGTGGGCGAGTATCACGCGGTGACCGAGCTGGGTCGCAGTGATGCTGAGGTCTGGAACAGCCAGAAGGAGGTCCTGGAGGACGCACGGGCCGCGGTGGACACGTAC

>HM107858_Myodes_glareolus_MHC_class_II_antigen_(Mygl-DRB)_gene_Mygl-DRB*102_allele_exon_2_and_partial_cds

CAGCGCCTGCGGTTTCTGGAAAGACACATCTTCAACCGGGAGGAGTTCGTGCGCTTTGACAGTGACGTGGGCGAATACCGTGCGGTGAACGAGCTGGGGCGACCGGTCGCTGAGGTCTGGAACAGCCAGAAGGAGCGCCTGAAGTACGCACGGGCCGCGGTGGACACTTAC

>HM107859_Myodes_glareolus_MHC_class_II_antigen_(Mygl-DRB)_gene_Mygl-DRB*103_allele_exon_2_and_partial_cds

CAGCGCGTGCGGTTTCTGGACAGATACTTCTACAACCGGGAGGAGTACGTGCGCTTCGACAGTGACGTGGGCGAGTACCGCGCGGTGACCGAGCTGGGGCGGCCCTCAGCCGAGTACTGGAACAGCCAGAAGGAGCTCCTGGAGCAGAAGCGGGCCAATGTGGACACGTAC

>HM107860_Myodes_glareolus_MHC_class_II_antigen_(Mygl-DRB)_gene_Mygl-DRB*104_allele_exon_2_and_partial_cds

CAGCACGTGCGGCTTCTGGAAAGATACTTCTACAACCGGAAGGAGTACGTGCGCTTTGACAGTGATGTGGGCGAGTACCGCGCAGTGAACGAGCTGGGGCGGCCCTCAGCCGAGTACTGGAACAGCCAGAAGGAGTTCCTGGACAACAGGCGGGCCACGGCGGACACGTAC

>HM107863_Myodes_glareolus_MHC_class_II_antigen_(Mygl-DRB)_gene_Mygl-DRB*107_allele_exon_2_and_partial_cds

CAGCGCGTGCGGTATCTGTTCAGAGACATCTACAACCAGGAGGAGCATGTGCGCTTCGACAGCGACGTGGGCGAGTTCCGCGCGGTGACCGAGCTGGGGCGGCCAGACGCCGAGTACTGGAACAGCCGGAAGGACTACCTGGAGCGGAAGCGGGCCGAGACGGACACGGTG

>HM107864_Myodes_glareolus_MHC_class_II_antigen_(Mygl-DRB)_gene_Mygl-DRB*108_allele_exon_2_and_partial_cds

CAGCGCGTGCGGTATCTGTACAGAGACATCTACAACCAAGAAGAGGTCGTGCGCTTCGACAGTGATGTGGGCAAGTATCACGCGGTGACCGAGTTGGGTCGGAGTGATGCTGAGGTCTGGAACAGCCAGAAGGAGGTCCTGGAGGATGCACGGGCCGCGGTGGACACTTAC

>HM107865_Myodes_glareolus_MHC_class_II_antigen_(Mygl-DRB)_gene_Mygl-DRB*109_allele_exon_2_and_partial_cds

CAGCGCGTGCGGTTTCTGGACAGATACTTCTACAACCAGGAGGAGTACGCACGCTTCGACAGCGACATTGGCGAGCACCGTGCAGTGAACGAGCTGGGGCGGCCCTCAGCCGAGTACTGGAACAGCCAGAAGGAGCTCCTGGAGCAGAAGCGGGCCTATGTGGACACGTTC

>HM107866_Myodes_glareolus_MHC_class_II_antigen_(Mygl-DRB)_gene_Mygl-DRB*110_allele_exon_2_and_partial_cds

CAGCACGTGCGGCTTCAGGACAGATTCTTCTACAACCGGGAGGAGTACGTGCGCTTTGACAGCGACTTGGGCGAGTTCCGTGCGGTGACCGAGCTGGGGCGGCCCTCAGCCAAGTACTGGAACAGCCAGAAGGAGATCCTGGACTACAGGCGGGCCGCGCTGGACACGTTC

>HM107867_Myodes_glareolus_MHC_class_II_antigen_(Mygl-DRB)_gene_Mygl-DRB*111_allele_exon_2_and_partial_cds

CAGCACGTGCGGTATCTGGACAGATACTTCTACAACCGGAAGGAGTACGTGCGCTTTGACAGCGACGTGAGCGAGTACCGTGCAGTGACCGAGCTGGGGCGACCGGACGCCAAGTACTGGAACAGCCAGAAGGAGCTCCTGGAGCAGAAGCGGGCCAAGGTGGACACATAC

>HM107868_Myodes_glareolus_MHC_class_II_antigen_(Mygl-DRB)_gene_Mygl-DRB*112_allele_exon_2_and_partial_cds

CAGCGCGTGCGGTATCTGTACAGAGACATCTACAACCAGGAGGAGGTCGTGCGCTTCGACAGTGATGTGGGCGAGTATCACGCGGTGACCGAGCTGGATCGCAGTGATGCTGAGGTCTGGAACAGCCAGAAGGAGGTTCTGGAGGACGCACGGGCCGCGGTGGACACGTAC

>HM107869_Myodes_glareolus_MHC_class_II_antigen_(Mygl-DRB)_gene_Mygl-DRB*113_allele_exon_2_and_partial_cds

CAGCGCGTGCGGTATCTGTACAGAGACATCTACAATCAGGAGGAGGTCGTGCGCTTCGACAGTGATGTGGGCGAGTATCACGCGGTGACCGAGCTGGGTCGCAGTGATGCTGAGGTCTGGAACAGCCAGAAGGAGGTCCTGGAGGACGCACGGGCCGCGGTGGACATGTAC

>HM107870_Myodes_glareolus_MHC_class_II_antigen_(Mygl-DRB)_gene_Mygl-DRB*114_allele_exon_2_and_partial_cds

CAGCACATGAAGCTTGTGGCCAGAATATTCTACAACCGGGACGAGATCGTGCGCTACGACAGCGACGTGGGCGAGTTCCGCGCGGTGACCGAGCTGGGGCGGCCGGACGCCGAGTACTGGAACAGCCAGAAGGACTACCTGGAGCAGCTGCGGGCCGAGATAGACACGGTG

>HM347503_Myodes_glareolus_MHC_class_II_antigen_(Mygl-DRB)_gene_Mygl-DRB*115_allele_exon_2_and_partial_cds

CAGCACATGCGGCACGTGGACAGAATATTCTACAACCGGGAGGAGATCGTGCGCTACGACAGCGACGTGGGCGAGTTCCGCGCGGTGACCGAGCTGGGGCGGCCGGACGCCGAGTACTGGAACAGCCAGAAGGACTACCTGGAGCAGCTGCGGGCCGAGATAGACACGGTG

>HM347504_Myodes_glareolus_MHC_class_II_antigen_(Mygl-DRB)_gene_Mygl-DRB*116_allele_exon_2_and_partial_cds

CAGCACATAAGGCTTGTGTCGAGCTACGTCTACAACCAGGAGGAGGTCGTGCGCTTCGACAGTGACGTGGGCGAGTTCCGCGCAGTGACCGAGCTGGGGCGGACGGACGCCGAGTACTTCAACAGCCAGAAGGACTATCTGGAGCAGAAGCGGGCCGCGGTGGACACGGTG

>HM347506_Myodes_glareolus_MHC_class_II_antigen_(Mygl-DRB)_gene_Mygl-DRB*118_allele_exon_2_and_partial_cds

CAGCGCGTGAGGTATCTGGTCAGAGTCATCTACAACCGAGAGGAGTACGCGCGCTTCGACAACGACGTGGGCGAGTTCCGCGCGGTGACCGAGCTGGGGCGGCGGGACGCCGAGTACTGGAACAAACAGAAGGAGTACATAGAGCAGAAGCGGGCCGAGGTGGACACGGTC

>HM347507_Myodes_glareolus_MHC_class_II_antigen_(Mygl-DRB)_gene_Mygl-DRB*119_allele_exon_2_and_partial_cds

CAGCGCATAAGGCTTGTGGCGAGCTACGCCTACAACCAGGAGGAGGTCGTGCGCTTCGACAGTGACGTGGGCGAGTTCCGCGCGGTGGCCGAGCTGGGGCGGTCGTGGGCCGAGGACTTCAACAGTCGGAAGGACTACCTGGAGCAGATGCGGGACGCGGTGGACACGGTG

>HM461912_Spermophilus_suslicus_MHC_class_II_antigen_(Spsu-DRB)_gene_Spsu-DRB*17_allele_exon_2_and_partial_cds

GAGGGGGTACGGTTCCTGAACAGATACTTCCACAACCGGGAGGAGTTCGTGCGCTTCGACAGCGACGTGAGGGGGTTTCTCGCGGTGAGCGAGCTGGGGCGACCGGACGCCGAGGACTGGAACAGCCAGAAGGACTTCCTGGAGGGGAGGCGGGCCGAGGTGGACACTGTG

>HM461913_Spermophilus_suslicus_MHC_class_II_antigen_(Spsu-DRB)_gene_Spsu-DRB*18_allele_exon_2_and_partial_cds

GAGGGGGTACGGTTCCTGGACAGATACTTCCACAACCGGGAGGAGTTCGTGCGCTTCGACAGCGACGCGAGGGGGTTTCTCGCGGTGAGCGAGGTGGGGCGACCGGACGCCAAGGACTGGAACAGCCAGAAGGACGCCCTGGAGGGGAGGCGGGCGGAGGTGGACACCTAC

>JF681146_Spermophilus_citellus_MHC_II_class_antigen_(Spci-DRB)_gene_Spci-DRB*01_allele_exon_2_and_partial_cds

GAGCGGGTGCGGCTCCTGGACAGATACTTCTACAACCGGGAGGAATACGTGCGCTTCGACAGTGACGTGGGGGAGTTCCGCGCGGTGAGCGAGCTGGGGCGGCCGGACGCCGAGTACTGGAACAGCCAGAAGGACATCCTGGAGCGGAAGCGGGCCGAGGTGGACACTGCG

>JF681147_Spermophilus_citellus_MHC_II_class_antigen_(Spci-DRB)_gene_Spci-DRB*02_allele_exon_2_and_partial_cds

GAGCGGGTGCGGCTCCTGGACAGATACTTCCACAACCGGGAGGAGTACGCGCGCTTCGACAGCGACGTGGGGGAGTTCCGCGCGGTGAGCGAGCTGGGGCGGCCGGCAGCCGAGTACTGGAACAGCCAGAAGGACATCCTGGAGGGGAGGCGGGCCGCGGTGGAAACTACT

>JF681148_Spermophilus_citellus_MHC_II_class_antigen_(Spci-DRB)_gene_Spci-DRB*03_allele_exon_2_and_partial_cds

GAGCGGGTGCAGTTACTGGTCAGATTCTTCTACAACCGGGAGGAGTTCGCGCGCTTCGACAGCGACGTGGGGGAGTACCGCGCGGTGACCGAGGTGGGGCGGCAGGACGCCAAGTACTGGAACAGCCAGAAGGACGCCCTGGAGCGGAGGCGGGCCGAGGTGGAACTGTAT

>JF681149_Spermophilus_citellus_MHC_II_class_antigen_(Spci-DRB)_gene_Spci-DRB*04_allele_exon_2_and_partial_cds

GAGCGGGTGCGGTTCCTGGAGAGATACTTCCACAACCGGGAGGAGGTCGTGCGCTTCGACAGCGACGTGGGGGAGTACCGCGCGGTGACAGAGCTGGGGCGGCTGGACGCCGAGAGCTGGAACAGCCAGAAGGACTTCCTGGAGCGGAAGCGGGCCGAGGTGGAACGTTCT

>JF799108_Ctenomys_talarum_MHC_class_II_antigen_(Ctta-DRB01)_gene_exon_2_and_partial_cds

GAGCATGTTCAGATGGTGGTCAGACACATCTACAAGCAGGAGGAGTTCTTACGCTACGACAGTGACCTGGGCAAGTACCTGGCAGTGACTGGGTTAGGGCGGCAGGAGGCAGAAGACTGGAACACCAGGAAAGACCTCCTGGAGCAGAGGCGTTCCCAGCTGGACACCTTG

>JF799109_Ctenomys_talarum_MHC_class_II_antigen_(Ctta-DRB02)_gene_exon_2_and_partial_cds

GAGCATGTTCAGATGGTGGTCAGACACATCTACAAGCAGGAGGAGTTCTTACGCTACGACAGTGACCTGGGCAAGTACCTGGCAGTGACTGGGTTAGGGCGGCAGGAGGCAGAAGACTGGAACACCAGGAAAGACCTCCTGGAGCAGAGGCATTCCCAGCTGGACACCTTG

>JF799110_Ctenomys_talarum_MHC_class_II_antigen_(Ctta-DRB03)_gene_exon_2_and_partial_cds

GAGCATATTCAGATGGTGGTCAGACACATCTACAAGCGGGAGGAGTTCCTGCGCTATGACAATGACCTGGGCAAGTACCTGGCAGTGACTGGGCTAGGGCGGCAGGAGGCGGAAGACTGGAACACCAGGAAAGACCTCCTGGAGCAGAGGCGTTCCCAGCTGGACACCTTG

>JF799111_Ctenomys_talarum_MHC_class_II_antigen_(Ctta-DRB04)_gene_exon_2_and_partial_cds

GAGCATGTTCAGATGGTGGTCAGACACATCTACAAGCGGGAGGAGTTCCTGCGCTATGACAATGACCTGGGCAAGTACCTGGCAGTGACTGGGTTAGGGCGGCAGGAGGCAGAAGACTGGAACACCAGGAAAGACCTCCTGGAGCAGAGGCGTTCCCAGCTGGACACCTTG

>JF799112_Ctenomys_talarum_MHC_class_II_antigen_(Ctta-DRB05)_gene_exon_2_and_partial_cds

GAGCATGTTCAGATGGTGGTCAGACACATCTACAAGCAGGAGGAGTTCTTACGCTACGACAGTGACCTGGGCAAGTACCTGGCAGTGACTGGGTTAGGGCGGCAGGAGGCAGAAGACTGGAACACGAGGAAAGACCTCCTGGAGCAGAGGCGTTCCCAGCTGGACACCTTG

>JF799113_Ctenomys_talarum_MHC_class_II_antigen_(Ctta-DRB06)_gene_exon_2_and_partial_cds

GAGCATGTTCAGATGGTGGTCAGACACATCTACAAGCAGGAGGAGTTCTTACGCTACGACAGCGACCTGGGCAAGTACCTGGCAGTGACTGGGTTAGGGCGGCAGGAGGCAGAGGACTGGAACACCAGGAAAGACCTCCTGGAGCAGAGGCGTTCCCAGCTGGACACCTTG

>JF799114_Ctenomys_talarum_MHC_class_II_antigen_(Ctta-DRB07)_gene_exon_2_and_partial_cds

GAGCATGTTCAGATGGTGGTCAGACACATCTACAAGCAGGAGGAGTTCTTACGCTACGACAGTGACCTGGGCAAGTACCTGGCAGTGACTGGGTTAGGGCGGCAGGAGGCAGAAGACTGGAACACGAGGAAAGACCTCCTGGAGCAGAGGCATTCCCAGCTGGACACCTTG

>JF799115_Ctenomys_talarum_MHC_class_II_antigen_(Ctta-DRB08)_gene_exon_2_and_partial_cds

GAGCATGTTCAGATGGTGGTCAGACACATCTACAAGCAGGAGGAGTTCTTACGCTACGACAGTGACCTGGGCAAGTACCTGGCAGTGACTGGGCTAGGGCGGCAGGAGGCGGAAGACTGGAACACCAGGAAAGACCTCCTGGAGCAGAGGCGTTCCCAGCTGGACACCTTG

>JF799116_Ctenomys_talarum_MHC_class_II_antigen_(Ctta-DRB09)_gene_exon_2_and_partial_cds

GAGCATGTTCAGATGGTGGTCAGACACATCTACAAGCGGGAGGAGTTCTTGCGCTATGACAATGACCTGGGCAAGTACCTGGCAGTGACTGGGTTAGGGCGGCAGGAGGCAGAAGACTGGAACACCAGGAAAGACCTCCTGGAGCAGAGGCGTTCCCAGCTGGACACCTTG

>JQ317130_Ctenomys_talarum_MHC_class_II_antigen_(Ctta-DRB11)_gene_exon_2_and_partial_cds

GAGCATGTTCAGATGGTGGTCAGACACATCTACAAGCGGGAGGAGTTCCTGCGCTATGACAATGACCTGGGCAAGTACCTGGCAGTGACTGGGCTAGGGCGGCAGGAGGCAGAAGACTGGAACACCAGGAAAGACCTCCTGGAGCAGAGGCGTTCCCAGCTGGACACCTTG
