# Supplementary material for: Duplication and population dynamics shape historic patterns of selection and genetic variation at the major histocompatibility complex in rodents
Source: Ecol Evol. 2013 Apr 22;3(6):1552–68. doi: 10.1002/ece3.567 (PMC3686191; doi:10.1002/ece3.567)
Supplement: Supplementary file 2 [file ece30003-1552-SD2.docx]

**SUPPLEMENTARY MATERIALS**

**S1. Genetic methods and statistical analyses**

**Tagged primer design, amplification, and 454 sequencing**

Genomic DNA was extracted using the PureGene DNA isolation kit (Gentra Systems), following the manufacturer’s protocol. We used oligonucleotide forward primer JS1 (5’-AGTGTCATTTCTACAACGGGACG-3’) and reverse primer JS2 (5’-GATCCCGTAGTTGTGTCTGCA-3’) described by [Schad *et al.* (2004](#_ENREF_22)). These primers were previously successful in amplifying the DRB locus for numerous rodent species ([Froeschke & Sommer 2005](#_ENREF_5); [Harf & Sommer 2005](#_ENREF_7); [Meyer-Lucht & Sommer 2005](#_ENREF_16); [Oliver & Piertney 2006](#_ENREF_17)); specifically, they amplified a 171 bp fragment of the second exon of the DRB gene that includes part of the functional antigen-binding site (ABS). This primer system has also been successful in amplifying sequences from multiple loci in rodents when the DRB locus has been duplicated ([Galan *et al.* 2010](#_ENREF_6)).

All PCRs were performed in a reaction volume of 20 μl, each containing 40–100 ng of DNA, 0.5 mM of each primer (Invitrogen), 4ul of 5X reaction buffer, 2ul of 2.5mM MgCl2, 2ul of a mix of 10 mM deoxyribonucleotide triphosphates, and 0.2ul of (5u/ul) GoTaq^®^Flexi DNA Polymerase (Promega M8295). Thermocycling was carried out on an Eppendorf Mastercycler^®^ ep with an initial denaturation step at 96°C for 120s followed by 30 cycles of denaturation at 94°C for 30s, annealing at 57°C for 30s , elongation at 72°C for 60s, and a final extension at 72°C for 10 min. PCR products were cloned using the TOPO TA Cloning Kit (Invitrogen K4500-01) and transformed plasmids into One Shot^®^Top10 Chemically Competent *E. coli*. Transformed cells were grown on X-gal coated LB plates with 50ug/ml ampicillin overnight, and recombinant clones were detected by white/blue screening. Six to ten colonies containing inserts were randomly selected per individual (N=20) and sequenced using M13 primers. Sequencing reactions were performed with the Big Dye 3.1 sequencing kit (ABI) and their products were separated on an ABI 3730xl 96-capillary DNA Analyzer. Sequences were checked and aligned using Geneious Pro v5.5 ([Kearse *et al.* 2012](#_ENREF_9)). Preliminary cloning and sequencing revealed the possibility of a duplicated locus in *M. montanus*, but we were not able to perform mRNA analyses. Other studies have shown that duplicated DRB loci can be nonfunctional ([Axtner & Sommer 2007](#_ENREF_1); [Oppelt *et al.* 2010](#_ENREF_18)) but their RNA products may still be expressed ([Zagalska-Neubauer *et al.* 2010](#_ENREF_23)). Therefore, the functional significance of alleles from duplicated MHC loci will not always be directly revealed by transcription analysis.

To assign 454 sequencing reads to specific individuals, 9-bp tags were used to create 18 forward and 18 reverse 5’ tagged primers that resulted in 324 unique JS1-tagged and JS2-tagged primer pairs. These 9-bp sequences (created at http://faircloth-lab.github.com/edittag/) were developed to have an edit distance of five, whereby five mutations are required for one tag to transform into another sequence ([Faircloth & Glenn 2011](#_ENREF_4)). We visually assessed band intensities on agarose gels to verify that approximately equimolar quantities of PCR products were obtained with the 36 different tags in all combinations. Individual PCR products were concentrated and normalized using the SequalPrep™ Normalization Plate Kit (Invitrogen A10510-01). 10ul of sample from each individual (N=284) was pooled and sequenced as a single 454 Titanium run at the Georgia Genomics Facility, Athens, Georgia. To further visualize and analyze sequencing data and assign reads to individuals, we used the SESAME software ([Meglécz *et al.* 2011](#_ENREF_15)).

**MHC genotyping and allele validation**

In addition to sufficient coverage, reliable genotyping of individuals requires that true alleles be distinguished from artifacts that can arise from PCR amplification errors and 454 sequencing errors. Point mutations due to DNA polymerase errors in PCR and sequencing reactions, and indels (insertion or deletion less than 3bp) are common errors ([Margulies *et al.* 2005](#_ENREF_13); [Huse *et al.* 2007](#_ENREF_8)) and can be identified if they cause frame shift mutations or if they produce shortened or elongated alleles of relatively low frequency within amplicon reads. Finally, chimeras, produced by in-vitro recombination of true alleles during PCR, are difficult to address because they may look similar to true in-vivo recombinants and may occur in a relatively high number of reads ([Longeri *et al.* 2002](#_ENREF_12); [Galan *et al.* 2010](#_ENREF_6)). Since artificial chimeras must always co-occur with parental alleles, they should be identified by examining all sequence variants present in an individual ([Zagalska-Neubauer *et al.* 2010](#_ENREF_23)).

We followed procedures for genotyping non-model species from previously published papers to filter out artifacts ([Galan *et al.* 2010](#_ENREF_6); [Kloch *et al.* 2010](#_ENREF_11); [Zagalska-Neubauer *et al.* 2010](#_ENREF_23)). We based our filtering procedure on a series of thresholds under the assumption that true alleles will be more common than artifacts across all individuals and within individuals ([Babik *et al.* 2009](#_ENREF_3)). First, we called sequence variants putative alleles if they were present in at least 3 reads and made up at least 3% of reads of all variants within an individual ([Babik *et al.* 2009](#_ENREF_3)). Second, we looked across individuals and retained putative alleles that were present in at least two individuals, representing the gold-standard two-PCR criterion of MHC studies (i.e., where an allele must be obtained from two independent PCRs to guard against PCR artifacts ([Babik 2010](#_ENREF_2)). Only 1 putative allele that qualified based on length, read number per individual, and > 3 base pair differences from higher frequency alleles was found in a single individual and was retained in the final dataset. Third, we checked all putative alleles for indels and stop codons (putative pseudogenes) and removed 14 variants that had insertions or deletions less than 3bp in length, which would result in a frameshift mutation. Fourth, to check for low frequency true alleles, we examined each individual with sequence variants between 2-3% frequency and retained variants that differed by at least three substitutions from the most similar higher frequency allele ([Kloch *et al.* 2010](#_ENREF_11)). All those that differed by 3-bp or less were at low frequency (< 3%) and low read number (< 3 reads), and always co-occurred with the same more frequent alleles. These represented 59 putative alleles out of 82 and were discarded from the final dataset. Two alleles were greater than 3-bp differences from the most frequent alleles, yet were only found in a single individual each, had less than 3 reads, and were at 1% frequency within their respective amplicons. These were removed as well. Fifth, we checked for chimeras by visually inspecting alleles per individual and determined whether putative alleles always co-occurred with putative parental alleles and using the program Chimaera ([Posada & Crandall 2001](#_ENREF_19)) in the RDP3 program ([Martin *et al.* 2010](#_ENREF_14)). This represented one putative allele, which was removed from the full set. Finally, we tested the coverage threshold of 46 reads per individual using linear regression between sequence number and putative alleles, and found a slight increase in allele numbers associated with increasing read counts (*P* = 0.02; adj.R^2^ = 0.032), indicating more coverage would identify more alleles. This relationship ceased to be significant at a threshold of 54 reads per individual (*P* = 0.06; adj.R^2^ = 0.023).

**Population dynamics characterization**

We delineated species to these categories based on published studies of their population ecology derived from systematic literature searches on Web of Science using the search terms “species binomial name and pseudonyms” and “pop*” or “cyclic” or “bottleneck”. Species assigned to multiannual cycles were those that fluctuated in abundance by a factor of more than 2 at a minimum of every 2 years (Table S1). Species assigned to the bottlenecked category were those that underwent a reduction in population size of greater than 80% or that could be observed in the molecular history of the species (Table S1). If a species was found to experience multiannual cycles or bottlenecks in one population, we classified the entire species by that category. Species for which studies of population size or abundance over time reported no evidence of multiannual cycles or bottlenecks were assigned to the ‘stable’ category. A minimum of two studies per rodent species detailing population dynamics was required to classify species.

**Microtus montanus DRB phylogeny**

The Kimura two-parameter distance ([Kimura 1980](#_ENREF_10)) was applied to construct a neighbor-joining phylogenetic tree of the 21 alleles in MEGA 5.05 based on the shared sequence sections of all alleles ([Saitou & Nei 1987](#_ENREF_21)). We conducted a bootstrap analysis with 5,000 replicates to assess reliability of branching within the tree. Another phylogenetic tree of DRB alleles was constructed within a Bayesian framework with MrBayes 3.2 ([Ronquist *et al.* 2012](#_ENREF_20)). The likelihood settings of the model corresponded to the parameter values estimated from the data, as there was no one model with posterior probability > 0.05 after sampling across the entire general time reversible (GTR) model space. We set priors to default values. Four Metropolis-coupled Markov chains (three of them ‘heated’, temperature = 0.10) were run for 10^6^ generations and sampled every 100 generations. The first 25% of trees were discarded as burn-in, resulting in a total of 15002 sampled trees. To calculate the posterior probability of each bipartition, the majority-rule consensus tree was computed from these 15002 sampled trees.

**Table S1.** The comparative dataset of 16 rodent species used in the analysis.

**Table S2**. Results from codon-based Z tests for i) departures from neutrality (dN/dS ≠ 1) at the antigen binding sites (ABS) based on Brown *et al.* (1993) and Bondinas *et al.* (2007); ii) negative selection (dS-dN > 1) at non-ABS across 16 rodent species and *Tupaia*. The species are categorized by presence of duplicated DRB loci (0,1) and presence of cyclic population dynamics (0,1). Significant P-values are in bold.

|  |  | **dN/dS^a^** | | **dN/dS^b^** | |  | **dS-dN^a^** | | **dS-dN^b^** | | |  |  |
| --- | --- | --- | --- | --- | --- | --- | --- | --- | --- | --- | --- | --- | --- |
| **Species** | **No. Sequences** | **ABS** | **P-value** | **ABS** | **P-value** |  | **nonABS** | **P-value** | **nonABS** | **P-value** | | **Duplicated** | **Cyclic** |
| *Apodemus flavicollis* | 26 | 3.770 | **0.001** | 5.923 | **0.000** |  | -2.331 | 1.000 | -1.701 | | 1.000 | 0 | 0 |
| *Apodemus sylvaticus* | 38 | 3.281 | **0.000** | 4.455 | **0.000** |  | -3.040 | 1.000 | -2.481 | | 1.000 | 0 | 0 |
| *Arvicola terrestris* | 23 | 4.481 | **0.000** | 6.893 | **0.000** |  | -1.697 | 1.000 | -1.284 | | 1.000 | 1 | 1 |
| *Castor fiber* | 10 | 5.252 | **0.014** | 6.395 | **0.000** |  | -0.917 | 1.000 | -0.106 | | 1.000 | 1 | 0 |
| *Ctenomys talarum* | 26 | 0.000 | 0.459 | 0.000 | 0.342 |  | 1.332 | 0.093 | 1.543 | | 0.063 | 0 | 0 |
| *Dipodomys spectabilis* | 5 | 3.086 | 0.111 | 3.861 | 0.058 |  | -0.255 | 1.000 | 0.092 | | 0.463 | 1 | 0 |
| *Gerbillurus paeba* | 33 | 3.526 | **0.001** | 3.213 | **0.000** |  | -0.396 | 1.000 | -0.299 | | 1.000 | 1 | 0 |
| *Hypogeomys antimena* | 4 | 2.883 | 0.275 | 4.314 | **0.027** |  | -0.053 | 1.000 | 0.369 | | 0.356 | 0 | 0 |
| *Microtus montanus* | 21 | 0.761 | 0.585 | 0.677 | 0.399 |  | 1.895 | **0.030** | 1.929 | | **0.028** | 1 | 1 |
| *Microtus oeconomus* | 18 | 0.730 | 0.468 | 0.606 | 0.326 |  | 2.027 | **0.022** | 2.000 | | **0.024** | 1 | 1 |
| *Myodes glareolus* | 106 | 1.308 | 0.396 | 1.222 | 0.463 |  | 2.434 | **0.008** | 2.328 | | **0.011** | 1 | 1 |
| *Peromyscus maniculatus* | 28 | 2.528 | **0.004** | 5.101 | **0.000** |  | -0.691 | 1.000 | 0.193 | | 0.423 | 0 | 0 |
| *Rattus rattus* | 5 | 2.665 | **0.023** | 2.384 | **0.016** |  | 0.462 | 0.322 | 0.309 | | 0.379 | 0 | 0 |
| *Rhabdomys pumilio* | 257 | 2.571 | **0.020** | 3.140 | **0.001** |  | 0.065 | 0.474 | 0.629 | | 0.265 | 0 | 0 |
| *Spermophilus citellus* | 4 | 6.937 | **0.000** | 2.848 | **0.029** |  | 0.030 | 0.488 | -0.712 | | 1.000 | 0 | 0 |
| *Spermophilus suslicus* | 18 | 5.402 | **0.001** | 3.523 | **0.003** |  | 0.467 | 0.321 | 0.069 | | 0.472 | 0 | 0 |
| *Tupaia belangeri* | 28 | 2.628 | **0.020** | 2.544 | **0.007** |  | 1.470 | **0.046** | 1.721 | | **0.044** | 1 | 0 |
| ^a^ABS based on Brown *et al.* (1993) | |  |  |  |  |  |  |  |  | |  |  |  |
| ^b^ABS based on review from Bondinas *et al.* (2007) | | |  |  |  |  |  |  |  | |  |  |  |

**Table S3.** The subset of highly supported phylogenetic generalized least squares regression (PGLS) models (≥ 10% AICc weights of the top model) explaining the following dependent variables: log number of alleles, nucleotide diversity (π), dN/dS at ABS, and dN – dS at nonABS. Pagel’s λ was estimated using the rodent phylogeny (see Figure 3) and observed trait data and significance determined by likelihood ratio tests comparing models assuming the maximum likelihood estimate of λ to models assuming no phylogenetic signal (λ=0). All models included a significant intercept term and had full model *P* values < 0.05. Abbreviated terms are described as follows: dS: synonymous substitutions; dN: non-synonymous substitutions; ABS: antigen binding site. AICc weights (*w*) describe the normalized relative likelihood of the model, or the probability the model is the best, given the data and set of candidate models.

| **Models for Log number of alleles** | **AICc** | | **∆AICc** | ***w*** | **adjR^2^** | **P-value** | **λ** | **λ P-value** |
| --- | --- | --- | --- | --- | --- | --- | --- | --- |
| Population dynamics | | 19.63 | 0 | 0.69 | 0.40 | 0.01 | 0.00 | 1.00 |
| Log sample size + Population dynamics | | 21.89 | 2.26 | 0.22 | 0.41 | 0.02 | 0.00 | 1.00 |
| Number of DRB loci + Log sample size + Population dynamics | | 24.07 | 4.44 | 0.08 | 0.43 | 0.03 | 0.00 | 1.00 |
|  | |  |  |  |  |  |  |  |
| **Models for π (average nucleotide divergence)** | | **AICc** | **∆AICc** | ***w*** | **adjR^2^** | **P-value** | **λ** | **λ P-value** |
| Number of DRB loci + Log population size | | -65.43 | 0 | 0.76 | 0.52 | 0.00 | 0.36 | 0.48 |
| Number of DRB loci + Log population size + Log body mass | | -62.99 | 2.44 | 0.22 | 0.50 | 0.01 | 0.00 | 1.00 |
|  | |  |  |  |  |  |  |  |
| **Models for dN/dS at ABS** | | **AICc** | **∆AICc** | ***w*** | **adjR^2^** | **P-value** | **λ** | **λ P-value** |
| Log population size + Log body mass | | 61.25 | 0 | 0.97 | 0.42 | 0.01 | 0.00 | 1.00 |
|  | |  |  |  |  |  |  |  |
| **Models for dS-dN at nonABS** | | **AICc** | **∆AICc** | ***w*** | **adjR^2^** | **P-value** | **λ** | **λ P-value** |
| Number of DRB loci | | -30.4 | 0 | 0.81 | 0.32 | 0.00 | 0.00 | 1.00 |
| Number of DRB loci + Population dynamics | | -27.17 | 3.23 | 0.16 | 0.36 | 0.03 | 0.00 | 1.00 |

**Table S4.** Phylogenetic signal in rodent traits measured by Blomberg’s *K* and Pagel’s *λ*. Values of Blomberg’s K were estimated from continuous traits only using the *Picante* package of R, and significance was determined based on variance of phylogenetically independent contrasts relative to 1000 tip shuffling randomizations of trait values on the rodent phylogenetic tree extracted from the mammalian supertree (Bininda-Emonds et al. 2008). Pagel’s λ was estimated using the *Caper* package of R with significance determined by likelihood ratio tests comparing models assuming the maximum likelihood estimate of λ to models assuming no phylogenetic signal (λ=0). Significant *P*-values (α<0.05) are in bold.

|  | **Blomberg's *K*** | | **Pagel's *λ*** | |
| --- | --- | --- | --- | --- |
| **Trait** | ***K*** | ***P*** | ***λ*** | ***P*** |
| Number of DRB loci | 0.35 | 0.06 | 0.60 | 0.14 |
| Log population size | 0.21 | 0.31 | 0.00 | 1.00 |
| Log body mass(g) | 0.19 | 0.42 | 0.00 | 1.00 |
| Population dynamics | NA | NA | 0.92 | 0.23 |
| Bottleneck | NA | NA | 0.57 | 0.07 |
| Cyclic | NA | NA | 0.78 | 0.08 |
| Duplicated | NA | NA | **0.81** | **0.00** |
| Log sample size | 0.22 | 0.32 | 0.00 | 1.00 |
| dS at ABS | **0.50** | **0.02** | 0.64 | 0.09 |
| dS at nonABS | 0.34 | 0.07 | 0.21 | 0.68 |
| dN at ABS | **0.99** | **0.01** | **1.00** | **0.00** |
| dN at nonABS | 0.28 | 0.19 | 0.00 | 1.00 |

**Table S5.** Model-averaged estimates of the different parameters in the subset of models with high confidence (≥ 10% AICc weights of the top model), as well as the unconditional standard error, 95% confidence intervals, and importance. Importance describes the sum of the parameter weights in the subset of models in which the parameter is present. Parameter estimates with 95% CIs that did not cross zero are in bold.

**REFERENCES**

Axtner, J, Sommer S (2007) Gene duplication, allelic diversity, selection processes and adaptive value of mhc class ii drb genes of the bank vole, clethrionomys glareolus. *Immunogenetics*, **59**, 417-426.

Babik, W (2010) Methods for mhc genotyping in non-model vertebrates. *Molecular Ecology Resources*, **10**, 237-251.

Babik, W, Taberlet P, Ejsmond MJ, Radwan J (2009) New generation sequencers as a tool for genotyping of highly polymorphic multilocus mhc system. *Molecular Ecology Resources*, **9**, 713-719.

Faircloth, BC, Glenn TC (2011) Large sets of edit-metric sequence identification tags to facilitate large-scale multiplexing of reads from massively parallel sequencing. Available from Nature Precedings <<http://hdl.handle.net/10101/npre.2011.5672.1>> (2011).

Froeschke, G, Sommer S (2005) Mhc class ii drb variability and parasite load in the striped mouse (rhabdomys pumilio) in the southern kalahari. *Molecular Biology and Evolution*, **22**, 1254-1259.

Galan, M, Guivier E, Caraux G, Charbonnel N, Cosson JF (2010) A 454 multiplex sequencing method for rapid and reliable genotyping of highly polymorphic genes in large-scale studies. *BMC Genomics*, **11**, 296.

Harf, R, Sommer S (2005) Association between major histocompatibility complex class ii drb alleles and parasite load in the hairy-footed gerbil, gerbillurus paeba, in the southern kalahari. *Molecular Ecology*, **14**, 85-91.

Huse, SM, Huber JA, Morrison HG, Sogin ML, Welch DM (2007) Accuracy and quality of massively parallel DNA pyrosequencing. *Genome Biology*, **8**, R143.

Kearse, M, Moir R, Wilson A*, et al.* (2012) Geneious basic: An integrated and extendable desktop software platform for the organization and analysis of sequence data. *Bioinformatics*, **28**, 1647-1649.

Kimura, M (1980) A simple method for estimating evolutionary rates of base substitutions through comparative studies of nucleotide sequences. *Journal of Molecular Evolution*, **16**, 111-120.

Kloch, A, Babik W, Bajer A, SiŃSki E, Radwan J (2010) Effects of an mhc-drb genotype and allele number on the load of gut parasites in the bank vole myodes glareolus. *Molecular Ecology*, **19**, 255-265.

Longeri, M, Zanotti M, Damiani G (2002) Recombinant drb sequences produced by mismatch repair of heteroduplexes during cloning in escherichia coli. *European Journal of Immunogenetics*, **29**, 517-523.

Margulies, M, Egholm M, Altman WE, Attiya S, Bader JS, Bemben LA, Berka J, Braverman MS, Chen YJ, Chen Z (2005) Genome sequencing in microfabricated high-density picolitre reactors. *Nature*, **437**, 376-380.

Martin, DP, Lemey P, Lott M, Moulton V, Posada D, Lefeuvre P (2010) Rdp3: A flexible and fast computer program for analyzing recombination. *Bioinformatics*, **26**, 2462-2463.

Meglécz, E, Piry S, Desmarais E, Galan M, Gilles A, Guivier E, Pech N, Martin JF (2011) Sesame (sequence sorter & amplicon explorer): Genotyping based on high-throughput multiplex amplicon sequencing. *Bioinformatics*, **27**, 277-278.

Meyer-Lucht, Y, Sommer S (2005) Mhc diversity and the association to nematode parasitism in the yellow-necked mouse (apodemus flavicollis). *Molecular Ecology*, **14**, 2233-2243.

Oliver, M, Piertney S (2006) Isolation and characterization of a mhc class ii drb locus in the european water vole ( arvicola terrestris ). *Immunogenetics*, **58**, 390-395.

Oppelt, C, Wutzler R, von Holst D (2010) Characterisation of mhc class ii drb genes in the northern tree shrew (tupaia belangeri). *Immunogenetics*, **62**, 613-622.

Posada, D, Crandall KA (2001) Evaluation of methods for detecting recombination from DNA sequences: Computer simulations. *Proceedings of the National Academy of Sciences*, **98**, 13757.

Ronquist, F, Teslenko M, van der Mark P, Ayres DL, Darling A, Höhna S, Larget B, Liu L, Suchard MA, Huelsenbeck JP (2012) Mrbayes 3.2: Efficient bayesian phylogenetic inference and model choice across a large model space. *Systematic Biology*.

Saitou, N, Nei M (1987) The neighbor-joining method: A new method for reconstructing phylogenetic trees. *Molecular Biology and Evolution*, **4**, 406-425.

Schad, J, Sommer S, Ganzhorn JU (2004) Mhc variability of a small lemur in the littoral forest fragments of southeastern madagascar. *Conservation Genetics*, **5**, 299-309.

Zagalska-Neubauer, M, Babik W, Stuglik M, Gustafsson L, Cichon M, Radwan J (2010) 454 sequencing reveals extreme complexity of the class ii major histocompatibility complex in the collared flycatcher. *BMC Evolutionary Biology*, **10**, 395.
